# Quo vadis, smallholder forest landscape? An introduction to the LPB-RAP model

**DOI:** 10.1371/journal.pone.0297439

**Published:** 2024-02-02

**Authors:** Sonja Holler, Daniel Kübler, Olaf Conrad, Oliver Schmitz, Carmelo Bonannella, Tomislav Hengl, Jürgen Böhner, Sven Günter, Melvin Lippe

**Affiliations:** 1 Thünen Institute of Forestry, Hamburg, Germany; 2 Center for Earth System Research and Sustainability (CEN), Hamburg University, Hamburg, Germany; 3 Department of Physical Geography, Faculty of Geosciences, Utrecht University, Utrecht, The Netherlands; 4 OpenGeoHub, Wageningen, The Netherlands; 5 Laboratory of Geo-Information Science and Remote Sensing, Wageningen University & Research, Wageningen, The Netherlands; Sichuan University, CHINA

## Abstract

The impacts of the Anthropocene on climate and biodiversity pose societal and ecological problems that may only be solved by ecosystem restoration. Local to regional actions are required, which need to consider the prevailing present and future conditions of a certain landscape extent. Modeling approaches can be of help to support management efforts and to provide advice to policy making. We present stage one of the LaForeT-PLUC-BE model (Landscape Forestry in the Tropics–PCRaster Land Use Change–Biogeographic & Economic model; in short: LPB) and its thematic expansion module RAP (Restoration Areas Potentials). LPB-RAP is a high-resolution pixel-based scenario tool that relies on a range of explicit land use types (LUTs) to describe various forest types and the environment. It simulates and analyzes future landscape configurations under consideration of climate, population and land use change long-term. Simulated Land Use Land Cover Change (LULCC) builds on dynamic, probabilistic modeling incorporating climatic and anthropogenic determinants as well as restriction parameters to depict a sub-national regional smallholder-dominated forest landscape. The model delivers results for contrasting scenario settings by simulating without and with potential Forest and Landscape Restoration (FLR) measures. FLR potentials are depicted by up to five RAP-LUTs. The model builds on user-defined scenario inputs, such as the Shared Socioeconomic Pathways (SSP) and Representative Concentration Pathways (RCP). Model application is here exemplified for the SSP2-RCP4.5 scenario in the time frame 2018–2100 on the hectare scale in annual resolution using Esmeraldas province, Ecuador, as a case study area. The LPB-RAP model is a novel, heuristic Spatial Decision Support System (SDSS) tool for smallholder-dominated forest landscapes, supporting near-time top-down planning measures with long-term bottom-up modeling. Its application should be followed up by FLR on-site investigations and stakeholder participation across all involved scales.

## 1. Introduction

A range of policy goals have emerged in recent years to tackle the challenges posed by the Anthropocene such as climate change [1] and the loss of biodiversity by deforestation [2]. These goals underscore the need for collaborative efforts to address the conflicts arising from limited land resources and their associated ecosystem services. This narrative has been supported by initiatives such as the United Nations’ "Decade on Biodiversity" (2011⁠–2020), the ongoing "Decade on Ecosystem Restoration" (2021⁠–2030), and related global restoration commitments like the "Bonn Challenge" led by the United Nations Environment Program.

The concept of Forest and Landscape Restoration (FLR) has gained prominence as an important management strategy to restore ecological functionality and enhance human well-being in deforested or degraded landscapes [3]. FLR encompasses however not only the restoration of forests but also other ecosystems within a landscape mosaic. Many nations have pledged substantial areas for restoration following the FLR paradigm [4]. Globally, estimations of the potential area for forest and tree restoration, as well as on specific FLR activities, vary significantly [5–7], while the feasibility of implementing large-scale FLR commitments on the ground remains uncertain [8].

A significant portion of forest restoration opportunities can be found in smallholder-dominated forest landscapes. While smallholders inhabit approximately 33% of forest landscapes globally [9], balancing the needs of biodiversity conservation, climate change mitigation, ecosystem services provision, and rural development in these landscapes is an ongoing challenge [10, 11]. For this case, FLR, with its emphasis on multifunctionality, presents an attractive policy option to approach this challenge. However, information regarding the potential for FLR in smallholder-dominated forest landscapes remains limited which is particularly true for the Global South and tropical environments in general.

Modeling approaches are valuable tools for assessing restoration opportunities in such contexts while considering smallholder needs and their impacts on forest landscapes. Dynamic and spatially explicit models have proven useful to support management decisions and have been utilized in Spatial Decision Support Systems (SDSS) [12]. Such models can capture future scenarios by incorporating the uncertainty inherent in management choices. Investigating the feasibility of restoration options as part of future FLR necessitates long-term simulations that cover projected population peaks and/or potential peak demands of land uses. This is important, as policy-making requires information on how future forest landscape settings may unfold and what implications this has on environmental management and FLR. Land Use Land Cover Change (LULCC) models, such as CLUEs [13, 14] or PLUC [15, 16] have been developed to simulate future landscape configurations. But despite the availability of diverse LULCC modeling tools [17], limitations remain in LULCC modeling for capturing forest disturbance regimes and succession stages [18]. Both are particularly relevant for the case of forest landscapes and FLR. The representation of forests in aggregated forms, as commonly done in many LULCC models, hampers decision-making for forest conservation and restoration.

A further challenge refers to the consideration of global and local scales in a single simulation approach [19]. On the one hand, the Anthropocene makes it eminent to incorporate climate change and its regional patterns into LULCC modeling, while on the other hand, local management decisions and land use choices need to be considered, too. To date, climate change patterns are still often excluded from LULCC models, although they are of particular importance at finer scales such as landscapes to align with the management areas of smallholders and the goals of FLR.

Against this background, we developed a new modeling approach to support environmental management and policy decision-making processes based on long-term scenario simulations with a particular focus on smallholder-dominated forest landscapes. The approach builds on two conceptual pillars: (1) Forest and Landscape Restoration (FLR) [3], and (2) the estimation of potentials for FLR in simulated future landscapes (further referred to as: “Restoration Areas Potentials”—RAP).

The novel modeling approach integrates deterministic and stochastic functions within a dynamic and spatially explicit cellular automata model inheriting features of the PLUC model. The newly developed model relies like PLUC on the PCRaster Python Monte Carlo framework for probabilistic modeling [20]. In contrast, it focuses on subnational smallholder-dominated forest landscapes at the hectare scale, employing up to 18 explicit land use types (LUTs) and up to five additional RAP-LUTs. By simulating and analyzing future LULCC patterns and deriving RAP and its potential impact, our model provides insights into feasible restoration measures while considering population dynamics, climate change and land use patterns. This approach can be of particular benefit for short- to long-term policy design and decision-making processes for sustainable landscape restoration in the Anthropocene.

The presented study is part of the LaForeT project (http://la-foret.org/), which conducted household surveys and forest inventories in 36 landscapes of Ecuador, Zambia and the Philippines between 2016 and 2019 (12 per country, each landscape covering approximately 10x10 km). Project phase 2 (LaForeT-R^2^) used this database for studies focusing on Restoration & REDD+. Our model was developed in the LaForeT-R^2^ context using the Esmeraldas province in Ecuador as a case study for model development. Here, we focus on the presentation of the newly developed modeling tool (LPB-RAP) and present outcomes for the comparison of future forest landscapes with and without FLR measures. Our model is versatile and can simulate different types of scenarios by changing input maps, time series and parameter settings. Its open-source PCRaster Python-based coding approach allows for further model developments or for coupling it with other modeling tools. LPB-RAP relies on a diverse set of data sources to parameterize and calibrate the model, including primary data, to specific scenario assumptions.

For the purpose of this model development study, we present in section 2 the underlying structural skeleton of the LPB-RAP model. This is supported by supplemental materials S1 File, which introduces further background information and further newly incorporated and applied methods. Section 3 showcases modeling results for the case study area of Esmeraldas province in Ecuador (see S2 and S3 Files for case study parametrization and S4 File, as well as S5 File forfurther results) to highlight the model’s versatility as a scenario-driven simulation tool for studying smallholder-dominated LULCC and RAP in forest landscapes. The Esmeraldas province is used because it has been under high deforestation pressure in the recent past, but also has several conservation zones [21], which amounted to 414,987 ha or 24.72% of the simulated landscape (see section 2.3 in S2 File and section 2.4 S4 File) in 2016. The province is characterized by smallholder-driven agricultural production and has been identified as a target for FLR by the Ecuadorian government [22, 23]. In section 4, we discuss internal and external model plausibility as well as the potential caveats and limitations of LPB-RAP. Section 5 provides conclusions regarding the new modeling approach and gives an outlook for tentative further model development stages.

## 2. Materials and methods

In this section, we first introduce the LPB-RAP modeling approach (section 2.1). We then describe essential new model components for extended landscape and LULCC simulation: determinants for long-term simulations (section 2.2), policy enforcement levels and related simulation choices (section 2.3), and allocation procedures (section 2.4).

LPB-RAP, including a detailed manual and the required data, is available open-source via GitHub (see S5 File). Open-source software used comprises SAGA GIS (version 8.0.0+), QGIS (version “Hannover”+), PCRaster (version 4.3.3+), Python (version 3.9+) and R (version 4.2.0+).

### 2.1 LPB-RAP modeling approach

This section provides a short overview of the major commonalities and differences between the base model PLUC [15, 16] and LPB-RAP, followed by a short introduction of the new main model features.

#### 2.1.1 LPB-RAP & PLUC

At the core, LPB-RAP and PLUC both refer to probabilistic LULCC-models that provide information on application-specific area potentials in simulated future landscape configurations. The original PLUC model [15, 16], used in case of LPB-RAP as a base model, simulates future area potentials for bioenergy crops that most likely will not be in competition with food production. PLUC was selected as a base model because its modeling design approaches bottom-up land use systems instead of top-down conditions, which is also often the case in smallholder-dominated forest landscapes. On the contrary, LPB-RAP estimates RAP in the context of introducing FLR measures to a landscape. It is designed as a scenario tool for long-term simulations covering demands for the population peak or for the particular year of the land peak demands. It offers the user to determine area potentials that are available in the short and long term. Both models allocate area demand in a user-defined order of primary active LUTs and abandoned, deforested or harvested LUTs (here referred to as secondary active LUTs). Allocation is determined by suitability maps, which serve as drivers of change. In LPB-RAP, suitability factors are current land use and its direct 3x3 neighborhood pixels, slope inclination, distance to street network, distance to surface freshwater network, and distance to cities and settlements.

Altogether, LPB-RAP inherited from PLUC: (1) an open-source approach implemented in Python and embedded in the PCRaster Python framework; (2) the reliance on a Monte Carlo framework; (3) expressing land use patterns during a timestep *t* by anthropogenic land use area demands (in LPB-RAP: built-up, food and wood); (5) excluding user-defined areas such as protected and topography-specific areas from a simulation if parametrized; (6) requiring only a single LULC map to initiate a model run due to its character of a scenario tool.

While PLUC provides uncertainty information only for a particular focus aspect (i.e., potentials for bioenergy crops) across all samples of different landscape trajectories, in contrast, LPB delivers uncertainty information for the entire aggregated landscape. This new feature supports the user in understanding simulated landscape configurations in a more coherent fashion and investigating areas of interest in their spatial context. For further information, see also section 3.1.

Many new model methods, parameters and assessment features were implemented in LBP-RAP targeting long-term simulation in a fine-scale spatial resolution, especially relevant for the consideration of smallholder-dominated forest landscapes. Section 2.1.2 describes the most important new main model features (long-term simulation approach, footprint approach, explicit LUTs, differentiated forest dynamics simulation) while further details can be found in the prelude section of S1 File. Table A1 in S1 File lists major components of both models as well as their commonalities and differences. The same table also provides references to sections where more information about LPB-RAP can be found.

#### 2.1.2 LPB-RAP main model features

The following subsections introduce main model features of model stage 1, including outlooks on further conceptualized development stages; for a brief description see also section 5.

### LPB-RAP model structure

**Fig 1:**
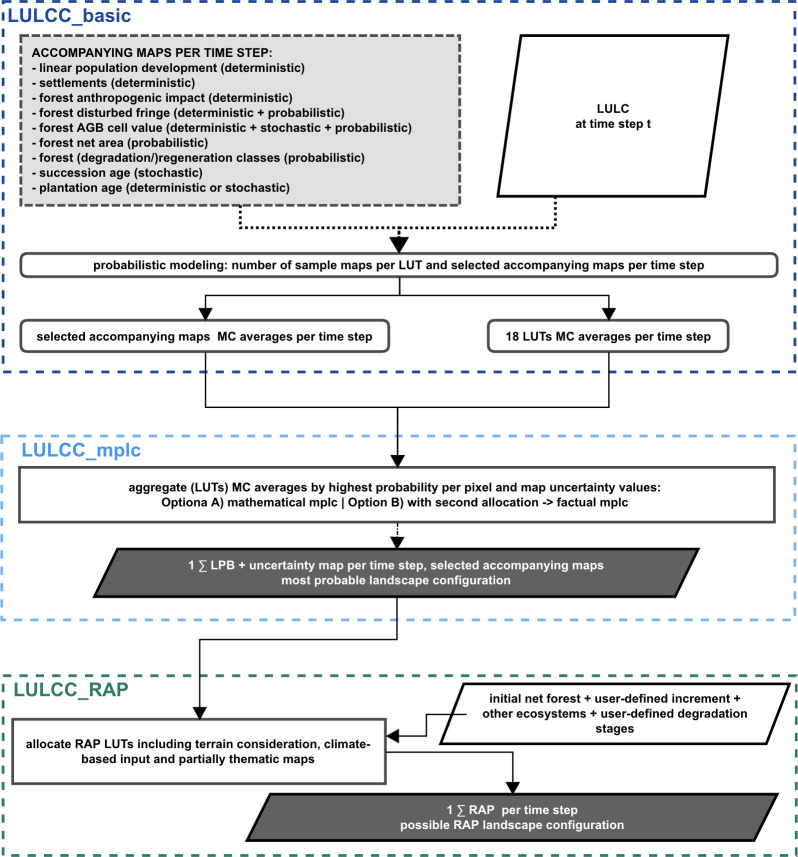
LPB-RAP model flowchart. The LPB-RAP model flowchart depicts the major simulation steps and outputs within the user-defined baseline and guideline scenario setting. The basis of the new model design is the aggregation and re-interpretation of probabilistically simulated datasets of the general landscape (LULCC LUTs Monte Carlo averages) and selected thematic aspects (accompanying maps). The three main modules (LULCC_basic, LULCC_mplc, LULCC_RAP) are executed consecutively, i.e., each module simulates all time steps (and in LULCC_basic all samples) before the next module is applied. For details regarding the allocations in modules LULCC_basic, LULCC_mplc and LULCC_RAP, see Figs 2 to 4.

LBP-RAP comprises three modules of LULCC simulation: LULCC_basic functions as the basic probabilistic simulation module, LULCC_mplc as an aggregating module (mplc stands for „most probable landscape configuration”) and LULCC_RAP as an interpretation module (see Fig 1).

**Fig 2:**
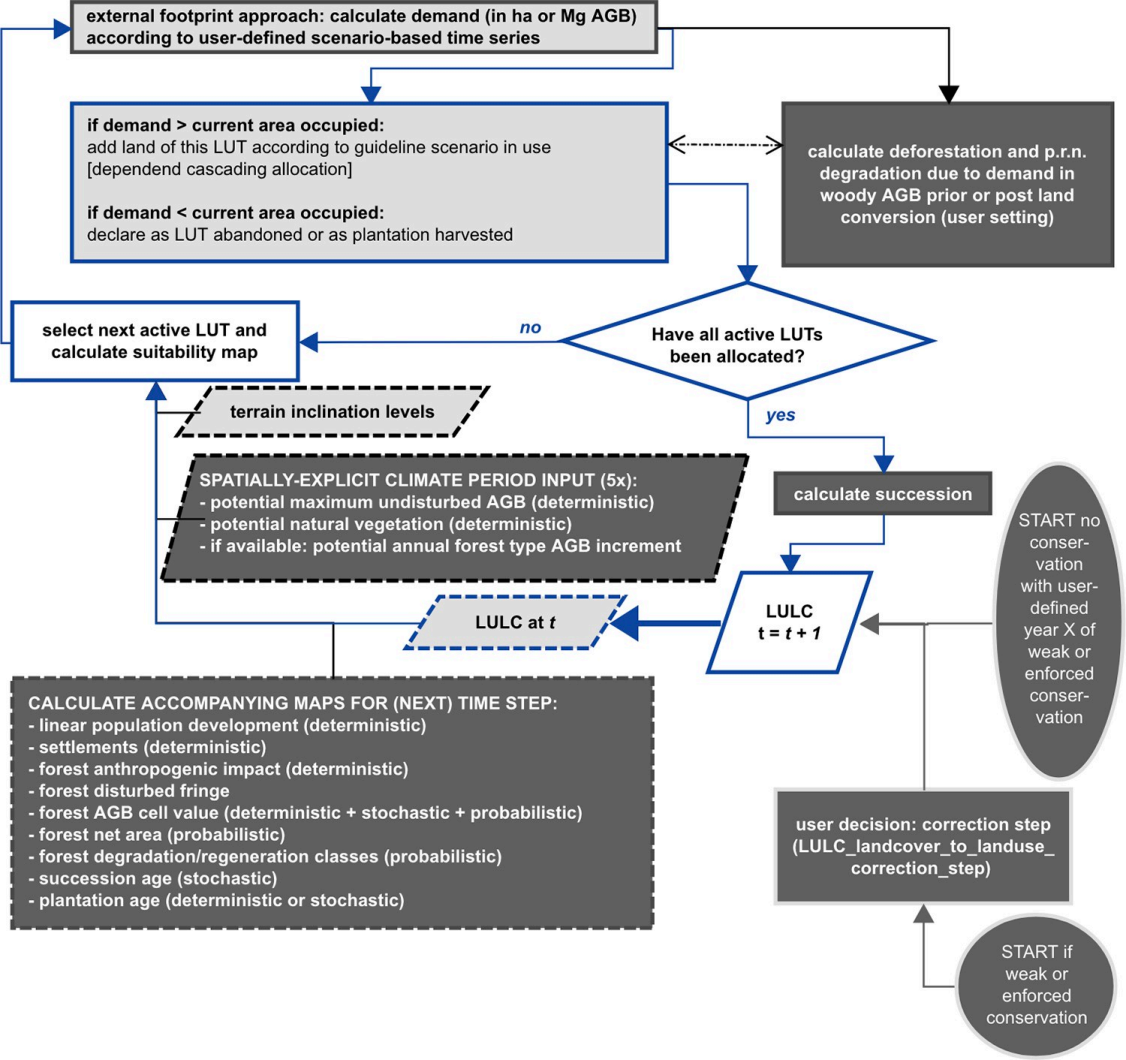
LPB-RAP module LULCC_basic allocation procedure. The flowchart presents the LULCC_basic allocation based on the redesigned PLUC model engine’s basic functionality (blue). The extended LPB-RAP concept includes a variety of adapted (light grey) or new (dark grey) simulation components. These are new inputs and accompanying maps, allocation rules per guideline scenario and user settings, as well as differentiated succession modeling based on the provided baseline scenario information. The cascading allocation for a restriction policy measure in LULCC_basic is steered within the available landscape extents, LUTs and slope classes (restricted and excluded areas, excluded LUTs and inaccessible terrain cannot be used). Accessible terrain is differentiated into favorable and difficult terrain. Cascading allocation refers to coding where the next level of accessible terrain, i.e., the next pool of available cells, is only used if there is still unsatisfied demand. The cascading allocation for weak conservation is: (1) favorable terrain outside restricted areas, (2) difficult terrain outside restricted areas, (3) favorable terrain inside restricted areas, and (4) difficult terrain inside restricted areas. For enforced conservation, the allocation excludes restricted areas, thereby the allocation occurs in the order (1) favorable terrain outside restricted areas, (2) difficult terrain outside restricted areas. For the no conservation scenario, the cascading allocation is partitioned into (1) favorable terrain landscape-wide and (2) difficult terrain landscape-wide.

**Fig 3:**
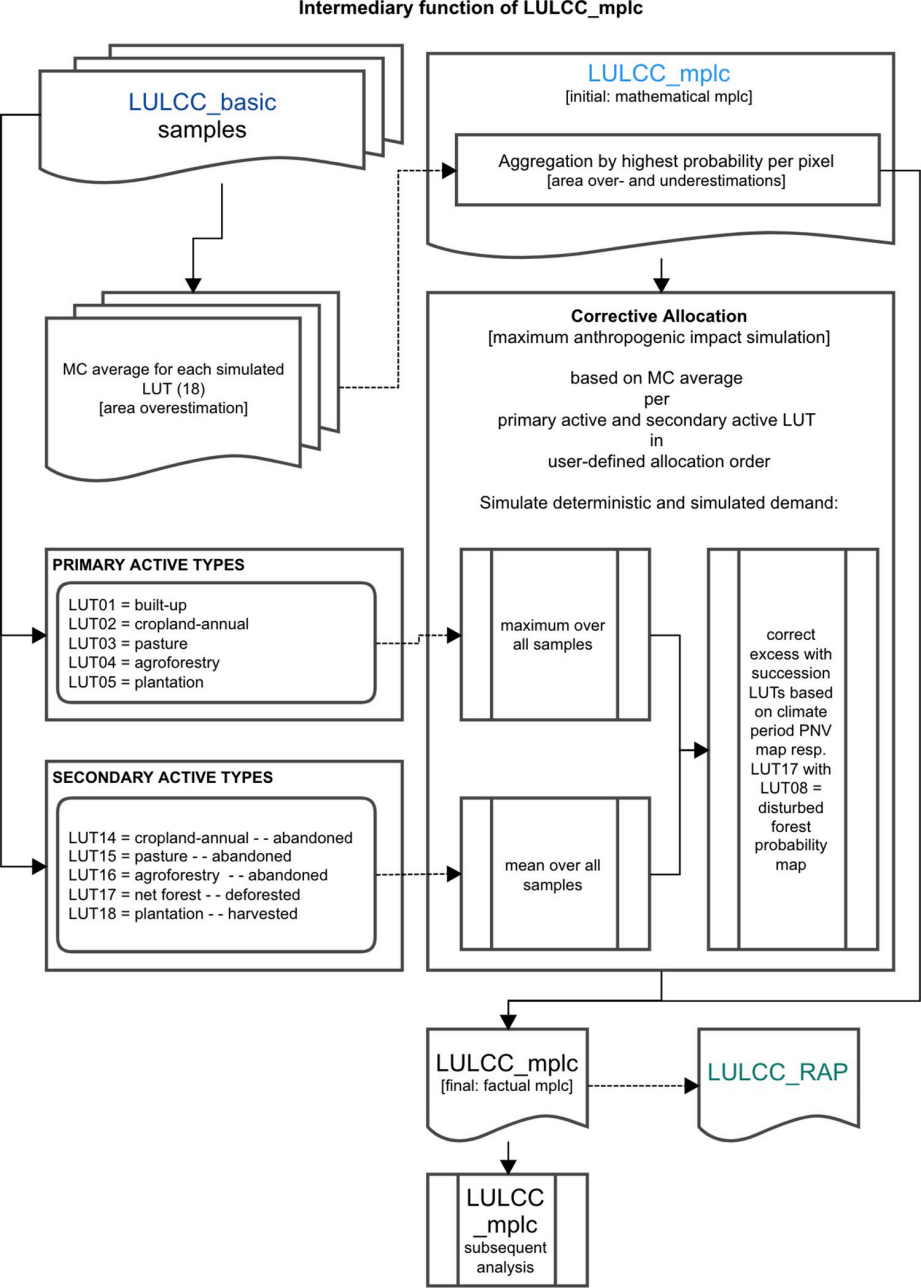
LPB-RAP module LULCC_mplc allocation procedure. The diagram displays the essential components of the LULCC_mplc procedures to approximate the discrete anthropogenic impact during a simulation (factual mplc) within scenario assumptions based on prior derived probabilities (Monte Carlo, short: MC, averages) and aggregated results (mathematical mplc) per time step. In LPB-RAP, this module serves as an intermediary step to provide the basis for possible landscape configurations of potential FLR as simulated in LULCC_RAP. This is based on the probable landscape configurations in the factual mplc calculation simulated in LULCC_mplc.

**Fig 4:**
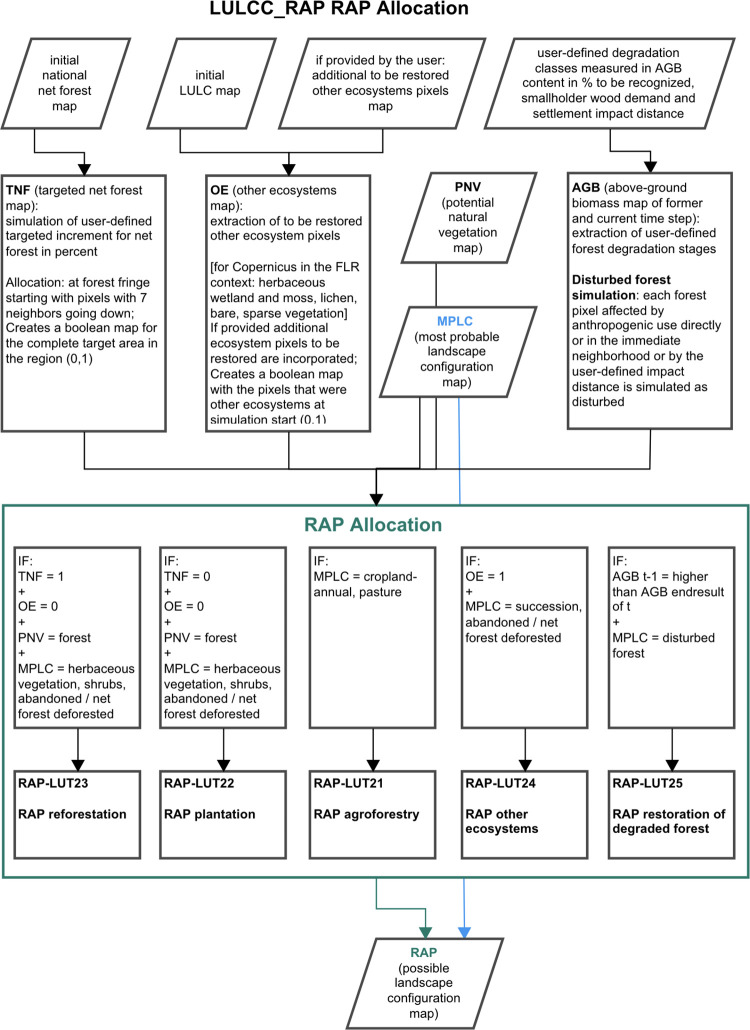
LPB-RAP module LULCC_RAP allocation procedure. The diagram shows the RAP allocation procedure within the landscape previously simulated in LULCC_mplc per consecutive time step. The algorithm procedure is additionally steered by the user in the form of the user-defined targeted net forest increment in percent and the defined additional other ecosystem pixels map, besides user-defined and user-selected degradation classes to be simulated with RAP-LUT25. Targeted net forest and other ecosystems input maps derived from the initial simulation year depict the target status which should be restored (e.g., incorporating a 3% increase for net forest extents). To incorporate the potential climate change impact element, the calculation is conducted for each time step according to the current drawn potential natural vegetation map. Note that this approach does not simulate afforestation for non-forest biome pixels.

We implemented several innovations in LPB-RAP, which expanded model code and algorithm functionality (for a comprehensive overview see prelude in S1 File).

### New main model features

For the *long-term simulation approach*, LPB-RAP features spatially explicit annually interpolated population data and climate-based datasets on a twenty year average. The latter were derived from five climate periods that serve as guiding posts for the long-term simulations until 2100. In its present form, LPB-RAP can simulate various scenario combinations of SSP/RCP scenario data, consideration of restricted areas in different scenario environments, and gradual simulations of terrain parameters and regional systemic simulation choices. The model can be adjusted to a diversity of parametrized initial conditions and scenario variations depending on the scope of the study and region.

For the depiction of land use dynamics, LPB-RAP can, besides the basic PLUC demand/yield approach, utilize a newly developed *land footprint per smallholder capita approach*, similar to the footprint approach as depicted by Kastner and Nonhebel, 2009 [24], and Laroche et al., 2019 [25]. For the purpose of LPB-RAP, the derivation of the agricultural footprint per time step was adapted for the use of cross-sectional household survey data and scenario-based population data for the initial simulation year and scenario assumptions for the consecutive time steps. This allows to incorporate assumptions on impacts of the underlying macroeconomic scenario development, such as kilocalorie intake per person and future diet patterns of a society (see section 1.6.2 in S1 File and S3 File). This approach holds several advantages. First, a regionally derived mean footprint per agricultural LUT incorporates different plot conditions, cultivated crop types and livestock options and can thereby capture heterogeneous conditions in the landscape. Second, footprints derived from actual or recent primary data describe the potentially prevailing long-term land area demands more realistically than building on the assumption of static potential maximum yield of a singular chosen crop type as implemented in PLUC. The latter one provides also no option to consider climate-influenced yield increase or decrease long-term. The application of climate period-based potential yields is implemented in the LPB-RAP source code as a dynamic suitability factor to expand the repertoire of simulation choices for long-term simulations based on initial time series data. As such time series were not available yet, this functionality is not featured in this study, which is based on the application of the footprint approach. Third, the footprint approach enables a significantly reduced model run time. As the land footprint approach does not require suitability factors for crop yields or cattle density, as used in the original PLUC model, these were omitted in LPB-RAP for the footprint approach. For the generic footprint derivation and calibration to scenario assumptions, the reader is referred to section 1.6 in S1 File., for case study parametrization to sections 1.1 and 3.1.12 in S2 File, and for explicit calculations to S3 File.

For the depiction of the entire landscape, LPB handles *explicit LUTs* while maintaining granular high-resolution at the hectare scale instead of aggregated mosaic LUTs, as for example done for the case of PLUC and its considered crop LUTs. Therefore, LPB-RAP comprises n = 23 LUTs, divided into 18 basic LUTs and up to five RAP-specific LUTs (see Table A3 in S1 File for the available basic LUTs and section 2.5 in S2 File for their further descriptions in the context of the applied case study). Due to the generic approach of LPB for global applications, the model was optimized for using modified Copernicus land cover-based maps [26] as initial spatial input.

To enhance the *differentiated simulation of forest landscapes* the user can further parameterize (forest) succession stages and succession rules (see section 2.2 in S1 File and section 3.1.10 in S2 File for further explanations).

### 2.2 Determinants for long-term simulations

The deterministic simulation part of LPB-RAP builds on spatially explicit datasets of climate data-based and population scenario projections as well as scenario-based long-term time series projections of land use until 2100. These projections provide the underlying long-term guiding posts, or baseline scenario, for the dynamic simulation of landscape and land use change. Datasets can be substituted for completely diverging scenarios or only gradual variations depending on user needs.

We refer to sections 2.7, 2.8 and 3.1.12 in S2 File for further information on the applied SSP2-RCP4.5 scenario assumptions in the case study context of the Esmeraldas province.

#### 2.2.1 Climate

Incorporating long-term climate data-based projections is one key innovation in LPB-RAP compared to the base model PLUC. The climate-based determinants serve as the scenario-based guiding posts for simulating future landscape development. They steer and limit the simulation of succession and maximum woody above-ground biomass (AGB) development, as described in the following sections.

#### Potential natural vegetation

Building on climate reference data and projections, *potential natural vegetation* (PNV) per climate period was computed to derive recent and potential future biome distributions. We rely on ensemble-based stacked model generalizations [27] to combine predictions made by level 0 models and use them as training data for a level 1 model. For a more detailed description of the computation framework and validation strategy, see Bonannella et al., 2022 [28]. The original work from Hengl et al., 2018 [29], was limited to current potential biomes distributions based on observed predictor variables (FAPAR and other remote-sensing predictor variables). For our study, the estimates of current and future potential biomes distributions are based on climatic and bioclimatic variables from bioclimatic layers and static predictor variables such as elevation, slope and aspect (see section 3 in S1 File and section 2.8.1 in S2 File for further details). PNV estimates are the basis for succession simulation in LULCC_basic (see section 2.4.1 for further details). They are further used in the corrective allocation of the factual mplc in LULCC_mplc (section 2.4.2) and support the definition of Restoration Areas Potentials in LULCC_RAP (section 2.4.3).

#### Potential maximum undisturbed woody AGB

We estimated the *potential maximum undisturbed forest AGB* as an approximation to the climax stadium to not overestimate woody AGB dynamics in the forest-related LUTs. For this, we combined the ESA biomass dataset version 3 for 2018 [30], henceforth: “ESA AGB V3”, with the „undisturbed forest”information of the „Tropical Moist Forest”dataset [31], henceforth “TMF”. We then used these datasets to estimate the potential maximum undisturbed AGB for the case study area for the applied climate reference period and projected it with climate period scenario information (see section 2.8.2 in S2 File).

#### Potential annual AGB increment

The current model design can accommodate maps of annual increments per selected potential forest type (undisturbed, disturbed, plantation) to enable a spatially explicit approximation of AGB development. The model is conceptualized to use ESA AGB V3 or similar high-resolution data if available. This could currently not be realized for the Esmeraldas region due to quality issues of the increment from 2017 to 2018 within the ESA AGB V3 data (quality flag 3, i.e., “improbable change“). Accordingly, for this study, we solved the simulation of AGB development based on a stochastically derived increment between values reported in the literature (see section 2.2.5 in S1 File and section 3.1.4 in S2 File for further details). For the case of LUT agroforestry, this was already simulated stochastically within the range of the disturbed forest minimum and maximum values because this LUT can potentially depict a broad range of different systems and development stages in space and time during the simulation.

#### Potential yields (forthcoming study)

For the long-term simulation within the PLUC demand/yield approach, it is necessary to also provide potential yields per simulated climate period of representative crops and livestock density per agricultural LUT to describe potential land use development. This feature is already implemented in the provided LPB-RAP model and will be subject to a forthcoming publication but is not further described here.

#### 2.2.2 Population

The second component of the underlying baseline scenario is a SSP projection of long-term population development [32, 33]. This data is available in a downscaled 1 km^2^ resolution and separated into *total*, *rural* and *urban* categories. We obtained decadal datasets for 2010 to 2100, harmonized them to a 100 m resolution and computed a linear interpolation for each year and each pixel. Accordingly, the population is implemented in LPB as a dynamic suitability factor. In contrast, PLUC uses a singular population dataset as a static suitability factor for short-term simulations.

#### 2.2.3 Land use

The third pillar of the user-defined baseline scenario refers to the land use component of the presented dynamic footprint approach. For each simulation, land use is depicted by the actively simulated LUTs that represent anthropogenic demands in built-up area (representing streets and building structures in LPB-RAP), agricultural land and demand in woody AGB (see section 1.6 in S1 File for an overview of all components). For long-term simulations and subnational regional modeling, global narratives may have to be adjusted to fit the regional context and current trends (see for the implementation example differences between the global and regional SSP2 narrative in section 3.1.12 in S2 File Table B13 in S2 File). The resulting projected demands for a particular primary active LUT are allocated based on suitability maps within LPB-RAP. The resulting landscape configuration for a time step *t* is thereby an interplay of methods as depicted in S1 File and the configured time series of demand (see S3 File). Subsequently, land use allocation incorporates inter alia the newly developed dynamic settlement algorithm (see section 2.3.4 in S1 File), the guideline scenario allocation rules (i.e., how restricted areas can be used, see section below) and the terrain inclination levels (see section 2.3.5 in S1 File).

### 2.3 Restriction policy enforcement levels

Three forms of potential policy measures and assumptions about underlying pressure aspects can be simulated with LPB-RAP, all of which display plausible *what*-*if* scenarios in their own sense. This conceptual scenario level steers the distribution of anthropogenic land use demands by the definition of available areas. Therefore, the location of restricted conservation areas in the case study region is required as model input. Restricted areas are treated differently in the three implemented scenarios.

#### 2.3.1 Weak conservation

Scenario *weak conservation* depicts land use change in restricted areas when land-use demands cannot be satisfied in favorable terrain (i.e., more gentle slopes) or difficult terrain (i.e., steeper slopes) in unrestricted areas. It uses a four-step procedure of a cascading allocation per time step. This scenario assumes that restricted areas are respected by the population as long as unrestricted land is available. The model will record unallocated demands if they occur despite the usage of restricted areas for user information purposes.

#### 2.3.2 Enforced conservation

Scenario *enforced conservation*, on the contrary, simulates enforced protection of restricted areas. Land use expansion to and logging in protected areas in this setting is strictly prohibited. Land use is only simulated until land outside restricted areas is available. Any unallocated land demands are registered as an indicator for potential transregional leakage or food security risk.

#### 2.3.3 No conservation

Scenario *no conservation* allows land use expansion in restricted areas, thus also potentially amplifying pressure on forests located within such areas. It assumes that such restricted areas are either repealed or ineffective. The scenario is implemented as part of model parametrization, based on a user-defined time step X ≥ initial simulation year of a *weak conservation* or *enforced conservation* simulation run. The scenario is realized in a two-step procedure of first considering favorable terrain use and second by the opportunity to expand land use on difficult terrain for the entire case study area. By this design, land use is allocated until the topographic boundary conditions (e.g., for user-defined inaccessible slopes of >45%) for the considered simulation area are reached.

### 2.4 Allocation procedures

This section introduces the allocation procedures considered in the three consecutively executed modules of LPB-RAP.

#### 2.4.1 Probabilistic modeling of projected landscape configurations

LULCC_basic offers the basic functionality of the PLUC model, i.e., the dynamic modeling of LULCC based on suitability factors and anthropogenic demands. It further calculates probabilities based on the embedded PCRaster Monte Carlo framework for all employed basic LUTs and further thematic aspects. Within LPB-RAP, the PLUC base model was substantially expanded to allow for the simulation of all new simulation targets (see Fig 2).

For more detailed information, the reader is referred to prelude information in S1 File

#### 2.4.2 No FLR scenario: Aggregated landscape configuration

The second module is an extension of the module LULCC_basic. LULCC_mplc aggregates the simulated probabilistic outputs by the highest probability per pixel to derive one discrete landscape configuration for each time step. These landscape configurations can also be considered as a standalone result if the focus is on probabilistic simulations (mathematical mplc). For the simulation of RAP within the module LULCC_RAP, however, it is required to run LULCC_mplc with a corrective allocation (factual mplc within scenario assumptions) based on the *a priori* derived probability maps per LUT (Monte Carlo averages per primary or secondary active LUTs) to discretely simulate the provided and simulated anthropogenic demands (see Fig 3).

Subsequently, the module runs an extensive analysis on the mplc landscape configuration (depending on the user’s choice using the mathematical or the factual mplc), deriving 300+ variables of different thematic categories per time step (including simulated land use area, landscape share, and a variety of pressure and forest condition aspects; for full scope, see mplc log-file in S5 File). Further information is provided in the prelude section of S1 File.

#### 2.4.3 Potential FLR scenario: re-interpreted landscape configuration

The LULCC_RAP module is the final calculation step in the LPB-RAP modeling framework and serves both as a form of impact assessment and as a scenario of its own: first, the mplc landscape is evaluated and area potentials according to the land use impact are calculated, i.e., the derivation of potential FLR measures in accordance with the simulated land use; second, the module suggests RAP-LUTs which follows an ecological restoration context when considered as a scenario. These RAP-LUTs describe by intent overarching land use categories, as the model has no information to depict the FLR potential in greater detail, such as the description of specific agroforestry or timber plantation systems. LULCC_RAP provides estimates that potentially would result from FLR implementation (65+ variables are derived additionally for the resulting log-file, see RAP log-file in S5 File). To do so, the LULCC_RAP module evaluates the most probable landscape configurations derived from LULCC_mplc for each time step. LULCC_RAP uses the mplc information to identify available areas for restoration purposes after all anthropogenic demands are satisfied. Area availability is simulated by three different approaches with diverging algorithm procedures: RAP maximum, RAP minimum, and RAP suggested additional restricted areas (see for further information below). Caution has to be taken as annual outputs of LULCC_RAP cannot be interpreted as a sequence of implemented restoration measures over a period of *t*+n but instead represent potential entry points for future FLR projects.

Aside from the suggested conversions of LUTs cropland-annual and pasture to agroforestry, LUT21–LUT24 (RAP agroforestry, RAP plantation, RAP, reforestation, RAP other ecosystems) evaluate pixels that lost their initial ecosystem condition (forest or other ecosystems) while RAP-LUT25 describes the Restoration Area Potential of degraded but not entirely deforested forest pixels. LULCC_RAP allocates the module-specific LUTs and considers pixels which are not simulated under certain land use any longer (RAP other ecosystems, RAP reforestation, RAP plantation) or qualify for adaptation measures (RAP agroforestry, RAP restoration of degraded forest) and thereby become available for restoration purposes (see Fig 4 and section below).

Of importance in this case is the total available area which is defined as suitable for restoration by LULCC_RAP after anthropogenic land use needs are satisfied (LULCC_mplc). RAP-LUT25 (RAP restoration of degraded forest) in contrast evaluates pixels which are still considered as forest by the model. For more detailed information regarding RAP-LUT25 see prelude in S1 File.

### RAP maximum

The first algorithm in the module LULCC_RAP evaluates the entire modeled study area following a broader perspective of FLR towards Landscape Restoration [34]. This includes the rehabilitation of different ecosystems aiming to foster multifunctionality at the landscape-level. Therefore, this module operates from a geographically-based (i.e., via integration of biosphere [by use of potential forest biome pixels] and anthroposphere [which areas are occupied by current demands?] aspects), and regional landscape perspective (how is the initial and current landscape configured?), acknowledging common restoration goals of different stakeholders in the conceptualized LUTs. The allocation of the RAP-LUTs is simulated without competition between these RAP-LUTs (see Fig 4). This setting was chosen instead of simulating multiple scenarios of singular stakeholder goals which may lead to competing claims and overlapping areas of interest. The module simulates the derivation of the maximum restoration potential of the five module-specific LUTs (RAP agroforestry, RAP plantation, RAP reforestation, RAP other ecosystems, RAP restoration of degraded forest) as suited for the context of smallholder-dominated forest landscapes (see Table 1).

**Table 1:** Rationale of LULLC_RAP-specific LUTs suggested for envisioned landscape transformation in the FLR context (building on Temperton et al., 2019, in the dynamic LULCC landscape modeling framework for a general smallholder-dominated forest landscape).

RAP LUT	Envisioned realization	Envisioned tree cover outcome	Envisioned forest site quality outcome	Suggested on climate-period-based biome information and initial targeted net forest	Envisioned goals achieved
**RAP-LUT21 agroforestry**	rehabilitation efforts by use of fruit or fertilizer trees in combination with cropland or pasture systems (any kind of animal or husbandry) or other agroforestry systems	> 0% to approx. 70% depending on per plot targeted agroforestry system to sustain yields (equivalents)	minor to medium (envisions some forest site qualities, e.g., for soil parameters)	Independent from underlying biome and targeted net forest information as areas remain under land management within scenario assumptions.	This LUT is conceptualized as a compromise between mitigation and production goals.
**RAP-LUT22 plantation**	reforestation efforts by use of sustainable multifunctional mid- to long-term rotation periods mixed tree species for timber plantations	≥ 70%	high (resembles secondary forest naturally developed)	Only suggested on forest biome pixels where net forest is not targeted.	This LUT is conceptualized as a compromise for economically used plantations which resemble natural potentials under Sustainable Forest Management until harvest to combine the restoration targets of mitigation and production (in time limited potential).
**RAP-LUT23 reforestation**	reforestation efforts by passive or active reforestation with the goal of a status of almost all recovered primary forest traits	≥ 70%	full (resembles almost primary forest)	Only suggested on forest biome pixels where net forest is targeted (resembling forest characteristics at terrestrial surface level at timestep t+n).	This LUT envisions the combined restoration goals of forest (habitat) re-establishment and mitigation, hence, simulated on sites connected to the initial net forest extent or located within targeted net forest.
**RAP-LUT24 other ecosystems**	Landscape Restoration efforts, which denote, for example, the potential need for the recreation of riverbanks and other small ecosystems for Landscape Restoration, including full functionality and biodiversity. The LUT incorporates target pixels up to succession or net forest deforested, as the conversion for other ecosystem pixels cannot be distinguished. This application covers, for example, the case that a pixel of herbaceous wetland is converted to agricultural land use (e.g., by drainage), was abandoned and is in succession to undisturbed forest. The initial landscape mosaic would demand herbaceous wetland, though.			Corresponding pixels and associated information exceed biome and net forest target information due to microclimate, plot conditions and habitat et cetera (e.g., also for user-defined cultivated landscape ecosystem types) and represent the initial and potentially later still given landscape mosaic within the general forest landscape.	This LUT envisions the necessary re-establishment of other ecosystems within the general forest landscape.
**RAP-LUT25 restoration of degraded forest**	enrichment planting in degraded forest plots towards growth for potential maximum AGB	≥ 70%	high (in regard to AGB, under use again due to proximity)	Suggested on all user-defined degradation stages, considered as impacted by wood AGB extraction, on simulated disturbed forest pixels	This LUT envisions the recovery of forest site conditions and thereby the achievement of mitigation goals.

### RAP minimum

LPB-RAP calculates the RAP minimum potential of mitigation measures when FLR measures shall be initiated neither landscape-wide nor short-term. To achieve this, LULCC_RAP firstly evaluates the projections from the population data to identify the year of the population peak when considering an internally calculated static footprint approach. On the contrary, it considers the year of summarized peak demands when using the dynamic footprint approach using external time series as presented here. Secondly, LULCC-RAP draws on the mplc landscape for the same year and evaluates all areas under anthropogenic use to derive the population peak or the peak demands land use in case of the external footprint approach. This information is used in a third step to eliminate all overlapping RAP areas per time step *t* until the respective year is reached. As a result, only those areas remain which are not projected to satisfy the maximum anthropogenic demand in the simulated future setting, i.e., pixels within the LUTs of RAP plantation, reforestation, other ecosystems and p.r.n. degraded forest.

### RAP suggested additional restricted areas

For the sake of policy development, LULCC_RAP simulates a spatially explicit and user-defined single output of potential areas p.r.n. suited for a short-term rededication as additional restricted areas. This output may serve as a potential counterpart of conservation compared to restoration under the consideration of population peak demands or summarized peak demands. In this case, the model user can define a list of LUTs, which will be evaluated for the peak year, where the anthropogenically used areas are subtracted before. This approach allows for an approximation of the maximum demand and the available maximum area suited for restoration and conservation until the population or demand stabilizes or declines. The computed information provides an estimate of long-term regional additional potentials for conservation areas (and partly restoration if parameterized), which ideally will be established based on further plot-specific investigations and stakeholder elucidations. Therefore, this output refers to a top-down measure and is only produced in a weak or enforced conservation scenario setting.

## 3. Results

The results section showcases results of the LPB-RAP model as derived for the implementation case study Esmeraldas province of Ecuador (see S2 File for further information on background and parametrization). The basic message of the LPB-RAP simulation approach is the estimate of impact and impact mitigation options of land use patterns in the investigated smallholder-dominated forest landscape in the long term. The model, therefore, provides the user with the scenario information of the landscape without and with potential FLR per simulated time step. Further information on potential long-term annual development are available as visual outputs (e.g., maps and gif) and numeric outputs (e.g., time series) for a broad range of variables. These variables, which include for example pressure aspects, or potential biomass and carbon sequestration, are presented in detail for the case study Esmeraldas in S4 File (selected) and S5 File (full spectrum). This information can be, for example, also used for short- to mid-term measures in regional landscape planning or policy development. In the following sections, we present major modeling results of the simulation that can support decision-making processes; these are (1) simulation uncertainty (section 3.1), (2) comparison of guideline scenarios in combination with the applied land use setting and demand scenarios results (section 3.2.1), and (3) scenario results for the aggregated landscape simulated without FLR (mplc scenario) and (4) with initial simulated FLR measures (RAP scenario), highlighting the aspects of potential future forested areas and land use shares (see section 3.2.2).

### 3.1 Simulated probabilities: implied internal simulation uncertainty

**Fig 5:**
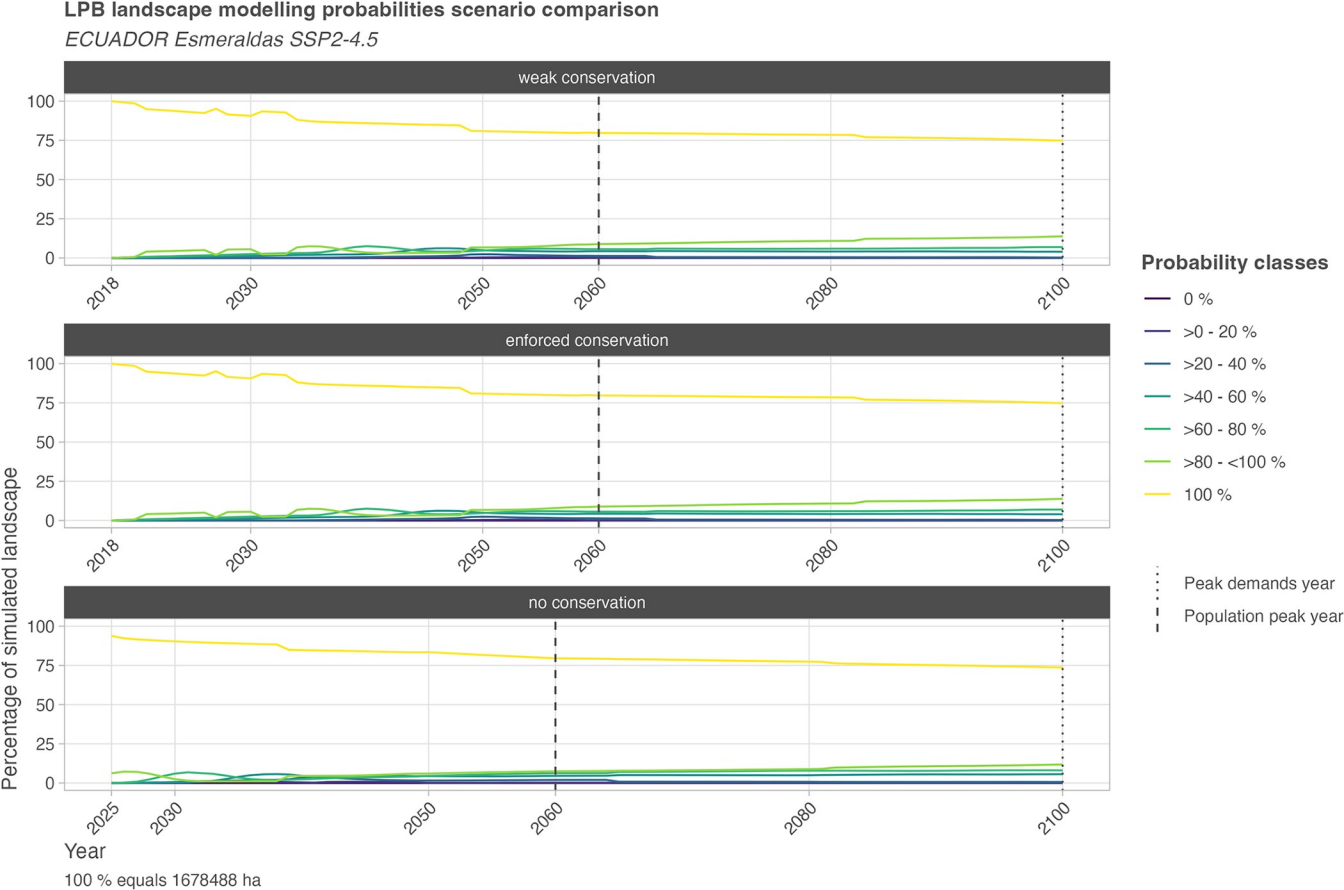
Landscape modeling probabilities classified numerical depiction for the entire simulation time frame. The diagram depicts the classified probabilities in LULCC_mplc, here following the corrective allocation to the factual mplc, i.e., for particular pixels, smaller probabilities than derived from the mathematical mplc can be in place. The class 0% does not occur; it is only implemented to track potential simulation errors. Otherwise, the distribution of probability classes is related to the chosen number of samples; here, the pseudo-random sampling approach was chosen, resulting in 49 samples for the Esmeraldas region. Further uncertainty elements are the stochastically based simulations of plantations and succession besides the development of previously unused areas. For an example visualization of the spatial distribution of uncertainty for a selected probing date, see Fig 6; for all probing dates of the three guideline scenarios marked on the x-axis see section 1.1.2 in S4 File; for discrete numeric values in annual resolution see S5 File.

The resulting information on simulation probability is an essential element of this model approach. It can be used for landscape planning and policy development because it provides information on implied uncertainty of the future LULCC configurations. As shown in Fig 5, depending on the varying trajectories of the Monte Carlo samples, the overall class “100% probability” declines gradually over time. As patterns of land use expansion slightly vary between samples, uncertainty increases partially within the simulation. The stochastic simulation of forest age also leads to increased uncertainty in later simulation stages (change of LUT08 = disturbed forest to LUT09 = undisturbed forest). Overall trends are similar when comparing all three guideline scenarios.

The given probability and uncertainty information refers to the technical simulation within the applied scenario information only, i.e., the probability is based on the number of occasions a pixel is simulated with the same LUT over all samples per time step. Therefore, the model simulates the highest probability for steady land cover or land use, i.e., mainly for LUTs such as water and built-up as well as land use near settlements and undisturbed forest areas not impacted by anthropogenic demands. Uncertainty mainly occurs (1) at the dynamic forest or agricultural frontier where the modeling approach shows variability despite the deterministic setting of terrain parameters and suitability factors, their weights and parameters, or (2) based on stochastic operations (plantations and forests).

**Fig 6:**
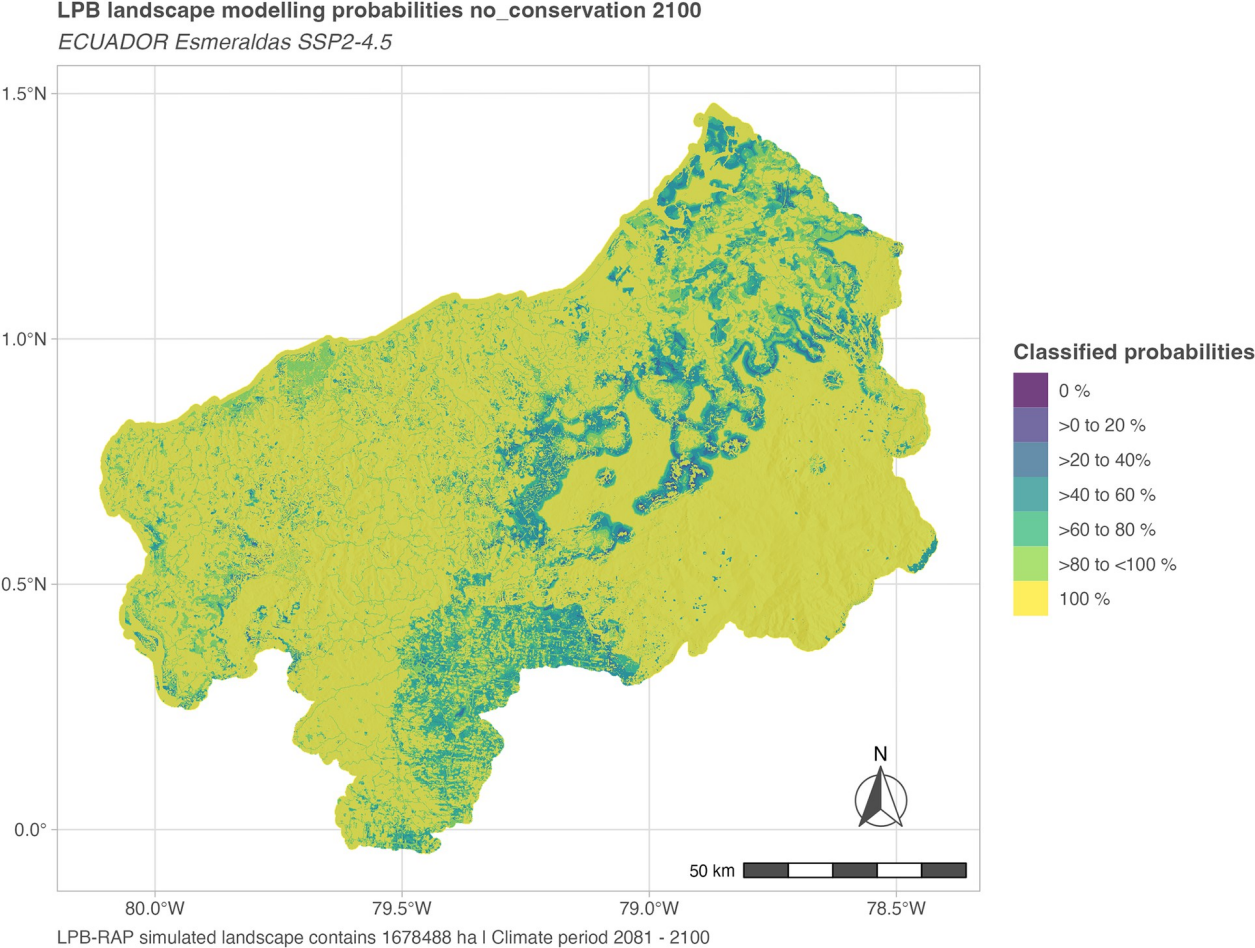
Landscape modeling probabilities spatial depiction for a singular probing date. Spatial distribution of landscape modeling probabilities in 2100 (long-term) in the SSP2-4.5 based policy scenario no conservation. Visible are the differentiated uncertainty classes and their spatial distributions in accordance with simulation rulesets in interplay with dynamic modeling, e.g., for the no conservation scenario land use development in restricted areas (see section 2.3 in S2 File) is depicted and increased uncertainty at the land use development frontiers. For aspects of to be considered model internal and external plausibility please refer to the discussion in section 4.3.

The visualization of the spatial distribution of landscape modeling probabilities supports the model user in the interpretation of simulated landscape configurations. The provided formats are, besides PCRaster map outputs, GIF files (for the entire simulation time frame) and R-based maps (probing dates, see S4 File Background information). Uncertainty increases per simulated time step as expected. Nonetheless, the simulation rulesets, for example, whether restricted areas are used for land use expansion purposes or not, are clearly visible in the emerging patterns. These datasets are provided for each simulated year and defined probing dates for the entire study landscape. They can be subject to further inspections and GIS operations to identify smaller areas of interest for a particular year, for example, for identifying restoration or conservation zones.

### 3.2 Main model findings

The following sections present the results of selected comparative model scenario outcomes for demonstration purposes, i.e., the concluding juxtaposition of simulated scenarios “no FLR” (mplc) and “potential FLR” (RAP) landscape configurations for the baseline scenario SSP2-4.5 setting.

For further selected results depicting all probing dates and for all three simulated policy scenarios the reader is referred to S4 File, which illustrates results for mplc (section 1) and RAP (section 2), such as SSP-related simulated urbanization, derived probable pressure aspects (e.g., indicated pressure on the population by use of difficult terrain and unallocated demands; pressure on restricted areas; pressure on forests by deforestation, conversion or degradation; pressure on habitats by remaining forest quality regionally and in restricted areas), possible mitigation measures (RAP minimum and RAP suggested additional restricted areas). For all covered landscape components, we refer to S5 File.

#### 3.2.1 Restriction policy enforcement levels: scenario comparison

As expected, within the applied scenario assumptions (same demands and largely unchanged parametrization) all three policy guideline scenarios show very similar emerging land use patterns during the course of simulation (see section 1.3 in S4 File and S5 File).

For the Esmeraldas region, scenarios weak conservation and enforced conservation perform de facto identical under consideration of the same parametrization to approximate the starting conditions (see S2 File). This occurs due to the decreasing number of oil palm plantations, a decreasing wood demand and the large share of parameterized unprotected (forest) areas, which in the case of the Esmeraldas province largely denote suitable areas for land use expansions. In case of Esmeraldas province, all projected SSP2-based demands can be allocated throughout the simulation time frame 2018/2025–2100 within the parametrized scenario assumptions. The landscape development is firstly offset by decreasing oil palm plantations area, which allows for the expansion of agricultural LUTs until the population peak (2060) despite an increase in kilocalorie intake per person. Afterwards, the continuously simulated societal shift towards a higher animal-based diet causes the expansion of pastures increasing forest conversion towards 2100, which here coincides with the year of peak demands (due to scenario assumptions in conjunction with data limitations). In the no conservation scenario, land use expands to the formerly restricted areas, increasing the area of land use in (formerly) restricted areas from 12% in 2025 to 23% in 2100. This is in contrast to both conservation scenarios, which did not reveal such patterns.

#### 3.2.2 No FLR vs. potential FLR scenario [mplc RAP juxtaposition]

The juxtaposition of LPB mplc and RAP as the last conceptual scenario stage is tied to an FLR context. The difference can best be visualized for the potential forested area and spatial landscape configurations for a probing date per scenario mplc (*no FLR*) and, within this scenario, the RAP scenario (*potential FLR*) as a counter-proposal.

### Simulated future forested area per time step

**Fig 7:**
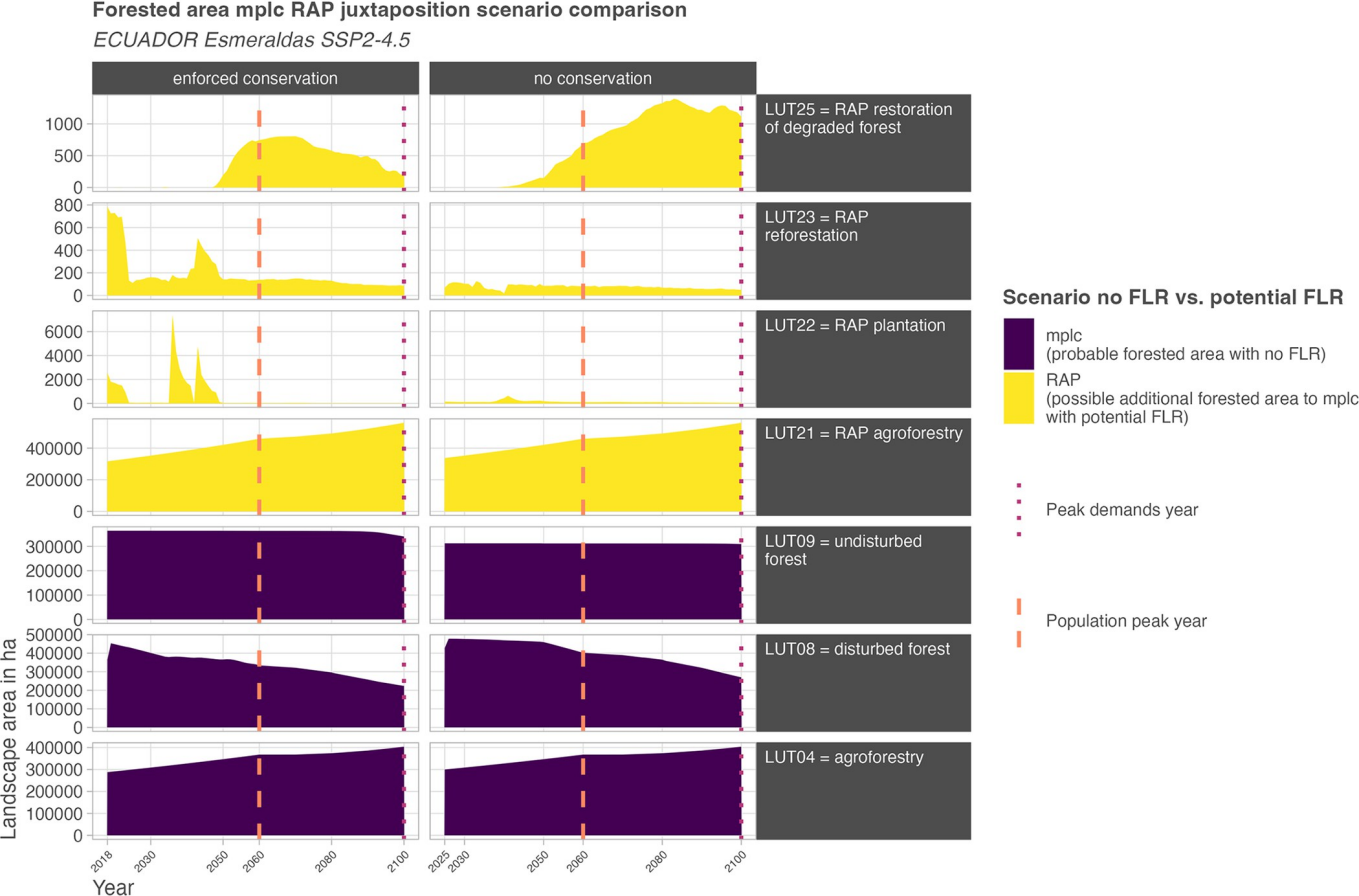
Juxtaposition mplc RAP potential forested area. Potential forested area per time step during simulation based on the mplc landscape configuration and RAP interpretation of forest at terrestrial surface level (LUT08, LUT09 and LUT23), of tree cover (LUT04 and LUT21), as an in time-limited potential (LUT22), or as existing disturbed forest area that can be improved in quality after anthropogenic wood extraction (LUT25). Note that the y-axes differ. Scenario weak conservation is not explicitly depicted here as its simulation, under the case study area conditions in interplay with scenario assumptions, is identical to the enforced conservation scenario.

The main model result is the detection of forested areas that have a likelihood to remain despite anthropogenic use (mplc) and possible suitable area potentials to reinstate forested areas (RAP) with either more forest cover (LUT21 = RAP agroforestry and LUT22 = RAP plantation) or even forest site characteristics (LUT23 = RAP reforestation and LUT25 = restoration of degraded forest) per time step following the implemented scenario assumptions (see Fig 7).

Mplc describes the potential of remaining forested areas in the user-defined scenario setting. It follows here the SSP2-4.5 baseline scenario rationale by simulating population and smallholder demands under changing climate conditions. RAP denotes this aspect per time step by additionally considering possible areas of tree to forest cover and p.r.n. locations resembling forest site characteristics. Agroforestry (LUT04 as well as LUT21) in this context depicts a spectrum. Moreover, the initially simulated peaks in enforced conservation for LUT22 = RAP plantation are mostly based on larger initial areas of herbaceous vegetation and shrubs which are located on potential forest biome pixels outside the targeted net forest distribution. LUT25 (RAP restoration of degraded forest) is also covered in LUT08 (disturbed forest) and in this context implies the potential of forest pixels to fully regenerate under supported management action (for a visualization of the spatial configurations of the selected probing dates see section 2.2 in S4 File or S5 File). Note that the counterintuitive larger share of RAP-LUT25 in the no conservation scenario results from the different spatial use of the landscape due to the assumed repealed or ineffective restricted areas. Due to the thereby possible different spatial allocation of the same demands other forest pixels are affected. This leads to different remaining forest pixels after land use allocation with diverging AGB contents. The higher share of LUT25 thereby documents, that (1) for satisfaction the projected demand, more forest pixels were used, and (2) the used forest pixels are farther apart from the potential maximum AGB recovery.

Conceptually, each time step depicts a potential entry point for FLR measures, which could result in larger areas of potential forested areas. Within the parameterized scenario, LPB-RAP delivers for each time step the theoretical maximum potential under the assumption that landscape-wide FLR has not been implemented yet.

#### Example of simulated dichotomous scenario landscape configurations

**Fig 8:**
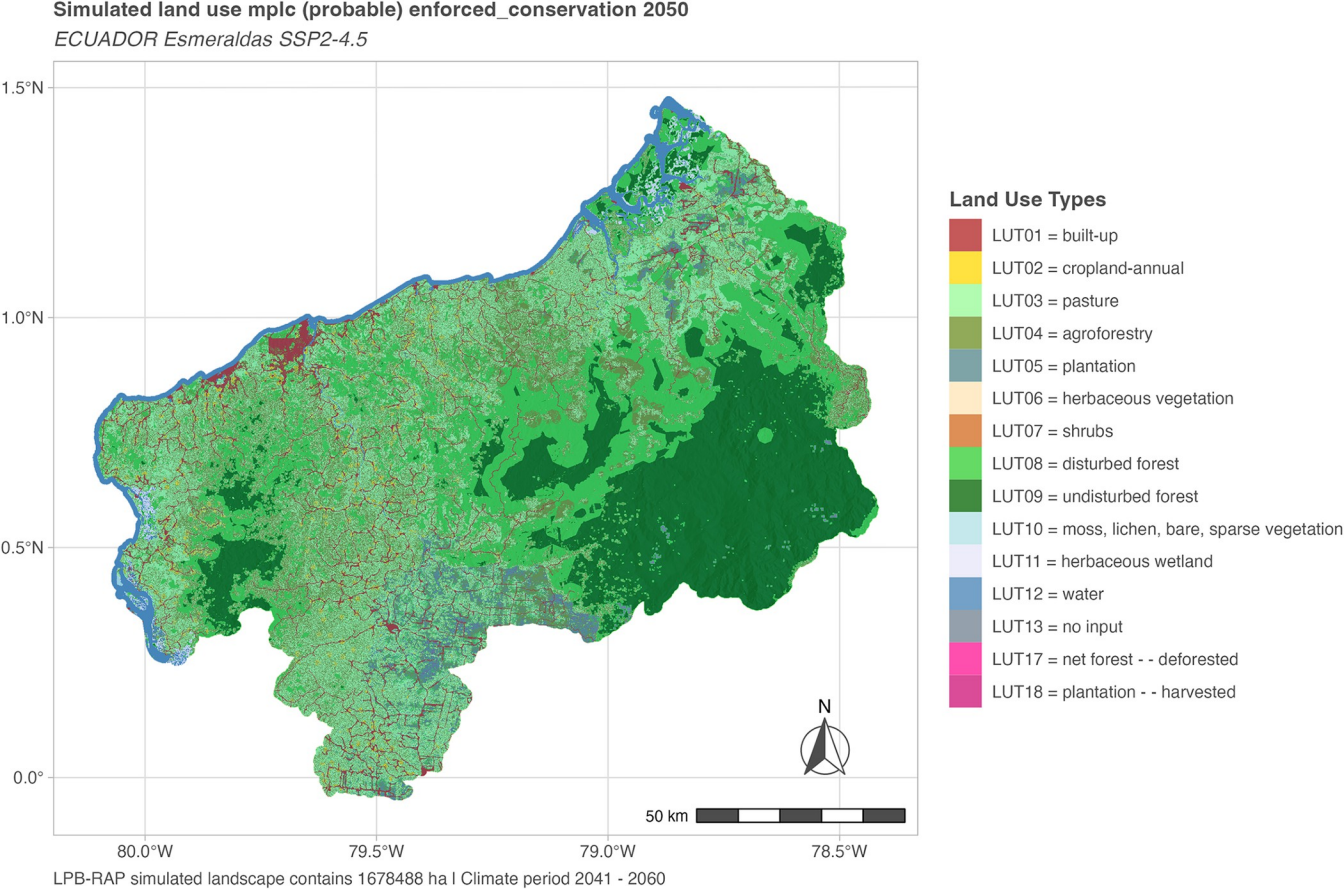
Scenario mplc “no FLR” landscape configuration for a probing date. Mplc “no FLR” spatial distributions of basic LUTs in 2050 (mid-term) for SSP2-4.5 policy scenario enforced conservation. For further probing dates and scenarios see section 1.3 in S4 File. Note that by steadily increasing demands no abandoned LUTs are simulated and only in t = 1 already existing land use in restricted areas occurs.

**Fig 9:**
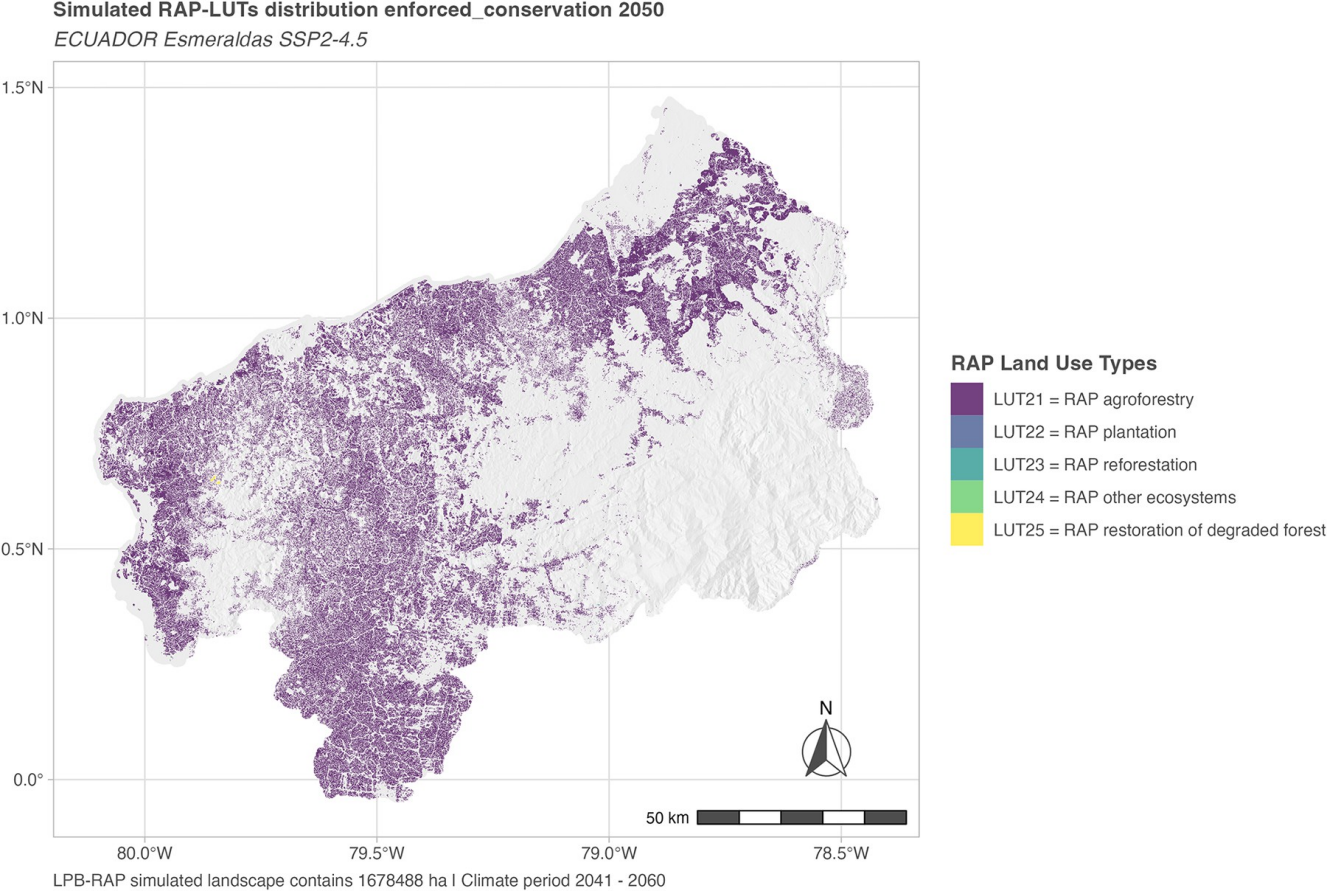
Scenario RAP “potential FLR” landscape configuration for a probing date. Spatial distributions of RAP-LUTs in 2050 (mid-term) for SSP2-4.5 policy scenario enforced conservation (based on mplc landscape configuration depicted in Fig 8). To highlight the potential distribution of RAP the remaining regional landscape area is here only depicted as a hill shade. Note that LUT21 dominates the case study landscape due to the demand in food production. LUTs 22 to 25 also occur but only in very minor quantities and mostly scattered in the landscape. Note also that RAP only describes area potentials for severely impacted pixels that require active measures while conservation potentials are not depicted. For further probing dates and scenarios see section 2.2 in S4 File.

To showcase the new model functionality, we present model outputs for the simulation year 2050 based on LULCC_mplc (see Fig 8) and Restoration Areas Potentials computed in LULCC_RAP (see Fig 9), exemplified for the enforced conservation scenario.

RAP simulation in this scenario case showcases that due to agricultural demands for food production interests in non-food RAP-LUTs can be only marginally fulfilled.

#### Simulated potential additional restricted areas

**Fig 10:**
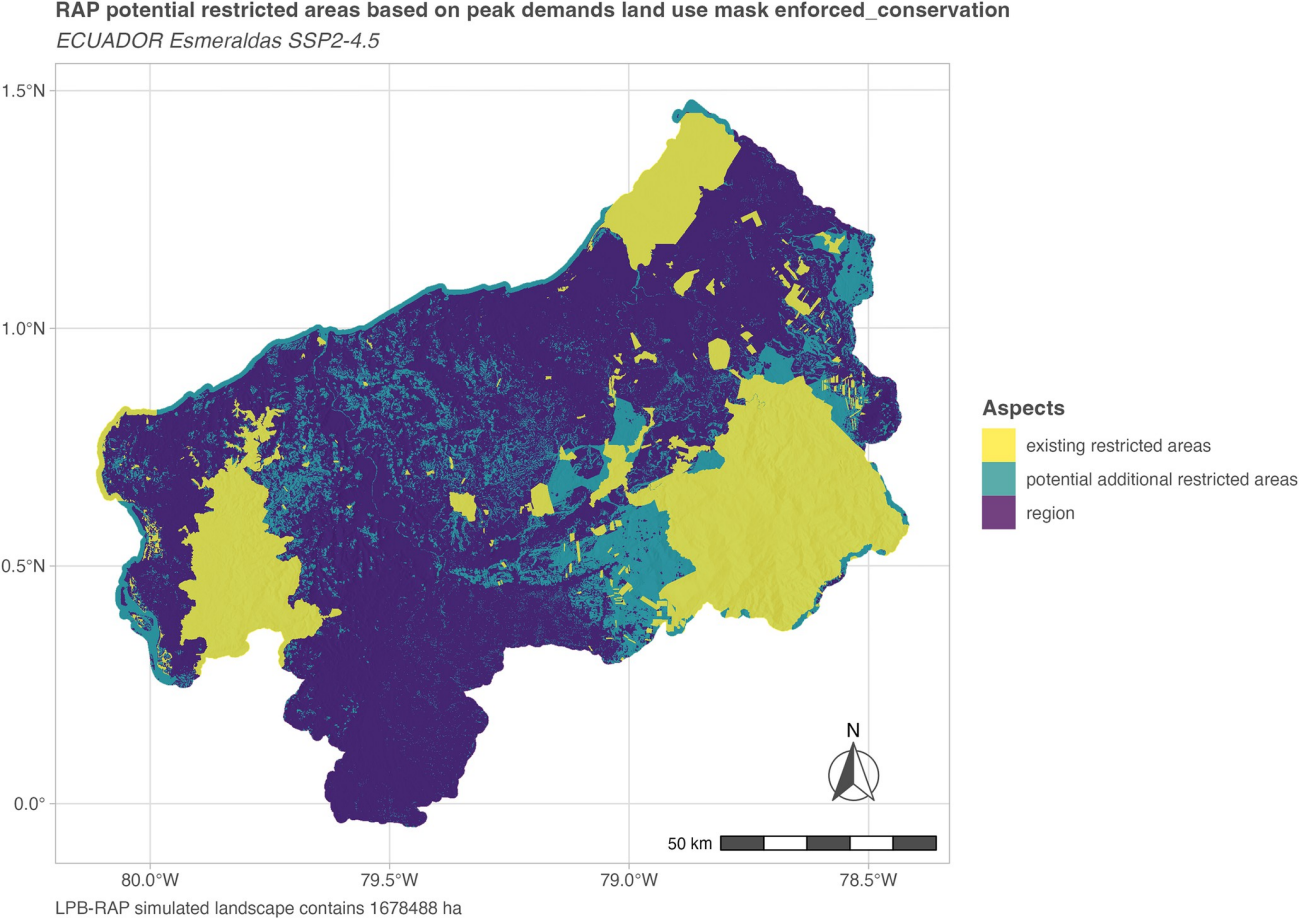
Simulated potential additional restricted areas. Potential additional restricted areas (petrol) compared to existing restricted areas (yellow) within the simulated regional extent (purple). These are area extents that remain available despite land use of the peak demands year (here 2100, enforced conservation scenario).

Within the combined scenario assumptions, there still remain areas that can be potentially protected in the long term as they are not simulated under use for the peak demands year. This coincides here with the final simulation year of 2100 (see Fig 10).

This is one example of how LPB-RAP can be used to support top-down landscape planning and policy development while building on information from long-term bottom-up scenario simulations. In the case of Esmeraldas province for example, existing restricted areas in the simulated extent comprise 414,987 ha or 24.72% of the landscape. Additionally, 261,660 ha could be protected without risking smallholder demands. This would amount to a total area of 676,647 ha or 40.31% of the entire simulated study landscape (Esmeraldas province plus buffer). These scenario-based suggested areas comprise a range of to be individually weighted cases of different levels of protection and management choices and should be further investigated in situ.

## 4. Discussion

This section discusses the novel LPB-RAP modeling approach with a particular emphasis on the RAP modeling components. This is followed by a discussion on the model’s internal and external plausibility as well as current caveats and limitations for the presented model design.

### 4.1 Restoration Areas Potentials (RAP) modeling

To our knowledge, this is the first time a sub-national region such as a province was modeled dynamically and long-term following a SSP2-4.5 narrative in a fine granular 100 m resolution with a particular focus on smallholder land use patterns and to derive RAP estimates. The LPB-RAP modeling approach in its present form approximates RAP of formerly converted, deforested or degraded areas in a forest landscape context. The model does not focus on recent global potential hotspots of restoration [6, 35] but can be understood as a tool for determining the available areas and as a suggested compromise of landscape-based FLR prior to further simulations of other restoration-type scenarios. The latter one could be conducted with additional local information after determining potential areas of interest with the LPB-RAP model, for example, with the use of participatory approaches and cost-benefit evaluation [36–38], or other preparational measures for a more concrete implementation [39–42]. LPB-RAP represents an entirely new approach to RAP modeling with a focus on initiating (F)LR and deriving long-term restoration potentials while ensuring smallholder needs based on the chosen determinants.

For the application of future, especially long-term, scenario-based RAP estimates, we did not encounter a model in the literature to date that (1) functions on the conceptual basis of LPB-RAP (nested scenario simulation, based on combined future climate, population and (smallholder) land use projections); and (2) produces estimates of RAP with underlying simulation uncertainty information as a direct output per projected year.

Additionally, LPB-RAP provides information on pixel-level land use conditions for each consecutive year when aiming to enter into landscape-wide FLR measures. Thereby, LPB-RAP results have the potential to support relevant stakeholders for a long-term planning horizon, especially based on different scenario combinations of SSPs/RCPs, area restrictions and land use.

For the general application of LPB-RAP in the presented form and the depiction of SSP2 assumptions, model results overall indicate the need to break “glocal” (i.e., local to global) patterns near time towards SSP1 measures; this may entail explicit measures for climate protection such as (1) a reduction of the footprint per capita (as an approach of sustainability, e.g., via sustainable intensification measures, changes of consumption patterns or technological advancements); (2) stabilization of population development (e.g., in reaching Agenda 2030 SDGs and to improve human wellbeing in general); (3) increasing the economic value of standing biomass for landholders and smallholders by different means (e.g., by measures such as REDD+); and (4) as model results suggest changing land use types to maintain or increase the share of tree or forest cover as well as forest-associated surface qualities in the impacted landscape despite anthropogenic use (e.g., as suggested via the landscape-based compromise suggestions of RAP agroforestry and RAP sustainable plantations). Overall, modeling results indicate that in an FLR conservation and restoration context the projected moderate worst-case scenario should be avoided in general. RAP land use types depict suitable options to optimize the conditions in this scenario setting under the assumption that the pursued goals are adapted and smallholders and other related stakeholders are supported.

### 4.2 Internal and external model plausibility

We can build for internal model plausibility on two aspects: firstly, the considered base LULCC modeling framework embedding features of the PLUC model thereby relying on the same principle of suitability maps and bottom-up modeling. Secondly, on the assessment of endogenous parameters such as the computed suitability maps, based here on a detailed representation of settlements and corresponding LULCC in the smallholder simulation context. Here we rely on empirical data of settlement points and parameters derived from in situ investigations of land use demands and walking distances. This altogether depicts plausible approximations of the case study context for the initial simulation year as presented here.

Regarding external plausibility, the main aspect of caution is the scenario character of the model as it operates not on the perpetuation of historic trends as common for models such as CLUE but on a set of current empirical data based on a^3^ (approximations, aggregations and assumptions) to describe *future conditions in what-if scenarios*. These scenario assumptions contain in parts the parametrizations, the applied baseline and guideline scenario, the aggregation to mplc and the interpretation in RAP as well as some implemented methods for example in case of settlement growth. LPB-RAP model outcomes should therefore not be interpreted as a projection of historic developments but as the “probable” (no FLR) and “possible” (potential FLR) outcomes for the sum of all scenario assumptions as relevant for the considered modeling duration (here 2018 to 2100). The merit of this approach is thereby the ability to account for possibly changing conditions in the simulated future based on the underlying inputs (e.g., the SSP2-4.5 baseline scenario guiding posts) and the dynamic simulation in general. It must be noted that the model itself is a stand-alone scenario tool and delivers simulation uncertainty for land uses within the applied what-if scenarios; all what-if scenarios can be thus interpreted as equitably plausible futures as demonstrated for the SSP scenarios.

For this study, we chose the SSP2-4.5 scenario narrative “Middle of the Road”. In comparison, the overall SSP1 scenario “Taking the Green Road” would, on the contrary, indicate a more sustainable global to local pathway with a fundamentally more positive impact on climate and population development and land management. However, given the current debates on the impact of climate and global environmental change [43], this optimistic outcome seems less likely. SSP1, associated with a radiative forcing of 2.6. W/m2 by 2100, could be employed for landscapes with no existing FLR to derive a basic, more optimistic restoration and conservation potential. SSP3 (“Regional rivalry-A rocky road”), SSP4 (“Inequality-A road divided”) and SSP5 (“Fossil-fueled development-Taking the highway”), on the other hand, might indicate, besides higher radiative forcing, less supportive circumstances for restoration measures, which is another reason why we decided against them. However, they can potentially be employed to describe such population-climate-land use framework conditions in mplc and derive the remaining potential RAP.

The question if perpetuated trends of historic and recent developments are plausible for a chosen case study is another subject and should be answered externally if desired [44, 45].

The following section illuminates, for such a model external plausibility assessment, backgrounds of the four major modeling components to establish the “most probable landscape configuration” on trends for Ecuador and the simulated case study province of Esmeraldas. It further discusses how well they fit to implemented methods and what-if-scenario assumptions for long-term simulation:

(1) *Climate*: Ecuador has a complex climate due to its location and topography, which is changing rapidly already [46], stressing the importance of climate scenario applications. (2) *Population and urbanization*: Population development in the Esmeraldas province still displays an increase in total population [47], which argues for SSP2(+) assumptions. Obaco and Díaz-Sánchez [48] found that urbanization in terms of Functional Urban Areas has rapidly increased in Ecuador since the 1960s, affirming the assumptions for the applied scenario simulation. In case of the province capital, other simulations also came to the conclusion of a larger area increase [49].

(3) *Policy enforcement level scenarios*: For policy guideline scenarios, we consider the weak and enforced scenarios’ assumptions as realistic, as restricted areas are already demarcated and would be respected under certain circumstances (weak conservation) or strictly protected (enforced conservation). This result can for example support spatially targeted protection measures or conservation zones in the current policy framework of Esmeraldas province.

(4) *Demands*: Demand scenarios have been simulated based on available statistical data and SSP2 assumptions. The scope and trends of regional to international commercial and regional smallholder demands satisfied in this landscape extent is unknown and hence were only approximated via available but to some extent limited primary and secondary data. For agricultural land use types, the approximation is conducted via primary survey data and remote sensing information [26, 31, 50] for the initial simulation year, incorporating conditions based on household surveys and other data sources. Here, land use expansion trends causing deforestation were recorded for Esmeraldas province for the past decades [51] overall supporting our simulation results. Plantations, depicting oil palm in this case, were simulated according to available statistical data. Wood or timber demands on the regional level are not available in full scope and were approximated by remote sensing data and information of recent household surveys. For fuelwood, a declining trend in the employed UN data sources referring to the national trend of Ecuador [52] was visible, indicating that this demand type might decline further in the future despite a continuous population growth. However, subsistence timber demands could not be quantified for the case of Esmeraldas.

For the overall historical trends and recent developments perpetuation, it should be noted that Ecuador is assessed as being on the brink of the transition to an emerging nation [53], which might slowly alter demands in agricultural land, plantations and wood as displayed here. This supports the applied model assumptions and fits the perpetuation of historic trends and recent developments for the case study area.

### 4.3 Current caveats and limitations–external uncertainty

While section 3.1. of the results covered the internal simulation uncertainty implied by the model internal recording of landscape modeling probabilities, this section focuses mainly on external uncertainty for the developed LPB-RAP model. Three aspects should be pointed out in detail for the overall modeling approach and its limitations as presented in this study.

Firstly, limitations of depictions of land use systems must be addressed. Currently, the general model is capable of smallholder land use simulations, which depends on input data for parametrization and as such acknowledges commercial demands to a certain degree only. However, the explicit dynamic simulation of commercial demands with its own parametrization, in addition to the smallholder land use simulation, is not yet part of the LPB model due to lacking primary or secondary regional data, and points to a future task for regional landscape studies as well as the further LPB-RAP model development. For example, in the case of timber demands, according to recent studies, smallholder subsistence demand in the Esmeraldas is rather marginal [23]. However, the province is known for timber production [54]. Hence, in case regional commercial timber data become available, such a demand could also be depicted via an increase of the population-related parameter. But in the case of Esmeraldas, we omitted this solution due to a lack of data at the provincial scale and therefore acknowledge the potential uncertainty that overall deforestation might be underestimated. Likewise, fallow periods (> 1 year) with land system rotations are not yet incorporated in LPB-RAP, limiting the system presentation within the current modeling approach to some extent.

Secondly, the aspect of validating the model’s spatial simulation representation should be clarified [17]. The approximation of disaggregated high-resolution land use at the terrestrial surface level, in combination with the PLUC-based simulation approach of the combined use of an allocation order and suitability factors, their weights and parameters, improves the numerical landscape-based estimates in forest landscapes. However, it inherits spatial uncertainty due to remaining generalization (e.g., the remaining von-Thünen-rings simulation in areas of low population values) and missing validation opportunities. For example, the current distribution and future allocation of built-up are not constrained to the immediate neighborhood of main streets, settlements and cities pixels, as done here in the approximated spatially explicit resolution of one hectare. Nonetheless, the landscape share will likely increase following the simulated trend, further including the representation of urbanization patterns. Notably, the major aspect of external uncertainty is that we cannot validate the internal parametrization step to approximate land use at the terrestrial surface level, e.g., where exactly the singular pixels of simulated cropland-annual, pasture and agroforestry prevail in reality. This limitation arises from the lack of an available map product for confirmation or direct parametrization, which would make the currently embedded parametrization step obsolete. This uncertainty is likely to be experienced by many modelers until more detailed remote sensing map products become available. Thus, (1) the parametrization step to approximate land use is required for an explicit simulation in the current data-limited situation, and (2) explicit modeling will likely improve in the future due to secondary data updates as inputs for parametrization, e.g., the differentiation of agroforestry and plantations from a more generic forest cover land cover class as already suggested for Copernicus land cover maps [26]. This will provide more granularity to represent landscapes in further detail. The major advantage and also aspect of uncertainty of the PLUC approach, and by extension LPB, is the use of only one initial LULC map. This allows in case of PLUC for a starting date for which (national) demand and yield is available in correspondence to a single remote sensing product, which is especially helpful in cases where map coverage is not available for several years, e.g., as required for CLUE modeling. One should further bear in mind that the perpetuation of historic land use trends is not the intended application of models such as PLUC or LPB-RAP, but rather the simulation of future what-if scenarios, which is of growing relevance due to the uncertainty of future pathways, as for example conceptualized by SSP storylines.

Thirdly, the aspect of forest degradation and deforestation must be considered in the forest landscape simulation [18]. The implemented simulation of deforestation for demand in woody input biomass for the potential subsistence demands of timber, fuelwood and charcoal is currently based on a dynamically simulated pixel location and stochastically by increasing biomass content per pixel. This approach was chosen as we could not rely on a diversified dataset of tree species distributions relevant for simulating specified timber demands or their like. This procedure can therefore only serve as an indicator of generalized land use patterns for a landscape under consideration but should not be viewed as full spatially explicit projection. For potential future forest degradation, the current model design acknowledges the depiction of all conceptualized degradation stages based on gradual AGB extraction. However, as this is a fairly complex procedure based on a variety of scenario-based and/or user-defined model inputs and settings, results should be treated with care and only within the explicit scenario context.

However, all of the discussed issues may apply to other land use models too or are not yet acknowledged at all. Likewise, other caveats and limitations representing external uncertainty are mainly based on model parametrization and calibration data of the chosen local to global data products. This may comprise (1) scale effects, such as the baseline scenario information mainly provided on the only harmonized 1000 m scale in contrast to the applied 100 m simulation scale, which emerges in the simulated urbanization as residuals; (2) uncertainty in simulation inputs, for example, restricted areas, which include community-managed land and may therefore diverge from the implemented assumption of prohibited land use; the simulation based on OSM [55] swarm intelligence data, which may include missing or misclassified data points of settlements; the use of a stochastic AGB increment without a climate change signal; and (3) uncertainty of simulation outputs. An example of the latter may refer to the AGB simulation which should not be considered on the pixel level but only as an approximation at the landscape level and per climate period. Nonetheless, given the provision of simulation uncertainty information per pixel, we deem our LULCC model design a functional scenario-based SDSS based on available information within the described limitations, its scenario type character and the target application focus on smallholder-dominated forest landscapes, respectively.

## 5. Conclusions and outlook

The presented LPB-RAP modeling approach presents a valuable new tool for supporting local to regional landscape planning and policy development focusing on smallholder-dominated forest landscapes at the level of a sub-national administrative unit such as a province. In its current design, LPB-RAP raises awareness of the consequences of projected land use development in a changing landscape framework and offers compromise solution suggestions. For future applications, we expect that model performance in regard to the approximation of spatial patterns will further improve with the growing availability of more detailed secondary and current primary data sources, p.r.n. also by the extension of incorporation of regional commercial demands. Further analysis steps are possible to extend the range of potential research questions, e.g., deforestation hotspot analysis or the simulation of different baseline scenarios and subsequent analysis of overlapping areas for restoration.

LPB-RAP model stage 2 aims to extend the simulation of potential future multifunctional landscapes in the Anthropocene with ecosystem fragmentation analysis and the modeling of potential habitat corridors for a user-defined regional umbrella species. Further model development stages may include the incorporation of a) economic aspects in the form of opportunity costs, as well as b) incorporation of potential sustainable forest management.

The model in its present form demonstrated its usefulness of simulating long-term dynamic land use change patterns in combination with potential climate change, population dynamics and developing land use demands. Overall, LPB-RAP proves to be a suitable scenario tool for long-term projections and RAP investigations. It can be characterized as a top-down planning tool based on bottom-up modeling, which requires local follow-up investigations, stakeholder participation across scales and local expertise for the implementation and continued realization of restoration.

Having depicted the two dichotomous different plausible destinations of a continuance of the status quo (mplc) or the entry into landscape-wide FLR (RAP) as a counter proposal on each time step landscape for the simulated region, and likely others like it, we ask all involved stakeholders again: *Quo vadis*, *smallholder forest landscape*?

## Supporting information

S1 File LPB-RAP new generic and user-defined methods. (PDF)

S2 File Case study modeling: parametrization and calibration. (PDF)

S3 File Case study modeling: parametrization of demand time series and calibration to scenario assumptions. (XLSX)

S4 File Case study modeling: Selected results. (PDF)

S5 File GitHub repository containing open-source model, user-manual and required data. Additionally, all secondary result outputs for this study are deposited.
(PDF)
