# Supplementary material for: Quo vadis, smallholder forest landscape? An introduction to the LPB-RAP model
Source: PLoS One. 2024 Feb 2;19(2):e0297439. doi: 10.1371/journal.pone.0297439 (PMC10836681; doi:10.1371/journal.pone.0297439)
Supplement: S1 File — (PDF) [file pone.0297439.s001.pdf]

S1 File – LPB-RAP new generic components and user-defined methods

S1 File introduces shortly an overview of commonalities and differences between the base model PLUC and LPB-RAP and then gives an additional overview of generally applicable model components such as (1) preparation of datasets -if the study design is followed- and new model elements, e.g., available LUTs, and (2) new model internal methods that can be user-defined applied and steered.

S1 File to: "Quo vadis, smallholder forest landscape? An introduction to the LPB-RAP model."

CONTENTS

Prelude **3**
LPB-RAP vs. PLUC 3
LPB-RAP underlying conceptual scenario levels 8
LPB-RAP methods interplay 10
LPB-RAP altered and new allocation procedures 13

1 LPB-RAP new generic methods for data preparation, parametrization, calibration and simulation **15**
1.1 Inter- and extrapolation of climate periods and related potential natural vegetation 15
1.2 Overview and simulation type of available implemented LUTs 16
1.3 Application of correction step for parametrization and calibration of initial simulation year conditions 17
1.4 User-defined or automatically derived samples number in the footprint approach 18
1.5 Parameters for regional case study simulation 18
1.6 Scenario time series of anthropogenic demands projections 19
1.6.1 Built-up LUT (default) 19
1.6.2 Agricultural LUTs (description for footprint approaches) 20
1.6.3 Plantation LUT (depending on approach) 21
1.6.4 Woody above-ground biomass (AGB) 21

2 LPB-RAP further new model specifics compared to PLUC and user-defined methods **22**
2.1 Landscape and topography differentiation 22
2.2 Simulating impacts on forest extent, forest habitat quality and forest AGB quantity 22
2.2.1 Dynamic net forest extent 24
2.2.2 Anthropogenic impact distance 24
2.2.3 Disturbed forest fringe 24
2.2.4 Succession 25
2.2.5 AGB simulation 25
2.2.6 Dynamic forest degradation & regeneration simulation and RAP-LUT25 25
2.3 Systemic choices and gradual simulation options 26
2.3.1 Deforestation prior to or post-conversion to other LUTs 26
2.3.2 AGB increment stochastic or spatially-explicit 27
2.3.3 Street network as input for dynamic built-up simulation 27
2.3.4 Dynamic settlement simulation threshold 27
2.3.5 Slope inclination levels per primary active LUT 28

S1 File references **29**

Prelude

The prelude section provides an overview of essential model development components and their background information.

LPB-RAP vs. PLUC

The following table A1 shortly lists commonalities and differences between the base model PLUC and the newly developed LPB-RAP.

Table A1: LPB-RAP vs PLUC overview

Topic	PLUC	LPB-RAP	Description	Further information section regarding LPB-RAP
**Model type**	Extended land use change model		Both models utilize land use change as the main driver of dynamically simulated landscape configurations but have different focal points of simulation targets	Manuscript section 2.1.2
**Realized as / in**	Open-source model in PCRaster Python		Both models allow for further adaptation and extension (selection criteria)	S5 File
**Modeling approach**	Dynamic in annual resolution		Selection criteria of base model for LPB-RAP	Manuscript section 2.1.2
	Simulation of land use based on suitability maps			S2 File section 3.1.6
	probabilistic with stochastic elements	LULCC_basic: probabilistic with deterministic and stochastic elements	Landscape elements simulation is realized as a deterministic use of climate-period information in LPB, while land use is simulated stochastically as in PLUC	Manuscript section 2.4.1; S1 File prelude
	-	LULCC_mplc: aggregating again to one discrete landscape	New LPB-RAP feature to support applied landscape planning and policy development	Manuscript section 2.4.2; S1 File prelude
	-	LULCC_RAP: re-interpreting the aggregated landscape		Manuscript section 2.4.3; S1 File prelude
**Optimized Application**	National	Sub-national regional smallholder-dominated forest landscapes	New simulation target of LPB-RAP	Manuscript section 2.1.2
	1 km^2 resolution	1 ha resolution	High-resolution modeling in LPB-RAP	Manuscript section 2.1.2
	Aggregating mosaic LUTs (12)	Up to 18 explicit base LUTs and 5 RAP-LUTs		S1 File 1.2; S2 File 2.5
	1 forest LUT, 1 deforested LUT	Up to 5 forest types LUTs		
	National statistics on demand and yield with scenario projections	Primary data of footprint based plus statistics and scenario projections		S2 File 1.1, S2 File 3.1.12
**Time horizon for dynamic modelling**	Short-term (i.e., about 20 years)	Long-term (i.e., about 80 years until data limitation)	New simulation target of LPB-RAP	Manuscript section 2.1.2

**- Climate**	-	Climate scenario-based information of climate reference period and scenario-based consecutive climate periods		Manuscript section 2.2.1; S1 File 1.1; S2 File 2.8
**- Population**	(static dataset, limits to short-term simulation)	Annual scenario-based population development simulation		Manuscript section 2.2.2
**- Land Use**	External scenario-based time-series for demand and yield stochastically simulated	Demand and Yield for agricultural LUTs – simulated stochastically	Expanded code in LPB-RAP to allow for different modelling approaches based on available (primary) data and scenario assumptions	Not featured here
	-	Demand and Yield for agricultural LUTs – simulated deterministically		Not featured here
	-	Footprint internal in the scenario of persistent patterns – simulated statically		Not featured here
	-	Footprint external scenario-based – simulated dynamically		Presented in this study, see Manuscript section 2.1.2, S1 File 1.6; S2 File 3.1.12; S3 File
**Searched for area potentials**	Areas for bioenergy crops cultivation that will not be in concurrence with food production	Restoration Areas Potentials (RAP), primarily in forest landscapes	New simulation target of LPB-RAP	Manuscript section 2.1.2
**Policy enforcement simulation**	Binary (areas will be either excluded from LULCC simulation or not)	Weak conservation	Will use restricted areas only if landscape is otherwise fully developed	Manuscript section 2.3.1
		Enforced conservation	Excludes restricted areas in dynamic probabilistic modelling	Manuscript section 2.3.2
		No conservation	Uses the entire landscape only based on terrain parameters	Manuscript section 2.3.3
**Correction of input map from land cover to land use for forest landscapes**	-	Extended parametrization step, upstream simulation module	LPB-RAP innovation for depiction of conditions at terrestrial surface level in forest landscapes	S1 File 1.3; S2 File 4.; S4 File background information; S5 File
**Monte Carlo framework application**	High user-defined sample numbers for demand/yield approach (about 500 to 1000 for derivation of a meaningful mean)		High TB output and computation time	S1 File 1.4
	-	Automized minimum sample number derived for the footprint approach based on regional conditions of agricultural land use	Significantly reduced TB output and computation time	
**Parameters for sub-national regional simulation (primary data-based)**	-	Case study-specific	Required for sub-national regional modelling, mainly household-questionnaire -based	S1 File 1.5; S2 File 1.; S2 File 3; S2 File 5

**Landscape and Topography differentiation**	Exclusion of user-defined areas and topography-specific areas		Basic LULCC model application	S1 File 2.1;<br>S2 File 2.3;<br>S2 File 2.10;<br>S2 File 3.1.6.3;<br>S2 File 3.1.7;<br>S2 File 3.8;<br>S2 File 4.
**- Exclusion of LUTs**	Static user-defined LUTS	Static and dynamic user-defined LUTs	Improved application in LPB-RAP	
**- Differentiation of slopes**	Only maximum suitable slopes and inaccessible slopes for all LUTs combined	Differentiation of favorable terrain (currently used slopes), difficult terrain (slopes accessible by technology advancement or, e.g., terrace culture) and inaccessible terrain (too steep) per active LUT distinguishable		
**Forest aspects simulation, especially for anthropogenically caused disturbances in regard to forest quality and habitat aspects**	1 LUT forest, 1 LUT deforested	LUT 8 disturbed forest, LUT 9 undisturbed forest, LUT 17 net forest - - deforested , potential forest types LUTs 4 agroforestry, 5 plantation and 18 plantation - - harvested	LPB-RAP innovation for new simulation target	S1 File 2.2;<br>S2 File 2.5;<br>S2 File 3.1.2
**- Dynamic gross and net forest extent**	Dynamic forest LUT only	Differentiation and dynamic modelling of gross forest (all user-defined forest type pixels) and net forest (based on initial extent of national forest definition)		S1 File 2.2.1
**- Simulation of anthropogenic impacts on forest quality**	-	User-defined anthropogenic impact distances for 1) general impact in regard to potential habitat function and 2) especially wood extraction around settlements		S1 File 2.2.2;<br>S2 File XX
**-Disturbed forest fringe**	-	Automated simulation of disturbed forest fringe		S1 File 2.2.3;<br>S2 File
**-Succession**	Regrowth of forest after 9 years deforested	Detailed user-defined succession depiction based on potential natural vegetation modeling per climate period		Manuscript section 2.1.2;<br>S1 File 2.2.4;<br>S2 File 3.1.10
**-Simulation of woody aboveground biomass (AGB)**	Demand and Yield in m^3, increment simulated stochastically	Demand and Yield in Mg, increment simulated spatially-explicit (climate-period based) or stochastically	Here stochastically, since spatially-explicit was not applicable to the regional extent	Manuscript section 2.1.2;<br>S1 File 2.2.5;<br>S2 File 3.1.4
	Deforestation only simulation	User-defined simulation of deforestation at net forest fringe only or additionally smallholder steered AGB extraction around settlements (degradation simulation basis)	LPB-RAP innovation for new simulation target	Manuscript section 2.1.2;<br>S1 File 2.2.6;<br>S2 File 3.1.3
	-	Dynamic, user-defined simulation of forest		Manuscript section 2.1.2;<br>S1 File 2.2.6;

		degradation and regeneration based on AGB content in % in relation to potential maximum undisturbed cell value of AGB		S2 File 3.2.3
**Systemic choices and gradual simulation choices**	Active LUTs allocation order		Base model selection criteria to depict bottom-up land use of smallholders compared to top-down approaches	S1 File 2.3;<br>S2 File 5.
**- Deforestation prior to or post-conversion to other LUTs**	Partly depictable by allocation order	User-defined parameter that will simulate deforestation and p.r.n. smallholder based local wood extraction prior to or post the en bloc simulated land conversion	Systemic depiction of case study	S1 File 2.3.1;<br>S2 File 5.1
**- AGB increment stochastic or spatially-explicit**	Only stochastic increment applicable	Climate-period-based spatially-explicit or stochastic increment	Allows for simulation of climate change impact if spatially-explicit	S1 File 2.3.2;<br>S2 File 3.1.4
**- Street network as input for dynamic built-up simulation**	Built-up is static in PLUC	Dynamic simulation of built-up with user-defined input to steer magnitude of change according to regional conditions or scenario assumptions	LPB-RAP innovation for new simulation target	S1 File 2.3.3;<br>S2 File 5.2
**- Dynamic Settlement simulation threshold**	No settlement simulation in PLUC, only static depiction of Cities	Static Cities depiction for sub-national regional extent, new and dynamic settlement simulation, user-steered by parameter adjustment for different scenario assumptions		S1 File 2.3.4;<br>S2 File 5.3
**- Slope inclinations levels per primary active LUT**	-	User-defined parameters to adjust regionally and to scenario assumptions		S1 File 2.3.5;<br>S2 File 3.1.7
**Parameters overview**	Base parameters	Extension and adaptation of parameters	LPB-RAP extension of PLUC base parameters to serve new simulation target	S2 File
**Analysis output**	Probability map for bioenergy crop cultivation area potential	Not featured any longer	Change of simulation target focal point	Manuscript section 2.1.2
**Focal landscape variables analyzed**	Only pixel probability for bioenergy crop cultivation area potential	Landscape modelling probabilities for aggregated landscape	LPB-RAP extension to ease output interpretation	Manuscript section 3.1.;<br>S4 File 1.1;<br>S5 File

		About 370 variables analyzing per time step the impact of landscape and land use change on the landscape configuration as whole and with the primary foci on pressure aspects of impacts on the smallholder population as well as forest quality and quantity before and after potential FLR implementation		Manuscript section 3 Results, S4 File (selected Results), S5 File (complete secondary results)
**CSVs**	-	One main CSV per LPB and RAP featuring about 370 variables plus thematic and tidy_data output for machine interpretation		S5 File
**GIFs**	1 sample of the probabilistic modelling approach is depicted	Up to 26 thematic GIFs of landscape development in LPB (LULCC_basic samples and LULCC_mplc aggregated results;<br>5 RAP GIFs;<br>Most of them realized in the accessible VIRIDIS color spectrum		S5 File

LPB-RAP underlying conceptual scenario levels

LPB-RAP relies on a nested what-if scenario approach in its conceptual scenario levels to simulate LULCC driven by population dynamics, projected climate periods and smallholder-driven land use area demands until 2100 in diverse policy scenarios and to derive the target outcomes in LULCC_mplc and LULCC_RAP, see Fig A1.

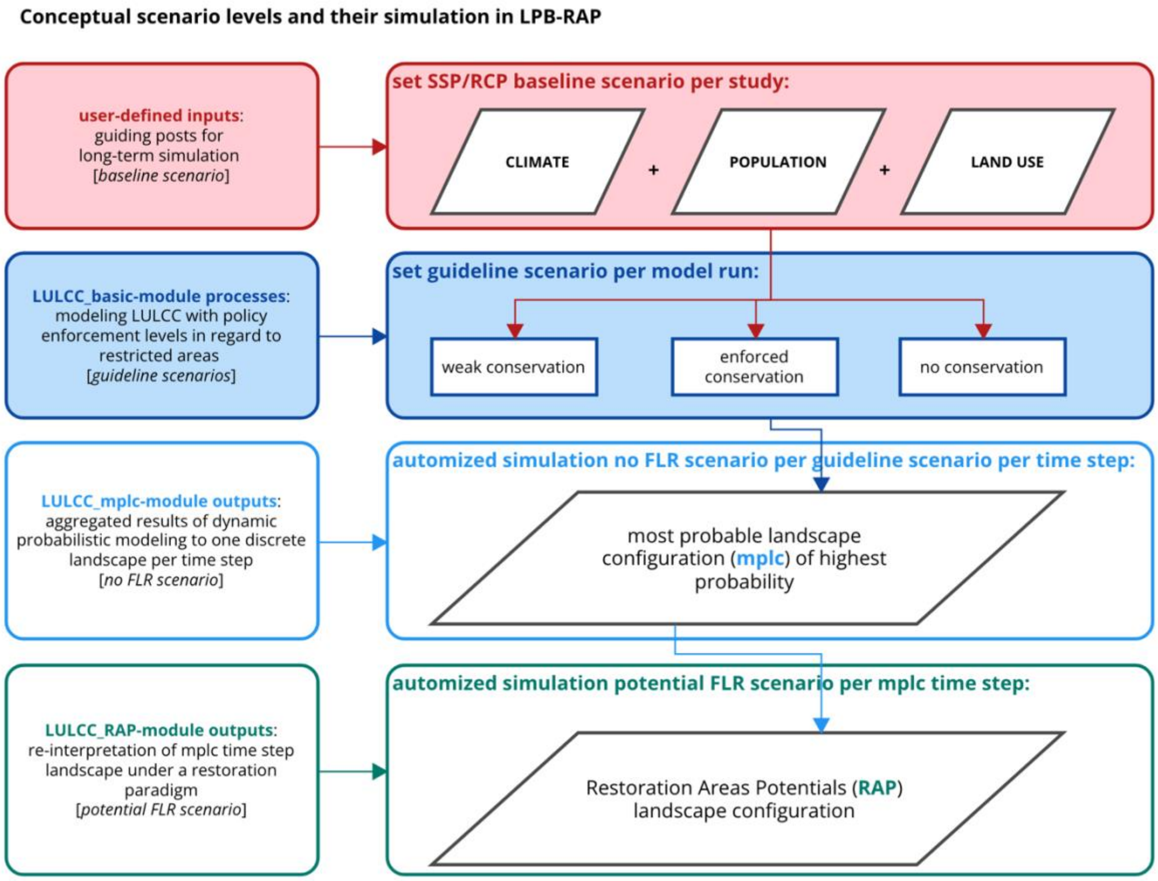

Fig A1: *Overview of nested scenario rationale in LPB-RAP realized by a consecutive execution order of user-defined inputs and implemented modules (LULCC_basic, LULCC_mplc, LULCC_RAP) leading to the primary results of the juxtaposition of "no FLR" (mplc) and "potential FLR" (RAP) landscape configurations. Scenario descriptions are shown in brackets.*

Nested, in this case, refers to a conceptual and modular structure of LBP-RAP which eventually allows the user to simulate scenarios as part of module LULCC_basic, LULCC_mplc or LULCC_RAP. This design is implemented in the model to allow the user to simulate different scenarios in the approximation of a complex coupled human-environmental system. The underlying rationale for the primary simulation target outcomes is that a "no FLR" result, i.e., a landscape based on development without implemented FLR, must first be produced before a "potential FLR" result's dichotomous but entwined juxtaposition can be derived, i.e., the same time step landscape as an entry point to landscape-wide FLR.

All SSP and RCP scenarios of climate, population and land use development can be used and adapted to a particular scenario narrative for a subnational regional forest landscape. However, in case of built-up the model uses a hardcoded approach to interpret demand as a proportional growth based on population development. In case of agricultural land use types LPB-RAP can employ either the PLUC demand/yield approach or a newly implemented land footprint per smallholder capita approach based on cross-sectional household survey data instead of trend information from time series. Deforestation indirectly results from the combined subsistence demands in timber, fuelwood and charcoal in Mg woody above-ground biomass. This can be user-defined either simulated only per population total as complete deforestation of plots at

the net forest fringe or in contrast with a share of simulation of smallholder AGB extraction around settlements, which can result in several user-defined degradation stages and the according RAP-LUT.

In its present form, LPB-RAP allows the user to choose several options to simulate scenarios:

The *first level* consists of user-defined baseline scenario determinants of projected future climate, population and land use development.

The *second level* allows user to define policy enforcement scenarios (guideline scenarios) that define whether restricted conservation areas in a landscape can be used *(weak conservation scenario)* or not used *(enforced conservation scenario)*, or whether no conservation areas prevails any longer (*no conservation* scenario). These scenario-based adjustments steer the dynamic simulation accordingly throughout a simulation. For a 21st-century scenario narrative, we anticipate that general framework conditions may change over time and thus, demands must be satisfied regionally if plausible due to a future lack of expansion area. Unallocated demand as part of scenario-specific population development could be a driver of land use change spill-over effects to neighboring or other regions via teleconnections (Meyfroidt et al., 2018), or be interpreted as threatening smallholders' food security. In the context of the aforementioned scenario assumptions, policy scenarios are not conceptualized as a perpetuation of historic trends or recent developments, but as assumptions on conditions that may apply for the simulation timeframe 2018 until 2100. These scenarios are realized via user-defined parameter inputs of spatially defined restricted areas and defined terrain inclination levels (favorable, difficult and inaccessible terrain). The location of restricted areas and terrain inclination levels must be parameterized depending on the context of the particular case study region or for scenario assumptions (see S2 File section 2.3 for details on Esmeraldas Province).

Note that in this modeling approach, the combined guideline plus land use scenario outcomes are not the primary target of simulation and analysis. Restricted areas or formerly restricted areas and their recognition by the population here simply function as a landscape simulation element determining where land use in which form might be located in the probabilistic base module, before deriving the following target results at the regional landscape level:

The *third* and final *fourth level* of scenario applications build on the previous stages, here the aggregated most probable landscape configurations (LULCC_mplc) as the continued "no FLR" scenario and corresponding the possible landscape configurations under a restoration paradigm (LULCC_RAP) as the "potential FLR" scenario are simulated by LPB-RAP as the primary target model results. We differentiate between these two scenario levels for the following reason: FLR is a relatively new concept and still not widely implemented, therefore the model in model stage 1 assumes this development as a use case and thereby the most probable landscape configuration as a scenario of "no FLR" (progressive scenarios like implemented Sustainable Forest Management (SFM) or Payment for Ecosystem Services (PES) could be incorporated in further model development stages, see Chapter 5). In contrast, the juxtaposition of a "potential FLR" landscape configuration indicates respective pixels under a restoration paradigm that may or may not be realized by stakeholders and is therefore independent of the simulated potential land use probability other than the spatial allocation. This is because LPB-RAP can only rely on a limited number of variables to describe RAP and hence cannot fully account for all factors relevant to fulfilling restoration options, such as biophysical conditions, e.g., soil fertility status, and stakeholders' motivations at the plot level, e.g., based on tenure aspects or opportunity costs. Accordingly, LPB-RAP is to be characterized as a heuristic SDSS because it delivers a planning basis at the larger regional landscape level, based on user-provided information, for the theoretical maximum potential of restoration, but for the implementation of restoration on the ground further local investigations and stakeholder participation will be necessary.

LPB-RAP methods interplay

Fig A2 (next page) describes the major methods interplay referenced in this document. The consecutive Fig A3 describes dynamic methods interplay in the module LULCC_basic for dynamic simulation of the scenario time frame to build a scenario landscape configuration based on the rulesets applying to a pixel of one of the basic LUTs.

Regarding Fig A2 Major methods interplay:
The methods apply in the conceptual and technical structure from bottom to top as steering layers of simulation information column-wise but also in consecutive order from left to right on the time axis. Basically, time step 1 is simulated twice (by user-choice in the correction step and as part of the dynamic simulation) to approximate the starting conditions by parametrization and thereby calibrate the model. Time steps t_2 to t_n then apply the long-term scenario assumptions. Baseline scenario information is drawn for each time step (whereby information resolution depends on the data and applicable algorithms, by default climate data-based information is provided with 20 years resolution, population annually interpolated and land use user-provided but annually drawn). User-defined maps and parameters of the chosen guideline scenario as well as further set parameters then steer the simulation of the active landscape configuration simulation for a landscape trajectory per sample. Herein, we refer to land use elements and landscape elements simulated. The land use element modeling (simulation of active LUTs) is simulated first, since it affects the characteristics of the landscape elements of vegetation modeling in distribution, simulated age and impact, and accordingly succession and degradation. Lastly, the new consecutive modules derive for each time step simulated the target scenarios of the aggregated no FLR landscape and the concluding potential FLR landscape subsequently.

Regarding Fig A3 Minor methods interplay:
On the pixel level the rulesets apply basically in chronological order based on the specific basic LUT of LPB. Additionally, user-choices determine which areas and LUTs can be impacted and when and how. Since a flowchart diagram of a decision tree is hard to realize for the complex methods interplay, we here decided to show the basic framework of applicable rules for a pixel per LUT or LUT category. The diagram is best interpreted reading it top-down and left to right.

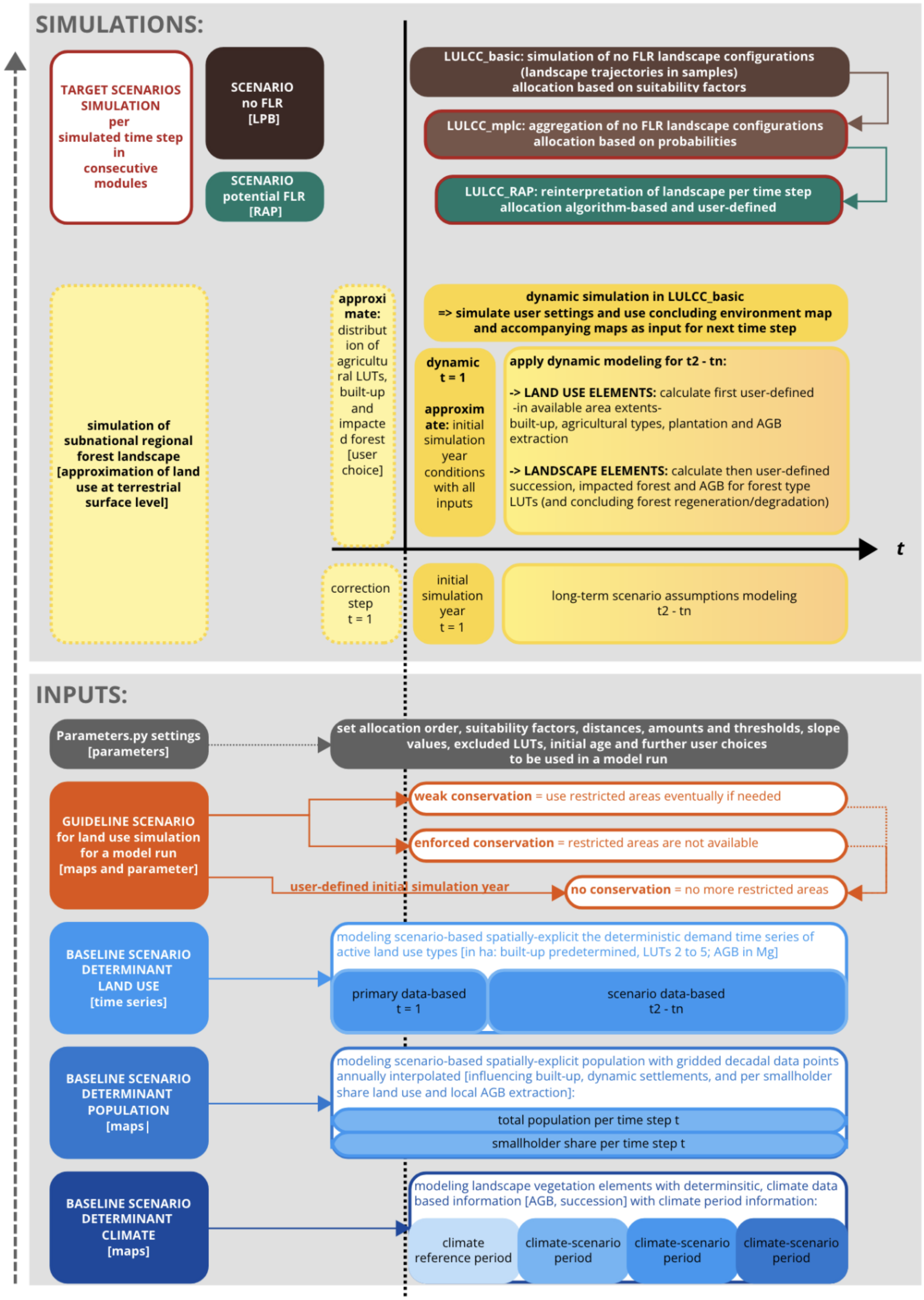

Fig A2: Major methods interplay

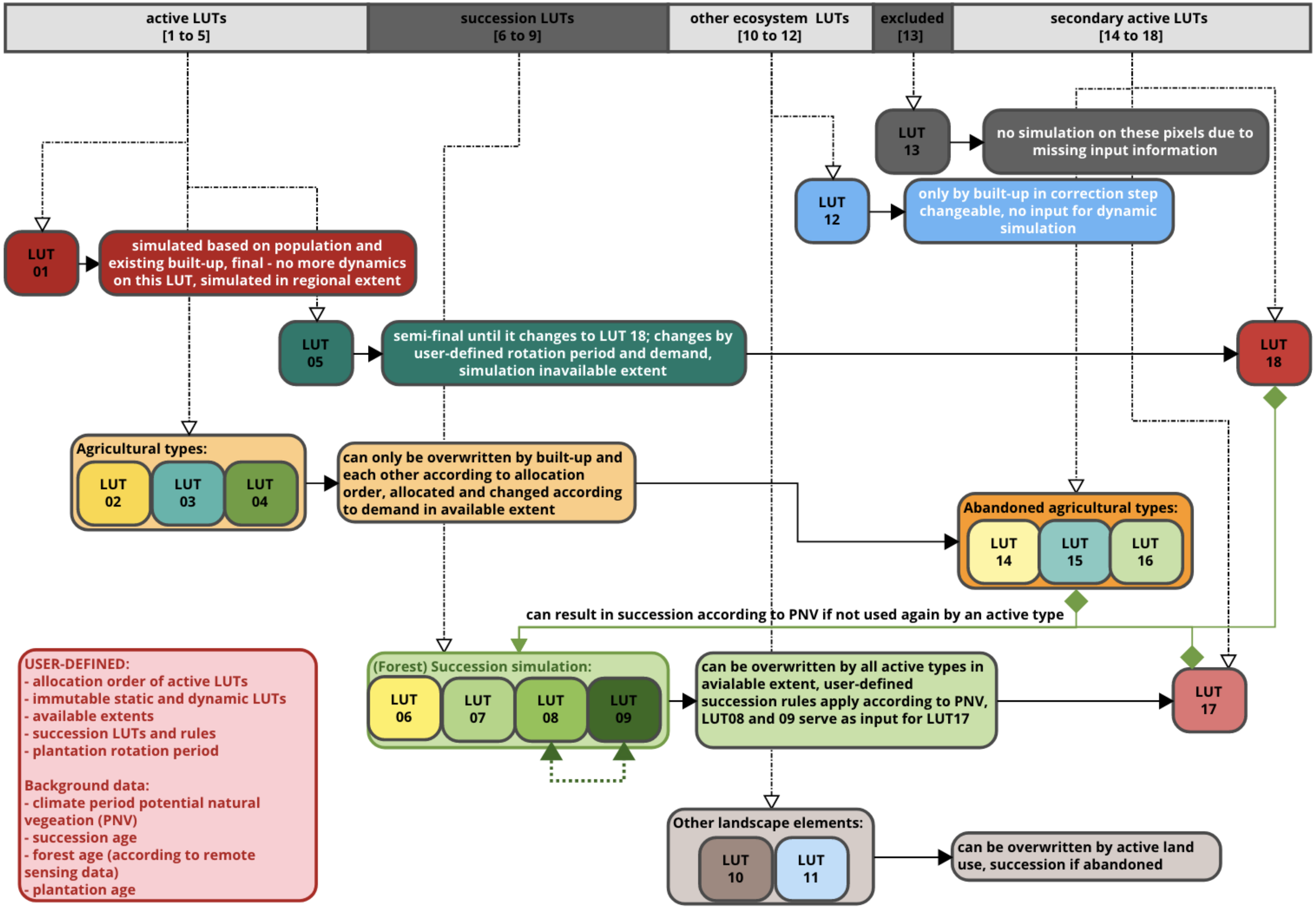

Fig A3: Minor methods interplay on pixel level

LPB-RAP altered and new allocation procedures

1. LULCC_basic module (former PLUC model file)

The original PLUC model was redesigned and extended to serve the needs of LPB, forming the new module LULCC_basic. The redesigned model structure incorporates new aspects of deterministic modeling (climate-based determinants, population development, land use demands and dynamic settlements), stochastic modeling (land use allocation, plantation age and succession age, forest type-specific above-ground biomass (AGB) increments if chosen by the user) and probabilistic modeling relying on the PCRaster Python Monte Carlo framework (singular LUTs and a range of accompanying maps). The latter refers to the general modeling framework of LULCC_basic where now, for each LUT and time step *t,* a Monte Carlo average is drawn and area-specific outputs are generated, e.g., net forest and related deforested areas as well as conflict zones of land use and forest in restricted areas. Further additions to this module are (1) a user-defined, more nuanced representation of slope inclination levels for each primary active LUT by categorizing areas into favorable terrain, difficult terrain and inaccessible terrain; (2) impacts on forest quality and forest extent; (3) forest successional stages and (4) annual AGB increments per considered forest type.

2. LULCC_mplc (new aggregation module)

LULCC_mplc simulates the land use demand solely based on probabilities that inherit the suitability criteria information applied in the LPB base module LULCC_basic and derived aggregation values for the related LUTs. In addition to the deterministic demand of agricultural LUTs, the prior derived aggregation values of mean and maximum simulated land use for a primary active LUT or abandoned/deforested/harvested secondary active LUT over all samples are essential simulation information in this module. LPB uses the maximum value of allocated pixels for the primary active types to avoid an underestimation of area required for anthropogenic activities, i.e., impact on the target variables of succession LUTs (especially LUT08 = disturbed forest and LUT09 = undisturbed forest) and other ecosystems LUTs. For the abandoned, deforested or harvested secondary active LUT, the mean value is used to avoid and overestimation of the landscape shares during a simulation step. These LUTs could be overwritten in the LULCC_basic allocation and serve mainly as input for RAP. Passive and static types are equally passed along without aggregation other than in the mathematical mplc. To correct the area overestimations of MC averages per LUT, the model requires information on the alternative pixel type, which is solved by using the climate period-dependent biome maps of potential natural vegetation, depicting forest succession types within the general forest landscape. This procedure is executed to either avoid over- or underestimations concerning the simulated area demands in the following LULCC_RAP module and for estimating RAP according to available areas for the considered study context. LULCC_mplc adapts the LULCC_basic allocation procedure, i.e., using the user-defined allocation order and transferring immutable cells that have already been changed. Where the allocation diverges from the mathematical mplc by using the initial MC averages, the probability value of the pixel is recorded during the simulation since it can have a lower probability than the mathematical mplc result. In the case of LULCC_mplc, this option is solely based on the Monte Carlo averages for each time step for the primary and secondary active land use types. Subsequently, they also depict -constrained- a sequence of development and are simulated accordingly for each time step. Therefore, the simulated time frame depicts a sequence of development for the overall landscape but displays minor deviations for singular pixels based on the necessary aggregation and corrective allocation per time step t. Therefore, each time step can be viewed individually as a simulation output on its own or as an intermediary output that serves as input for the module LULCC_RAP.

3. LULCC_RAP (new interpretation module)

While the allocation of RAP-LUTs 21 to 24 is relatively simple, RAP-LUT25 is relatively complex in simulation. The following components influence the distribution of this LUT in its entirety:

Potential maximum undisturbed AGB for climate periods	Since degradation is measured in % AGB content in relation to the potential maximum undisturbed AGB value of a cell, these datasets steer the development over time for each provided climate period, one might recognize these effects in the output data, especially for time step 1 (since the last time step used for this simulation year is the initial AGB map) and the years depicting a climate period change.
AGB increments for disturbed and undisturbed forest (p.r.n spatially explicit for climate periods)	Depending on the stochastic or spatially explicit AGB annual increment the time step end result of AGB content may change and thereby if the cell is still counted as degraded or in regeneration.
Total wood demand population and smallholder _share for local wood demand extraction simulation	Degradation (remaining AGB > 0 Mg AGB after AGB extraction) in this model can only be simulated via smallholder wood extraction to satisfy demands, which in turn is simulated as the share of the total demands of the population. Thereby, if demands are very low or decreasing, simulated degradation and in turn restoration will decline or not be simulated at all (if increments exceed the extraction fraction), depending on the input and simulation data.
Impact distance of local wood consumption	This parameter steers the simulation very much since the algorithm it is used in distributes the smallholder wood demand in fractions in a loop over the available cells around settlements in this calculated forest buffer zone. Thereby, if the same demand is allocated in a smaller radius, there will be less cells but with potentially a higher degradation stage. To approximate reality this distance should include transportation with motorized vehicles. Still, only a rough approximation can be reached, since simply all settlement surrounding forest cells are used and no further spatially explicit information of wood extraction locations is available.
Degradation AGB thresholds	This parameter also offers the choice to adapt regionally or simulate different scenarios, depending on the underlying definition respectively the set two values to derive the three user-defined classes in addition to the preset fourth stage of degradation absolute (0 Mg AGB after complete deforestation).
Selected degradation stages to simulate restoration	Here again the user has the opportunity to simulate different scenario goals or policies. One can choose one, two or all three stages of degradation as input for the RAP-LUT simulation. Simulated results will differ accordingly.

1 LPB-RAP new generic methods for data preparation, parametrization, calibration and simulation

1.1 Inter- and extrapolation of climate periods and related potential natural vegetation

This section provides information on applicable procedures and data for model applications based on the CHELSA data. These datasets were initially calculated on the global extent.

Climate periods:

For dynamic modeling of the time frame 2018 to 2100, the provided climate period means (2050 for 2041–2060 and 2070 for 2061–2080) could be used directly. For estimating the climate of the remaining two targeted time periods 2021–2040 and 2081–2100, the linear trend in temperature and precipitation values has been derived cell-wise from the closest available climate periods for 2030 and 2090, respectively, and has subsequently been used to supplement these values for the missing time periods. While deriving the climate variables for the period 2021–2040 (2030) is a pure interpolation from current and 2041–2060 data and does not need further processing, the extrapolation of precipitation trends for 2081–2100 (2090) from 2041–2060 and 2061–2080 data additionally had to prevent the possible occurrence of negative values. All processing has been done for the complete global dataset, followed by the derivation of bioclimatic variables. The SAGA Python scripts provided in S5 File can be used to execute the required operations.

Table A2: Simulation periods gives a short overview of the applied climate period data

Simulated period	applied climate period (mean)	period description
**2018–2020**	**2010**	**climate reference period CHELSA (1979–2013)**
**2021–2040**	**2030**	**climate period CHELSA MPI-ESM-mr interpolated**
**2041–2060**	**2050**	**climate period CHELSA MPI-ESM mr**
**2061–2080**	**2070**	**climate period CHELSA MPI-ESM mr**
**2081–2100**	**2090**	**climate period CHELSA MPI-ESM mr extrapolated**

Potential natural vegetation:

Note that the produced global dataset based on the CHELSA data cannot be applied to all locations for studies since we did not implement a function to describe future glacier extents because these are not featured in the LaForeT regions. Hence, for the scope of this analysis, the distribution and extent of the glaciers have been considered as not changing and masked out from the maps.

The datasets are based on the full scope of the climate periods data. The original classes for the global file for each climate period of potential natural vegetation (see list below) are aggregated in an approximation of the applicable model LUTs (see LPB application below):

1. cold deciduous forest
2. cold evergreen needleleaf forest
3. cool evergreen needleleaf forest
4. cool mixed forest
5. cool temperate-rainforest
6. desert
7. erect dwarf shrub tundra

8. graminoid and forb tundra
9. low and high shrub tundra
10. prostrate dwarf shrub tundra
11. steppe
12. temperate deciduous broadleaf forest
13. temperate evergreen needleleaf open woodland
14. temperate sclerophyll woodland and shrubland
15. tropical deciduous broadleaf forest and woodland
16. tropical evergreen broadleaf forest
17. tropical savanna
18. tropical semi-evergreen broadleaf and mixed forest
19. warm-temperate evergreen broadleaf and mixed forest
20. xerophytic woods/scrub

LPB application:

- not applicable types for the succession of forest landscape modeling based on Copernicus land cover types in the region (6, 20) are assigned class 0
- overall grassland biome types pixels are assigned class 1 (11, 8) - the model simulates succession on these pixels only until the land cover type "herbaceous vegetation"
- overall shrubs and bushland biome types pixels are assigned class 2 (this also serves as an aggregation for the type tropical savanna; 7, 9, 10, 17) - the model simulates succession on these pixels only until the land cover type "shrubs"
- overall forested and wooded biome types pixels are assigned class 3 (1, 2, 3, 4, 5, 12, 13, 14, 15, 16, 18, 19) - the model simulates succession on these pixels until land cover type "disturbed forest" or "undisturbed forest" – here after a period of 100 years without anthropogenic impact

1.2 Overview and simulation type of available implemented LUTs

Table A3: LPB-RAP Land Use Types (LUTs)

land use type ID	land use type name [depicting land use at the terrestrial surface level]	simulation type
**LUTs that are recognized in modules LULCC_basic and LULLC_mplc (LPB)**		
**LUT01**	built-up	primary active LUT, final LUT
**LUT02**	cropland-annual	primary active LUT
**LUT03**	pasture	primary active LUT
**LUT04**	agroforestry	primary active LUT
**LUT05**	plantation	primary active LUT, semi-final LUT
**LUT06**	herbaceous vegetation	only active in succession or corrective allocation
**LUT07**	shrubs	only active in succession or corrective allocation

**LUT08**	disturbed forest	only active in succession or corrective allocation
**LUT09**	undisturbed forest	only active in succession
**LUT10**	moss, lichen, bare, sparse vegetation	passive, other ecosystem LUT
**LUT11**	herbaceous wetland	passive, other ecosystem LUT
**LUT12**	water	passive, other ecosystem LUT if user-defined
**LUT13**	no input	excluded from simulation
**LUT14**	cropland-annual -- abandoned	secondary active LUT
**LUT15**	pasture -- abandoned	secondary active LUT
**LUT16**	agroforestry -- abandoned	secondary active LUT
**LUT17**	net forest -- deforested	(primary or secondary) active LUT
**LUT18**	plantation -- harvested	secondary active LUT
**LUTs that occur only in the LULCC_RAP module**		
**LUT21**	RAP agroforestry	RAP LUT (interpretation of mplc landscape)
**LUT22**	RAP plantation	RAP LUT (interpretation of mplc landscape)
**LUT23**	RAP reforestation	RAP LUT (interpretation of mplc landscape)
**LUT24**	RAP other ecosystems	RAP LUT (interpretation of mplc landscape)
**LUT25**	RAP restoration of degraded forest	RAP LUT (interpretation of mplc landscape)

1.3 Application of correction step for parametrization and calibration of initial simulation year conditions

The newly added parametrization step is used to adjust the original information by a given land cover map at a resolution ≤ 100 m, especially for areas of high tree/forest cover. It applies a set of user-defined rules for the initial simulation year (to be viewed as simulation time step 0, adjusting the input to available information that describes the starting conditions of the initial simulation year) to combine the provided land cover land use input map with further case study-specific land use information.

Firstly, the model adjusts the initial information of built-up by incorporating additional anthropogenic features to the landscape, namely, pixels of cities, settlements and streets. Secondly, the allocation of anthropogenic demands in agricultural LUTs (here: cropland-annual, agroforestry and pasture) is added to the land cover land use input map according to the set parametrized suitability criteria. Thirdly, pixels that were previously classified as undisturbed will be reclassified to LUT disturbed forest based on the user-defined anthropogenic impact distance. Based on further user-defined information which LUTs shall remain unchanged, i.e., land cover information that already depicts the terrestrial surface level, LPB applies the agricultural land use only on available land use types and only applies the simulation of anthropogenic

features to the former. The finally derived LULC map is then used as input for the Monte Carlo framework simulation. The described parametrization step is applied based on the model user's choice; otherwise, the initially provided LULC input map is used by LPB.

1.4 User-defined or automatically derived samples number in the footprint approach

We adapted the Monte Carlo framework to simulate landscapes at a sub-national, regional level in high spatial resolution with a probabilistic approach in reasonable simulation time and to serve the model targets.

Firstly, LULCC_basic now calculates in the Monte Carlo framework an average depicting occurrence probability for each particular LUT for the entire landscape to be used in the subsequent module in contrast to PLUC. Further probabilistic results are subject to the same procedure if the user chooses the option to simulate RAP.

Secondly, for the derivation of the averages based on samples, two approaches are implemented to determine the number of samples: (1) the original PLUC approach, where a produced Monte Carlo average is based on a user-defined higher number of samples for a random sampling approach (necessary in the PLUC demand/yield simulation); (2) the automatically derived required number of samples specific to the region based on user-provided data on agricultural land use realized in an implemented pseudo-random sampling approach. The latter effectively limits the number of samples to the automatically derived required minimum number of samples with the given spatial information in a pseudo-random sampling approach.

As the most influential variable for randomness in the smallholder and footprint approach and resulting landscape configurations, we identified the distance from an agricultural plot to a household. Therefore, the model has been coded to determine the required number of samples based on cell edge length and user-provided distances (minimum and maximum distance per LUT) of agricultural LUTs plots to households, respectively, settlements to patch – here applied for the singular settlement pixels. The calculation takes the minimum and maximum distance of all user-defined provided distances of agricultural LUTs into account, derives the combined maximum range, and by division with cell edge length, the required number of samples to depict the at minimum possible landscape configurations. For each sample, it calculates in the initial section the new maximum distance to be applied per agricultural LUT in allocation throughout the individual sample, starting with the minimum value in the first sample, adding one cell edge length per sample and ending with the maximum distance in the last sample.

This allowed for a simulation based on 49 samples for the case study area compared to a user-defined random sampling, which requires between 500 and 1000 samples in the PLUC demand/yield approach to derive a meaningful mean [1]. The advantage of this approach is the significantly reduced computation time and reduced TB data output. Such was only possible due to the reduced uncertainty element within LPB using an agricultural simple land footprint approach.

1.5 Parameters for regional case study simulation

LPB requires regional, case study-specific information to depict regional development pathways in LULCC_basic based on the rationale of smallholder land use patterns. This information can be drawn from cross-sectional household surveys, census-based data and other similar sources and influences the simulation of (1) dynamic settlements (e.g., by mean household size and mean impact distance of settlements based on the modal value of transportation), (2) agriculturally based land use change (e.g., agricultural demand per LUT, distances in meter, agricultural land use and smallholder share of population)

and (3) deforestation-driven demand by woody ABG-demands for subsistence purposes and per person (see S2 File section 1 for details on case study Esmeraldas Province). As a descriptive outcome, LPB provides regional and scenario-based yield projections ranging from potential minimum to maximum yields per projected year of up to five user-defined crop types of national or regional significance apportioned to cropland-annual or agroforestry use, which are calculated on the aggregated landscape in LULCC_mplc.

1.6 Scenario time series of anthropogenic demands projections

Anthropogenic demand is building on PLUC expressed in an area demand for a particular primary active LUT, following user-defined inputs of scenario assumptions. Four approaches are implemented, from which the first two refer to a simulation with a land footprint per smallholder capita and the latter two refer to PLUC model demand/yield approach. (1) For the option to simulate with the internally calculated demand in a static scenario of persistent patterns it mostly either depends on the simulated population total for a time step t (built-up and demand in woody AGB) or the simulated smallholder share of the population (agricultural types). In this case, plantations are simulated statically. (2) LPB-RAP also offers the option to simulate with an external user-defined dynamic time series (featured in this study). (3) The original PLUC/demand yield approach is implemented for agricultural land use types with the original stochastic behavior to derive demands per time step t. (4) Additionally, we implemented the PLUC demand/yield approach in a deterministic fashion, which enables the user to simulate different guideline scenarios with the same demand. The Demand/Yield approach is not further featured in the following subsections. Therefore, smallholder demands as a proxy for the total population demands in a landscape here define the simulation and the model application. For all LUTs, the basic assumption is that in the future all demand must be allocated if plausible (i.e., if demand can be satisfied within the available areas of land and topographic conditions). This eventually depends on the size of the study area and chosen restriction scenario, as LPB does not allow for spill-over effects that could lead to indirect land use change or leakage [2]. The simulation of the status quo, respectively land use demands with a persistent pattern, is interpreted here as a “moderate worst-case scenario” regarding the Agenda 2030 based on the assumption that a progressive scenario enabling Agenda 2030 goals realization would entail SSP1 measures, SSP2 persistent patterns already would entail a miss of Agenda 2030 goals and all other SSP scenarios likely depict even further deviation from the set goals.

1.6.1 Built-up LUT (default)

For long-term development and in a hectare resolution, we assumed that built-up had to be depicted dynamically, contradicting to PLUC. Therefore, demand in built-up (representing housing and infrastructure as well as all compacted area in a hectare resolution) is incorporated by a simple proportional approach per time step t based on population and simulated existing built-up, p.r.n. under incorporation of new settlement pixels. The effective simulation of the combination of demand and suitability settings can be characterized as „spontaneous growth’, „spreading-center growth’, „edge growth“ and „road-influenced growth“ in an urban sprawl context [3]. The simulation magnitude can be user-defined adjusted by a setting for consideration or non-consideration of street pixels.

1.6.2 Agricultural LUTs (description for footprint approaches)

To express demands in food-based LUTs of cropland-annual, agroforestry -which includes perennial cropping systems and agro-silvopastoral systems- and pasture, we incorporated a simplified land footprint approach (i.e., average land area in hectare used per agricultural LUT per smallholder capita, not in the meaning of the ecological or environmental footprint approach). This enables the use of cross-sectional survey information instead of time series for yields and livestock choices (see S2 File section 1.1 for details on the case study Esmeraldas Province). Within the static modeling approach, the land footprint per agricultural LUT is applied to the regionally defined smallholder share of the population, which is derived dynamically for each time step t. For a dynamic depiction, as presented here, the user has to parametrize and calibrate time series externally, which offers the choice to also include variations of the smallholder share as well as plantation and wood demand development according to scenario assumptions. In this approach, we utilize a kilocalorie (kcal) intake per person scenario projection as well as a societal diet change projection, which can be regionally adjusted to a scenario narrative based on underlying socio- and macroeconomic assumptions (see S2 File section 3.1.12 for further details on the case study). For t_1 = initial simulation year the primary data is used as an input, for t_2 to t_n projections based on scenario assumptions are incorporated. To derive the time series the following two equations are employed:

Equation 1 – applied to t_1 = initial simulation year per agricultural LUT with primary data of footprint and scenario data of smallholder population, derived from primary data and remote sensing:

$$\frac{kcal\ demand}{(smallholder\ population * \boldsymbol{smallholder\ footprint})} = adjustment\ factor$$

Equation 2 – applied to all consecutive simulated time steps with scenario projections of kcal demand and smallholder population:

$$\frac{kcal\ demand}{(adjustment\ factor * smallholder\ population)} = \boldsymbol{smallholder\ footprint}$$

Whereby:

kcal demand	=	Calculated scenario-based average kcal demand of the total population to be satisfied per smallholder capita per agricultural LUT
smallholder population	=	Calculated scenario-based rural population share of total scenario-based population
smallholder footprint	=	The smallholder footprint in ha per smallholder capita per agricultural LUT
adjustment factor	=	The required mathematical bridge to link primary data of LUT footprint to the scenario assumptions and remote sensing-based regional information

The primary data smallholder footprint already includes fodder for livestock production in the region. The calculated adjustment factor expresses a range of elements that cannot be depicted in greater detail, for example, fertilization techniques and their potential state subsidiaries or the import of (partially ultra-processed) goods to satisfy demand, and in this case (statically applied) also has a legacy component. To explore the calculation example used for this study see S3 File.

1.6.3 Plantation LUT (depending on approach)

The plantation LUT is a challenging subject in LPB respectively for regional parameterization. Firstly, the user must define if plantations describe a woody LUT in the simulated landscape or other crops, e.g., tea or cotton. If a woody LUT is displayed in the parameterized initial LULC map, the user must parameterize if it handles in the majority crops such as oil palm, coconut and rubber or, on the contrary, timber plantations. LPB can consider for all types of plantations short- to long-term rotation periods by the simulation of the user-defined harvest mean rotation period and subsequent for timber types simulation of user-defined AGB increments. LPB allows for uncertainty since the land use management patterns can be differentiated from forest types. Only if the plantation LUT refers undoubtedly to timber, the user may consider the plantation LUT as gross forest, as shown in the FAO classification [4]. Within the static footprint approach the land use type plantation is handled statically in the model, i.e., the base demand is derived in time step t=1 after the potential correction step from the remaining number of pixels of plantation in use and plantations harvested. For the static footprint approach in the no conservation scenario, applied to a time step t ≥ initial simulation year based on weak conservation or enforced conservation output, the user must provide the information of the demand derived in weak conservation or enforced conservation. The information is then passed on as a static deterministic demand during the dynamic simulation. For the dynamic footprint approach, plantation development can be calibrated to scenario assumptions based on projections. For case study application see S2 File section 3.1.12 and S3 File.

1.6.4 Woody above-ground biomass (AGB)

Within the static footprint approach, the demand in woody AGB is calculated based on the newly coded user-provided combined information of subsistence demands for timber, fuelwood and charcoal in Mg. It is possible in the model design to incorporate information on additional biomass lost via the extraction of these base demands by logging. One must bear in mind that this must be done on an average basis and applied to the whole population of the study region per time step t, as urban population demands, in this case, must be satisfied too (see for further details on Esmeraldas Province: S2 File sections 1.5 and 3.1.4). On the contrary, within the dynamic footprint approach featured here, the wood demand in Mg is provided externally in the demands time series and can be subject to further scenario assumptions. For case study application see S2 File section 3.1.12 and S3 File.

In the current LPB-RAP model, the user has different simulation choices. If the user wants to simulate potential local forest degradation and the according RAP-LUT25, it firstly employs smallholder wood demand extraction around settlements (AGB > 0 Mg) and only the rest demand is added to the urban population wood demand, which results in LUT17 (AGB = 0 Mg). Otherwise, only LUT17 is simulated (then resulting in more area affected).

In the case of smallholder wood demand extraction, the model considers a user-defined distance to create a buffer region around settlements, then it evaluates how many pixels qualify as forest and divides the AGB demand accordingly. Only pixels that can accommodate this demand fraction are used. It then loops over the pixels with the same procedure and remaining demand again and again, until the demand is satisfied or cannot be further satisfied.

The demand in woody AGB in Mg by the urban population will be subtracted from the net forest fringe (LUT17 net forest - - deforested). To simulate LUT17 the model assesses the current simulated AGB content in Mg in the forest fringe cells and subtracts cells of suitability until woody AGB demand is satisfied. For the application of deforestation, the model assumes that first unrestricted areas are considered in a time step; additionally, the user must provide a maximum slope value until deforestation is possible. Only if demand per time step t exceeds the AGB provided by deforestation of the current state of the available forest fringe

does the model also consider restricted areas when it iterates again. The iteration stops for the time step, if the demand is satisfied, no more net forest or no more suitable slopes for deforestation are available. Note that potential wood demands satisfied in and yields provided by silvicultural practices of subsistence or commercial backgrounds, which may occur in forest landscapes, are not yet part of the model, such shall be incorporated in further foreseen model development stages.

2 LPB-RAP further new model specifics compared to PLUC and user-defined methods

2.1 Landscape and topography differentiation

LPB excludes user-defined areas, land use types and topography-specific areas similar to PLUC. The model further divides slope restrictions. It now does not only distinguish between maximum suitable slopes and inaccessible slopes but has a threefold range: favorable terrain, difficult terrain and inaccessible terrain, each user-defined for a respective primary active LUT. The basic assumption that guides this rationale is that favorable terrain is used first by LULCC_basic to allocate the demand for land use during a timestep t. In contrast, difficult terrain can be made only accessible by increasing management efforts if the demand for land use increases. Based on user settings, these ranges can be adjusted per active LUT or serve as a basis for scenario simulations.

2.2 Simulating impacts on forest extent, forest habitat quality and forest AGB quantity

The differentiated forest dynamics simulation of LPB-RAP is depicted in Fig A4 and the section below. Minor methods involved are depicted in the following subsections.

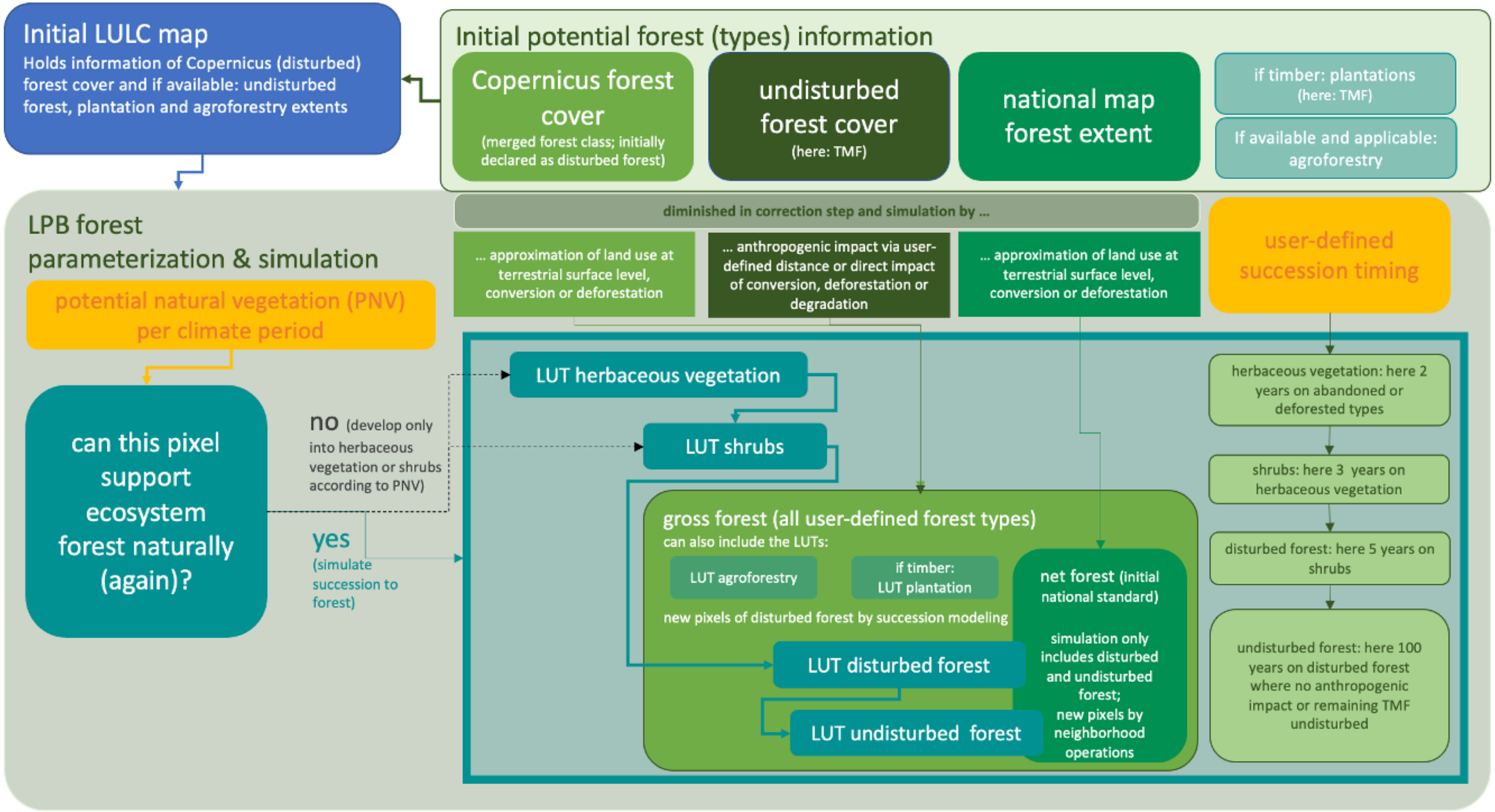

Fig A4: *The diagram displays the combined inputs and processes applied to forest information within LPB to approximate the number of forest pixels with assumed forest site characteristics at the terrestrial surface level (LUT08 = disturbed forest and LUT09 = undisturbed forest). In the case study, agroforestry is approximated by simulation (see for the input map alteration S2 File section 2.5 and for the consecutive correction step S1 File section 1.3.) and the type of plantations depicting oil palm, not a timber plantation respectively forest type (for further details, see S1 File section 1.6.3 Plantation LUT). Details of PNV are given in S1 File section 1.1 and S2 File section 2.8.1. For further details on implemented user-defined succession, see S1 File section 2.2.4 and S2 File section 3.1.10.*

Within the model, the LUTs disturbed and undisturbed forest describe an assumed quality of the pixel regarding anthropogenically caused disturbances. Disturbed forest pixels in LPB follow the rationale that these areas are affected directly (explicit pixel) or by the extended neighborhood area, such as agricultural uses, and by anthropogenic impacts over a user-defined distance (this relates primarily to the usability as habitat). For further details, see S1 sections below and S2 File section 3.1.2. Undisturbed forest pixels are only describable via remaining pixels of initial a priori classified information or not affected (any longer) by the simulated anthropogenic use. The differentiation was done to emphasize forest habitat quality and accessibility. Undisturbed and disturbed forest can also be interpreted as old-growth and secondary forest pixels, where secondary is applied to a pixel impacted by anthropogenic use or as a pixel in the state of forest re-growth. Depending on user-defined parameter input, undisturbed forest areas can only increase if a disturbed pixel (as part of the succession stages during a simulation) reaches a by the user defined age (see for example [5]) continuously without anthropogenic impact. For demonstration purposes, we simulate a century as the default value, which is just used as a model convention; depending on user needs, this value can be adjusted to any other value.
A further separation of gross and net forest is implemented in LPB to acknowledge different forest definitions and thus forest extents that often exist alongside each other. In the case of this study, the merged Copernicus-based land cover map defines forests to have a minimum of 15 % tree cover (gross forest), while the Ecuadorian national LULC cover map product defines „native forests“ by a more ecological definition, but basically to have a minimum of 30 % tree cover [6] (net forest). Hence, both describe in parts overlapping different initial forest extents in the case of the Esmeraldas region and potentially other to be simulated forest landscapes (see Fig A4). A discrepancy depends on the modeling area and its applied country definition and study context (see S2 File section 2.5 for further details on the case of Esmeraldas Province). The differentiation comes into effect when determining deforestation and restoration targets, as this is handled in LPB-RAP as a user-defined to-be-reached increment applied on net forest extents, to connect to potential national goals via the applied standard.

To describe forest quality, particularly regarding habitat functions, the model applies different methods to account for anthropogenically caused disturbance approximation:

2.2.1 Dynamic net forest extent

In contrast to PLUC, the simulation of deforestation for demand in input biomass has been adjusted in LPB to take only net forest pixels into account since the gross forest in the here applied assumptions can also contain private gardens and communal parks, et cetera. The net forest is simulated dynamically based only on undisturbed and disturbed pixels as an approximation of forest site quality and can therefore experience contraction (conversion and deforestation for subsistence demand in input biomass) as well as expansion (disturbed pixels re-growth by succession at net forest fringe).

2.2.2 Anthropogenic impact distance

This user-defined parameter simulates a buffer around anthropogenic features (cities, settlements, streets) and overlapping undisturbed pixels (remote sensing-based or simulated) will get reassigned to a disturbed status. The parameter can user-defined represent different kinds of impacts as applicable, e.g., impact distance of noise, anthropogenic use frequency, reach of contaminants in air and soil, etc., which will impact the site quality, especially concerning habitat functions. The range of the applied distance can therefore vary between m and km. Only the provided chosen value is used.

2.2.3 Disturbed forest fringe

The forest fringe is dynamically simulated as disturbed as an approximation of land use in the immediate neighborhood, e.g., streets, agricultural land use or deforestation. Overlapping pixels will, therefore, not be

simulated as undisturbed - the succession age is set back to 1 year. Only if these pixels are no longer under anthropogenic influence the succession age counts up until the user-defined age of disturbed forest without further impact is reached; only then is the transformation to undisturbed forest simulated.

2.2.4 Succession

Succession is based on the biome information of the potential natural vegetation maps per climate period and restricted to the basic land use types depicting succession stages in the Copernicus-based-LUTs range. The model currently distinguishes the generic classes grassland (i.e., herbaceous vegetation), bushland (i.e., shrubs) and forest (i.e., disturbed and undisturbed forest) only. Forest succession can only occur where a forest biome pixel is located. Succession stages time frames are provided user-defined in column tables and can be adjusted to the simulated landscape. The last succession stage is undisturbed forest; this only applies if an original disturbed or succession-based re-growth pixel reaches a user-defined age without any anthropogenic impacts.

2.2.5 AGB simulation

Based on the spatially explicit AGB map for the initial simulation year and stochastic or spatially explicit increments, the model simulates increasing AGB by annual increments until the potential maximum undisturbed AGB per cell is reached. AGB in this model is only counted for simulated gross forest pixels to be able to derive the agglomerated and singularly differentiated effect of forest types in the form of potential carbon sequestration. This may underestimate the total AGB in the landscape indicated by tree cover or other biomass sources but sharpens the estimate for forest types, where forest cover indicates actual forest-associated site qualities in different forest types at the terrestrial surface level.

2.2.6 Dynamic forest degradation & regeneration simulation and RAP-LUT25

In order to approximate within dynamic modeling on a climate change background and in annual resolution the state of the future forest in this pixel-based approach we implemented an innovative automized and user-steered categorization. The approach borrows from the postulate of a quantitative measurement application for IPCC definition criteria and remote sensing techniques using AGB measurements as a basis for automized classification.

To depict the annual development the model draws the two major categories per simulated pixel for AGB development: less AGB than last time step signifies overall degradation, while equal or increased AGB content signifies overall regeneration. Both categories are divided into four classes depending on the range of currently simulated AGB content in relation to a potential maximum value for undisturbed forest modeled per pixel per climate period. A new AGB value of 0 Mg AGB signifies complete deforestation or here the class “degradation absolute” at the lower range of the spectrum. On the upper range of the spectrum resides a 100 % AGB content in relation to the potential maximum, here signified as the class “regeneration full” (“regeneration absolute” would only be qualifying for a pixel, that is additionally again simulated as undisturbed, therefore the term is not used here).

The range between > 0 % and < 100% is divided into three classes per degradation and regeneration by two user-defined percent values. These can be adjusted regionally based on primary or secondary data, for case study parametrization see S2 File section 3.2.3. For degradation, these three classes describe the state of partially logged forest by anthropogenic local wood consumption simulated as AGB extraction around settlements as an AGB loss and an applied AGB increment as an AGB gain in sum for each year. Within this

model, the classes describe mainly forest degradation stages for logged-over but not entirely deforested pixels besides the regrowth of secondary or growth of parametrized forest.
RAP-LUT25 uses the user-defined selected classes of at maximum degradation severe, moderate and low to describe the potential of restoration of degraded forest. For case study parametrization see S2 File section 3.2.3 and 5.4.
Table A4 summarizes the applications set by the two user-defined AGB thresholds in percent:

Table A4: User-defined AGB thresholds and simulation of degradation, regeneration and restoration

Regeneration classes [AGB gain]	Simulated AGB content % in relation to modeled potential maximum undisturbed value	Degradation classes [AGB loss]	RAP-LUT25 user-defined input
Full	100		
High		Low	1 to 3 user-defined degradation classes can be selected
**Upper user-defined AGB threshold**			
Medium		Moderate	
**Lower user-defined AGB threshold**			
Low		Severe	
	0	Absolute	

2.3 Systemic choices and gradual simulation options

In addition to the existing allocation order, a range of systemic choices has been implemented via user-defined settings to provide a model user with the ability to depict regionally varying systems and specifics or simulate diverging scenario stages, described in the following sections.

2.3.1 Deforestation prior to or post-conversion to other LUTs

The model can simulate deforestation due to subsistence-driven woody AGB demands, which is simulated at the net forest fringe and if chosen also as local AGB extraction. The algorithms can be applied prior to or post conversion to other LUTs (all conversion is simulated en bloc), depending on the context to be simulated. If deforestation for demand in woody AGB prior to conversion is chosen, this information might not be depicted any longer in the resulting sample time step map and, therefore, could not be recognized in the subsequent aggregation module. This can occur either because these pixels are subsequently converted or due to low probabilities for remaining deforested plots based on the different locations in all samples. In this case, LPB, therefore, evaluates the pixels of LUT17 “net forest - - deforested”, which remain after the allocation of primary active LUTs in the LULCC_basic module. This evaluation is done for each time step in each sample to calculate the mean value per time step. The information is passed on to the subsequent module LULCC_mplc for the factual mplc because deforested sites also serve as input for the RAP simulation.

2.3.2 AGB increment stochastic or spatially-explicit

The model user can choose to simulate AGB increments stochastically in a user-defined range, simulated based on a uniform distribution, or spatially explicit depending on available data. With a stochastic range, climate-based variation in the landscape cannot be depicted and no climate change signal can be incorporated. Spatially explicit simulation can provide both but relies on external data quality and could not be tested for the Esmeraldas region due to quality flag 3, i.e., „improbable change', of the ESA AGB V3 increment 2017 to 2018 data.

2.3.3 Street network as input for dynamic built-up simulation

Depending on raster resolution and to be depicted system or assumption, the user can choose to simulate dynamic built-up with or without incorporating street pixels. Using this information leads to a significant increase in simulated built-up and distinct urbanization patterns and might account for the missing housing footprint in the regional simulated total area for built-up in high resolutions. Here, a housing footprint was not evaluated in the primary data collection and could not be derived from secondary sources for the current time frame and the global south or the specific case study area; therefore, the incorporation of street pixels is applied as a moderate worst-case scenario assumption. Simulation without incorporating street pixels shows less landscape transformation to the final land use type. However, it may underestimate the amount of transformed total area at the terrestrial surface level in the landscape drastically. See S2 File section 5.2 for details on Esmeraldas Province.

2.3.4 Dynamic settlement simulation threshold

A settlement development algorithm was newly implemented in LPB to serve as a dynamic suitability factor. Settlement growth is directly related to population growth and will only occur when the population increases. Firstly, the model considers the population as apportioned in the parameterized mean household size for the particular region outside the also parameterized draw area of existing settlements. Secondly, it simulates based on the population number and settlements number of the last time step potential new settlements based on a window operation taking population in the settlements draw area into account. Thirdly, final new settlements are only simulated where the agglomerated pixel value matches or exceeds the user-defined threshold of households required for a new settlement in the number of proportionally calculated required new settlements. Another user-defined regulating parameter of simulation to depict regional settings is, therefore, the required parameter of the number of households needed during the simulation to form a new settlement. For the case study of Esmeraldas province, we calculated an average of four persons per household based on household survey data. Since the term "settlement" is not clearly defined by a number of households or inhabitants, we here chose a relatively strong signal as the default value of 100 households to form a new settlement in accordance with the systemic break of settlements to cities (which incorporates towns) at larger villages; thus, for the case study area, a pixel must match or exceed a value of 400 inhabitants and be situated out of the drawing area of existing settlements to form a new settlement in the presented case study. If lowered, the simulation depicts a higher number of new settlements per time step while population growth continues, where each settlement functions as an epicenter of new simulated land use for the allocation order.

2.3.5 Slope inclination levels per primary active LUT

In contrast to PLUC, which differentiates only current suitable slopes from inaccessible slopes, we here differentiate the class “difficult terrain” additionally. Difficult terrain refers to presently rather unused slopes for primary active LUTs in the region, which are potentially suitable slopes for land use under assumed progress in technology or simply by the adaptation to land cultivation forms prior not being used in the landscape, e.g., terrace agriculture. We associate higher labor or monetary investment cost with such plots, which is why they will be only simulated as used if favorable terrain is no longer available. The user can define gradual variations of slope levels via (1) current used slopes derived in GIS approaches or literature-derived ranges; (2) in primary studies gathered information on current slopes (favorable and difficult terrain); or (3) scenario assumptions. This information is used in the cascading allocation of LULCC_basic per primary active LUT. For details regarding the default settings used in this case study, see S2 File section 3.1.7.

S1 File references

1. Verstegen JA, Karssenberg D, van der Hilst F, Faaij A. Spatio-temporal uncertainty in Spatial Decision Support Systems: A case study of changing land availability for bioenergy crops in Mozambique. Comput Environ Urban Syst. 2012;36(1):30–42.
2. Meyfroidt P, Roy Chowdhury R, de Bremond A, Ellis EC, Erb KH, Filatova T, et al. Middle-range theories of land system change. Glob Environ Change. 2018;53:52–67.
3. Gomes E. Sustainable Population Growth in Low-Density Areas in a New Technological Era: Prospective Thinking on How to Support Planning Policies Using Complex Spatial Models. Land. 2020 Jul 8;9(7):221.
4. FAO. Global Forest Resources Assessment 2020. 2020.
5. Poorter L, Craven D, Jakovac CC, van der Sande MT, Amissah L, Bongers F, et al. Multidimensional tropical forest recovery. Science. 2021 Dec 10;374(6573):1370–6.
6. Ministerio del Ambiente. Ecuador's Forest Reference Emission Level for Deforestation. 2014 [cited 2022 Dec 13]; Available from: https://redd.unfccc.int/files/2014_december_frel_submission_ecuador.pdf
