# Supplementary material for: Quo vadis, smallholder forest landscape? An introduction to the LPB-RAP model
Source: PLoS One. 2024 Feb 2;19(2):e0297439. doi: 10.1371/journal.pone.0297439 (PMC10836681; doi:10.1371/journal.pone.0297439)
Supplement: S2 File — (PDF) [file pone.0297439.s002.pdf]

S2 File - Case study modeling: Scenario tool Parametrization and Calibration

S2 File provides besides some case study simulation background information the description of the required model data input in a twofold approach: using (1) primary (land use) survey data as well as (2) secondary data sources of maps and literature or expert opinion and scenario assumptions. The model parametrization and calibration for this scenario tool is based either on empirical data for regional simulation or on assumptions for default values regarding a) the initial simulation year starting conditions or b) the long-term simulation for conditions that may not be yet in place but may be a factor in development for the 21st century under the assumed increase in pressure (lack of trans-regional evasion space in combination with population development).

S2 File to: "Quo vadis, smallholder forest landscape? An introduction to the LPB-RAP model."

CONTENTS

Please note: (new) or (adapted) refers to LPB-RAP components in comparison to the base model PLUC

Case study area background information 4
Case study parametrization and LPB-RAP model default settings.....5
1. REGIONAL PRIMARY DATA (new): 6
1.1 Footprint and derived regional smallholder share 7
1.2 Distances 8
1.3 Mean household size 8
1.4 Most important crop types and yield levels 8
1.5 Wood Density 9
2. SECONDARY SPATIAL DATA 10
2.1 Administrative boundaries 10
2.2 Anthropogenic features and surface freshwater based on OSM 10
2.3 Restricted areas (new) 12
2.4 Terrain 13
2.5 LULC map 15
2.6 Initial net forest (new, based on national LULC map) 17
2.7 Baseline scenario determinant population (new/adapted) 18
2.8 Baseline scenario climate data-based determinants (new) 18
2.8.1 Potential natural vegetation 19
2.8.2 Potential maximum undisturbed AGB 22
2.8.3 Potential forest types annual AGB increments 25
2.9 Initial AGB map (new/adapted) 25
2.10 Excluded areas map (based on PLUC).....25
2.11 Initial plantation age map (new) 26
2.12 Other ecosystems map (new, RAP application) 26
2.13 Null mask 26
3. SECONDARY PARAMETERS DATA, EXPERT OPINION AND SCENARIO ASSUMPTIONS 27
3.1 LPB (new or adapted).....27
3.1.1 Allocation order (adapted) 27

3.1.2 Distances describing anthropogenic impact on and use of forest (new) 27
3.1.3 Demand in input biomass in Mg (adapted) 27
3.1.4 Annual AGB increment ranges (new) 28
3.1.5 IPCC biomass to carbon conversion factor (new) 29
3.1.6 PLUC core residual parameters 29
3.1.6.1 Related land use types (adapted) 29
3.1.6.2 Suitability factors, their weights and parameters (adapted) 29
3.1.6.3 Land use types on which no allocation occurs (adapted and new) 35
3.1.7 Terrain restrictions parameters (adapted) 35
3.1.8 Maximum slope for deforestation value (new) 36
3.1.9 Mean plantation rotation period end (new) 36
3.1.10 Succession time frames (new) 36
3.1.11 mplc module corrective allocation input (new) 37
3.1.12 External time series of active land use demands calibrated to baseline scenario assumptions (adapted) 37
3.2 RAP (new) 41
3.2.1 Net forest increment goal in percent 41
3.2.2 List of LUTs for definition of potential restricted areas 41
3.2.3 Forest degradation and regeneration AGB thresholds (new) 41
4. CORRECTION STEP SETTINGS (new) 41
5. SYSTEMIC CHOICES SETTINGS (new) 42
5.1 Deforestation prior to or post-conversion to other land use types 42
5.2 Incorporation of street pixels for simulation of dynamic built-up 42
5.3 Required number of households for a new settlement 42
5.4 RAP-LUT25 inputs 42
S2 File references 43

Case study area background information

The implementation example Esmeraldas province is located in the northwestern part of Ecuador, bordering Columbia to the north and the Pacific Ocean to the west:

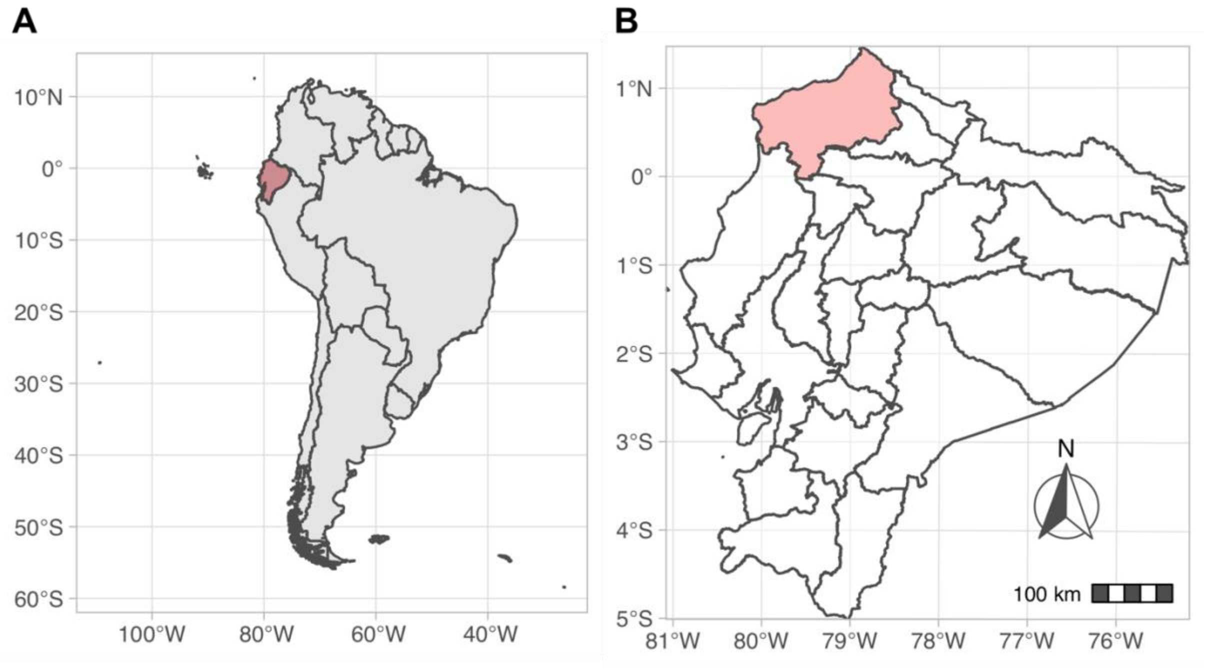

***Case study area location**: Depicted is A) Ecuador in the continental context and B) the provincial case study area Esmeraldas in the country context (mainland without Galapagos islands). **Map sources**: South America shapefile downloaded from http://www.efrainmaps.es. Carlos Efraín Porto Tapiquén. Geografía, SIG y Cartografía Digital. Valencia, Spain, 2020. Ecuador data by gadm.org and naturalearthdata.com*

The Andean foothills form a natural boundary to the eastern and southern parts and partly reach the province. The province comprises 16,132 km^2 with a population of 534,092 inhabitants (from 385,223 in 2001) according to the 2010 census. To illustrate a potential model application, we present in the following parametrization and calibration for the simulated case study area (> 1.67 million ha, including an applied buffer of 1250 m around the administrative border).

Case study parametrization and LPB-RAP model default settings

In the following sections, the specifics of the case study area parametrization (initial status and either static or alternated dynamic long-term depiction) are provided. Sections cover topics of field survey data (1), secondary spatial information (2), parameters and data derived from literature and parameter settings based on expert opinion or scenario assumptions (3) and user-defined simulation choices (4 and 5).

1. REGIONAL PRIMARY DATA (new):

The primary source of information for model parameterization in the case of the Esmeraldas Province refers to the LaForeT project (www.la-foret.org) which conducted household surveys among other field-based inventories during 2016 and 2019 in 12 landscapes of Ecuador (of 36 in total, covering also Zambia and the Philippines). In the Esmeraldas region, a total of 423 household surveys were conducted by LaForeT in four landscapes, each covering an area of approximately 10x10 km, representing 1,542 people of all age classes. The combined share of the four studied landscapes depicts 41,198 ha or 2.45 % of the simulated regional landscape of 1,678,488 ha.

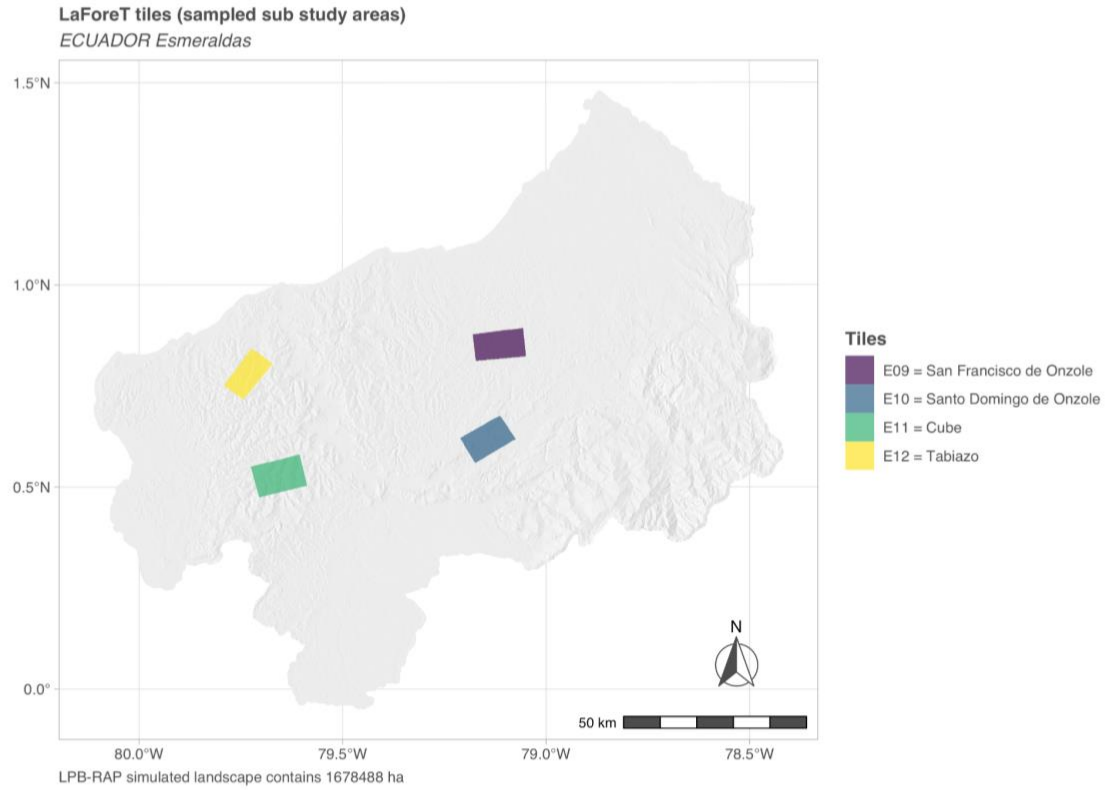

Figure B1 The aggregated results of the sampled subset study areas were used for extrapolation to the regional extent of the province.

The survey data in the sampled regional subset areas (see figure B1) comprises the surveyed LUTs, area of managed farming and forest land, distances to managed plots and reported crop yields for the interviewed 423 households and their managed 1,213 plots (for survey methodology see [1]. Land use was assessed by LaForeT in a four-tier system, with level 1 depicting aggregated LUT categories such as forest or water to a very diversified level 4 which for example includes detailed temporal information of forest type categories. For modeling purposes, we rely on tier 1 to tier 2 LUT information. The survey raw data were cleaned by expert opinion and trimming, using the interquartile range method (IQR) and winsorization with lower and upper fence from the IQR to eliminate extremes.

For this study area we derived at a total of 4.72 ha land per capita for smallholder land use purpose, from which 2.45 ha are simulated as active agricultural LUTs for smallholder purposes in the model. Fuelwood demand is apportioned by demand per person for the total population (as urban areas have to be

accounted for too). Since housing was not captured in the LaForeT questionnaire no explicit built-up demand was derived accordingly. The discrepancy between land use per capita evaluated and simulated relates to the individual forest parcel ownership, which cannot be acknowledged in the LPB concept as it would require to add further agent-based model functionality to the model design [2]. A second factor are farm area LUTs which refer to aggregated, passive, static, or successional active LUTs in case of LBP, such as wetland, aquaculture, or grass- and bushland. The latter one is not used as pasture area. On household level mean farm area was evaluated as 16.7 ha, with a mean cropping area of 4.12 ha and mean forest area of 7.14 ha per household (average of four persons, three adults above the age of 14).
Land use demand related to commercial farming activities, e.g., cash crops were not evaluated in LaForeT and no secondary data for the initial simulation year for commercial regional demand for wood, cropland and pastures were available for the project regions. Therefore, LPB follows the notion of simulating spatial patterns predominantly influenced by smallholder land uses and subsistence needs as evaluated in the LaForeT project (see for further details, e.g., [1,3,4]. This is further supplemented by information of plantations as depicted in the TMF dataset (see section 2.5). We decided for this procedure because downscaling available national data on demands for wood and cropland to regional extents was disregarded due to potential scaling problems (such as missing tree species distributions for timber demands and unclear distribution of different crops in the landscape). Additionally, historical census data may not depict the actual demand at the initial year of simulation in 2018. As the smallholder share (see section 1.1) of the study region is approximated from LUTs related to agriculture for the case of the national LULC map (see section 2.5; with a subtraction of LUT plantations from the TMF layer (see section 2.5)) and the projected population value, commercial demand for LUT cropland-annual and LUT pasture is simulated as a static fraction of the smallholder share. With this approach the discrepancy between the population data provided by the Ecuadorian census data (2010: Esmeraldas 534,092 persons) and population projections based on 2000 data (Esmeraldas plus buffer 2010: 412,483 persons; 2018: 457,430 persons, see section 2.7) is accounted for. Regarding the simulation of wood demand, it remains unclear whether the overall effect of missing data of commercial wood demand in the landscape (not accounted for in plantation areas), depicts an underestimation in the considered landscape transition.

1.1 Footprint and derived regional smallholder share

For the primary active agricultural LUTs depicting area demand, we calculated demand per person based on the LaForeT survey data (farmland/land use area managed) as described above. These data however were not compatible completely with the remote-sensing based LUTs (see table B4), but applied where possible (simulation of cropland-annual, pastures and agroforestry, see section 2.5).

To derive the smallholder land footprint, the evaluated sum of hectares used for each active LUT was divided by the total number of persons in all households that were interviewed by LaForeT. We included children under the age of 15 since the population projection data for the SSP2 does not discriminate in age groups, therefore LPB relies on the total population per year that must be accounted for during a simulation. Table B1 shows the derived regionally values of demand per capita in hectare per LUT as the basic principle of the land footprint approach:

Table B1 Land footprint (in ha) per smallholder derived from LaForeT survey information

active agricultural LUT	land footprint in ha per smallholder head
**cropland-annual**	0.04735

**pasture**	1.23802
**agroforestry**	1.16852

Since the study region of Esmeraldas consists also of population in urban and peri-urban areas, a simple projection of land use demand per person would result in a 51.7 % area excess compared to the reported national data of 2018. We, therefore, approximated the percentage of smallholders with the calculated LaForeT area demand based on the reported total agricultural area and the total population calculated by linear interpolation model internally based on the SSP2 datasets for 2018. This further required us to subtract the TMF (see section 2.5) plantation area from the national land use map product in the pre-step. The initial percentage of smallholders in the total population was calculated as 53.87 %.

1.2 Distances

The LaForeT survey provided distances for different transportation modi to agricultural plots. We extracted the modal value of transportation for each of the 36 landscapes, which resulted in the transport mode 'by foot'. Accordingly, we extracted information on walking distance (in m) from a particular household location to a managed farm plot per region. LPB uses this information for the simulation of new settlements and for the allocation of LUTs per timestep t. Hence, all related survey data were selected from the LaForeT databased and IQR and winsorization was applied. The mean value for all recorded survey observations was then calculated to define the radius of anthropogenic impact in relation to the mean settlements impact area which in the case of Esmeraldas refers to 1,710 m.
Using a similar approach, the minimum, maximum and mean walking distance per agricultural LUT was derived (table B2). This information is required to simulate land use patterns with the pseudo-random sampling approach.

Table B2 Agricultural distances derived from primary survey data used for LPB simulation

LUT	minimum distance	maximum distance	mean distance
**cropland-annual**	10 m	2,200 m	791 m
**pasture**	5 m	3,600 m	906 m
**agroforestry**	5 m	4,850 m	1,184 m

1.3 Mean household size

To depict the regional development of settlements dynamically the model requires, besides the number of required households for a new settlement, the information of the regional mean household size, to relate to the population data. Based on the survey data we applied a value of 4.

1.4 Most important crop types and yield levels

We determined the five most important crops per country from the LaForeT survey data to estimate regional crop yields based on simulated future land use cropping areas as a descriptive feature. We calculated individual and combined percentage of all regionally used cropping areas and then separated them into agroforestry and annual cropland. All reported yield values from the survey dataset were

cleaned by removing implausible values of > 100 Mg ha^{-1} a-^{1}, following IQR and winsorization, using FAO national level information as a reference. Mean and standard deviation were derived to parameterize LPB under the assumption that crop yields remain constant for the modeled time frame and hectare basis. To account for annual weather variability, which cannot be captured by LPB, the model provides potential crop yields with a minimum, mean and maximum value based on standard deviations. For the Esmeraldas province, the applied five most important crop types and yield are shown in table B3:

Table B3 Top crops yields shows the from primary data derived annual yields applied for regional modelling.

Crop	mean yield in Mg ha^{-1} a^{-1}	standard deviation yield in Mg ha^{-1} a^{-1}	standard deviation adjusted yield in Mg ha^{-1} a^{-1}	percentage of cropping area of agroforestry or cropland-annual	agroforestry or cropland-annual application
**Cacao**	0.90	0.70	-	82.03	agroforestry
**Coffee**	0.14	n.a. (only one observation)	0.01	0.07	agroforestry
**Platano**	4.82	6.1	4.81	12.89	agroforestry
**Maize**	1.03	0.89	-	51.77	cropland-annual
**Cassava**	5.99	7.04	5.98	10.77	cropland-annual

1.5 Wood Density

LaForeT further conducted forest inventories to derive information such as aboveground biomass, tree species composition and soil features. We rely on the reported tree species information to calculate the regional tree species wood density based on the weighted mean of all evaluated species (see section 3.1.3).

2. SECONDARY SPATIAL DATA

This chapter describes the spatial inputs required for LPB and the land cover to land use parameterization step which is executed before time step t=1 before the dynamic components of LBP-RAP commences.
As a basic spatial model feature, the Copernicus Land Cover map (see section 2.5) was chosen to allow for global model applicability and as a depiction of the "smallholder scale". Following LPBs spatial resolution of 100 m, all spatial data were harmonized to a 100 m pixel size (Projection: WGS 84 UTM 17 S; EPSG: 32717) and converted to the PCRaster map format. QGIS 3.16.7+ was used for most mapping calculations while SAGA GIS version 8.0.0+ was used for spatial inter- and extrapolation, regression and projection operations. For analysis and visualization, final operations were done using R 4.2.0+ and R Studio 2022.02.2+.

2.1 Administrative boundaries

We rely on administrative boundaries to define the spatial extent of the simulated area as a reference for policy development and implementation. We used administrative levels to determine the regional extents to encompass LaForeT landscape tiles located in Esmeraldas province. For the Esmeraldas region we used GADM [5] boundary information to take the impact of cities as potential means of settlement expansion into account, plus an additional buffer of 1,250 m to the province's extent to capture the Esmeraldas coastline and the province border area, and to account for the simulation of fringe effects.

2.2 Anthropogenic features and surface freshwater based on OSM

The OSM [6] vector database was used to initially allocate the anthropogenic landscape features of cities, settlements streets and location of surface freshwater (incl. rivers). OSM data were downloaded using the QuickOSM plug-in [7] in QGIS.
For information on the distributions within the LPB layer "cities" we chose the OSM datasets place:city and place:town, which includes the location of the provincial capital Esmeraldas and 10 towns, all agglomerated in the cities layer (n = 11).
For information on the LPB "settlements" layer, we chose the OSM datasets place:hamlet and place:village resulting in 743 + 84 (n = 827) data points to be recognized as initial 1 ha pixels.
For information on the regional "street" network, we rely on the OSM highway categories: trunk, primary, secondary, tertiary and unclassified. These were merged and converted to one raster dataset, resulting in n = 48,649 pixels.

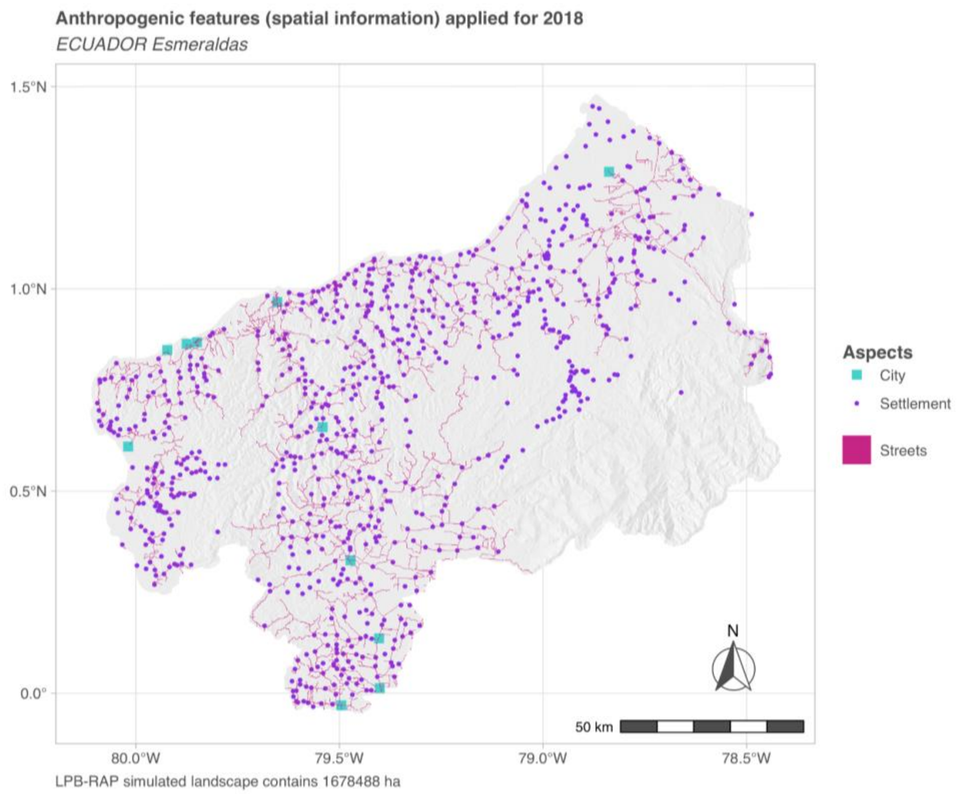

Figure B2 Here shown are the initial starting conditions in regard to anthropogenic features of built-up for the initial simulation year in both conservation scenarios. Cities (enhanced) are simulated with an impact distance of 50 km, settlements (enhanced) with 1.7 to a maximum of 10 km and streets with 5 km.

The features "cities, settlements, street" are applied in LBP altogether as built-up pixels in the correction step (see section 4.). The surface freshwater dataset (mainly depicting rivers, lakes and deltas) in contrast is not applied as land use, since it is a natural landscape component with unknown extents and simulated as static (larger waterbodies are captured by the Copernicus map already). The information on surface freshwater is an important information and serves in LPB as a distance factor to define the suitability of a particular LUT, e.g. pastures, for allocation.

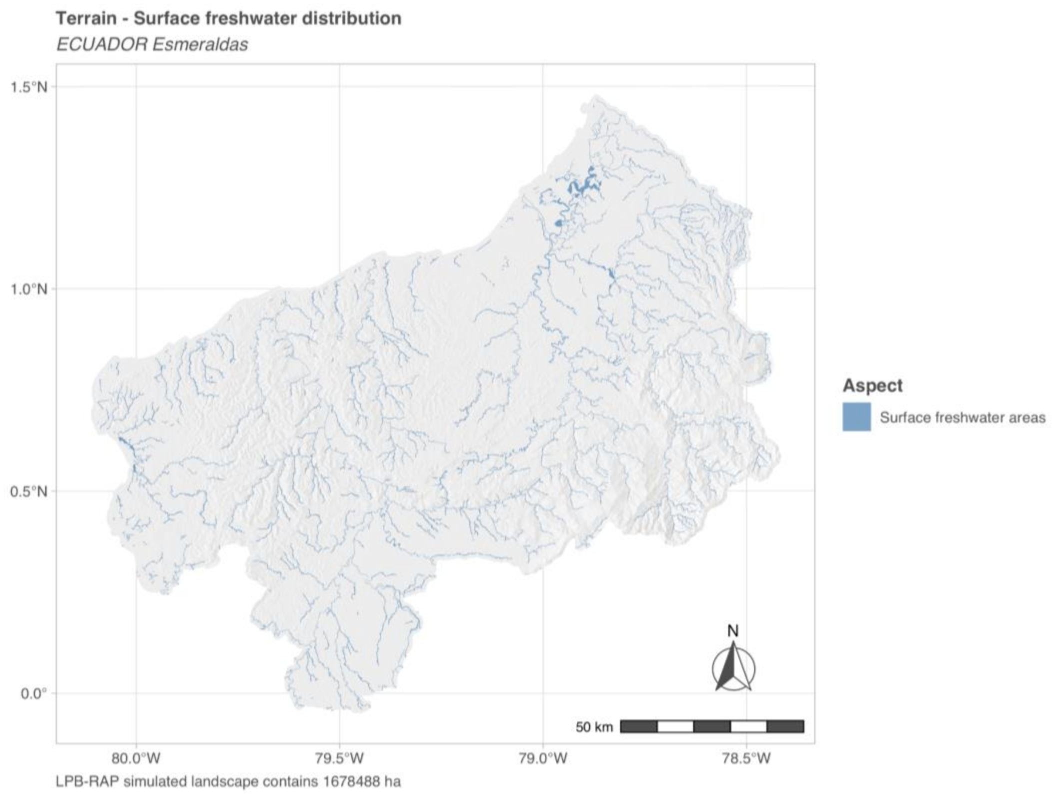

Figure B3 The OSM-based dataset of waterbodies complements the Copernicus merged water class information by information on conditions at the terrestrial surface level. It serves as a suitability factor for selected land use types.

2.3 Restricted areas (new)

For information on restricted areas, we combined available World Data Base on Protected Areas (WDPA) information [8] with the available national data on protected zones and community managed areas [9] information (conservation scenarios). We assume that these areas are restricted in the meaning of prohibited land use and logging which in reality is dependent on the local agreements and could be expressed in different degrees of forest use, management and protection.

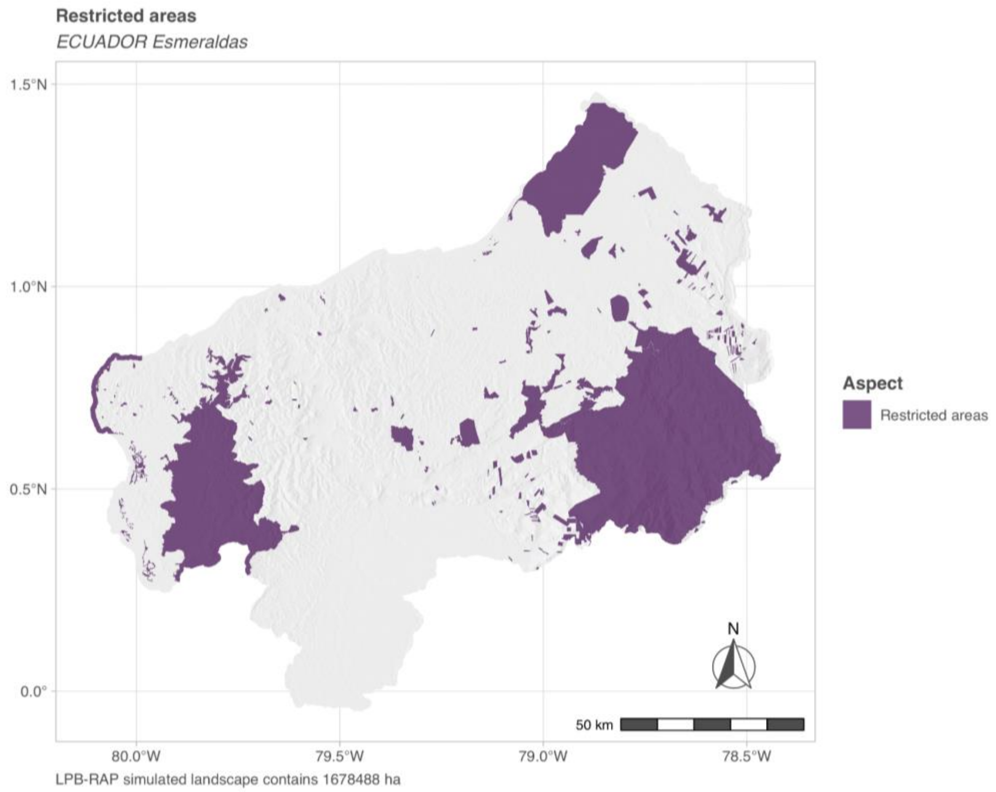

Figure B4 Location of restricted areas of Esmeraldas Province

2.4 Terrain

A digital elevation model (DEM) was derived from the 90 m SRTM dataset [10] which was resampled to a 1 ha pixel size using nearest neighbor and bilinear method. The DEM is further used for slope derivation within the model.

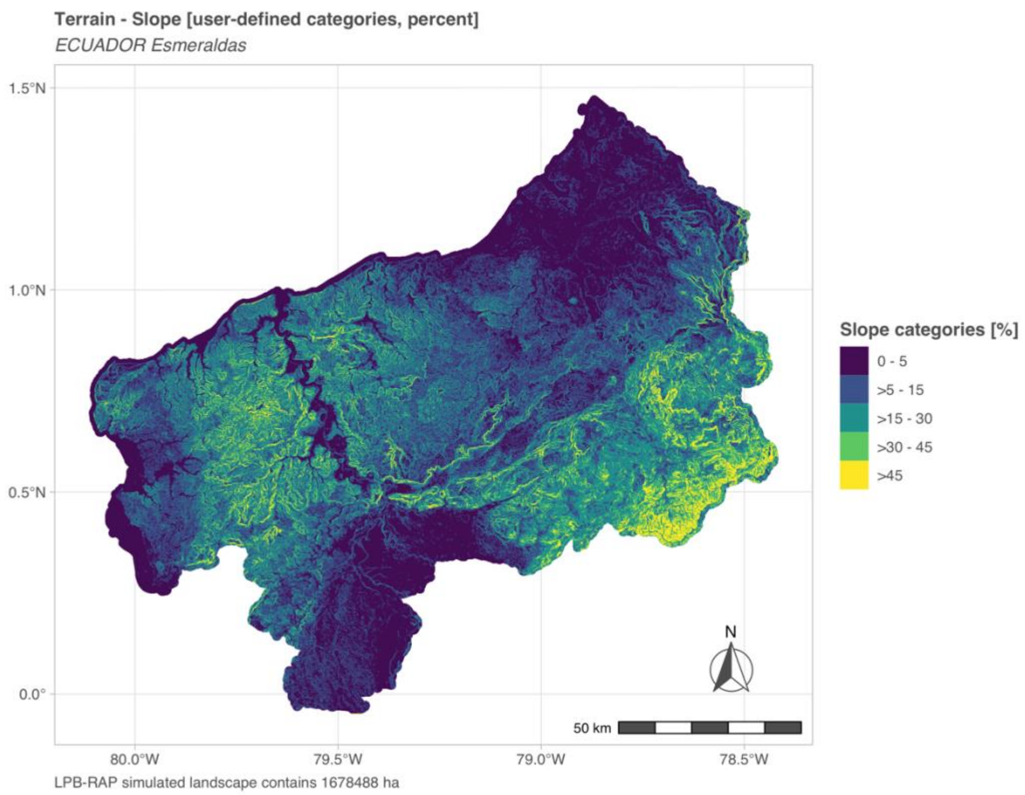

Figure B5 Slope levels (in percent) of Esmeraldas province

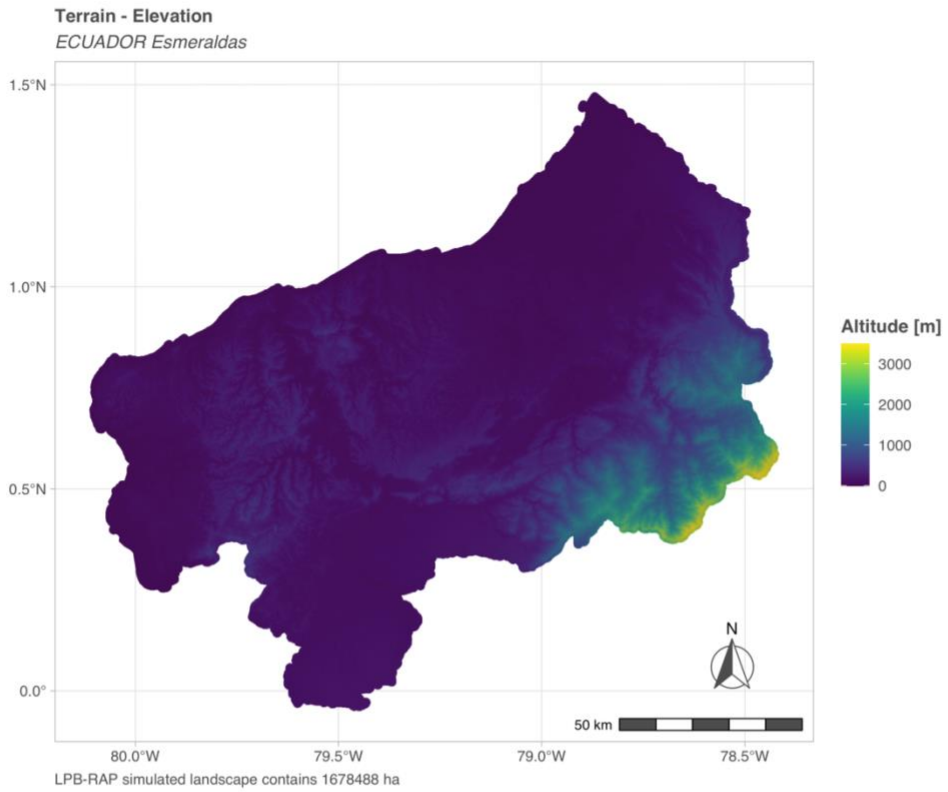

Figure B6 Altitude levels (in m) of Esmeraldas province

2.5 LULC map

Similar to the PLUC model, LPB requires only a singular initial land use land cover (LULC) map as input which depicts the regional LULC extent for the scenario simulations. For the case of Esmeraldas province, we used a multi-stage approach to derive the required LULC input map which we describe in the following section in more detail.

We rely on the Copernicus land cover map 2018 ([11]; henceforth referred to as "Copernicus") which has global coverage and hence is suitable for LPB applications in other regions and contexts. Copernicus was used as the basis for the initial input map due to several reasons: firstly, the LULC classification of Copernicus is based on time series data of consolidated maps with full year prior and pastor data; secondly, it uses the FAO Land Cover Classification System; thirdly, it provides an overall accuracy of 80 % with accuracy of unchanged pixels of up to 99.6 %; additionally, Copernicus is an ongoing mapping project that produces regular mapping information. Once such information becomes available, data could be also used for a comparison of modeled LULC maps compared to such a reference. The initial global Copernicus map consists of 23 discrete classes. For LBP modeling purposes, we reduced these classes and merged all forest classes into only one since we here follow another forest types separation. We further merged all water classes and moss, lichen and bare/sparse vegetation into a single class, too. Snow and ice do not occur in the LaForeT regions, hence, were omitted. For the Esmeraldas region, Copernicus reports almost full forest cover, which in the view of LPB depicts the gross forest extent prior to the following alteration and parametrization steps. Since we differentiate forest into disturbed and undisturbed areas, we initially classified the gross forest information of Copernicus as disturbed forest.

Unfortunately, the Copernicus map is incomplete (due to the faulty GSHHG [12] border dataset) for the coastline of Esmeraldas province. Hence, a total of 272.2 km^2 land area is missing compared to the reported administrative boundary reported by GADM. Hence, we patched this missing information with to a 100 m rasterized national [13] data and translated the LULC description of the MAE dataset as follows:

- forest plantation (n = 92 pixels) was assigned as LUT "disturbed forest", since it did not match with the TMF plantation data,
- settlement and infrastructure (n = 1,971+189 pixels) were reclassified to the LUT "built-up",
- areas without vegetation (n = 961 pixels) were assigned to the new combined LUT of "moss, lichen, bare, sparse vegetation",
- bushland (n = 157 pixels) was assigned to "shrubs",
- agricultural land (n = 11,828 pixels) was assigned to the pre-simulation LUT "cropland-annual" as coastal regions will be preferred for farming following the LPB approach and the given suitability information due to the prevailing marginal slopes. This is irrelevant since the agricultural extent is later overlayed with a randomized distribution of the three agricultural types, see below.
- native forest (n = 12,022 pixels) was assigned to the LUT "disturbed forest".

This step was necessary since the coastline is a rather well-developed part of Esmeraldas and hence we were required to fully represent the regional LULC extend. Since Copernicus is foreseen to solely rely on Sentinel 1 and 2 satellite data, this error will be corrected with the coming map releases. Because the affected area depicts only 1.66 % of the regional simulated total area, we deem this alternation justifiable in the regional modeling context of LPB.

This Copernicus+MAE based map is in a second processing step enriched with the basic (structural canopy status) "undisturbed forest" information from the “Tropical Moist Forest” dataset ([14]; henceforth called "TMF") differentiating the initial forest class into two forest types. We remain within the nomenclature of disturbed and undisturbed forest instead of primary and secondary forest, since the TMF dataset dates back to at maximum 1982 only and thus cannot be used to indicate if the undisturbed areas are truly primary forest extents or not. The undisturbed pixel information in this case was incorporated where the Copernicus+MAE map referred to a forest pixel. Additionally, we could differentiate plantations based on

TMF (95.14 % of pixels that were substituted with the TMF plantation information already belonged to the merged Copernicus forest class). The resulting hybrid map serves as the initial LULC input map upon which the simulations of LPB are initialized (correction step and subsequently dynamic simulation).
We then applied a further step to approximate the agriculture area extent and the more heterogenous smallholder land use pattern in the initial LULC map for simulation. Namely, we extracted the marked "agriculture" extent from the MAE 2018 map, eliminated TMF plantation pixels from it, and applied it including a random pattern simulation pattern of LUTs 02, 03 and 04 on the Copernicus+MAE+TMF hybrid map. The random pattern was constructed with the QGIS function GRASS "r.surf.random" and then pixels congruent to the agriculture map selected. Note that this approach does not allow to set maximum numbers of pixels per LUT, therefore the initial LULC input map requires later on correction in the model internal parametrization step. Both steps might get obsolete with further Copernicus map updates.

For an overview of case study specifics for the basic used LUTs see Table B4:

Table B4 LPB Land Use Types gives explanatory details of simulated (generic) land use types in regional modelling

land use type ID	land use type name [depicting land use at terrestrial surface level]	simulation type	first appearance in case of Esmeraldas	remarks (for Esmeraldas application)
**LUT01**	built-up	primary active LUT, final LUT	initial LULC map	Copernicus (and MAE) based; in the <= 100m resolution approach this LUT signifies the agglomeration of impacted terrestrial surface, either directly sealed or impacted by soil compactness etc.
**LUT02**	cropland-annual	primary active LUT	initial LULC map (remote sensing based and pre-classification)	Copernicus (and MAE) based. Depicts cropland with annual crops and short fallow periods.
**LUT03**	pasture	primary active LUT, purely coded	correction step	for Esmeraldas only computed. Depicts pasture without trees.
**LUT04**	agroforestry	primary active LUT, purely coded	correction step	for Esmeraldas only computed. Agroforestry depicts agroforestry systems starting with cropland-perennial up to agrosilvopastoral use.
**LUT05**	plantation	primary active LUT, semi-final LUT	initial LULC map	TMF based (likely no timber plantations). Here only an approximation of the used land management system.

**LUT06**	herbaceous vegetation	only active in succession or corrective allocation	initial LULC map	Copernicus (and MAE) based
**LUT07**	shrubs	only active in succession or corrective allocation	initial LULC map	Copernicus (and MAE) based
**LUT08**	disturbed forest	only active in succession or corrective allocation	initial LULC map	Copernicus (and MAE) based plus simulation of anthropogenic impact and succession.
**LUT09**	undisturbed forest	only active in succession or corrective allocation	initial LULC map	TMF based, p.r.n simulated
**LUT10**	moss, lichen, bare, sparse vegetation	passive, other ecosystem LUT	initial LULC map	Copernicus (and MAE) based
**LUT11**	herbaceous wetland	passive, other ecosystem LUT	initial LULC map	Copernicus (and MAE) based
**LUT12**	water	passive, other ecosystem LUT if user-defined	initial LULC map	Copernicus (and MAE) based
**LUT13**	no input	excluded from simulation	initial LULC map	Copernicus based
**LUT14**	cropland-annual -- abandoned	secondary active LUT, purely coded	simulation	for Esmeraldas only computed
**LUT15**	pasture -- abandoned	secondary active LUT, purely coded	simulation	for Esmeraldas only computed
**LUT16**	agroforestry -- abandoned	secondary active LUT, purely coded	simulation	for Esmeraldas only computed
**LUT17**	net forest -- deforested	(primary or secondary) active LUT, purely coded	simulation	for Esmeraldas only computed
**LUT18**	plantation -- harvested	secondary active LUT, purely coded	simulation	for Esmeraldas only computed

Note: LUT19 and LUT20 are not defined yet, but could be added, e.g., for depiction of regions which display static snow and ice or for applications which need to differentiate oceans and surface freshwater in the land use simulation.

For the LULCC_RAP module (see main body section 2.5.3), LPB calculates four to five additional LUTs (LUT21 to LUT24 resp. 25) which are based on the applied algorithms and which partially substitute pixels of the basic LUTs, which in turn depict the most probable landscape configuration under the assumption that restoration is not yet implemented as a landscape wide notable factor.

2.6 Initial net forest (new, based on national LULC map)

To make the connection to national forest policy goals, it is important to use national datasets of forest extents (net forest) in addition to the above described (relatively broad) Copernicus forest information. The map layer "initial net forest" is used for the dynamic simulations of LPB to indicate where pixels with forest site qualities at terrestrial surface level could be located in contrast to home gardens, parks etc.

Since such maps are also reliant on land cover data and do not always distinguish agroforestry systems, the dataset serves only as an initial information. Net forest in this simulation approach describes not only the possibly still intact forest landscape, but also future forest land use extents, that have the potential to return to a state of full biodiversity due to their immediate spatial relations to the initial net forest extent.

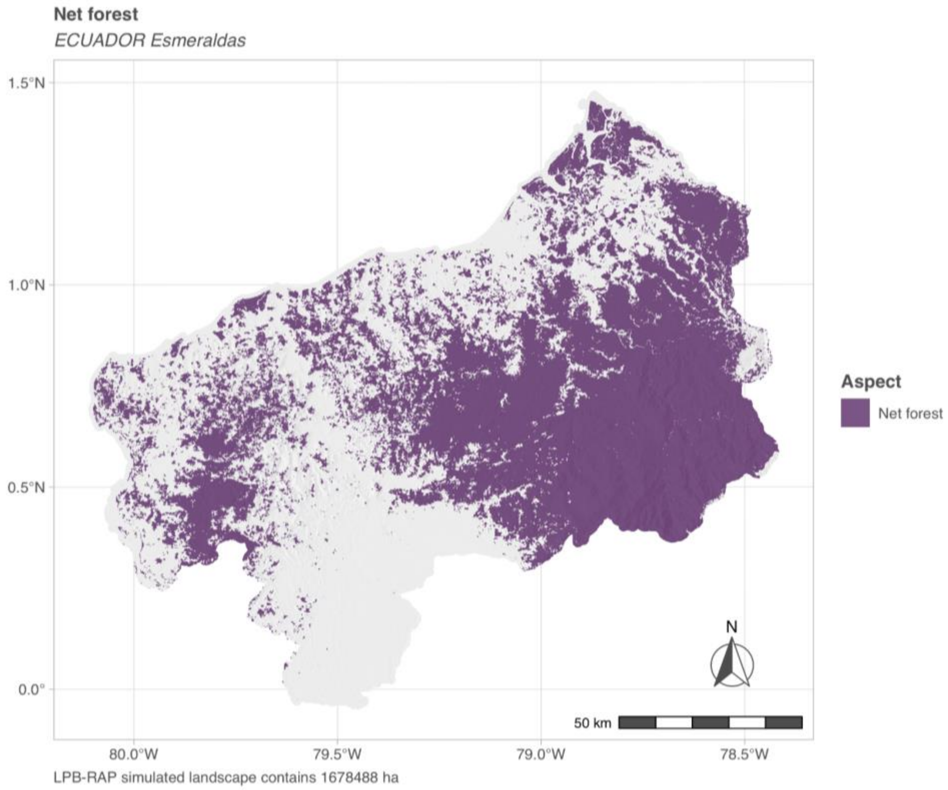

Figure B7 The figure shows net forest in the Esmeraldas region, meaning forest as defined by the national standard, in this case depicting tree cover > 30 %.

2.7 Baseline scenario determinant population (new/adapted)

We used „SSP2 total" of the provided datasets [41,42] to fit the narrative of persistent patterns and to account for the regional landscape configuration of the study area.

2.8 Baseline scenario climate data-based determinants (new)

For scenarios, the work of the Intergovernmental Panel on Climate Change (IPCC) Assessment Reports (AR) based on narratives and the outcomes of the Coupled Model Intercomparison Project (CMIP) provides guidance. IPCC AR5 (2013) featured CMIP5 model results while using the concept of „Representative Concentration Pathways" [27], short: RCP, scenarios. IPCC AR6 (2021/2022) featured CMIP6 model results while using „Shared Socioeconomic Pathways" [28,29], short: SSP, scenarios, providing complementary underlying economic and social arguments for the emission pathways as a range of plausible futures. CMIP6 models have thereby and due to the incorporation of new findings expanded functionality and resulted in slightly higher warming, but a subset of models is considered overly sensitive [30,31].

We followed here the SSP2 („Middle of the Road") scenario narrative for a population development scenario (as an approximation of persistent patterns) but in conjunction with the RCP 4.5 climate data, as a standard association, for climate-derived determinants; for the combined description, we use "SSP2-

4.5", but the reader must keep in mind, that this does not refer to the new calculations of CMIP6 climate data. However, the development to 4.5 W/m^2 by 2100 is here deemed a plausible minimum development [32,33].

Spatially downscaled high-resolution climate data (monthly temperature and precipitation) of 30 arcseconds (approximately 1000 m) are provided by the CHELSA project [34], covering all general circulation models involved in CMIP5 (n=38). We here rely on CMIP5 CHELSA v.1 RCP 4.5 based on MPI-ESM-mr calculations following suggestions by Sanderson et al. (2015). To acknowledge differences between climate models, additional investigations could be employed in future studies, simulating based on different climate model inputs.
Climate-derived data is given to LPB-RAP as thematic climate period projections based on a given climate reference period (here: 1979–2013 applied for the simulation time frame 2018–2020); for this study, we simulated future conditions based on four subsequent climate periods, namely 2021–2040, 2041–2060, 2061–2080 and 2081–2100. All calculations were made on the original 1000 m resolution, and the datasets posterior harmonized to the 100 m scale (see S1 File section 1.1 and S2 File section 2.8 for further information).

Climate data-based components are potential natural vegetation and woody aboveground biomass (AGB), see S1 File. Climate periods including inter- and extrapolation are used for the following three datasets, which are all based on the climate, here CHELSA [15] plus inter- and extrapolated climate periods (see S1 File), input datasets in combination with further secondary datasets. For the use case we apply the conditions of the climate reference period as still valid for the simulation years 2018 to 2020 followed by the four projected 20-years climate periods for the timeframe 2021 to 2100. These datasets are calculated on the original 1 km resolution and afterwards adapted to the 100 m model input scale with the original information still in 1 km resolution (no further downscaling applicable). Uncertainty information for each dataset is given in the description.

2.8.1 Potential natural vegetation

To simulate succession dynamics, discrete results based on probabilistic modeling and RAP, estimates of future potential natural vegetation are required (see S1 File). For further information on potential natural vegetation modeling see [16].

For the forest landscape succession simulation the maximum number of suitable pixels per climate period is relevant as a ceiling, these are shown in table B5 for the current study area:

Table B5 Potential forest area gives a short overview of climate period based potential forest area as a succession basis within LPB

Simulated period	applied period mean	Number of forest biome pixels (ha)	Percentage of total simulated landscape [1678488 ha]
**2018 – 2020**	**2010 (crp)**	1,497,169	89.20
**2021 – 2040**	**2030 (cp i)**	1,414,988	84.30
**2041 – 2060**	**2050 (cp)**	1,486,398	88.56
**2061 – 2080**	**2070 (cp)**	1,499,332	89.33

**2081 – 2100**	**2090 (cp e)**	1,303,031	77.63

The spatial distributions can be seen in figure B8.

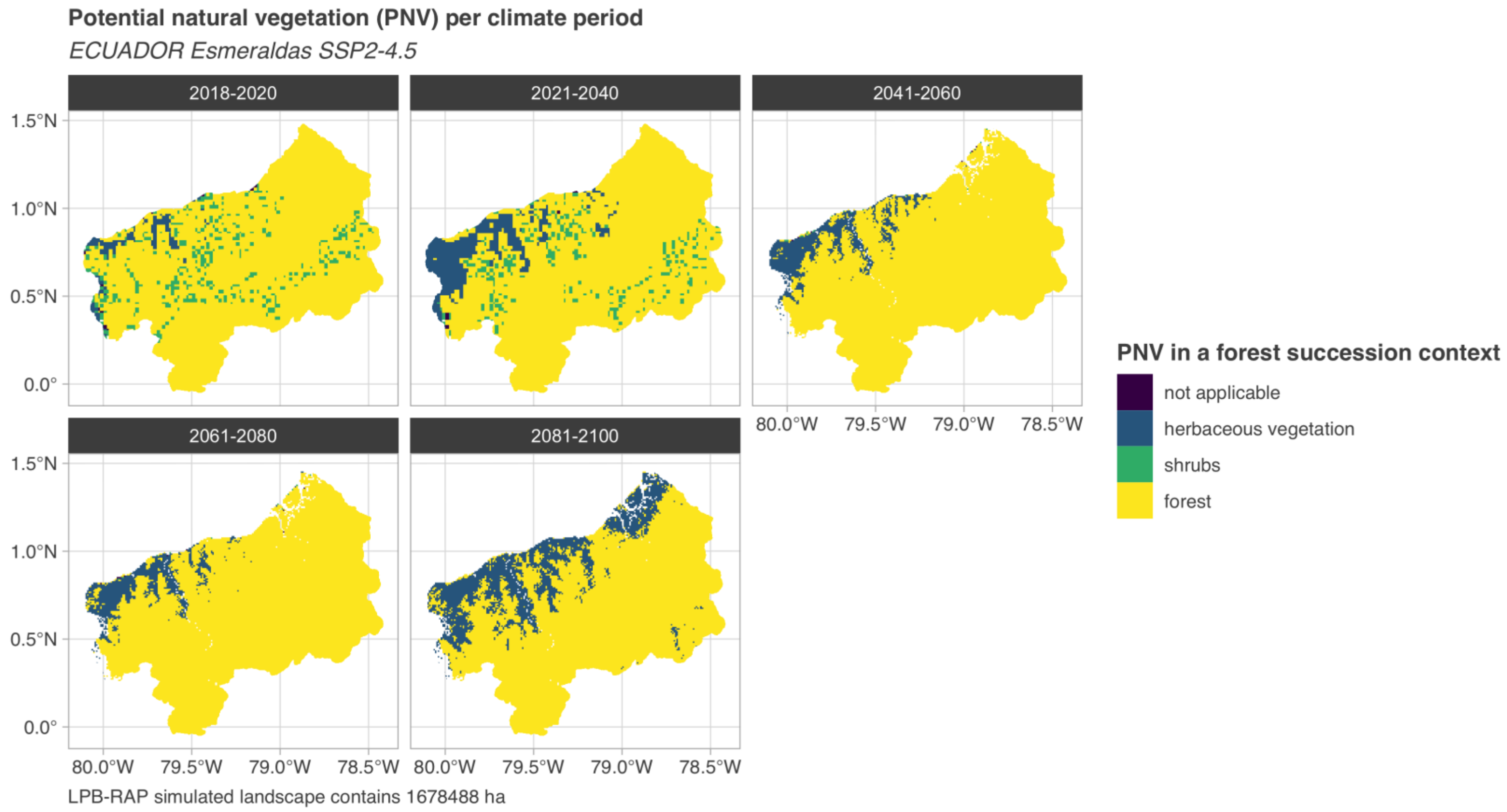

Figure B8 Potential natural vegetation distributions per climate period

2.8.2 Potential maximum undisturbed AGB

We estimated potential maximum AGB per climate period based on the available data to cap the simulation if needed to avoid an overestimation of AGB dynamics in case of the forest LUTs. As no data for primary forest AGB according to geo-climatic zones are yet available for Esmeraldas Province, we rely on the ESA biomass dataset version 3 for 2018 ([17], henceforth: "ESA AGB V3") to approximate potential maximum AGB initial input. This was combined with the TMF undisturbed forest dataset to which the following adjustment steps were applied:

- ESA AGB V3 pixels were selected if overlapping the TMF undisturbed forest information, indicating older-growth forest sites.
- Available information was resampled to a 1000 m resolution to adjust it to the climate data
- Multilevel-B-spline interpolation was applied to the regional tile extent
- Multiple regression with bioclimatic variables of the climate reference period was conducted (mean temperature of driest quarter, mean temperature of wettest quarter, precipitation of driest quarter, precipitation of wettest quarter) resulting in an adjusted multiple R^2 of 57.03.
- Projection with the regression coefficients for each future climate period was applied
- Pixels for the Esmeraldas were resampled to 100 m and for the simulation extent selected.

This correction may result in underestimates of individual cells of existing forest plots over the course of the simulation but describes the overall landscape AGB trajectory better than relying on a solely applied increment in a climate change context.

[Multiple Regression Analysis (Grid and Predictor Grids)] Parameters:
Grid System: 1000; 192x 172y; 598000x 9994000y
Dependent Variable: Regression
Predictors: 4 objects (Mean Temperature of Driest Quarter, Mean Temperature of Wettest Quarter, Precipitation of Driest Quarter, Precipitation of Wettest Quarter)
Regression: Regression
Residuals: Residuals
Details: Coefficients: Details: Coefficients
Details: Model: Details: Model
Details: Steps: Details: Steps
Resampling: B-Spline Interpolation
Include X Coordinate: false
Include Y Coordinate: false
Method: stepwise
Significance Level: 5
Cross Validation: none

Steps:

No.	R	R2	R2 adj	StdErr	F	P	F step	P step	Variable
1.	0.74	55.44	55.44	51.913	11757.374	0.000	11757.374	0.000	>> Precipitation of Wettest Quarter
2.	0.75	56.51	56.50	51.291	6137.961	0.000	231.606	0.000	>> Mean Temperature of Wettest Quarter
3.	0.75	56.89	56.88	51.066	4156.200	0.000	84.362	0.000	>> Precipitation of Driest Quarter
4.	0.76	57.04	57.03	50.980	3136.084	0.000	33.216	0.000	>> Mean Temperature of Driest Quarter

Correlation:

No.	R	R2	R2 adj	StdErr	t	Sig.	b	Variable
0.	-1.00	100.00	100.00	6.354	20.963	0.000000	133.207232	Regression
1.	0.40	15.99	15.95	0.003	42.398	0.000000	0.119337	Precipitation of Wettest Quarter
2.	-0.07	0.43	0.39	3.932	-6.377	0.000000	-25.078106	Mean Temperature of Wettest Quarter
3.	-0.11	1.20	1.16	0.004	-10.705	0.000000	-0.043728	Precipitation of Driest Quarter
4.	0.06	0.35	0.31	4.057	5.764	0.000001	23.385383	Mean Temperature of Driest Quarter

Formula: 133.207 + 0.119337 * X1 – 25.0781 * X2 – 0.0437275 * X3 + 23.3854 * X4

Residual standard error: 50.979604 (degrees of freedom: 9446)
Multiple R-squared: 57.044767 (adjusted: 57.026577)
F-statistic: 3136.083932 (4/9446 DF), p-value: 0

---

total execution time: 0 milliseconds (less than 1 millisecond)

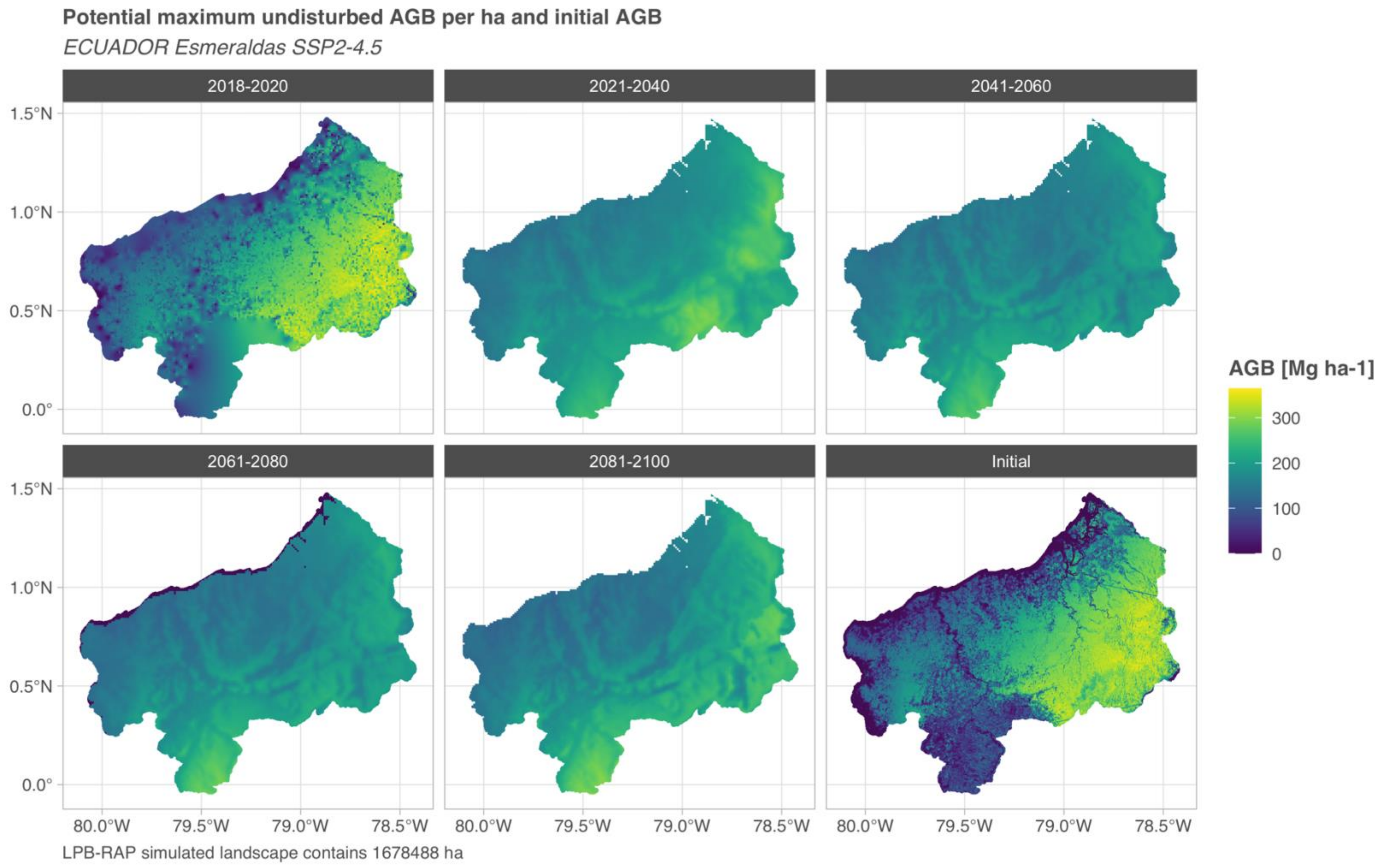

Figure B9 Potential maximum undisturbed AGB per ha. The Climate reference period results show the residuals of the applied undisturbed maximum AGB dataset, for future climate periods the regression coefficients were projected.

2.8.3 Potential forest types annual AGB increments

Spatially explicit increments were conceptualized as the primary approach and accordingly coded, since they would add more precision to the simulation. The approach then derives forest-type increments in the same manner as described above for the potential maximum undisturbed AGB which can subsequently be applied for the future climate periods if R^2 would be sufficient. For the Esmeraldas region, however, this could not be realized by relying on increments of ESA AGB V3 data 2017 to 2018 due to a reported quality flag 3 (improbable change). Hence, the approach has to be checked again on other regions if ESA AGB V3 data indicates reliable AGB gain.

2.9 Initial AGB map (new/adapted)

We rely on ESA AGB V3 2018 in 100 m resolution with biomass described in Mg ha^{-1} dry matter to define potential AGB per time step t which LPB requires prior the start of a simulation (figure B10).

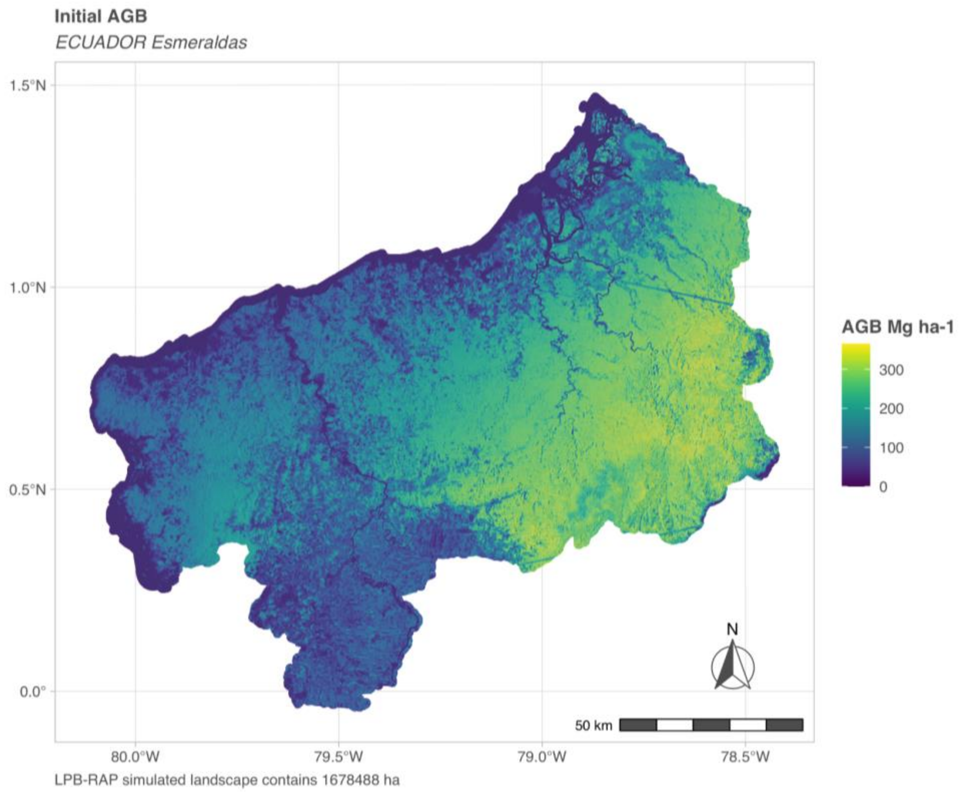

Figure B10 Initial AGB in original ha resolution as derived from the ESA AGB V3 dataset.

2.10 Excluded areas map (based on PLUC)

This dataset refers to the former PLUC "nogo" application, i.e., areas provided in this map are excluded from the simulation of active land use. No such areas due to in general applicable factors such as radiation etc. could be defined for the Esmeraldas province per se (resulting in a null mask). For the enforced conservation scenario this map is used congruent with the restricted areas map.

2.11 Initial plantation age map (new)

Prior to the start of a simulation, LPB requires information for each plantation pixel to define the rotation cycle in case plantations are part of a case study area. We retrieved this information from the TMF plantation datasets as an approximation. Since the TMF map provides such information until 2009 as the earliest, agglomerated date, we further incorporated a user choice to simulate the earliest date in the map stochastically by application of the set user-defined mean rotation period.

2.12 Other ecosystems map (new, RAP application)

In the case of Esmeraldas by default a null mask map, since we have no information on areas of small ecosystems deviating from forest resp. the available land cover types information. This application should be used for policy development and applied landscape planning, where actors most likely have according spatial information. The PCRaster data type is boolean.

2.13 Null mask

Like in PLUC, the user has to define a "null mask" in LPB for model simulation, meaning each pixel is set to 0 that simulates the total landscape extent within the "missing value" exterior. This dataset is used by the model for diverse applications and is necessary as a basis for, e.g., the calculation of maps that do not cover the whole simulated landscape in content but have to be aggregated again over time or samples. The current model uses this concept also for placeholders for potential additional user information, such as areas excluded from simulation in the weak conservation scenario or other ecosystems, which means the related map has to be provided under the file name but only includes zero-values for each pixel of the total simulated landscape unless the user can mask out corresponding areas.

3. SECONDARY PARAMETERS DATA, EXPERT OPINION AND SCENARIO ASSUMPTIONS

This chapter refers mainly to settings made in the model file Parameters.py, which is key to the long-term simulation of land use behavior and therefore contains besides empirical data necessarily expert opinion and scenario assumptions.

3.1 LPB (new or adapted)

Parameters that were altered or added in the new base model compared to PLUC:

3.1.1 Allocation order (adapted)

Like in PLUC, in LPB the allocation order can serve either as a depiction of the evaluated or assumed bottom-up land use or as the basis of different scenario simulations.
For the incorporation of the hectare scale, adaptation to the regional LUTs and long-term simulation, the original LUT allocation order of PLUC was extended to include built-up and plantation in addition to simulated agricultural LUTs described above. The default allocation order follows the default PLUC model [18,19] configuration as well as the assumption of chronological land use order and spatial arrangement in smallholder-dominated landscapes, with LUT01 = built-up (housing and logistics), food-based LUTs ,i.e.: LUT02 = cropland-annual, LUT03 = pasture and LUT04 = agroforestry as supported by the LaForeT survey data evaluated land use; and LUT05 = plantation depicting a semi-final, meaning it cannot be changed until the status changes to harvested, land use type of economic interest. For this study, only the default setting was used.

3.1.2 Distances describing anthropogenic impact on and use of forest (new)

Parameter Anthropogenic impact distance:
In the presented study, this parameter is only used for demonstration purposes that could eventually serve as means for landscape planning and was set to 2000 m. It serves as an approximation for a hypothetical impacted species using forest habitat and displaying avoidance of streets, cities and settlements besides forest fringes. For the applied use case the user will have to choose a source of impact with the maximum distance (e.g., pollutant range of chemicals transported via air or soil, impact of anthropogenic use frequency or known avoidance patterns in regard to anthropogenic features in forest landscapes) for the species of interest. The simulated value can therefore differ widely from a few meters up to several kilometers (which will be displayed in raster size). For the current study, no information for a special species of interest in regard to landscape planning or policy development was available.

Parameter Maximum distance for local wood extraction:
This parameter is only employed if the user wants to simulate forest degradation and regeneration stages and the according RAP-LUT25. It provides the basis for the simulated AGB demand extraction in local forest around settlements. In an weak conservation or enforced conservation scenario restricted areas are first excluded. Please note, that this parameter is describing most likely a greater distance than the settlement impact distance based on walking distances, since in wood extraction often animal or motorized transport is included. For the study at hand, we defined the local wood extraction distance with a value of 5000 m based on FAO, 2022, data [20].

3.1.3 Demand in input biomass in Mg (adapted)

Formerly described in PLUC in m^3 we transformed this demand to be depicted in Mg to enable a simulation based on the ESA AGB V3 input. Hence, metric wood demand had to be calculated from an external source. For the LaForeT domains we identified three potential components: subsistence timber, fuelwood

and charcoal input biomass demand. Subsistence timber was partially featured in the LaForeT survey data, but available information could not be converted to metric values due to qualitative notation of information, e.g., harvest of one tree per year.
For the case of fuelwood, we rely on UN data for fuelwood consumption in 2018 by households in m^3 [21]. For the dynamic application see section 3.1.12. With the provided UN datasets of population for 2018 [22], we then calculated the demand in m^3 per person for the year 2018.
Charcoal production is not a factor in the Esmeraldas region and hence was not further considered here [1].
To translate the demand in input biomass in the unit of m^3 for the model biomass calculation, all values had to be converted to Mg. Hence, we used LaForeT forest inventory information and calculated from all 2,413 individual apprehended trees (which refers to 230 species) the weighted mean of species wood densities as an approximation, which in case of Esmeraldas refers to 0.5.
The three subsistence demands for timber, fuelwood and charcoal are then summed up and calculated dynamically for each year based on population development in a static approach, for the dynamic application see section 3.1.12. For the Esmeraldas region, Table B6 summarizes the related LPB model settings:

Table B6 Input biomass demand shows the LPB recognized input compartments for simulation of demand per person

input biomass subsistence demand type	value in Mg a^{-1} per person	remarks
**timber**	0	no subsistence demand for timber in Mg could be derived from LaForeT or secondary sources
**fuelwood**	0.025898	-
**charcoal**	0	not a factor in the region, placeholder for e.g. Zambia simulation

3.1.4 Annual AGB increment ranges (new)

Estimates of potential annual AGB increments of forest types are required for the simulation of AGB dynamics in forest land use types. In absence of applicable spatially explicit data (see section 2.8.3 before), value ranges for stochastic simulation have to be applied for each pixel simulated on a uniform distribution per sample and per time step. In this case we rely on literature information covering the global scale and extracted information of South America and applicable area-based information. For an overview see table B7:

Table B7 AGB increment ranges

LUT	AGB increment range in Mg ha^{-1} a^{-1}	source	Further remarks
**disturbed forest**	0.4 to 5.9	[23]	Since the term disturbed is used in a qualitative fashion in the model cells can be disturbed but still display the undisturbed maximum AGB potential.
**undisturbed forest**			In this particular case undisturbed forest sites date back until latest 1982, but still cannot be distinguished in old-

			growth forest and older secondary forest.
**agroforestry**	0.1 to 12.5 [derived from the provided carbon stocks values and recalculated to biomass with the conversion factor]	[24]	The here used values have reference to agricultural agroforestry systems evaluated by an area approach, so this excludes the types hedgerow, fallow (depicted in succession stages until disturbed forest) and parkland (depicted in disturbed forest)
**plantation**	0 to 0	-	Since the Esmeraldas depict oil palm plantations in these extents and oil palm cannot be considered as a forest type, here set to 0

If spatially explicit data cannot be used, the coded stochastic simulation for these increments per time step in provided broad ranges should ensure an overall plausible average depicted in the landscape total AGB results per forest type per time step and provide therefore a sound basis for the derivation of potential carbon content. A climate change signal cannot be simulated with this approach.

3.1.5 IPCC biomass to carbon conversion factor (new)

For a generic approach (especially for countries and regions where forest inventory information is missing) we use the IPCC default conversion factor of 0.5 [25] for the conversion of dry matter forest AGB to carbon. It can be user adapted to specific IPCC tier-related values if applicable to the landscape. Here the application agglomerates different forest types (potentially natural -mainly in-land, but in the case of Esmeraldas also partially mangrove systems -, as well as under management) where additionally no specific tree species can be differentiated.
For conversion from carbon to CO_2 the stoichiometric conversion factor of (44/12) = 3.67 can be used (not featured by LPB).

3.1.6 PLUC core residual parameters

The provided parameters depict the basic model algorithm of the PLUC model and were adapted to the LPB approach to allow a larger number of explicit LUTs and partially diverging suitability factors. The basic proportionalities of reported default ranges were left from PLUC where possible due to the nature of bottom-up land use simulations. Assumptions for long-term depictions of land use were additionally incorporated in case of LPB.

3.1.6.1 Related land use types (adapted)

This parameter determines neighborhood operations in the cellular automaton approach. Adapted from the PLUC approach to the ha LPB resolution, LUT built-up is only related to built-up and LUT plantation is only related to plantations. In contrast, all three agricultural LUTs are related to each other (cropland-annual, pasture, agroforestry). This configuration is enabled via the initial pixels provided by secondary spatial data, the parameterization correction step application for anthropogenic features and the dynamic modeling rules, especially by agglomerated 1 ha built-up pixels for new settlements.

3.1.6.2 Suitability factors, their weights and parameters (adapted)

These settings still determine the spatial distribution of allocated demand. From the suitability factors used for PLUC, we diverted from or kept the original values in the following manner:

1) Number of neighbors in the same class was kept to a 3x3 window and therefore adapted to 300 m,
2) to 4) Distances to streets, freshwater and cities was adopted from PLUC,
5) Distance to settlements was added and regionally adapted based on LaForeT data of walking distances,
6) Yield was eliminated, as obsolete in the footprint approach (still available for the demand/yield approach),
7) Population density was kept without further changes,
8) Cattle density was eliminated due to the footprint approach (still available for the demand/yield approach),
9) Distance to forest edge was adapted to the newly added level of net forest,
10) Current land use was expanded and adapted to the new range of LUTs.

The discrete values were either derived from empirical primary data, adopted from the original model or adjusted from PLUC to the new approach using expert opinion.

Suitability factors (SF) in LPB:
SF1 = number of neighbors in same class
SF2 = distance to streets
SF3 = distance to freshwater
SF4 = distance to cities
SF5 = distance to settlements (NEW)
SF6 = population density
SF7 = distance to net forest edge (ADAPTED)
SF8 = current land use

Active LUTs, their SFs, their weights and underlying (long-term) assumptions:
Table B8 provides an overview of the default settings used for simulation of non-mosaic LUTs in a long-term simulation. Weights have to amount to 1 (100 %) for each LUT. Set by expert opinion based on the PLUC settings and the provided assumptions.

Table B8 Land Use Types Suitability Factors Weights Assumptions lists the underlying assumptions made for this simulation component

active LUT	SF1 neighbors	SF2 distance streets	SF3 distance fresh-water	SF4 distance cities	SF5 distance settle-ments (new)	SF6 popula-tion density	SF7 distance net forest edge (adapted)	SF8 current land use
**1 built-up (new as a dynamic LUT)**	X	X		X	X	X		X
	0.15	0.1		0.2	0.2	0.2		0.15
	new built-up will primarily occur in the neighborhood of	for housing a basic relationship to streets is given	irrelevant for built-up	cities will likely gain area	settlements will likely gain area	built-up is related to population growth needs	no relationship to forest (could be either within or outside)	built-up will occur on almost any land use type if needed

	existing built-up (dynamic covered by dynamic settlements, dynamic street network not yet implemented)							
**2 cropland-annual (explicit)**	X	X	X	X	X	X		X
	0.2	0.1	0.1	0.1	0.2	0.2		0.1
	fields will expand in size if possible (location factors)	for land management and harvest streets might be relevant	irrigation maybe a factor	cities population for demand maybe an economic factor	smallholder will farm land primarily near to their housing location	farmland is related to population density	no relationship to forest (could be either within or outside)	cropland-annual will occur where needed and possible
**3 pasture (explicit)**	X	X	X	X	X	X		X
	0.2	0.1	0.1	0.1	0.2	0.2		0.1
	pastures will expand in size if possible (location factors)	for land management and tending to livestock including transport streets might be relevant	providing drinking water and irrigation maybe a factor	cities population for demand maybe an economic factor	smallholder will use pastures primarily near to their housing location	pasture is related to population density	no relationship to forest (could be either within or outside)	pasture will occur where needed and possible
**4 agro-forestry (explicit, new)**		X			X		(X)	X
		0.25			0.5		(0)	0.25
	agroforestry plots are independent from neighbors (could	for land management and harvest streets might be relevant	in groundwater and rainfed areas irrigation by surface	cities are not a factor for agroforestry	smallholders associated with settlements need agroforest	plots are not related to population density	here the relationship is unclear, agroforestry could be either	current land use is a factor for decision for transformation to

	occur singularly or in bulk)		freshwater is not a factor		ry space		depicted within or outside net forest - depends on the national map	new plots or keeping the land management system
**5 plantation (new)**	X	X				X		X
	0.5	0.05				0.25		0.2
	plantation plots will occur in bulk due to economic interests	for land management and harvest streets might be relevant	in groundwater and rainfed areas irrigation by surface freshwater is not a factor	cities are not a factor for plantation	settlements are not a factor for plantations	plantations will occur where population density is lower	no relationship to forest (could be either within or outside)	current land use is a factor for decision for transformation to new plots or keeping the land management system

SF parameters (P):
The used parameters for suitability factors (overview listed below) are unchanged from PLUC (for application per land use type see further down):

- SF1 neighbors = P1: window length
- SF2 distance streets = P1: direction; P2: maximum distance effect; P3: friction, P4: relation type
- SF3 distance water = P1: direction; P2: maximum distance effect; P3: friction, P4: relation type
- SF4 distance cities = P1: direction; P2: maximum distance effect; P3: friction, P4: relation type
- SF5 distance settlements = P1: direction; P2: maximum distance effect; P3: friction, P4: relation type
- SF6 population density = P1: direction
- SF7 distance to net forest edge = P1: direction
- SF8 current land use = P1: suitability of current land use to become this land use type (range from 0 to 1; 0 = zero suitability; 1 = maximum suitability)

SF parameters application for the five active LUTs (for SF8 settings and the rationale behind it please see below the explanation provided for the overview of the land use transition matrix by suitability):

LUT01 = built-up
Suitability factors (SF) = 1, 2, 4, 5, 6, 8
Parameters (P):

- SF1 window length for a 3x3 window with a cell length of 100 m = 300 m
- SF2 distance streets = P1: negative direction; P2: 5000 m maximum distance effect; P3: unknown or not exponential friction, P4: inversely proportional relation type

- SF4 distance cities = P1: negative direction; P2: 50000 m maximum distance effect; P3: unknown or not exponential friction, P4: inversely proportional relation type
- SF5 distance settlements = P1: negative direction; P2: 10000 m maximum distance effect; P3: unknown or not exponential friction, P4: inversely proportional relation type
- SF6 population density = P1: positive direction
- SF8 current land use = P1: suitability of current land use to become this land use type: see table 9

Made assumptions: Due to assumed potential settlement growth we defined a distance of 10 km for the maximum distance of effect for SF5, diverging from the current LaForeT distances.

LUT02 = cropland-annual
Suitability factors (SF) = 1, 2, 3, 4, 5, 6, 8
Parameters (P):

- SF1 window length for a 3x3 window with a cell length of 100 m = 300
- SF2 distance streets = P1: negative direction; P2: 5000 m maximum distance effect; P3: unknown or not exponential friction, P4: inversely proportional relation type
- SF3 distance water = P1: negative direction; P2: 10000 m maximum distance effect; P3: unknown or not exponential friction, P4: inversely proportional relation type
- SF4 distance cities = P1: negative direction; P2: 50000 m maximum distance effect; P3: unknown or not exponential friction, P4: inversely proportional relation type
- SF5 distance settlements = P1: negative direction; P2: LAFORET maximum distance effect 2200m; P3: unknown or not exponential friction, P4: inversely proportional relation type
- SF6 population density = P1: positive direction
- SF8 current land use = P1: suitability of current land use to become this land use type: see table 9

LUT03 = pasture
Suitability factors (SF) = 1, 2, 3, 4, 5, 6, 8
Parameters (P):

- SF1 window length for a 3x3 window with a cell length of 100 m = 300 m
- SF2 distance streets = P1: negative direction; P2: 5000 m maximum distance effect; P3: unknown or not exponential friction, P4: inversely proportional relation type
- SF3 distance water = P1: negative direction; P2: 10000 m maximum distance effect; P3: unknown or not exponential friction, P4: inversely proportional relation type
- SF4 distance cities = P1: negative direction; P2: 50000 m maximum distance effect; P3: unknown or not exponential friction, P4: inversely proportional relation type
- SF5 distance settlements = P1: negative direction; P2: LAFORET maximum distance effect 3600 m ; P3: unknown or not exponential friction, P4: inversely proportional relation type
- SF6 population density = P1: positive direction
- SF8 current land use = P1: suitability of current land use to become this land use type: see table 9

LUT04 = agroforestry
Suitability factors (SF) = 2, 5, 7, 8
Parameters (P):

- SF2 distance streets = P1: negative direction; P2: 5000 m maximum distance effect; P3: unknown or not exponential friction, P4: inversely proportional relation type
- SF5 distance settlements = P1: negative direction; P2: LAFORET maximum distance effect 4850 m; P3: unknown or not exponential friction, P4: inversely proportional relation type
- SF7 distance to net forest edge = P1: direction: 0

- SF8 current land use = P1: suitability of current land use to become this land use type: see table 9

Made assumptions: For agroforestry depicted in the Esmeraldas forest landscape we cannot assume a positive or negative direction from or into net forest, i.e. the depiction of agroforestry only within or outside net forest extents since the original dataset does not single out the class agroforestry. Therefore it could be depicted within the class “native forest” or fall into the Copernicus gross forest classes (this parameter should be set to negative if the national map handles agroforestry as a single land use class and positive if net forest is congruent with gross forest).

LUT05 = plantation
Suitability factors (SF) = 1, 2, 6, 8
Parameters (P):

- SF1 window length for a 3x3 window with a cell length of 100m = 300 m
- SF2 distance streets = P1: negative direction; P2: 5000 m maximum distance effect; P3: unknown or not exponential friction, P4: inversely proportional relation type
- SF6 population density = P1: negative direction
- SF8 current land use = P1: suitability of current land use to become this land use type: see table 9

SF8 application suitability of current land use to become the active land use type for long-term simulation: Overview potential land use transition matrix by SF8

In table B9 an overview of the expert opinion applied SF8 default settings is given as a land use transition matrix: 0 indicates that this source LUT has no suitability to become the currently active LUT in allocation (for chronological order see 3.1.1), 1 that it has maximum suitability to stay the current LUT (set to simulate pattern stability over time). Static, final and semi-final LUTs are accordingly unavailable to change within this suitability factor. We set this assumption for built-up, which is a final land use type, and also plantations, which are a semi-final land use type, but also for food-based types to be no allocation cell for plantations (this could be again subject to different scenario simulations). Other active LUTs, abandoned and deforested types can theoretically become the current active LUT (0.5) but are set to have higher suitability to allocate the prior here active demand type again (0.75). The same suitability assumption is made for more easily accessible LUTs of ‘herbaceous vegetation’ and ‘shrubs’ (0.75). Forest types have lower suitability (0.4) due to implied friction (necessary logging to make land usable or arable). The lowest suitability (0.3) is assigned to the land use types ‘moss, lichen, bare, sparse vegetation’ and ‘herbaceous wetland’ due to assumed more intense efforts to make land usable or arable (enduring fertilization, drainage etc.) without the side effect of available wood, making them least appealing.

Table B9 Transition matrix gives a visual overview over the setting for Suitability factor 8. Colors are just for orientation.

active LUT / source LUT	1	2	3	4	5	6	7	8	9	10	11	12	13	14	15	16	17	18
**1**	1	0.5	0.5	0.5	0	0.75	0.75	0.4	0.4	0.3	0.3	0	0	0.5	0.5	0.5	0.5	0.5
**2**	0	1	0.5	0.5	0	0.75	0.75	0.4	0.4	0.3	0.3	0	0	0.75	0.5	0.5	0.5	0.5
**3**	0	0.5	1	0.5	0	0.75	0.75	0.4	0.4	0.3	0.3	0	0	0.5	0.75	0.5	0.5	0.5
**4**	0	0.5	0.5	1	0	0.75	0.75	0.4	0.4	0.3	0.3	0	0	0.5	0.5	0.75	0.5	0.5
**5**	0	0	0	0	1	0.75	0.75	0.4	0.4	0.3	0.3	0	0	0.5	0.5	0.5	0.5	1

Note that SF8 is only one suitability factor contributing to the accumulated total suitability map.

3.1.6.3 Land use types on which no allocation occurs (adapted and new)

Following model user definition, the provided LUTs are handled as immutable in the dynamic simulation of active LUTs, i.e. pixels of these static or dynamic LUTs are interpreted as (semi-)final and will not be changed in a sample during the Monte Carlo simulation:

- static LUTs (adapted): LUT12 = water and LUT13 = no input (only anthropogenic features in the correction step can change these pixels prior to the Monte Carlo simulation)
- dynamic LUTs (new): LUT01 = built-up (final) and LUT05 = plantation (semi-final, only harvested plantation (LUT18) plots can be subject to change). We added LUT4 = agroforestry based on the assumption, that cultivated plots are supposed to be yielded long-term.

3.1.7 Terrain restrictions parameters (adapted)

To describe the anthropogenic influence/impacted landscape under long-term changing conditions, we defined the categories of favorable terrain (level to moderate slopes), difficult terrain (strong to very strong slopes) and inaccessible terrain (extreme to very steep slopes) in regard to the active LUT. This concept is based on slope constraints and inherent implications for land use and management. These categories can be defined by the user based on survey data or be adjusted for new scenario simulations.

Slope restrictions of difficult terrain for LUT01 = built-up are based on the observation that restriction in reality is rather limited for construction projects for streets or housing. Difficult terrain, i.e., terrain that displays initial constraints for use, in the agricultural or cultivation context (LUT02 to LUT05) describes currently rather unused sloping land in the overall regional landscape setting but could be become relevant for future land uses in the long-term simulation due to population pressure, advancing available technological progress and adapted land management, such as terrace culture. This refers to all agricultural LUTs and also plantations. The maximum value for favorable terrain in regard to agriculture was adopted from PLUC, all other values presented here are scenario assumptions based on commonly used differentiations (for comparison see e.g., [26]) in accordance with a moderate worst-case scenario long-term application.
The use of difficult terrain is a function of demand over time and is implemented in LPB by: (1) the allocation order per time step t, (2) the allocation based on the policy scenario settings of land use and available areas, and (3) population development and its smallholder share with the set area demand. For the case study region of Esmeraldas, we used the provided ranges of slope (%) shown in table B10.

Table B10 Terrain shows the assumed terrain restrictions by slope in the three applicable modeling classes

LUT	favorable terrain [1st allocation of demand]	difficult terrain [2nd allocation of demand]	inaccessible terrain [no simulation of active LUTs]
**LUT01 = built-up**	0 to < 0.45	0.45 to 0.90	> 0.90
**LUT02 = cropland-annual**	0 to <0.17	0.17 to 0.45	> 0.45
**LUT03 = pasture**	0 to <0.17	0.17 to 0.45	> 0.45
**LUT04 = agroforestry**	0 to <0.17	0.17 to 0.45	> 0.45
**LUT05 = plantation**	0 to <0.17	0.17 to 0.45	> 0.45

3.1.8 Maximum slope for deforestation value (new)

This value can be adjusted regionally, the default is set to 0.45 = 45 % to limit the deforestation in the simulation to on average at maximum accessible slopes [26].

3.1.9 Mean plantation rotation period end (new)

We set a value of 25 years as a global approximation for the simulation of oil palm plantation land management patterns based on the TMF dataset. This value can be adjusted by the model user according to the verified or presumed plantation type depicted.

3.1.10 Succession time frames (new)

LPB requires information in user-defined column tables to simulate succession based on the provided potential natural vegetation datasets. This must follow the internal model logic of LPB that the first succession step can only start with a succession age of two years, i.e., in the next time step and the approximated succession stages in years. Succession is primarily applied to pixels of a deforested or abandoned LUT that are not converted to other anthropogenic LUTs during a simulation. For the simulation starting conditions, LPB refers to remaining pixels of the LUTs herbaceous vegetation, shrubs and disturbed forest, respectively.
In the default setting, LPB relies on succession values provided in table B11 for maximum succession to an undisturbed forest pixel (each biome type requires a singular user-defined column table input).

Table B11 Succession age gives a brief overview over the applied succession modelling for the three applied biome types herbaceous vegetation, shrubs and forest in LPB.

Simulated succession to LUT	after years (succession age)	featured in succession to LUT per biome pixel
**herbaceous vegetation**	2 years	herbaceous vegetation (grassland biome pixels)<br>shrubs (bushland biome pixels)<br>forest (forest biome pixels)
**shrubs**	2+3 = 5 years	shrubs<br>forest
**disturbed forest**	2+3+5 = 10 years	forest
**undisturbed forest**	100 years of disturbed forest pixel status without further anthropogenic impact	forest

Abandoned agroforestry is here depicted as either developing to a disturbed forest adjunct to forest biome pixels or declining again to shrubs or herbaceous vegetation on the adjunct biome pixels during a period of 5 years.

3.1.11 mplc module corrective allocation input (new)

To simulate deterministic demand 1:1 the model needs to apply a corrective allocation on probabilistic results aggregated by the highest probability per pixel (factual mplc). The model firstly corrects areas that are overshooting the discrete demand of the agricultural LUTs to the corresponding abandoned areas; secondly, then the abandoned LUTs excess area is corrected back to biome dependent land cover type by a user-defined column table. LUT01 = built-up is simulated with the maximum demand of all samples per time step and only until the population peak, which after the peak demand is simulated, since structures still exist. See table B12 for further information on the applied parameter setting for Esmeraldas Province.

Table B12 Correction depicts the applied correction LUTs within the corrective allocation in the LULCC_mplc module.

biome pixel	simulated LUT
**0 = undefined (singular remaining pixels at the coastline or rivers due to the 1 km resolution of the climate data, but p.r.n. also other vegetation types)**	herbaceous vegetation (approximation of land cover type; AGB is not calculated for these types)
**1 = grassland**	herbaceous vegetation
**2 = bushland**	shrubs
**3 = forest**	disturbed forest

3.1.12 External time series of active land use demands calibrated to baseline scenario assumptions (adapted)

For this case study, we simulated with the external footprint approach (i.e., an external time series of demands, where agriculture is applied based on a dynamic footprint for each time step based on initial primary data and the development of all active land use types is calibrated to scenario assumptions, here SSP2). Scenario assumptions regarding projections are applied for the time steps t2 (here: 2019) to n (here: 2100).

The superordinate global SSP2 narrative regarding land use can be summarized as depicted in Table B13 SSP2 assumptions - row "global", extracted from Popp et al., 2017, Table 1 [27]. Provided below are assumptions concerning the regional subnational simulation.

Table B13 SSP2 assumptions

SSP2 assumptions for projection	Land-use change regulation	Land productivity growth	Environmental Impact of food consumption	International Trade	Globalization	Land-based mitigation policies
**global**	Medium regulation; slow decline in the rate of deforestation	Medium pace of technological change	Material-intensive consumption, medium meat consumption	Moderate	Semi-open globalized economy	Delayed international cooperation for climate change mitigation. Partial participation of the land use sector

**Subnational regional study area**	Displayed all three policy enforcement scenarios regarding the protection of restricted areas within assumed continuing bottom-up land use; Deforestation due to fuelwood and oil palm plantation area demand declines, the commercial timber demands are unknown and therefore not part of the simulation.	Regional technological advancements are primarily expected in technologies to make steeper sloping terrain arable. For productivity growth we assume for this region a delayed availability as well as affordability and it is thereby not simulated.	Moderate increase in kcal intake per capita per day until 3000 kcal at 2060 – after which we projected this value to be constant; Moderate increase in meat consumption projected for the region which increases pasture area.	Unchanged. As no descriptive commercial demands for the regional extent are available, we simulate that current conditions are passed on under the assumption, that primarily smallholder demands shape the landscape.	Unchanged. Assumed is potentially increasing pressure to allocate demands (agriculture, wood, built up) in the landscape regionally caused by declining opportunities to expand.	Unchanged. Assumed is no further adaptation in land management patterns as "cropland-annual" is already only presented with a low share of cultivation area, compared to "agroforestry". For pasture we assume stable conditions as systemic transformation to agroforestry systems would require subsidies and knowledge transfer.

The subnational regional conditions in a landscape may diverge from the overall projected trend due to region-specific factors (e.g., climate, population development, culture and tradition, history and current developments in politics or turmoil and many more). For the Esmeraldas region and this modeling approach we adapted the narrative in the following manner:

The simulation is based on the global SSP2 population development scenario [28,29], where time steps have been approximated by linear interpolation between the decadal gridded information. For the subnational regional approach not all values can be obtained scale-specific (yet), making further approximations necessary. To incorporate the overall food demand trend we used the approximated value for kilocalories intake per person per day of 2,614 for Ecuador 2010 [30,31] and a global SSP2 projection value limited to 3000 kcal by 2060 as a moderate increase scenario, between which we also linearly interpolated, as large variations were visible in the approximated data for the current time frame. Projected consumption is the derived demand of the total population per year in kilocalories. This describes an overall growing food consumption trend, wherein the ratio of demands was altered to depict a societal diet shift towards a long-term trend of a higher animal-based diet [27].

To sharpen estimates of population development we calculated the urban population share based on trends derived from World Bank data [32]. In accordance with the model application of a built-up peak demand that is passed on after the population peak, we incorporated the assumption, that the urban population stays stable after this point in time. Based on this projection, we derived the trend for the smallholder share.

We assumed that the footprint changes depended on the societal diet of the total population, total demand, derived demand per agricultural type and smallholder share with an assumed stable adjustment factor expressing the regional conditions (e.g., fertilization and its state subsidies and demands that are satisfied by imports). To approximate the predicted higher animal-based share, we derived the current diet as approximated in the FAO Food Balance Sheet. Firstly, we subtracted all food sources that are in LPB not depicted by terrestrial LUTs. We hold this value static throughout the simulation. Secondly, we

increased the share of pasture-based kcal from 19.61 % to 25 % in 2100 and interpolated in between. The share of cropland-annual is subsequently adjusted per time step. Agroforestry remains stable as it represents already mixed systems.
In the absence of regional descriptive statistics on commercial timber demands and primary data only indicating marginal subsistence demands the overall total population wood demand declines in accordance with the SSP2 global scenario based on a visible declining trend in the UN fuelwood data [23]. Plantations for the case of Esmeraldas depict oil palm plantations. These show like the national wood fuelwood demand of households a sharp declining trend in the national statistics information [33], which we projected into the future.

This narrative and accordingly projected data results in the time series (see below table B14) for model simulation of the smallholder-dominated forest landscape, i.e., the smallholder share, the agricultural footprint per LUT and the wood demands of the population besides plantation area (see S3 File (excel calculations) and S5 File (time series input CSV)). Note that in the regional descriptive statistics no significant commercial demands, especially for timber, are described separately that may already or in the future be satisfied in this landscape extent.

Table B14 SSP2 time series. Highlighted are the probing dates (blue) and specifically the population peak (2060, yellow) and the peak demands year (2100, orange). The population peak here coincides with the peak of the kcal demand projection of 3000 kcal on average per capita per day. Peak demands are simulated for the long-term probing date 2100, which is here the last simulation time step due to data limitation.

YEAR	smallholder_share	2	3	4	5	regional_AGB_demand_per_population_total	REMARKS
**2018**	53.87	0.04735	1.23802	1.16852	119725	11846.522	Initial simulation year weak conservation and enforced conservation
**2019**	53.72	0.04714	1.23785	1.16445	104029	11476.385	
**2020**	53.56	0.04693	1.23786	1.16059	90391	11116.152	
**2021**	53.40	0.04682	1.24026	1.15898	78541	10746.408	
**2022**	53.24	0.04672	1.24280	1.15751	68244	10387.908	
**2023**	53.09	0.04662	1.24546	1.15616	59297	10040.348	
**2024**	52.93	0.04652	1.24824	1.15494	51523	9703.491	
**2025**	52.77	0.04643	1.25115	1.15384	44769	9377.039	Initial simulation year no conservation
**2026**	52.62	0.04634	1.25419	1.15287	38899	9060.720	
**2027**	52.46	0.04626	1.25734	1.15201	33800	8754.267	
**2028**	52.30	0.04619	1.26062	1.15126	29369	8457.398	
**2029**	52.14	0.04611	1.26402	1.15063	25518	8169.890	
**2030**	51.99	0.04605	1.26753	1.15012	22173	7891.469	Probing date short-term
**2031**	51.83	0.04609	1.27402	1.15229	19266	7604.818	
**2032**	51.67	0.04613	1.28060	1.15453	16740	7328.228	
**2033**	51.52	0.04618	1.28728	1.15685	14546	7061.363	
**2034**	51.36	0.04622	1.29405	1.15924	12639	6803.912	
**2035**	51.20	0.04628	1.30092	1.16170	10982	6555.533	
**…**							

**2045**	49.63	0.04748	1.39123	1.20415	2694	4456.536	
**2046**	49.47	0.04767	1.40247	1.21016	2341	4282.161	
**2047**	49.32	0.04786	1.41383	1.21623	2034	4114.541	
**2048**	49.16	0.04805	1.42531	1.22238	1767	3953.412	
**2049**	49.00	0.04825	1.43691	1.22859	1535	3798.539	
**2050**	48.84	0.04844	1.44864	1.23486	1334	3649.669	Probing date mid-term
**2051**	48.69	0.04875	1.46389	1.24410	1159	3498.442	
**2052**	48.53	0.04907	1.47931	1.25342	1007	3353.471	
**2053**	48.37	0.04939	1.49491	1.26284	875	3214.500	
**2054**	48.22	0.04971	1.51068	1.27234	760	3081.279	
**2055**	48.06	0.05003	1.52663	1.28194	661	2953.572	
**2056**	47.90	0.05036	1.54277	1.29164	574	2831.145	
**2057**	47.74	0.05069	1.55908	1.30143	499	2713.791	
**2058**	47.59	0.05102	1.57559	1.31132	433	2601.294	
**2059**	47.43	0.05135	1.59228	1.32130	377	2493.455	
**2060**	47.27	0.05169	1.60916	1.33139	327	2390.079	Population peak
**2061**	47.27	0.05164	1.61409	1.33156	284	2287.216	
**2062**	47.27	0.05160	1.61904	1.33174	247	2188.776	
**2063**	47.27	0.05155	1.62397	1.33191	215	2094.576	
**2064**	47.27	0.05150	1.62891	1.33207	187	2004.430	
**2065**	47.26	0.05146	1.63385	1.33224	162	1918.164	
**2075**	46.88	0.05212	1.72103	1.36384	40	1227.076	
**…**							
**2076**	46.81	0.05231	1.73385	1.37014	35	1172.659	
**2077**	46.73	0.05250	1.74677	1.37649	30	1120.652	
**2078**	46.66	0.05269	1.75980	1.38289	26	1070.950	
**2079**	46.58	0.05287	1.77295	1.38934	23	1023.450	
**2080**	46.50	0.05307	1.78621	1.39585	20	978.054	Probing date long-term
**2081**	46.36	0.05348	1.80700	1.40819	17	933.511	
**2082**	46.22	0.05390	1.82812	1.42072	15	890.990	
**2083**	46.07	0.05432	1.84960	1.43345	13	850.398	
**2084**	45.93	0.05475	1.87142	1.44638	11	811.651	
**…**							
**2095**	44.08	0.06075	2.16465	1.62398	2	483.945	
**2096**	43.88	0.06145	2.19763	1.64434	2	461.511	
**2097**	43.69	0.06216	2.23135	1.66515	2	440.113	
**2098**	43.49	0.06289	2.26585	1.68642	2	419.701	
**2099**	43.29	0.06363	2.30114	1.70818	1	400.230	
**2100**	43.08	0.06439	2.33724	1.73041	1	381.658	Peak demands year

3.2 RAP (new)

New user-defined LPB-RAP parameters:

3.2.1 Net forest increment goal in percent

By default the model simulates the UN goal of three percent forest increase [34], here applied directly for the 2018 status and referring to the nationally defined forest area. This can be adjusted to depict user-defined regionally adapted values for simulation of regional or national ratified goals, which can be further adapted in later years, e.g., as applied in the worst-case scenario or different scenario simulations. Please note, that this model does not actively simulate restoration due to the basic moderate worst-case scenario assumption.

3.2.2 List of LUTs for definition of potential restricted areas

The default setting maximizes the number of LUTs to be evaluated which can be adjusted by the model user. By default the model evaluates LUT IDs: 8 (disturbed forest), 9 (undisturbed forest), 10 (moss, lichen, bare, sparse vegetation), 11 (herbaceous wetland), 12 (water), 23 (RAP reforestation), 24 (RAP other ecosystems). All areas of the user-defined LUTs are extracted in the population peak mplc landscape configuration as an approximation to the maximum available area for conservation.

3.2.3 Forest degradation and regeneration AGB thresholds (new)

For the Esmeraldas we used values published for the neighboring Columbian Chocó region [35] as a basis for the to be calculated AGB percent thresholds in regard to the potential maximum undisturbed AGB value per cell. Thereby we defined for this study the two values as follows:

- Upper user-defined AGB threshold: 80.30 %
- Lower user-defined AGB threshold: 37.77 %

Both are derived from the mean values of low and severe degradation derived from FDI in relation to the described mean value of intact forest.

4. CORRECTION STEP SETTINGS (new)

For this application in high resolution the correction step from remote-sensing-based land cover to approximated land use at terrestrial surface level is applied. Due to this hybrid LULC input map (see section 2.5) we here exclude all land use types except the agricultural types from the simulation. This is done to achieve a more realistic distribution of the formerly randomized simulated agricultural LUTs. In this setting, the model applies all provided parameters including default algorithms to approximate built-up and impacted forest but is forced to simulate the agricultural extent solely based on suitability criteria, thereby not altering the remaining input map information. We, thereby, aimed to approximate the conditions at the terrestrial surface level.

5. SYSTEMIC CHOICES SETTINGS (new)

5.1 Deforestation prior to or post-conversion to other land use types

The model offers the possibilities to simulate deforestation for demand in input biomass prior the en bloc simulated active land use types or after. Deforestation simulated after conversion to other LUTs will reduce net forest extents further and leave deforested pixels for each time step in the amount of apportioned demand. This pattern is not in accordance with the initial input map based on global and national information, where no deforestation in the meaning of deforested but not converted pixel patterns could be derived. To depict the Esmeraldas case study context, we therefore choose the option to simulate deforestation for demand in input biomass prior to conversion to other LUTs. It is the basic assumption in this model that AGB from directly converted former forest plots is not available for the general public (e.g., it might be used for private construction or plot fertilization).

5.2 Incorporation of street pixels for simulation of dynamic built-up

The user-defined option will steer the dynamic built-up simulation due to areas in the proportionally calculated demand in built-up area. In the Esmeraldas study context, the rasterized streets network is the main share of initial built-up pixels after the application of the parameterization correction step (approximating land use below forest cover). LUT built-up serves here as an approximation to all anthropogenic used areas and thereby delivers a landscape share of maximum anthropogenic impact, that is likely not to be in succession, agricultural use or displaying other ecosystem types. Within the LaForeT project built-up also serves as an approximation of housing areas, which could not be derived from project data or secondary sources but should be recognized in a moderate worst-case scenario approach. The spatial allocation might not depict the future distribution exactly (especially for streets), but likely the derived landscape share is of valuable information for landscape planning. Hence, we set the option accordingly as a default for the use case of <= 100 m resolutions to not underestimate the future landscape “built-up” share, and to display potential maximum anthropogenic impact and as the incorporation of street pixels delivers within the proportional growth application a distinct urbanization pattern, which is in accordance with the SSP2 narrative.

5.3 Required number of households for a new settlement

By default, the user-defined value is set to 100 to deliver a strong signal (systemic break between large villages and small towns approximation). This depends on the cities and settlements data used, which might in other cases (not OSM-based) have an applied definition of inhabitants or number of households.

5.4 RAP-LUT25 inputs

This is a user-defined systemic simulation choice. The user can decide to simulate with three predefined classes (thresholds adjusted to user settings) of severe degradation, moderate degradation and low degradation in all selections/combinations to derive the simulation of the RAP-LUT25 restoration of degraded foret. We here chose for demonstration purposes the degradation stages “severe” and “moderate” as input.

S2 File references

1. Ojeda Luna T, Zhunusova E, Günter S, Dieter M. Measuring forest and agricultural income in the Ecuadorian lowland rainforest frontiers: Do deforestation and conservation strategies matter? For Policy Econ. 2020 Feb 1;111:102034.

2. Lippe M, Bithell M, Gotts N, Natalini D, Barbrook-Johnson P, Giupponi C, et al. Using agent-based modelling to simulate social-ecological systems across scales. GeoInformatica. 2019;23(2):269–98.

3. Eguiguren P, Ojeda Luna T, Torres B, Lippe M, Günter S. Ecosystem Service Multifunctionality: Decline and Recovery Pathways in the Amazon and Chocó Lowland Rainforests. Sustainability. 2020 Jan;12(18):7786.

4. Fischer R, Tamayo Cordero F, Ojeda Luna T, Ferrer Velasco R, DeDecker M, Torres B, et al. Interplay of governance elements and their effects on deforestation in tropical landscapes: Quantitative insights from Ecuador. World Dev. 2021 Dec 1;148:105665.

5. GADM. GADM - Ecuador [Internet]. 2021 [cited 2021 Apr 8]. Available from: https://gadm.org/download_country.html

6. OpenStreetMap contributors. OpenStreetMap [Internet]. OpenStreetMap. 2021 [cited 2021 Apr 9]. Available from: https://www.openstreetmap.org/

7. Gustry. QuickOSM — QGIS Python Plugins Repository [Internet]. 2021 [cited 2021 Apr 5]. Available from: https://plugins.qgis.org/plugins/QuickOSM/

8. UNEP/IUCN. Protected Areas (WDPA) [Internet]. Protected Planet. 2016 [cited 2016 Apr 16]. Available from: https://www.protectedplanet.net/en/thematic-areas/wdpa

9. MAE. MAE geoserver. Areas Protegidas (Sistema Nacional de Bosques y Vegetacion Protectora, Zonas Protegidas). Sociobosque (beneficario comunitario, beneficario individual). [Internet]. 2015 [cited 2015 Dec 20]. Available from: http://mapainteractivo.ambiente.gob.ec/geoserver/wms

10. NASA/CGIAR-CSI. SRTM 90m Digital Elevation Data from the CHIAR Consortium for Spatial Information [Internet]. 2021 [cited 2021 Apr 1]. Available from: https://cmr.earthdata.nasa.gov/search/concepts/C1214622194-SCIOPS

11. Buchhorn M, Smets B, Bertels L, De Roo B, Lesiv M, Tsendbazar NE, et al. Copernicus Global Land Service: Land Cover 100m: Version 3 Globe 2015 - 2019: Product User Manual. Zenodo. 2020;

12. Wessel P, Smith WHF. GSHHG - A Global Self-consistent, Hierarchical, High-resolution Geography Database [Internet]. 2021 [cited 2021 Apr 18]. Available from: https://www.soest.hawaii.edu/pwessel/gshhg/

13. MAE. COBERTURA DE LA TIERRA 2018 [Internet]. 2019 [cited 2022 Dec 12]. Available from: http://qa-ide.ambiente.gob.ec:8080/geonetwork/srv/api/records/64f61941-168c-4f4f-837b-5a3172c26d8e121

14. Vancutsem C, Achard F, Pekel JF, Vieilledent G, Carboni S, Simonetti D, et al. Long-term (1990–

2019) monitoring of forest cover changes in the humid tropics. Sci Adv. 2021 Mar 5;7(10):eabe1603.

15. Karger DN, Conrad O, Böhner J, Kawohl T, Kreft H, Soria-Auza RW, et al. Climatologies at high resolution for the earth's land surface areas. Sci Data. 2017 Sep 5;4(1):170122.

16. Bonannella C, Hengl T, Parente L, de Bruin S. Biomes of the world under climate change scenarios: increasing aridity and higher temperatures lead to significant shifts in natural vegetation (PREPRINT, Version 1) [Internet]. 2023 [cited 2023 Feb 13]. Available from: https://www.researchsquare.com

17. Santoro M, Cartus O. ESA Biomass Climate Change Initiative (Biomass_cci): Global datasets of forest above-ground biomass for the years 2010, 2017 and 2018, v3 [Internet]. NERC EDS Centre for Environmental Data Analysis; 2021 [cited 2021 Dec 1]. Available from: https://catalogue.ceda.ac.uk/uuid/5f331c418e9f4935b8eb1b836f8a91b8

18. van der Hilst F, Verstegen JA, Karssenberg D, Faaij APC. Spatiotemporal land use modelling to assess land availability for energy crops - illustrated for Mozambique. GCB Bioenergy. 2012;4(6):859–74.

19. Verstegen JA, Karssenberg D, van der Hilst F, Faaij A. Spatio-temporal uncertainty in Spatial Decision Support Systems: A case study of changing land availability for bioenergy crops in Mozambique. Comput Environ Urban Syst. 2012;36(1):30–42.

20. Newton P, Castle SE, Kinzer AT, Miller DC, Oldekop JA, Linhares-Juvenal T, et al. The number of forest- and tree-proximate people: A new methodology and global estimates [Internet]. Rome, Italy: FAO; 2022 [cited 2023 Aug 21]. 52 p. (Forestry Working Paper). Available from: https://www.fao.org/documents/card/en?details=cc2544en%2f

21. UN. UNdata | record view | Fuelwood [Internet]. 2018 [cited 2021 Apr 11]. Available from: https://data.un.org/Data.aspx?d=EDATA&f=cmID%3AFW%3BtrID%3A1231

22. UN. UNdata | record view | Total population, both sexes combined (thousands) [Internet]. 2018 [cited 2021 Apr 11]. Available from: https://data.un.org/Data.aspx?q=Population&d=PopDiv&f=variableID%3a12

23. Requena Suarez D, Rozendaal DMA, De Sy V, Phillips OL, Alvarez-Dávila E, Anderson-Teixeira K, et al. Estimating aboveground net biomass change for tropical and subtropical forests: Refinement of IPCC default rates using forest plot data. Glob Change Biol. 2019;25(11):3609–24.

24. Cardinael R, Umulisa V, Toudert A, Olivier A, Bockel L, Bernoux M. Revisiting IPCC Tier 1 coefficients for soil organic and biomass carbon storage in agroforestry systems. Environ Res Lett. 2018;13(12).

25. IPCC. IPCC Good Practice Guidance for LULUCF. Chapter 3: LUCF Sector Good Practice Guidance. Chapter 3.2 Forest Land [Internet]. 2003 [cited 2021 Apr 18]. Available from: https://www.ipcc-nggip.iges.or.jp/public/gpglulucf/gpglulucf_files/Chp3/Chp3_2_Forest_Land.pdf

26. GAEZ. GAEZ v3.0. Global Agro-Ecological Zones. User's Guide [Internet]. 2012 [cited 2021 Apr 25].

Available from: https://www.gaez.iiasa.ac.at/docs/GAEZ_User_Guide.pdf

27. Popp A, Calvin K, Fujimori S, Havlik P, Humpenöder F, Stehfest E, et al. Land-use futures in the shared socio-economic pathways. Glob Environ Change. 2017 Jan;42:331–45.

28. Gao J. Downscaling Global Spatial Population Projections from 1/8-degree to 1-km Grid Cells. NCAR Tech Note NCARTN-537STR. 2017;

29. Jones B, O’Neill BC. Spatially explicit global population scenarios consistent with the Shared Socioeconomic Pathways. Environ Res Lett. 2016 Jul;11(8):084003.

30. FAO. FAOSTAT - Food Balances (2010-) [Internet]. [cited 2023 May 21]. Available from: https://www.fao.org/faostat/en/#data/FBS

31. Roser M, Ritchie H, Rosado P. Food Supply. Our World Data [Internet]. 2013 Mar 5 [cited 2023 May 21]; Available from: https://ourworldindata.org/food-supply

32. World Bank. World Bank Open Data - urban population (% of total population) - Ecuador [Internet]. World Bank Open Data. 2023 [cited 2023 May 21]. Available from: https://data.worldbank.org

33. INEC. INEC - online [Internet]. 2023 [cited 2023 May 21]. Available from: https://app.powerbi.com/view?r=eyJrIjoiNjk1M2M4Y2UtNmYwOS00MDk1LWIxYzgtNmVkMzM5ODMzODNlIiwidCI6ImYxNThhMmU4LWNhZWMtNDQwNi1iMGFiLWY1ZTI1OWJkYTExMiJ9&pageName=ReportSection5b660c865b9de068070e

34. Inthakoun L. UN STRATEGIC PLAN FOR FORESTS 2030. 2019;20.

35. Meyer V, Saatchi S, Ferraz A, Xu L, Duque A, García M, et al. Forest degradation and biomass loss along the Chocó region of Colombia. Carbon Balance Manag. 2019 Mar 23;14(1):2.
