# Supplementary material for: Quo vadis, smallholder forest landscape? An introduction to the LPB-RAP model
Source: PLoS One. 2024 Feb 2;19(2):e0297439. doi: 10.1371/journal.pone.0297439 (PMC10836681; doi:10.1371/journal.pone.0297439)
Supplement: S4 File — (PDF) [file pone.0297439.s004.pdf]

S4 File - Case study modeling: selected results

S4 File provides a selection of restriction policy enforcement levels scenario simulation results on the described default scenario assumptions settings (see S2 File). For an overview of the complete secondary model output see S5 File (model files and secondary outputs of CSVs, GIFs and R-based outputs in DIN A4 format).

S4 File to: "Quo vadis, smallholder forest landscape? An introduction to the LPB-RAP model."

CONTENTS

Background information 3
- Scenario changes applied 3
- Scenario differences simulated 5
- Correction step conservation scenarios starting in 2018 - approximation of land use at terrestrial surface level 5
- Input map no conservation scenario starting 2025 10
- Probing dates of annual simulation for the time frame 2018 – 2100 11
- Landscape share or absolute numbers 12
- Gross and net forest in the applied case study modeling 12

1 LPB mplc results (probable) no FLR scenario 13
1.1 LPB mplc simulation uncertainty 13
1.1.1 Numerical time series 14
1.1.2 Spatial probing dates 15
1.2 LPB mplc population aspects, demands and concluding simulated anthropogenic features 18
1.2.1 Population and demands 19
1.2.2 Simulated built-up and urbanization patterns 20
1.3 LPB mplc land use types shares and landscape configuration 24
1.3.1 mplc numerical time series of simulated land use types shares 25
1.3.2 mplc spatial probing dates of simulated land use types shares 28
1.3.3 LUTs development scenario comparison (panels) 31
1.4 LPB mplc pressure aspects 32
1.4.1 Pressure on the population indicated by locally and regionally allocated demands 33
1.4.2 Pressure on the population indicated by use of difficult terrain and unallocated demand 34
1.4.3 Pressure on the population and pressure on restricted areas indicated by simulated use of restricted landscape extents 37
1.4.4 Pressure on restricted areas: land use conflict and forest land use conflict 39
1.4.5 Pressure on forest by deforestation, conversion and degradation of quality 42

2 RAP results (possible within mplc) potential FLR scenario 50
2.1 RAP targeted net forest 50
2.2 RAP maximum - land use types shares and landscape configuration 53
2.2.1 RAP total of formerly converted pixels (RAP-LUTs 21 to 24) 54
2.2.2 RAP of formerly degraded forest pixels (RAP-LUT25) 55
2.2.3 RAP numerical time series 56
2.2.4 RAP spatial probing dates 59
2.3 RAP minimum 62
2.4 RAP potential additional restricted areas 63

Discussion of selected results 65

S4 File references 67

SELECTED RESULTS OF POLICY ENFORCEMENT LEVELS SCENARIO SIMULATIONS

Here we give a selected regional results spectrum of the model output of numeric values (370+ newly coded variables per time step) and selected R output for the set SSP2-4.5 baseline scenario in combination with all three conducted “What-if” policy guideline scenario runs on the default settings (even a smaller selection is presented in the main body). Additionally, GIFs are produced in a run to track development over time for different aspects fast (more than 30 GIFs, mainly in the VIRIDIS color spectrum for accessibility). These are provided in Appendix E.
No further sampling method changes or scenario changes via systemic choices or gradual variations were applied in order to derive the concrete potential impact of forest policy in the meaning of use and enforcement of restricted areas - within an otherwise mainly unchanged framework - discreetly.
The model output describes within the applied scenario narratives and assumptions in detail the potential most probable landscape (LPB, mplc module) trajectory by composition, the according simulation uncertainty and corresponding pressure aspects and finally the possible landscape configuration (RAP module) within the prior mplc. The model concentrates besides these mainly anthropogenic-related aspects, especially on forest-related aspects (majority not covered here). The already coded output provides the basis for a broad range of study designs and can of course be still extended.

Background information

In the following subsections, we provide the background information for the derived numeric and spatial results within this study design (default settings plus policy scenario variation). These may vary in other study designs potentially conductible with this model, e.g. variation of baseline scenario or change of default settings for systemic choices and gradual variations et cetera.

- Scenario changes applied

The applied parameter changes in the three conducted forest policy scenario runs (all other default values stay unchanged) are given in table D1:

Table D1: *Overview over applied changes in model settings for this study*

Parameter	Policy enforcement level scenario<br>weak conservation	Policy enforcement level scenario<br>enforced conservation	Policy enforcement level scenario<br>no conservation
Function of restricted areas in regard to active land use by population	*hurdle, that falls, if pressure increases (redirected pressure on restricted areas)*	*hurdle that cannot be overcome (increased pressure on population)*	*no longer an obstacle due to an ensued repeal (no pressure on population, but possible pressure on former restricted areas)*
**model scenario**	weak conservation	enforced conservation	no conservation
**correction step required**	True	True	False

			(the model runs on the prior scenario output, where the correction step has already been applied)
**No conservation scenario planned, year**	True, 2025	False<br>(or set to True if the no conservation scenario shall be applied on enforced conservation output)	False
**initial simulation year**	2018	2018	2025*<br>(with weak conservation 2025 output as input **)
**time steps until 2100<br>(data limitation)**	83	83	76
**maximum forest age<br>(depends on the underlying satellite data time series and the year chosen for the no conservation scenario)**	36	36	44
**RAP targeted net forest increment**	3	3	7.38***
**prepared inputs folder**	inputs_weak_conservation	inputs_enforced_conservation	inputs_no_conservation<br>(with initial folder simulated for no conservation scenario in the weak_conservation run)
**-> change: excluded areas map content**	null mask	congruent with restricted areas	null mask

* The chosen year for the no conservation scenario is here arbitrary and only used for demonstration purposes of a what-if scenario - no implication of any political development in reality is intended. Since all restricted areas are de-jure still in place, a simulation starting 2018 would not be reasonable (some effect is to be expected), but a relatively near-time date provides potentially the greatest diverging course of the landscape trajectory over the remaining simulated time frame for demonstration of potential impact of an ensued repeal. This year can be user-defined set to any date >= initial simulation year of weak or enforced conservation.

** enforced conservation input should be only used in landscapes where protection is landscape-wide truly enforced, meaning where diverging land use is actually prevented, not posterior sanctioned.

*** The no conservation targeted increment has to account for the lost area of a) simulated land use at terrestrial surface level diminishing net forest area and b) the converted and deforested forest area until the chosen time step (this value is provided as csv output within the prior weak or enforced conservation run).

- Scenario differences simulated

Firstly, you will note in the here depicted policy enforcement level scenarios outputs, that de facto weak conservation and enforced conservation perform in this setting identically. This is based on the interplay of the following factors: (1) The projected SSP2 demand scenario, especially with decreasing demands in oil palm plantations and wood, can be satisfied for all time steps in the parametrized case study area outside restricted areas; thereby, the model has no basis to simulate the weak scenario assumptions, that restricted areas will be used if the landscape is otherwise fully developed. (2) The first point is in the case of the Esmeraldas case study area due to the fact that a) areas of decreasing oil palm plantations are freed for agricultural land use and b) unprotected forest pixels on accessible slopes are available for land use.
Secondly, thereby only the difference between the conservation vs. the no conservation scenario is visible.
Thirdly, we still decided to show here all selected results in comparison since for other landscapes and scenario assumptions the differences between the three policy enforcement level assumptions and their simulation can play a critical role.

- Correction step conservation scenarios starting in 2018 - approximation of land use at terrestrial surface level

In this study, the correction step is used to approximate land use at the terrestrial surface level in the forest landscape, especially in a spatial distribution of the prior randomized simulated agricultural pattern. The spatial distribution of newly allocated land use at the terrestrial surface level is determined by the model algorithms and user-provided information of spatial distributions and distances as well as allocation order and suitability factors, their weights and parameters.

Land cover to land use approximation correction step enforced conservation

ECUADOR Esmeraldas SSP2-4.5

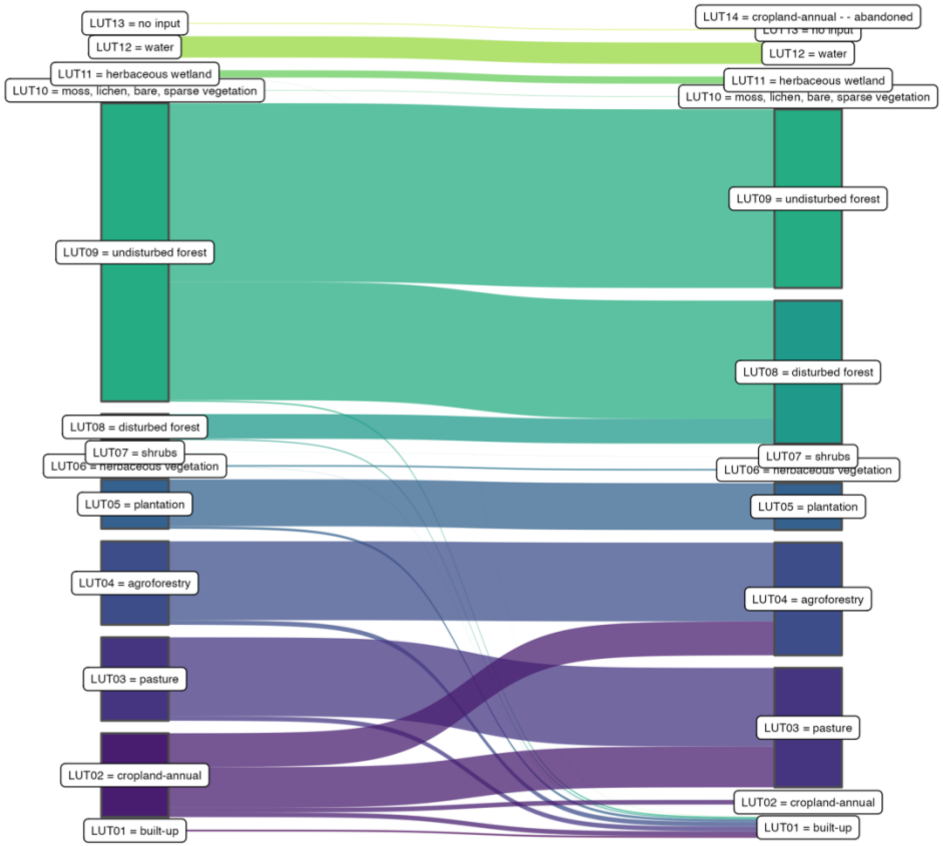

Figure D1: *Visualized is the correction step (conducted with a dynamic model, one sample, one time step, on mean agricultural distances) before simulation in the Monte Carlo framework, approximating from initial land cover (on the left) land use at terrestrial surface level (on the right) based on secondary sources of anthropogenic features, the user-defined allocation order, excluded land use types as well as scenario assumptions based suitability factors, their weights and parameters in the interplay with terrain forces. For the Esmeraldas province the applied rules and scenario assumptions result in congruent simulations for the weak and enforced conservation scenario.*

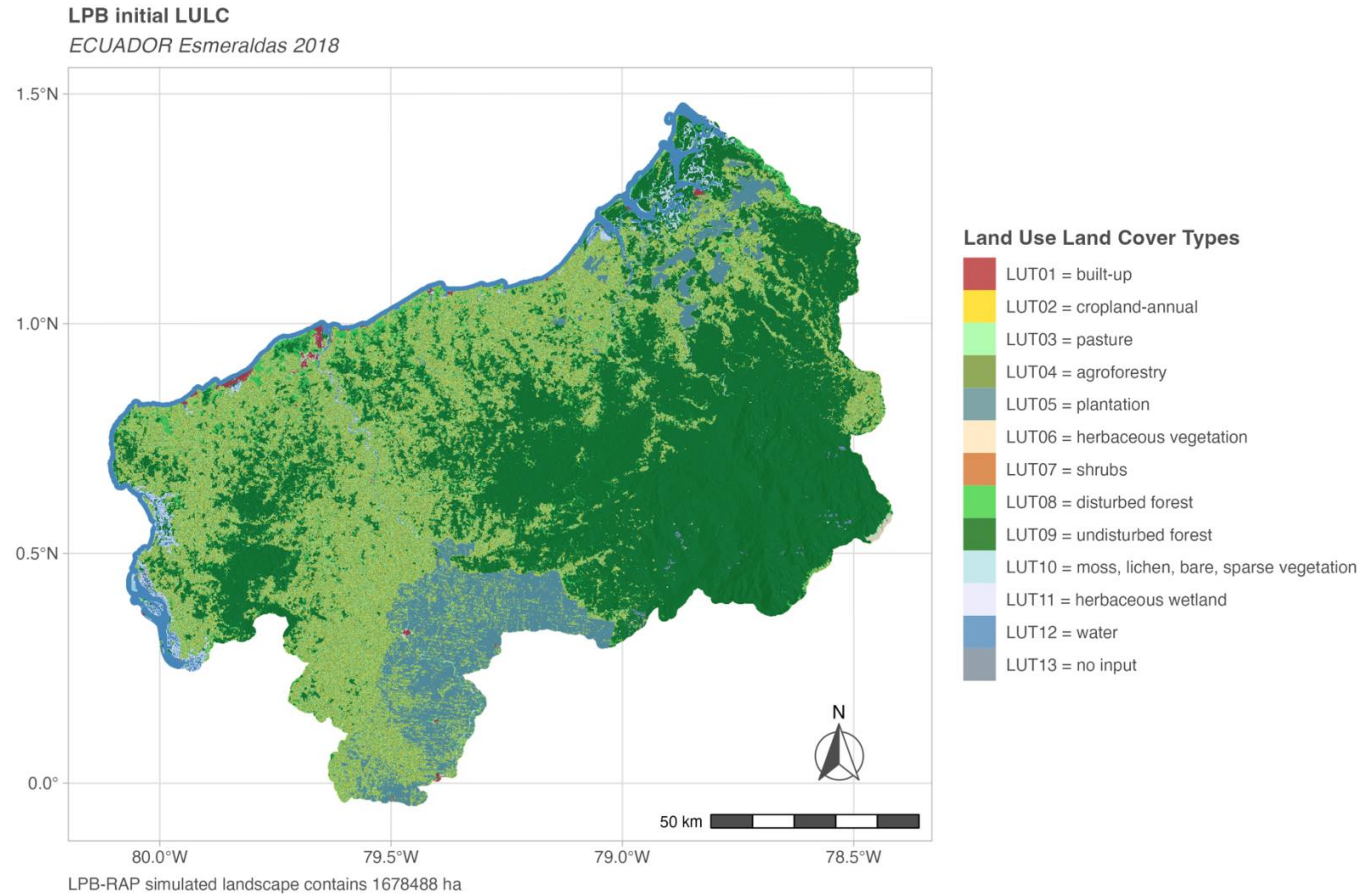

***Figure D2**: The Copernicus-based hybrid map (incorporated information of MAE national LULC map and TMF) depicting a mix of Land cover and Land use as provided to the model for simulation of the correction step. The agricultural pattern, prior simulated randomized, consists of nearly equal shares of the LUTs 2, 3 and 4.*

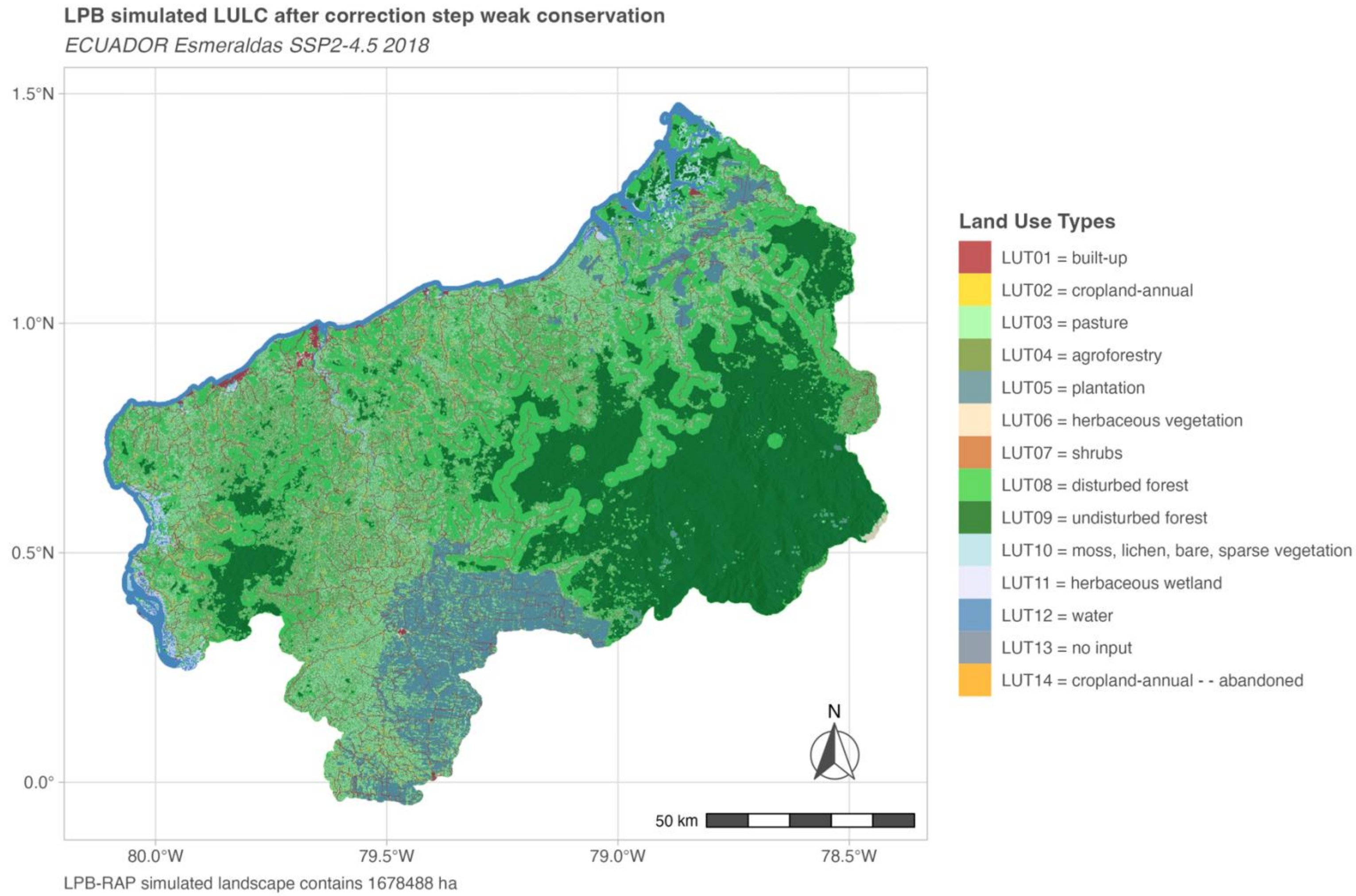

***Figure D3**: The spatial distribution of initial conditions after the applied correction step as supplied to the model for dynamic simulation in the Monte Carlo framework. Note that the landscape has been enriched by information of anthropogenic features, an application of corresponding remaining forest quality and now approximated land use at terrestrial surface level according to initial simulation year information and scenario assumptions.*

Table D2: *Correction step transition in absolute numbers*

LUT	1	2*	3*	4*	5	6	7	8	9	10	11	12**	13***	14****	15	16	17	18
**initial**	4475	213933	212966	213113	125944	4384	147	65548	762091	951	18751	54551	1634	0	0	0	0	0
**Conservation scenarios**	52628	11668	305071	287945	119725	4029	137	365363	456414	936	18476	54458	1634	4	0	0	0	0
**remarks**	application of static and initial anthropogenic features	allocation of demand<br>* pre-simulation simulated randomized agriculture re-distributed based on suitability factors and demands			reduced by allocation of anthropogenic features	reduced by allocation of demand and anthropogenic features/impact<br>** LUT12 = water is affected by allocation of anthropogenic features (bridges for streets etc.), otherwise no simulation source LUT<br>***LUT13 = no input: overall static - no simulation information; here untouched since no anthropogenic features overlap								****Uncertainty based on rounding	Exact demands simulation		Not simulated in correction step	

The four as cropland-annual - - abandoned simulated pixels depict an uncertainty of 0.03 % in regard to the LUT (11,672 pixel in total) and 0.00024 % in regard to the entire landscape simulation (1,678,488 pixel entirely). However, these pixels are eliminated in t = 2 by succession.

- Input map no conservation scenario starting 2025

Here based on the weak conservation scenario 2025 projection. The input map is directly used since no further correction step is required.

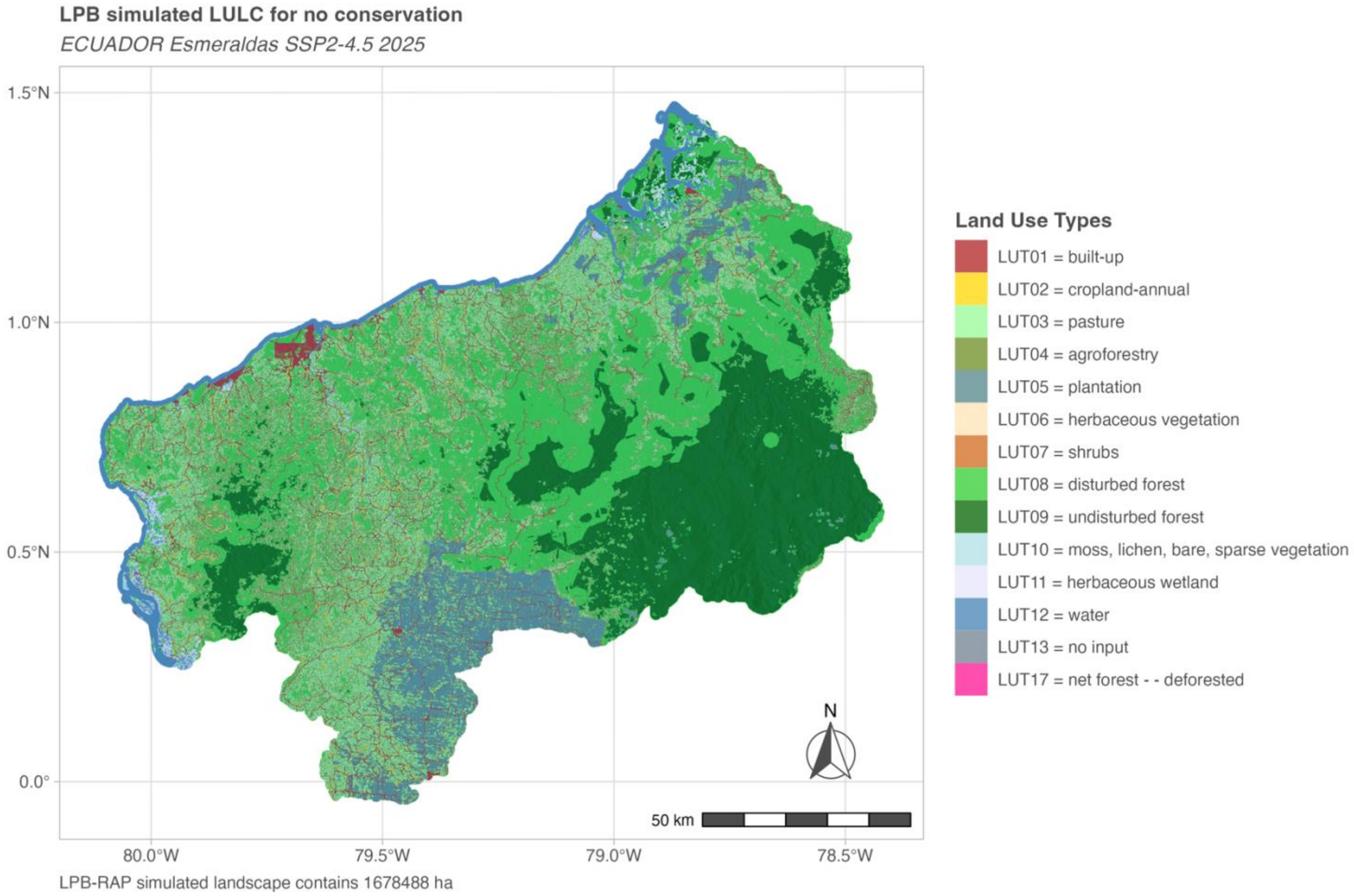

Figure D4: *By user choice, the output of the weak or enforced conservation scenario for any simulation year >= the initial simulation year can be produced and used for simulation of the no conservation scenario as the initial land use map.*

- Probing dates of annual simulation for the time frame 2018 – 2100

All results are produced with an annual time step (see Appendix E). In the following diagrams based on the provided CSV numeric output the given user-defined probing dates according to table D3 are highlighted and the according maps for these time steps per category and policy scenario provided. For visualization of annual development, we encourage the reader to visit the provided GitHub repository (i.e., Appendix E) and watch the result-GIFs per scenario, based on annual time steps for the complete mplc/RAP landscape configuration and thematic subsets. The here provided spatial syllabi based on probing dates cannot capture the complete dynamics as shown in visualization and absolute numbers per time step.

Table D3: *Probing dates*

Probing date	Rationale	Weak conservation	Enforced Conservation	No conservation
1st probing date: initial simulation year	conditions of simulation start year (time step 1)	2018	2018	2025
2nd probing date	conditions simulated short-term	2030		
3rd probing date	conditions simulated mid-term	2050		
4th probing date: population peak (pp)	regional simulated population peak (maximum anthropogenic impact within model logic)	2060 for Esmeraldas (population datasets)		
5th probing date	conditions long-term	2080		
6th probing date: peak demands (pd) year	conditions of long-term simulation end (time step 83/76) respectively in this scenario the peak demands year	2100		

- Landscape share or absolute numbers

For the results depicted in the following sections we use mainly the derived landscape share in percent (100 % = 1,678,488 ha) for a better overview of proportionalities within the simulated landscape, pixel values are displayed where the values are very low or a discrete depiction in absolute numbers gives a better impression of the magnitude. Note that the simulated landscape contains the buffer area depicting the coastline and province border area.

- Gross and net forest in the applied case study modeling

Conceptually gross forest depicts all potential forest cells in the landscape (including p.r.n. agroforestry and p.r.n. plantation if not stated only for undisturbed and disturbed forest), net forest, in contrast, originates from the forest extent declared by the national LULC map and its dynamic simulation and is based solely on the types disturbed and undisturbed forest (as an approximation of forest characteristics at terrestrial surface level), i.e. in case of Esmeraldas the initial net forest distribution is based on forest cover >= 30 % and dynamically simulated in contraction and expansion by conversion and deforestation or neighborhood operations on succession forest pixels after the initial correction step.

1 LPB mplc results (probable) no FLR scenario

The provided results are produced in the two base model modules (p.r.n. including the correction step) and depict the simulated most probable landscape configuration. The analysis is conducted on the probabilistic and/or aggregated results in the mplc module.

1.1 LPB mplc simulation uncertainty

For ease of interpretation, the probability range has been categorized into seven classes for numeric and spatial visual output. Naturally, the model simulates the highest probability for steady land cover or land use in all three scenarios, i.e., especially for the final land use type built-up, plantations in part as well as land use near settlement points and secluded forest areas. In the enforced conservation scenario accordingly also for restricted areas (here mainly only succession to undisturbed forest can vary in addition to population-based settlements pixels and rotation of existing plantation pixels). Uncertainty occurs concludingly mostly at the dynamic forest or agricultural frontier where the stochastic, probabilistic modeling approach has a certain variability despite the deterministic setting of allocation order, terrain parameters and suitability factors, their weights and parameters. Depending on the varying trajectories of the samples the overall class “100 % probability” resp. the according landscape share declines gradually over time. Note that by corrective allocation to display discrete demand partially land use type pixels with lower probability can be in place since the allocation order is applied.

1.1.1 Numerical time series

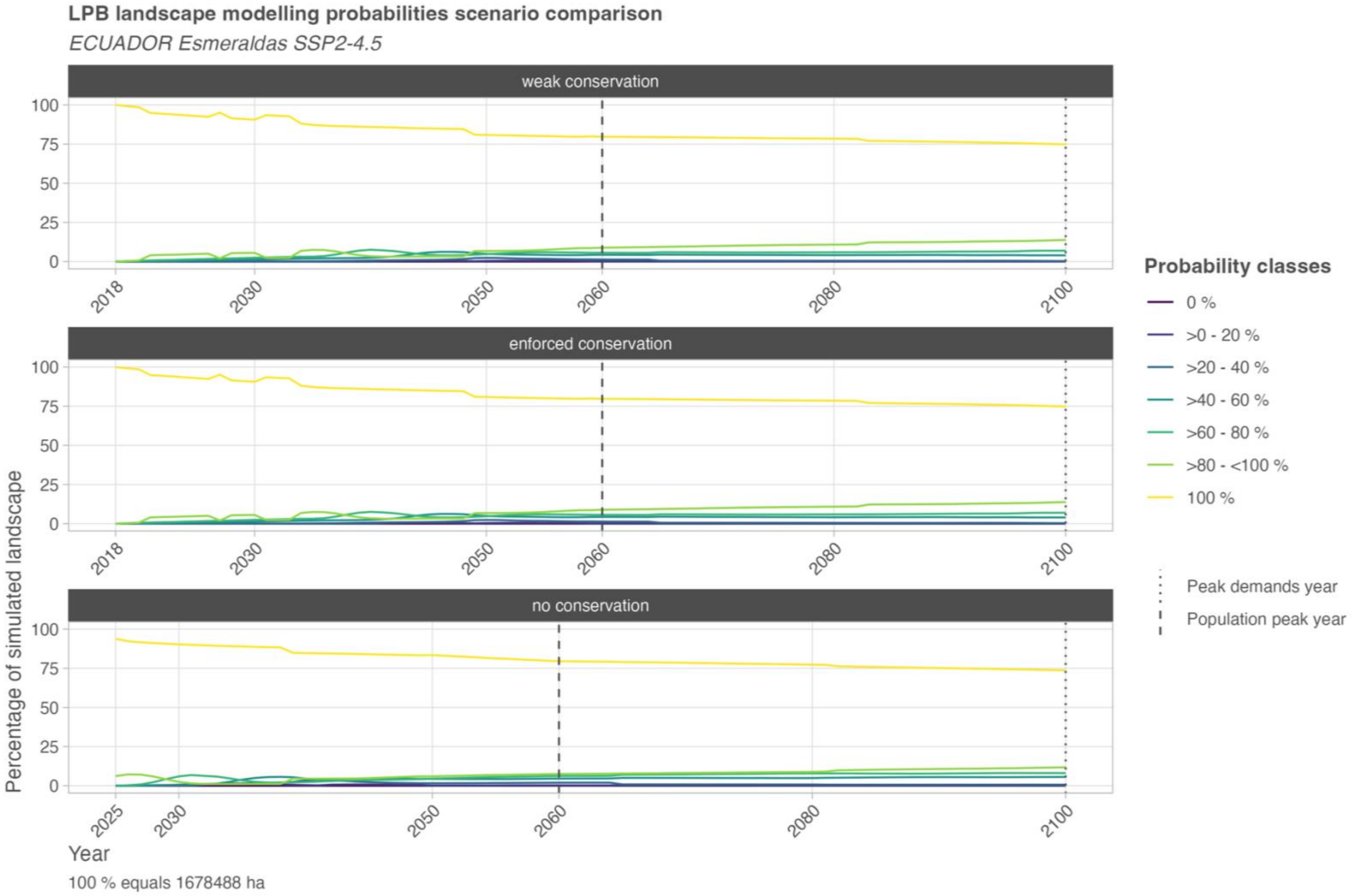

Figure D5: *Depicted is the numerical analysis results visualization of landscape modeling probabilities per pixel respectively the according landscape share. For their spatial distributions see the following figure. Marked are the probing dates according to the table D3 "Probing dates".*

1.1.2 Spatial probing dates

Probing date	Weak conservation	Enforced conservation	No conservation
**1 (2018/2025)**	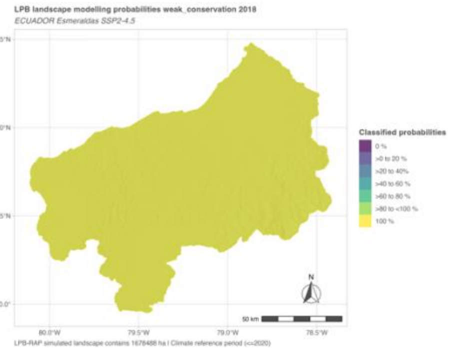	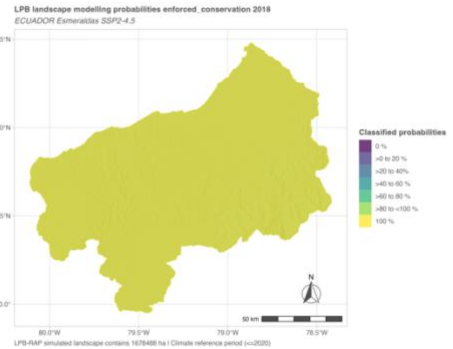	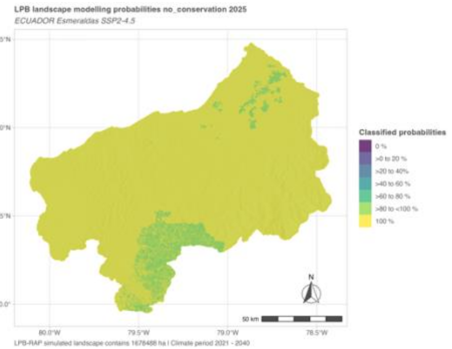
**2 (2030)**	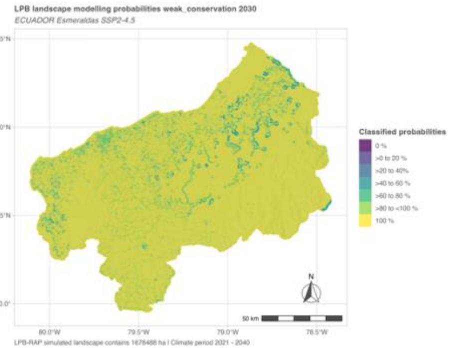	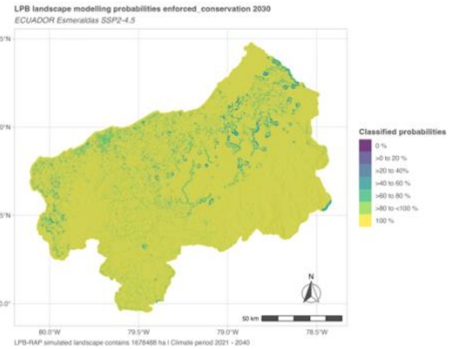	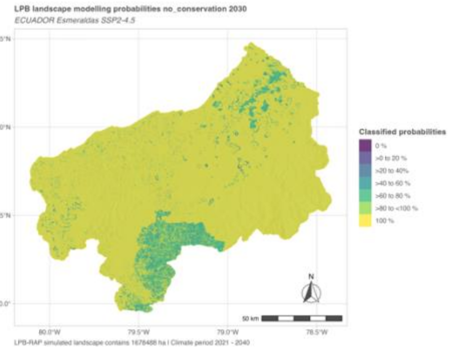

3 (2050)

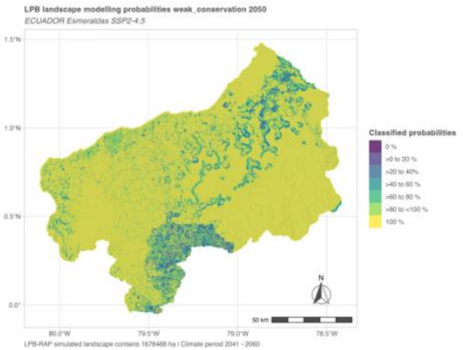

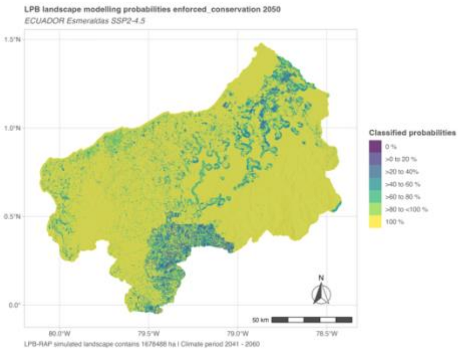

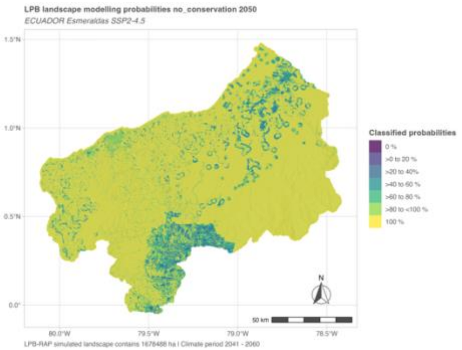

4 (2060, pp)

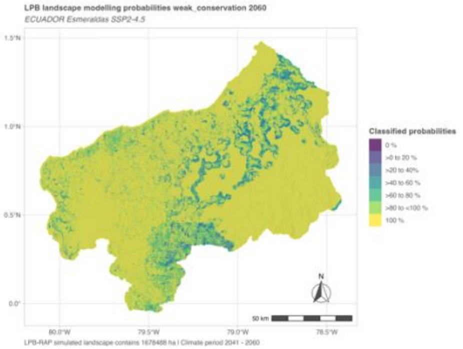

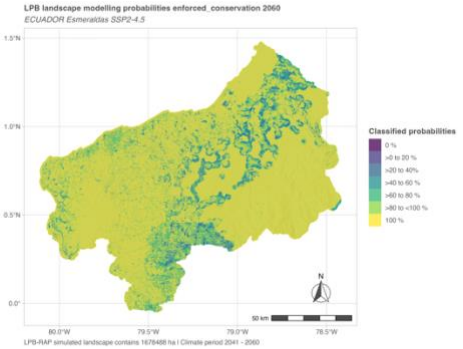

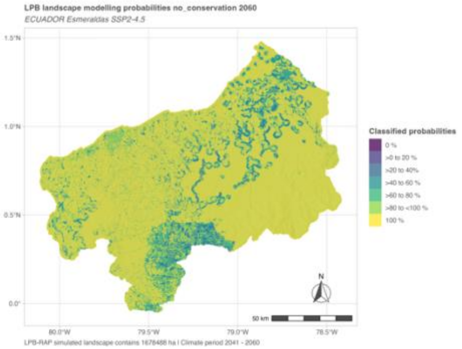

5 (2080)

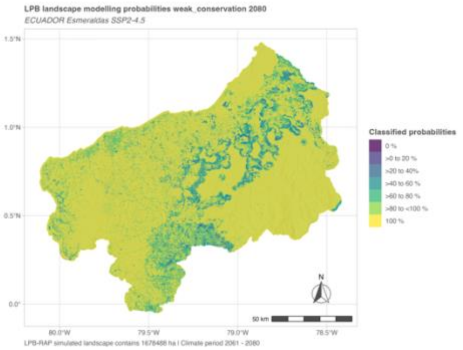

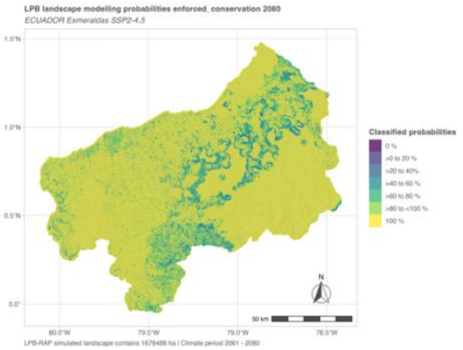

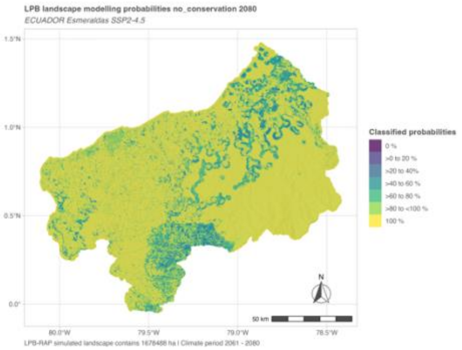

6 (2100, pd)

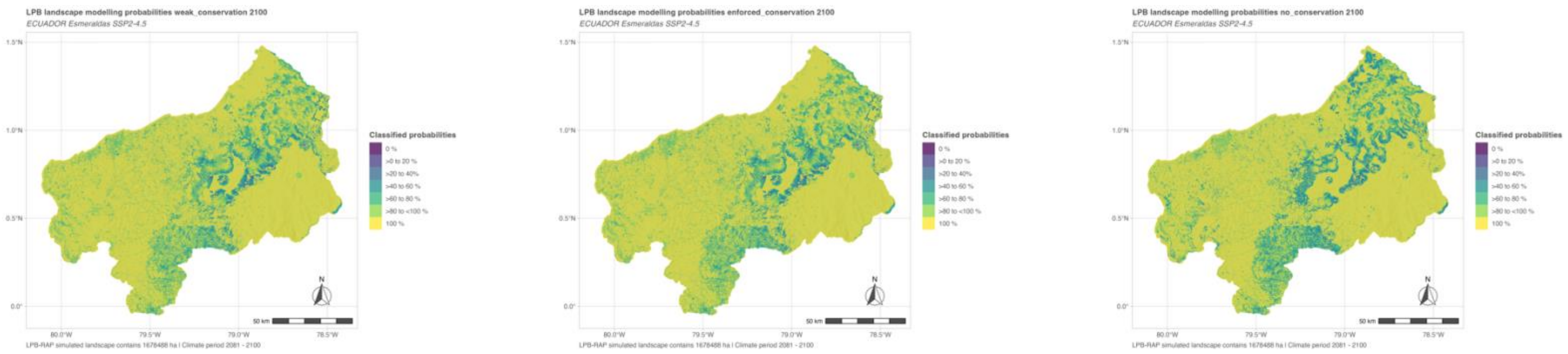

Figure D6: *Depicted are the spatial distributions of probability respectively uncertainty produced in the LULCC_mplc module (here after corrective allocation), according to the user-defined probing dates on annual production. Visible are the emerging patterns of uncertainty simulated by the underlying rulesets for simulation.*

1.2 LPB mplc population aspects, demands and concluding simulated anthropogenic features

Population, smallholder share, their demands in agricultural land use types and the on the entire population-based demand in woody biomass (AGB) are simulated deterministically as part of the baseline scenario and therefore numerically equal in all three policy scenarios (LULCC_basic and LULCC_mplc. The demand in additional built up is a function of total population and simulated built-up of the last time step and can therefore theoretically vary between guideline scenarios depending on the regional landscape configuration (in the case of Esmeraldas this does not apply). For all three scenarios the deterministic demand is in this study application the same.

1.2.1 Population and demands

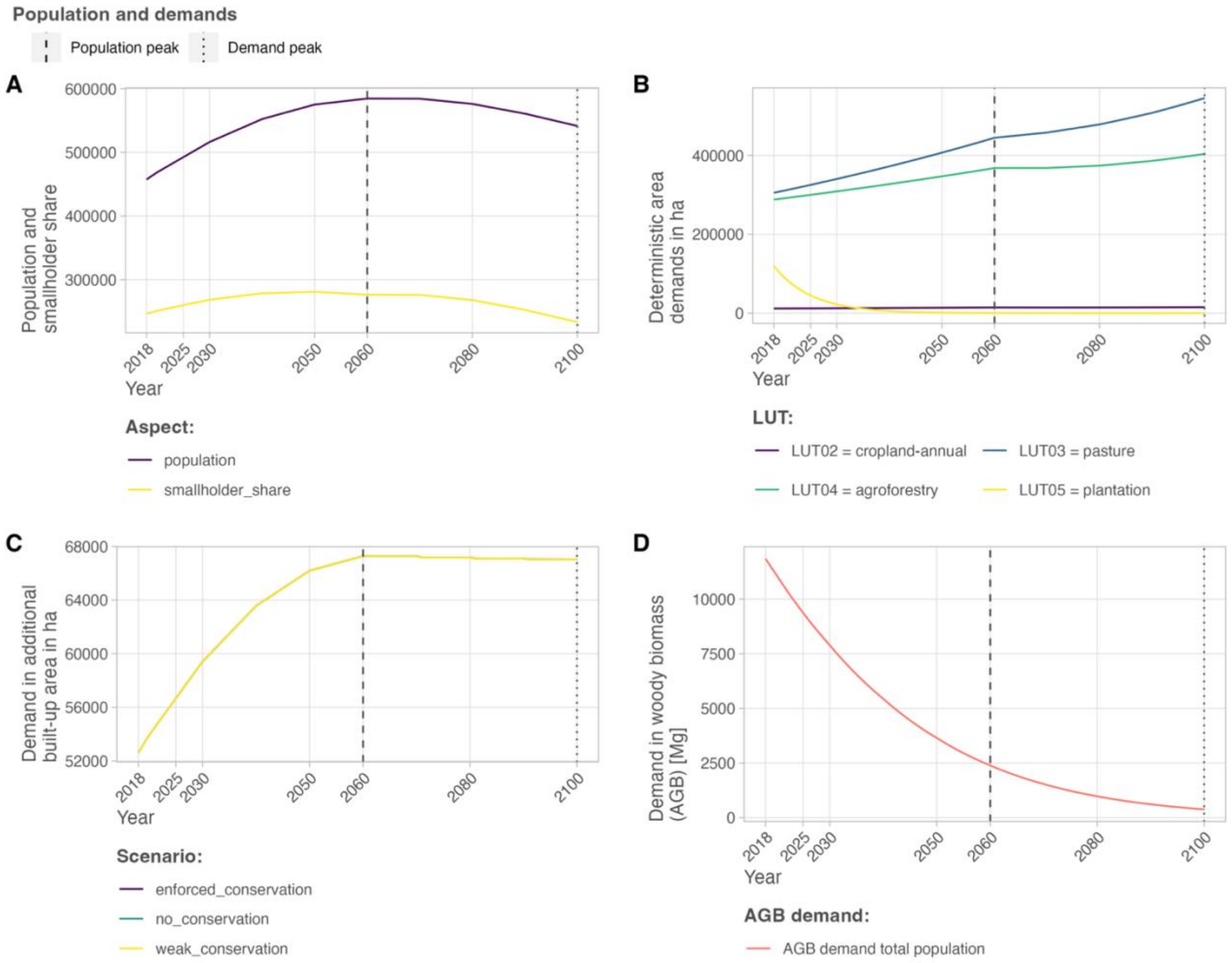

Figure D7: *The figure depicts the main drivers of the long-term land use simulation. Note that in the case of the Esmeraldas province, these demands are entirely met throughout the simulation. Demand in additional built-up is simulated until the population peak, after which the peak demand is passed on since structures still exist even if abandoned. Marked are the probing dates according to the table D3 "Probing dates".*

1.2.2 Simulated built-up and urbanization patterns

The effect of population density and settlements locations to simulate built-up may vary across the guideline scenarios, but for the Esmeraldas this is not the case. Total built-up is overall similar due to in general population-based demand, but is subject to the dynamic rule-of-three algorithm. The deterministic basis is provided by 1 ha pixels of streets and cities (both static) as well as settlement pixels (dynamic). Additional built-up is simulated based on the maximum demand for LUT01 over all samples per time step (based on the simulated area of the last time step, therefore it can theoretically vary across scenarios depending on the landscape) and starting with the population peak the peak demand is simulated for all remaining time steps, since sealing structures still exist even if abandoned. Note that all dynamic settlement points are simulated in all three scenarios based on the steady spatially explicit population input. This results for the conservation scenarios still in singular settlement points (built-up pixels) in restricted areas, where only the anthropogenic impact distance is applied, but no further land use is simulated.

The emergent urbanization pattern shows only slight differences due to unrestricted use, delayed use of or per se excluded restricted areas in between scenarios. Here partially the population original resolution of 1 km can be seen as an artifact. All built-up pixels remain after the population peak even if theoretically abandoned (2060 = 2100 for settlements; dynamic additional built-up can vary), since at terrestrial surface level the structure is likely still sealing soil.

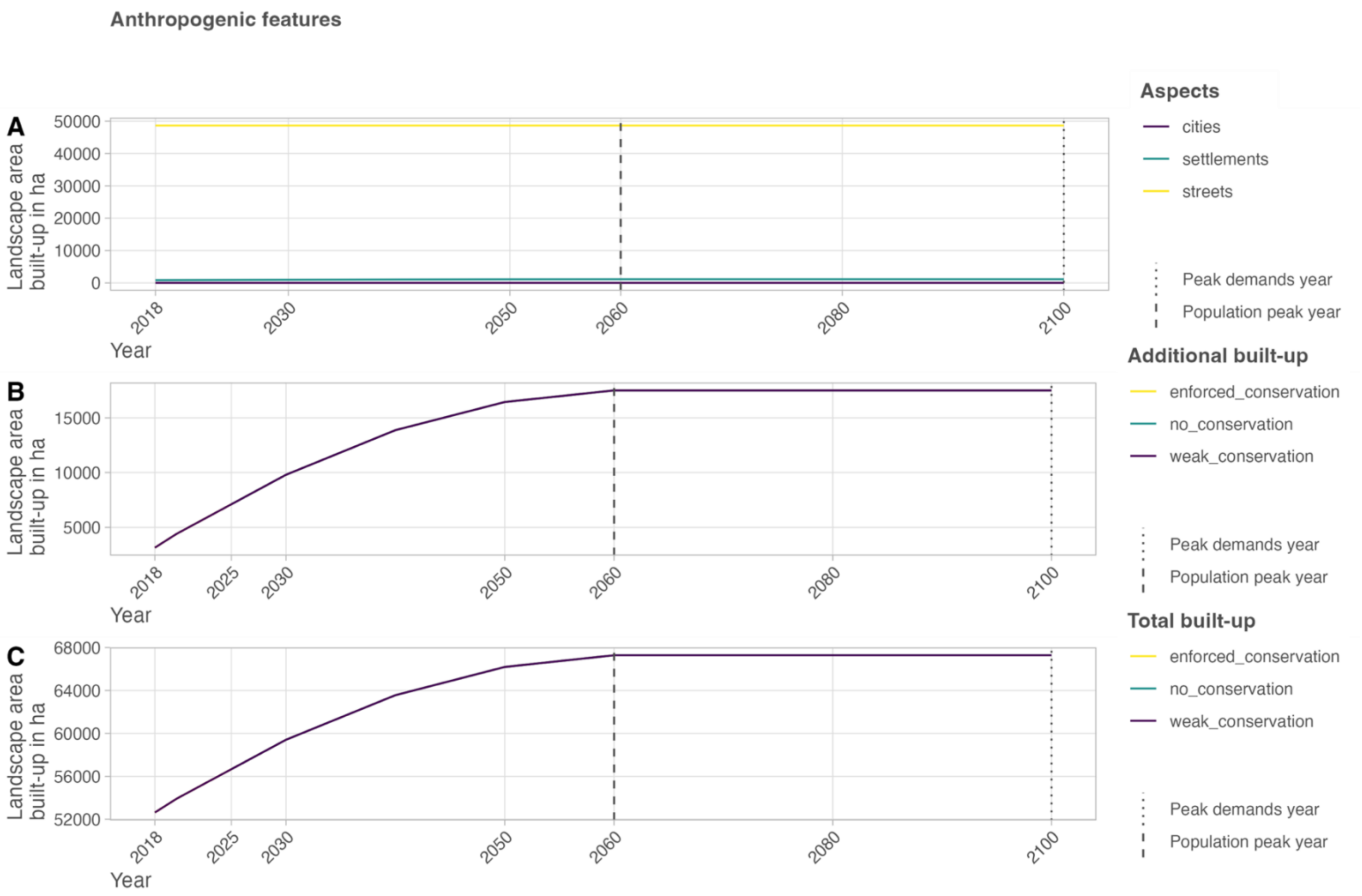

Figure D8: *The figure shows the development and proportion of built-up subtypes and in total. While cities (n=11) and streets are simulated as static, the dynamic settlements gain core settlement points during dynamic simulation (initial: 827, final: 1121). Additional built-up (i.e., built-up initially and simulated which is not city or settlement core pixel and not a street pixel) is scenario dependent but in the case of Esmeraldas de facto equal in all three scenarios. Total built-up is simulated until the population peak after which the peak demand is passed on since structures still exist even if abandoned. Marked are the probing dates according to the table D3 "Probing dates".*

Probing date	Weak conservation	Enforced conservation	No conservation
1 (2018/2025)	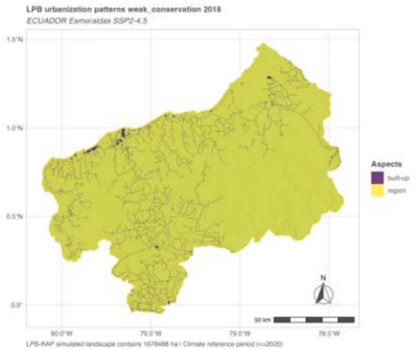	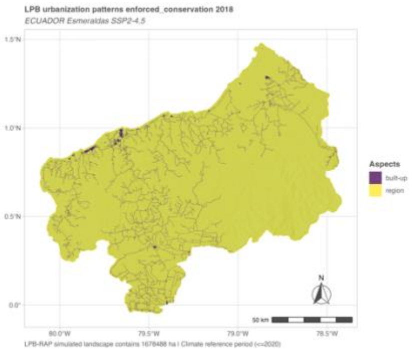	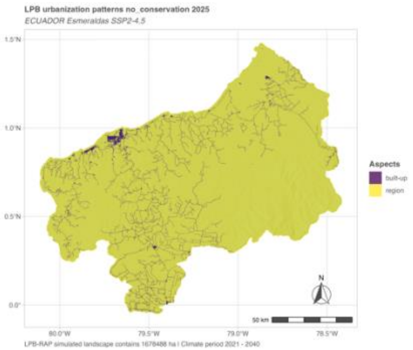
2 (2030)	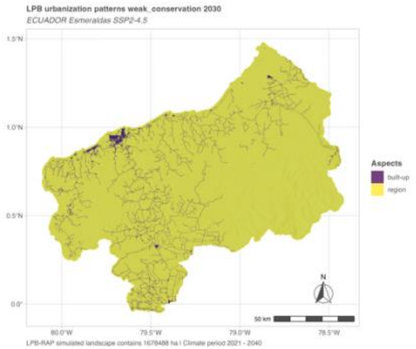	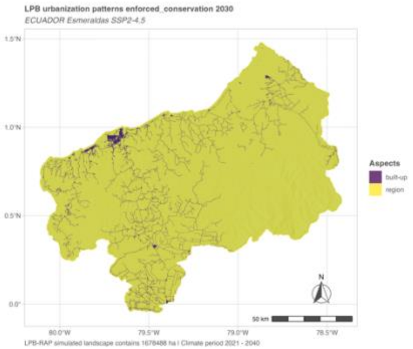	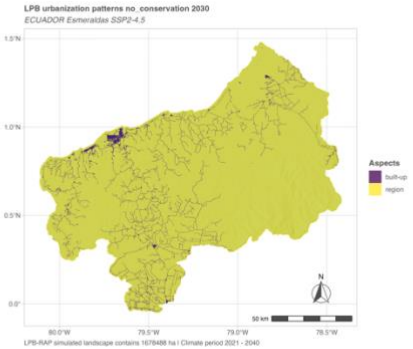
3 (2050)	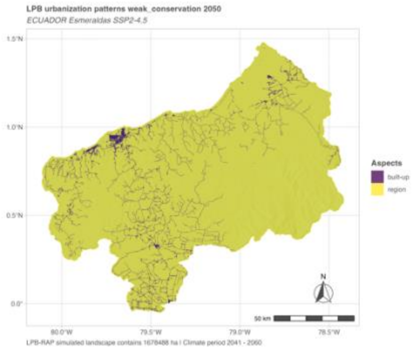	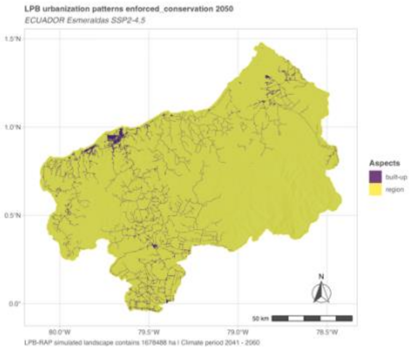	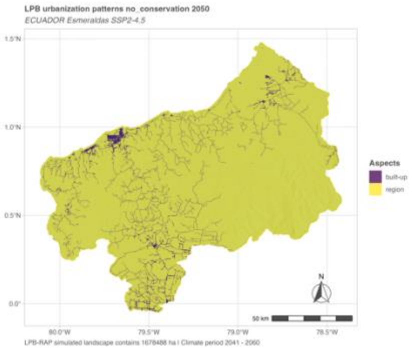

4 (2060, pp)

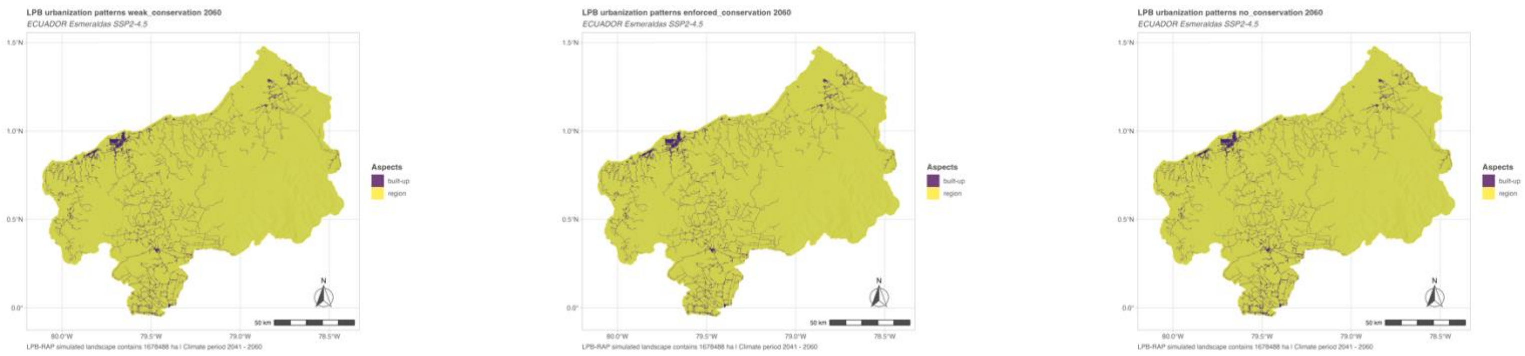

5 (2080)

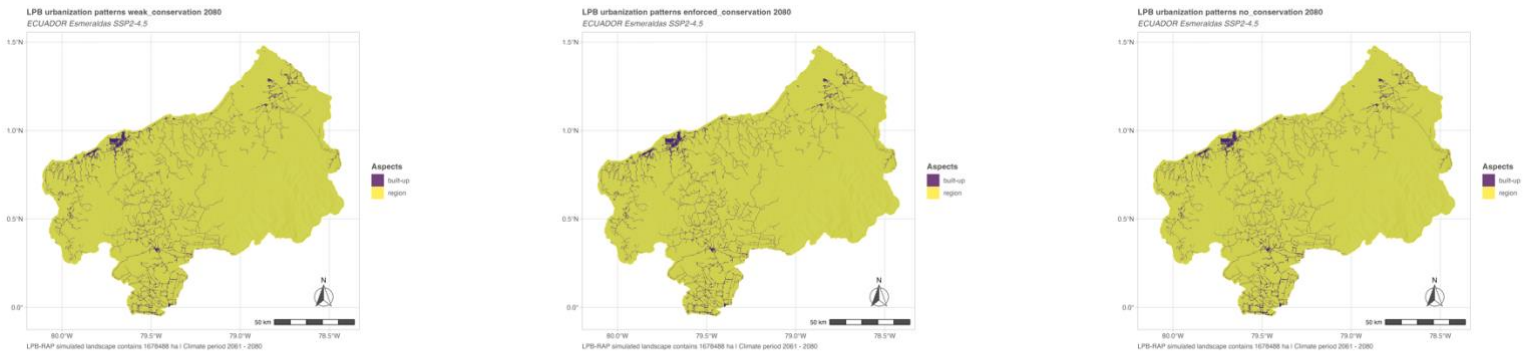

6 (2100, pd)

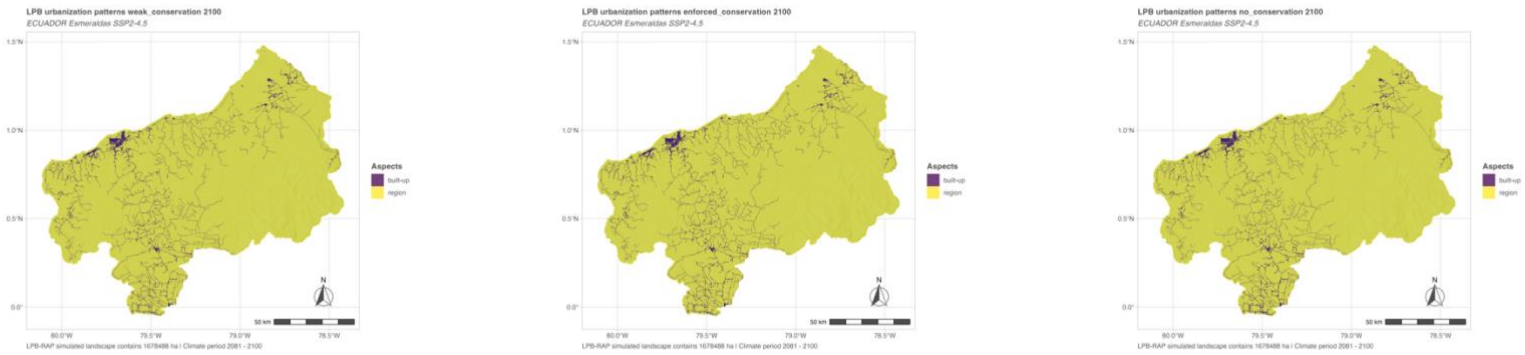

Figure D9: *Here depicted are the post-simulation extracted simulated urbanization patterns in regard to the distribution of the land use type built-up, depicting infrastructure, housing agglomerated around the static cities and street pixels as well as dynamic settlement pixels and initial remote sensing based additional built-up pixels.*

1.3 LPB mplc land use types shares and landscape configuration

Due to deterministic demand the land use type shares for LUT01 to LUT05 (primary active land use types) are equal within the three scenarios (depending on available area). LUT17 = deforestation of net forest is a more complex function of deterministic demand, climate period dependent maximum potential undisturbed AGB simulation with a stochastic increment and simulated land use change, all of which account for the simulated total number of required deforestation area to satisfy the demand in woody biomass (AGB). Differences can be seen for the passive land use types, which are subject to different degrees of pressures according to the simulated scenario (regional evasion space). Note that plantations are symbolized by LUT05 (plantation plot in use) and LUT18 (plantation plot harvested) combined as a landscape share.

1.3.1 mplc numerical time series of simulated land use types shares

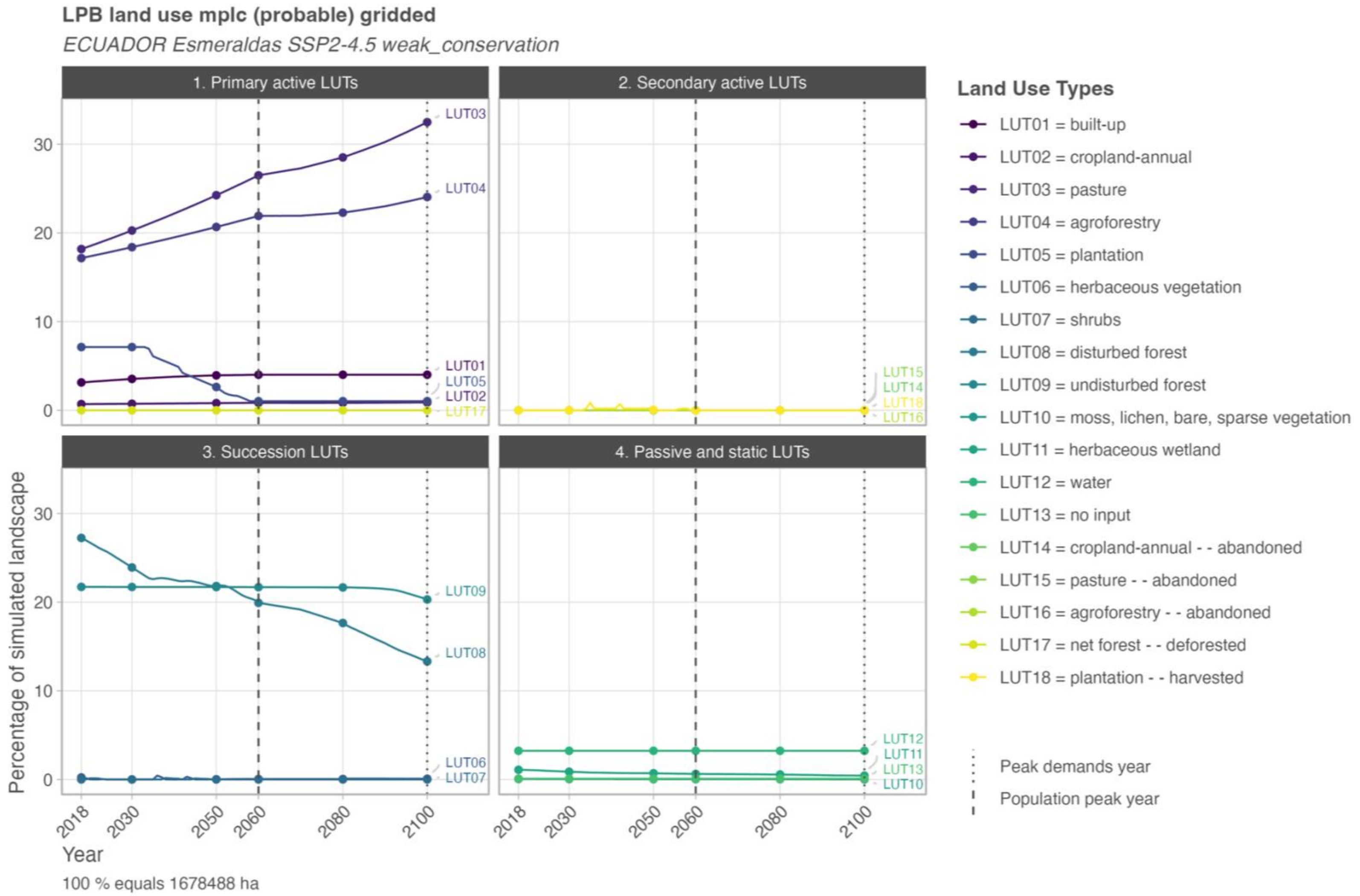

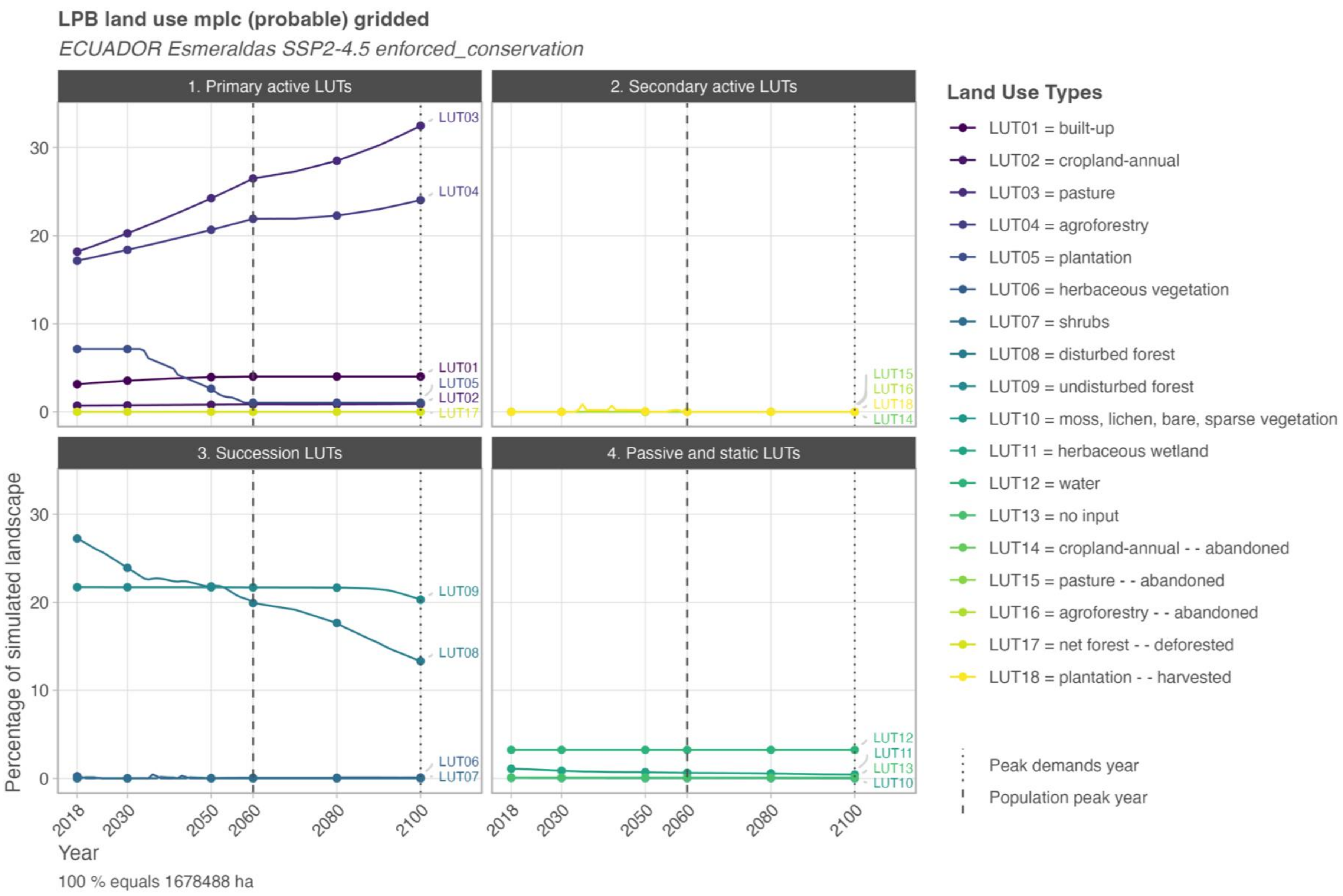
LPB land use mplc (probable) gridded
ECUADOR Esmeraldas SSP2-4.5 enforced_conservation
1. Primary active LUTs
2. Secondary active LUTs
3. Succession LUTs
4. Passive and static LUTs
Percentage of simulated landscape
Year
100 % equals 1678488 ha
LUT03
LUT04
LUT01
LUT05
LUT02
LUT17
LUT15
LUT16
LUT18
LUT14
LUT09
LUT08
LUT06
LUT07
LUT12
LUT11
LUT13
LUT10
Land Use Types
LUT01 = built-up
LUT02 = cropland-annual
LUT03 = pasture
LUT04 = agroforestry
LUT05 = plantation
LUT06 = herbaceous vegetation
LUT07 = shrubs
LUT08 = disturbed forest
LUT09 = undisturbed forest
LUT10 = moss, lichen, bare, sparse vegetation
LUT11 = herbaceous wetland
LUT12 = water
LUT13 = no input
LUT14 = cropland-annual - - abandoned
LUT15 = pasture - - abandoned
LUT16 = agroforestry - - abandoned
LUT17 = net forest - - deforested
LUT18 = plantation - - harvested
Peak demands year
Population peak year

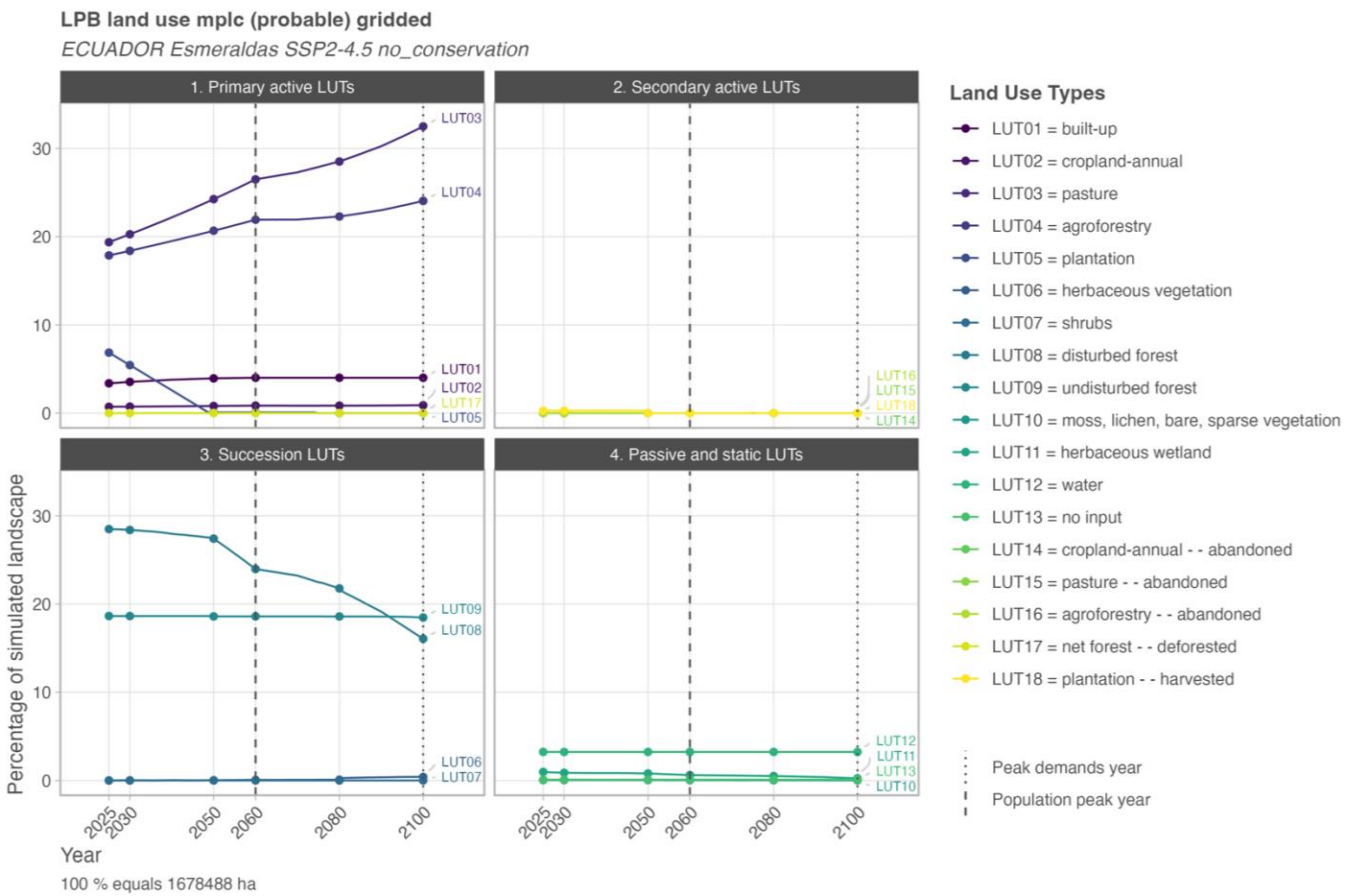

Figure D10: *The diagrams display the entire landscape configuration within the aggregating mplc simulation divided in thematic groups of land use types for each simulated policy scenario. Note that LUT17 = net forest - - deforested is an indirect active land use type but due to its landscape shaping factor here classified as primary active. Note that the trends based on equal demands are similar in all three scenarios with the exception of the development within the succession LUTs section. Marked are the probing dates according to the table D3 “Probing dates”, which display graphically the need to analyze on the time step interval as conducted in this model to capture all landscape configurations.*

1.3.2 mplc spatial probing dates of simulated land use types shares

<table>
<tr><th>Probing date</th><th>Weak conservation</th><th>Enforced conservation</th><th>No conservation</th></tr>
<tr><td>1 (2018/2025)</td>
<td>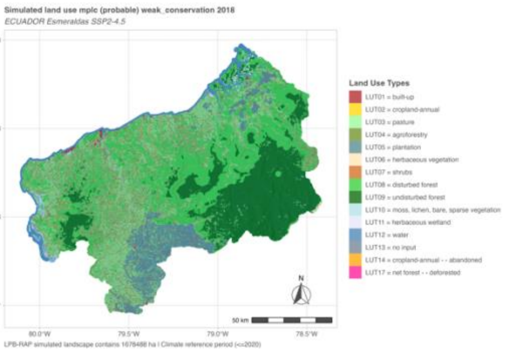
</td>
<td>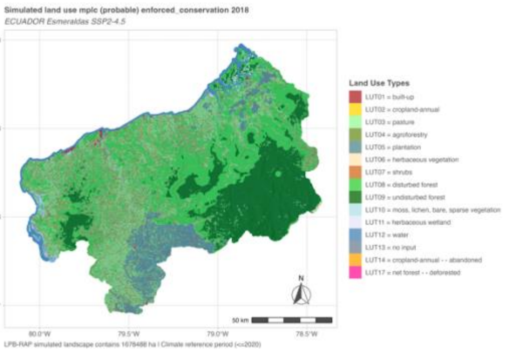
</td>
<td>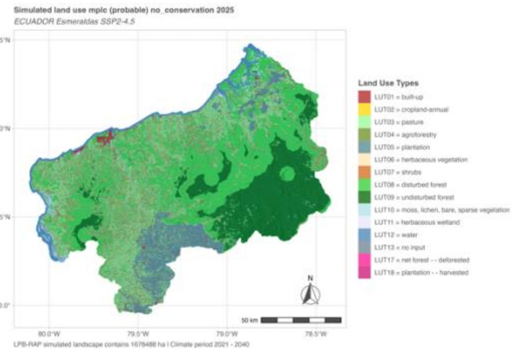
</td></tr>
<tr><td>2 (2030)</td>
<td>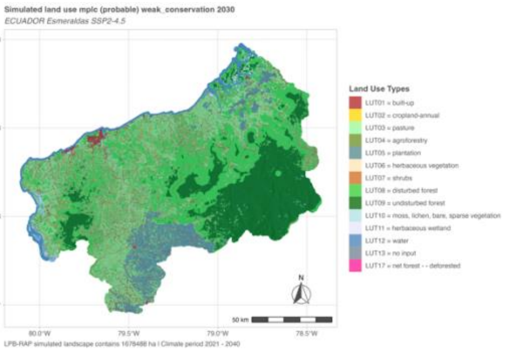
</td>
<td>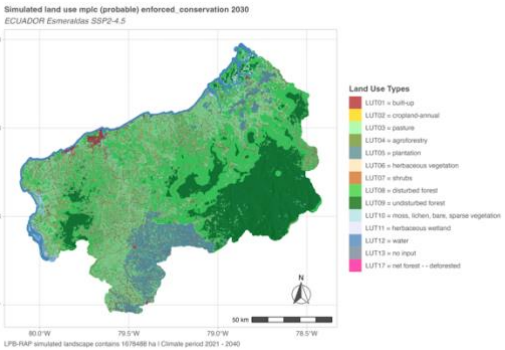
</td>
<td>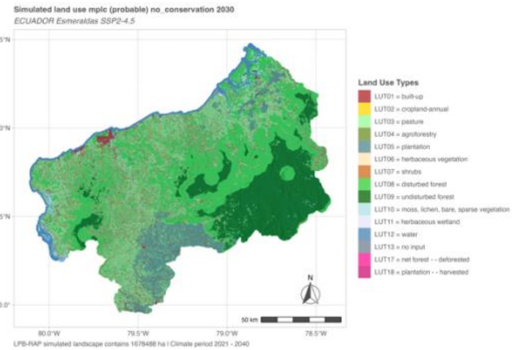
</td></tr>
</table>

3 (2050)

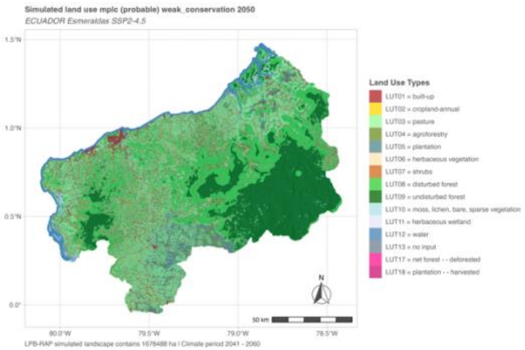

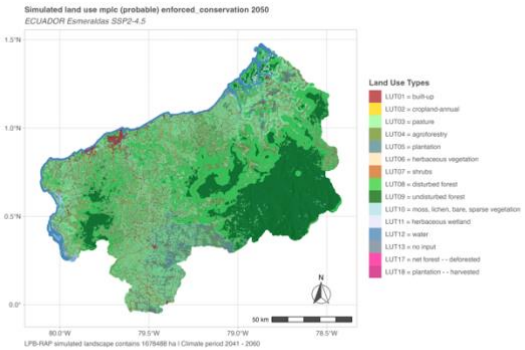

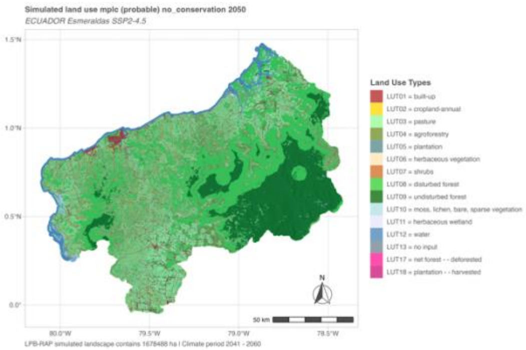

4 (2060, pp)

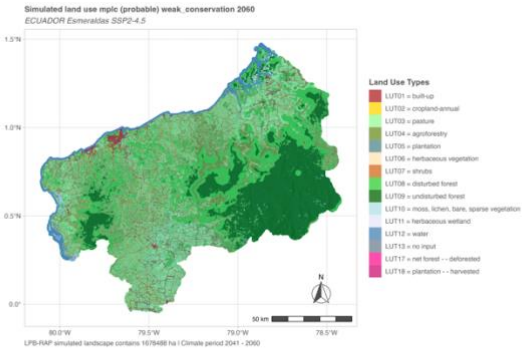

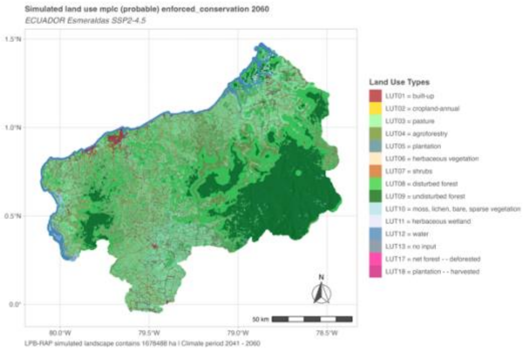

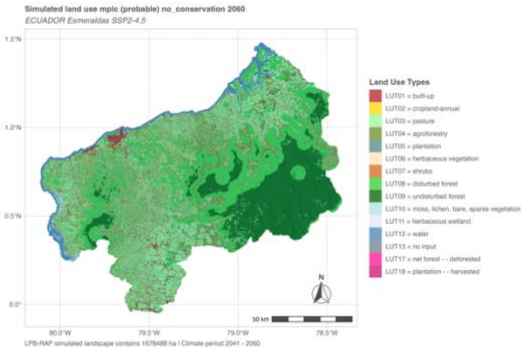

5 (2080)

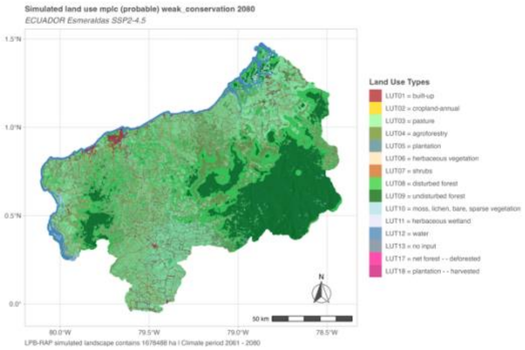

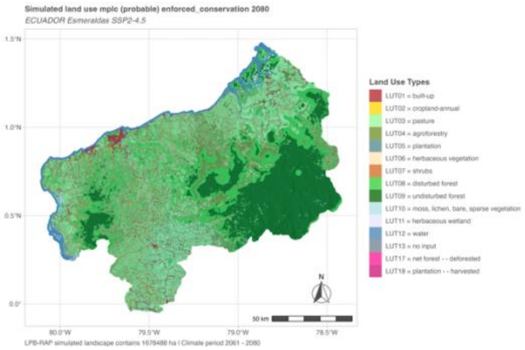

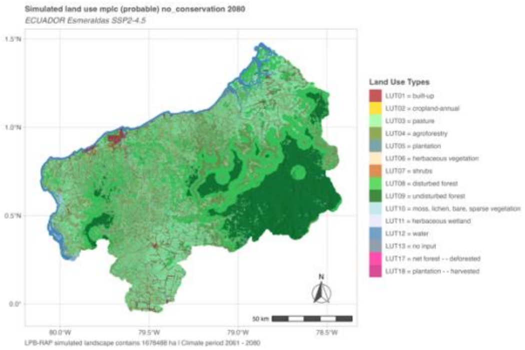

6 (2100, pd)	Note that after the population peak the land use types comprise the secondary active abandoned agricultural land use types, concluding the full spectrum of simulated land use types in LPB.

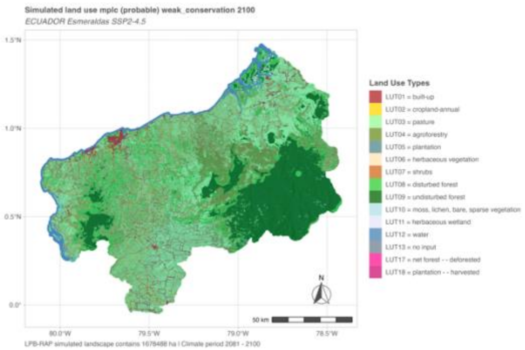

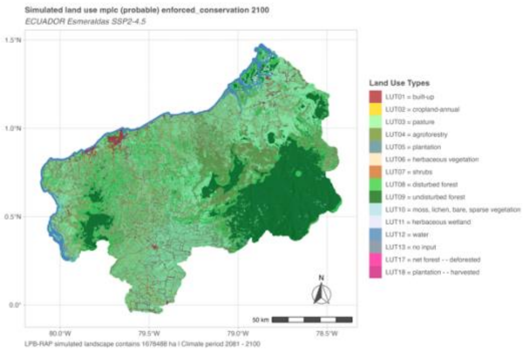

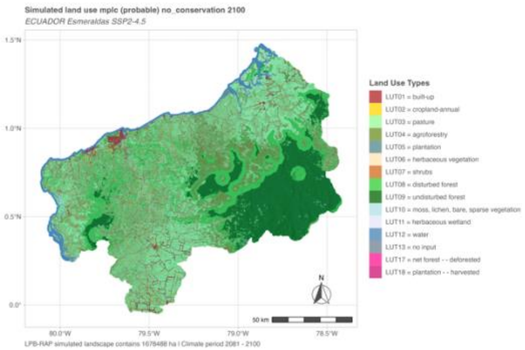

Figure D11: *Depicted are the spatial distributions of simulated land use change in LPB within the aggregation module mplc derived for the user-defined probing dates for each simulated policy scenario. Note that they depict a sequence on landscape level over all time steps, but that this interpretation can only be constrained applied to the singular pixel unit.*

1.3.3 LUTs development scenario comparison (panels)

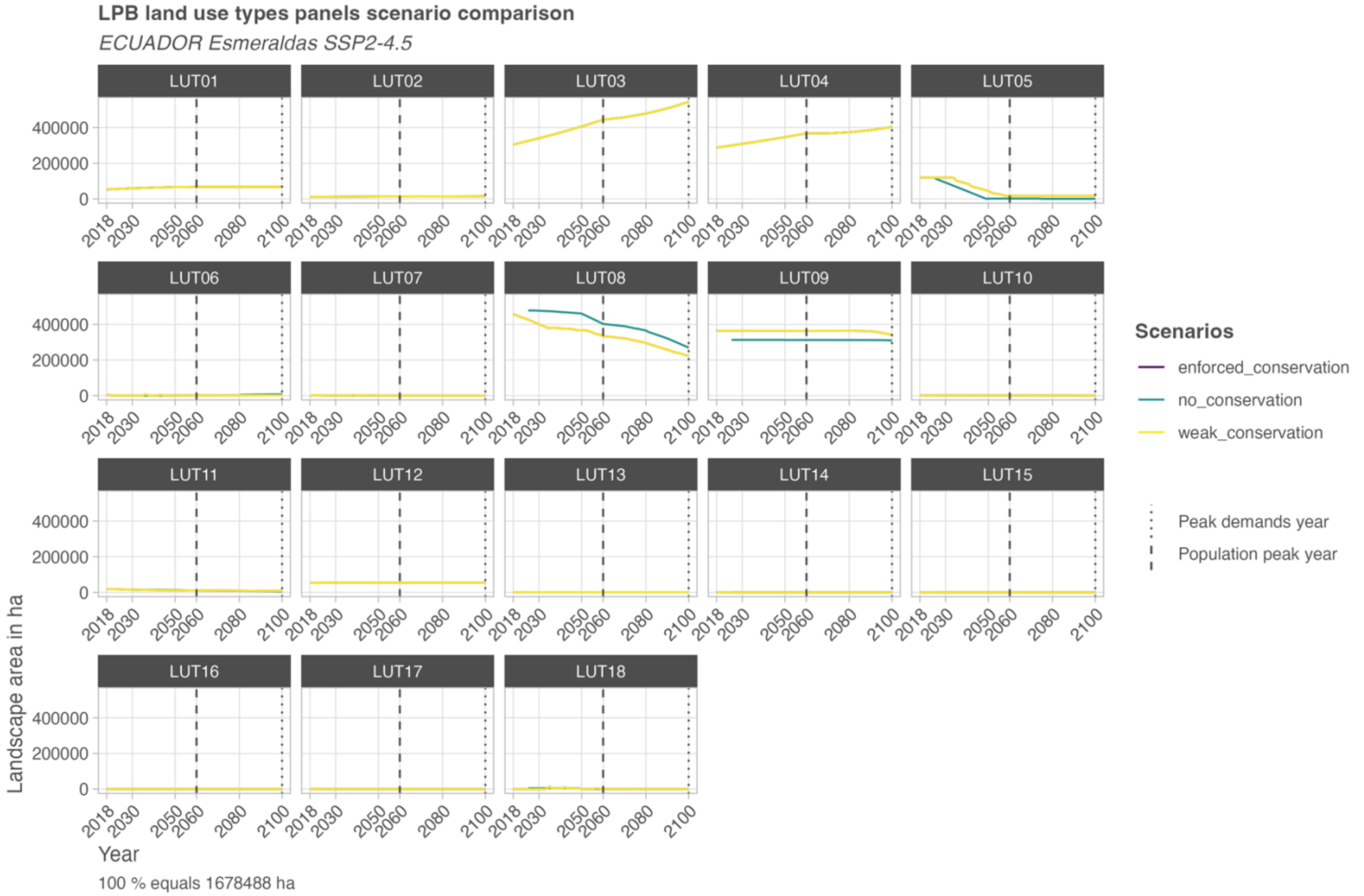

Figure D12: *The figure visualizes the simulated landscape area in ha per LUT as a scenario outcome. Note that in the case of the Esmeraldas region weak and enforced conservation behave identically despite different rulesets (enforced conservation thereby not visible). Slight differences can be seen between conservation scenario derivatives and the no conservation scenario: e.g. some variation in LUT05 = plantation and LUT18 = plantation harvested due to the stochastic age simulation. Minor differences due to the different spatial use of the landscape occur in LUTs 06 = herbaceous vegetation, 07 = shrubs, and 11 = herbaceous wetland. Most relevant is the visible different outcome for LUT08 = disturbed forest and LUT09 = undisturbed forest for the different use of the landscape. Marked are the probing dates according to the table D3 "Probing dates".*

1.4 LPB mplc pressure aspects

For the Esmeraldas settings, all derived pressure aspects are demonstrated in this section, predominantly in absolute numbers to show the magnitude. Note that here pressure on not protected forest extents is the most prominent.

1.4.1 Pressure on the population indicated by locally and regionally allocated demands

Locally allocated active land use types are evaluated by the distribution within the settlements draw area (see Appendix B section 1.2). All other pixels of the according land use type in the landscape are found outside this settlement's mean (for LaForeT: walking) distance radius and therefore imply greater cost by transport in time or monetary value for transportation mode for smallholders if situated within settlements.

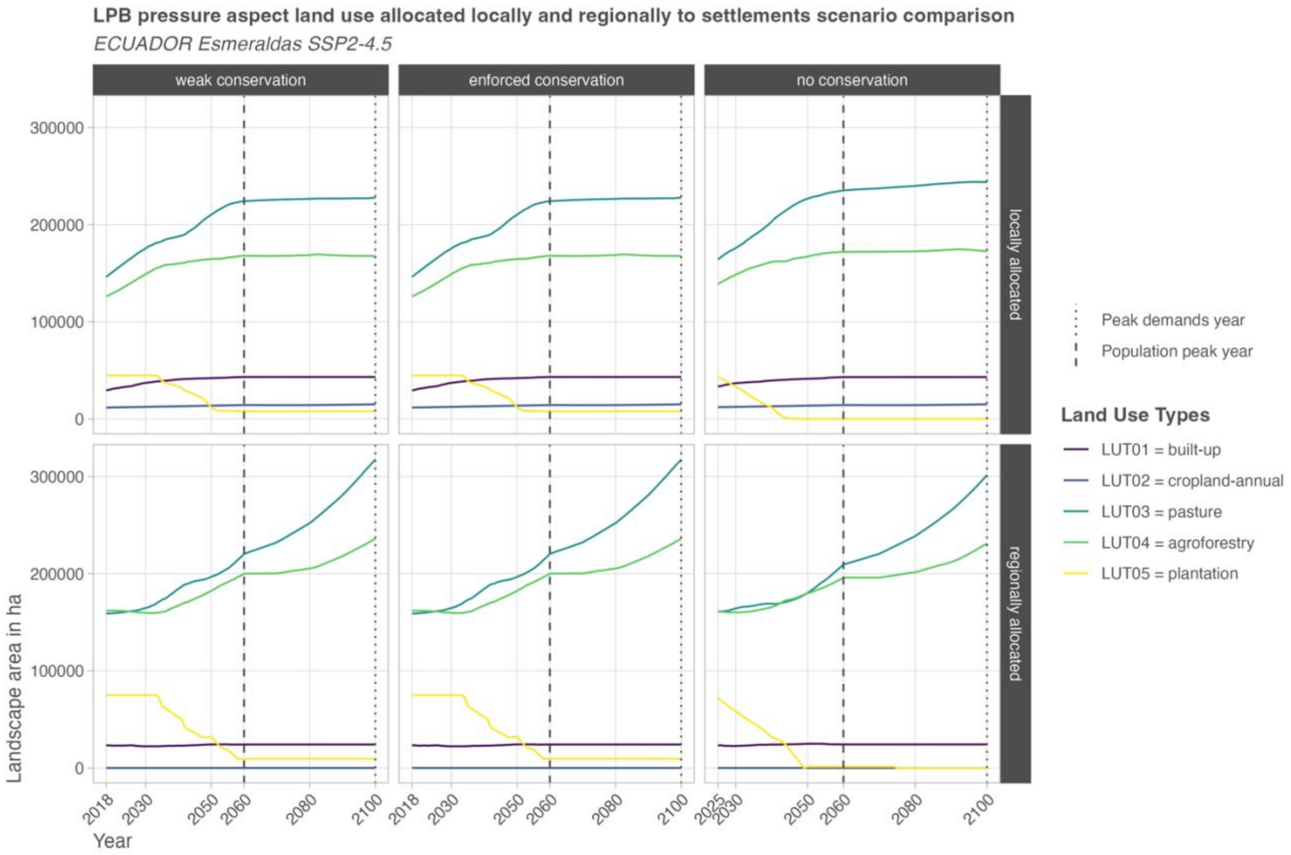

Figure D13: *The diagram depicts the effect of the applied user-defined allocation order and in the case of Esmeraldas points out the spatial relation of agroforestry and pasture to settlements to more remote areas, which is congruent to the notion of ground control points that agroforestry in the region indeed signifies often secondary forest characteristics. Note that locally allocated agricultural land use can only expand due to sinking plantation demand and forest conversion. Marked are the probing dates according to the table D3 "Probing dates".*

1.4.2 Pressure on the population indicated by use of difficult terrain and unallocated demand

The use of difficult terrain is theoretically diverging in all three scenarios due to the inherent variation of pressure: The weak conservation scenario requires use of difficult terrain if demand cannot be satisfied in favorable terrain outside restricted areas first, before simulating favorable terrain use and following difficult terrain use in restricted areas. The enforced conservation scenario excludes the restricted areas from simulation thereby demand overflow (unallocated demand) would theoretically indicate earlier that food security is no longer given for smallholders or trans-regional conflict over land resources may occur (this is not the case within the Esmeraldas simulation under the set scenario assumptions). The no conservation scenario setting implies the least pressure to use difficult terrain, since all favorable terrain is accessible first.

Unallocated demand per active land use type are output variables, but in case of the Esmeraldas forest landscape in all three scenarios 0. For the Esmeraldas region, the simulation does not produce actual unallocated demand, since the landscape's unprotected forest areas offer still enough evasion space. This variable would be populated, if the scenario of a general logging moratorium would be in place, i.e. if all forest plots would be protected, or if excluded areas were expanded, e.g. also by variation of terrain restrictions.

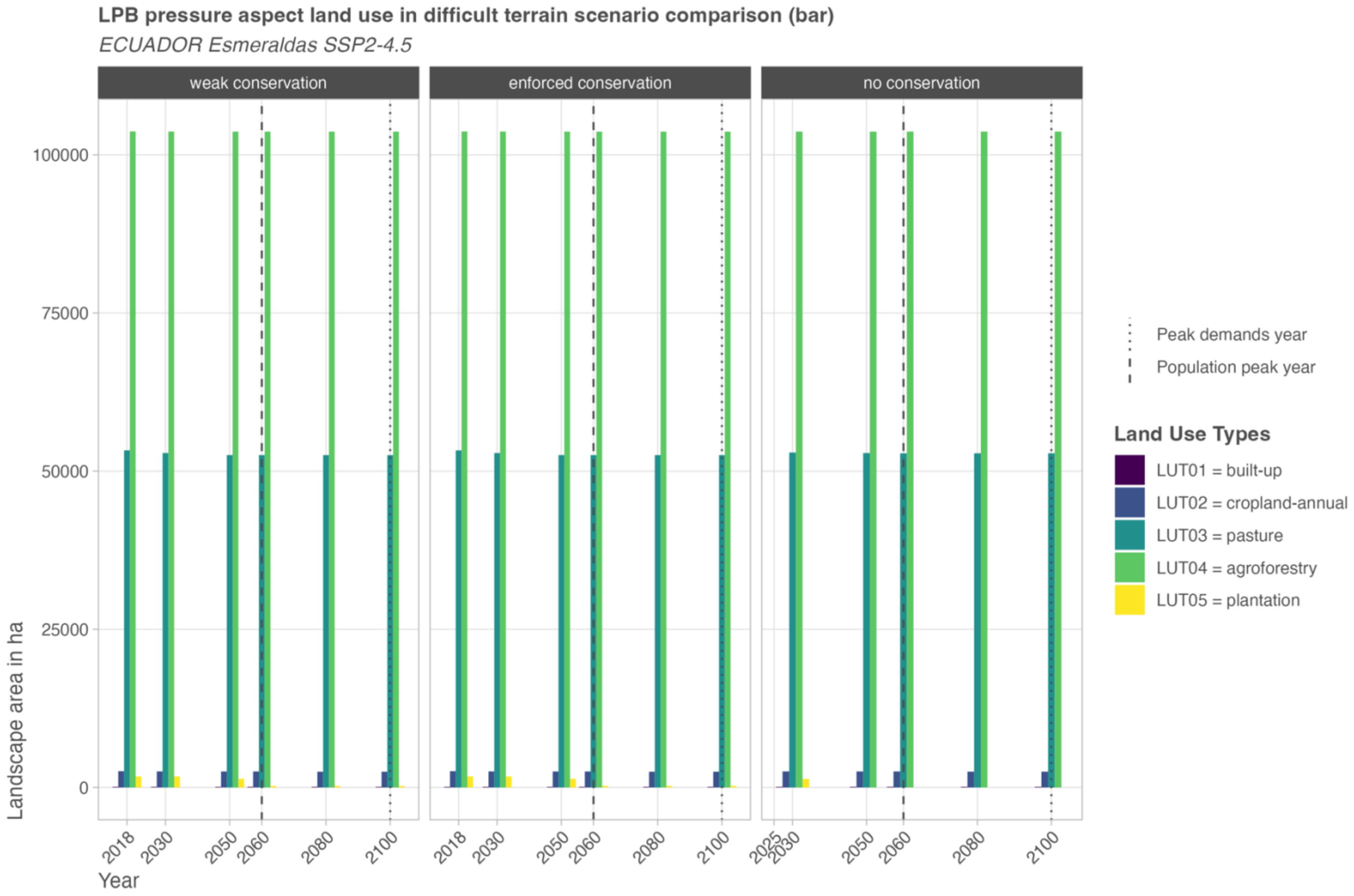

Figure D14: *In the case of Esmeraldas the conservation scenarios behave congruently despite slightly different rule sets. Note that difficult terrain (implying p.r.n. higher costs) needs to be used to satisfy demand within the scenario assumptions. Simulated are mostly initial LULC pixels.. Marked and visualized are the probing dates according to the table D3 "Probing dates".*

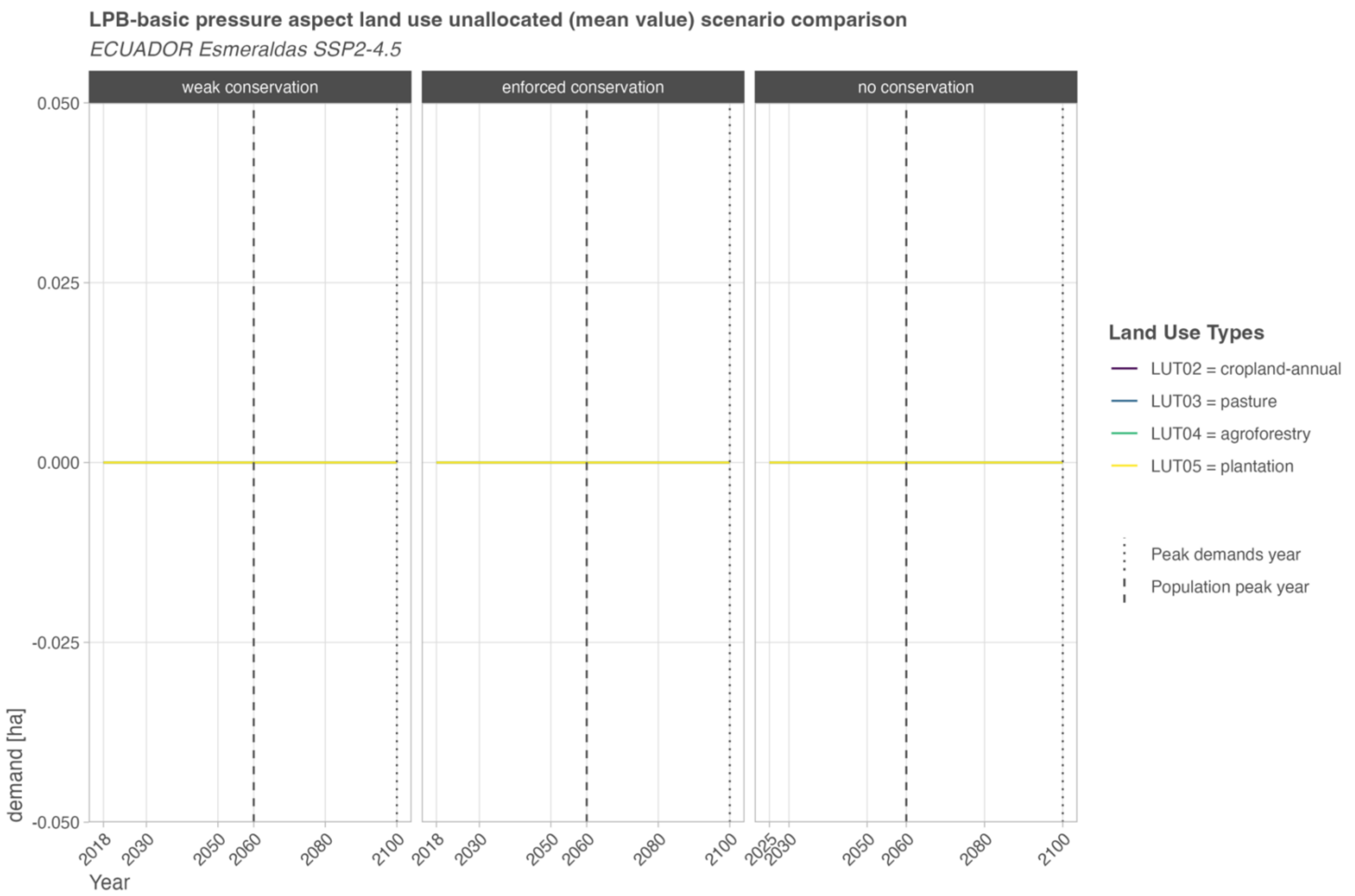

Figure D15: *This diagram visualizes the in-simulation unallocated demands as an indicator for threatened food security or possible spill-over effects etc. In the applied scenario assumptions, this is not the case. Marked are the probing dates according to the table D3 "Probing dates".*

1.4.3 Pressure on the population and pressure on restricted areas indicated by simulated use of restricted landscape extents

Only applicable in the weak conservation scenario for dynamic modelling. For comparison here all three scenarios are compared and showing the use of (current or formerly) restricted areas (as depicted by the initial LULC map or dynamically in the case of the no conservation scenario. The use of currently restricted areas depicts within model logic a land use conflict or especially forest land use conflict, if the pixel was forested in the prior time step. It is noteworthy that a large share of the currently restricted areas in the Esmeraldas province falls into areas with a larger slope and a large share of forest plots are not under protection, therefore parts of restricted areas are within this model logic not directly threatened by expanding land use. Note that existing land use is depicted in the diagram of accumulated land use, which we interpreted as sanctioned land use. A decline of accumulated pixels is partially visible and results from declining demand in combination with suitability. Formerly restricted areas land use in the worst-case scenario is largely associated with existing and dynamic settlements and the static streets distribution, which could not be simulated dynamically until now.

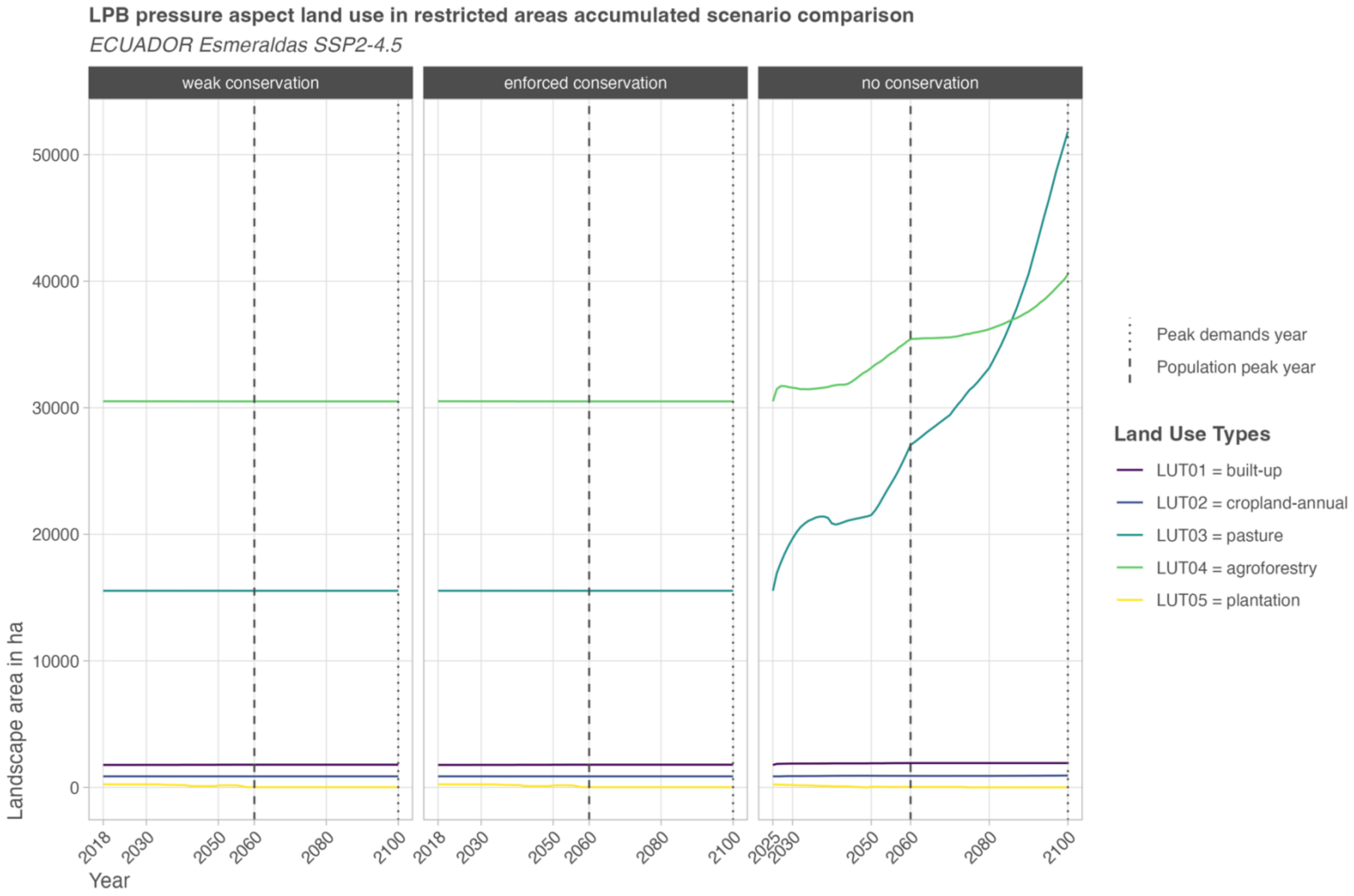

Figure D16: *Visible are the trends of accumulated land use in between the forest policy scenarios in current or former restricted areas. Note that initial pixels rely on remote sensing applications. The use of former restricted areas based on terrain factors in the no conservation scenario is related to existing as well as new dynamically simulated settlements and the existing street network. Marked are the probing dates according to the table D3 "Probing dates".*

1.4.4 Pressure on restricted areas: land use conflict and forest land use conflict

Per definition in this model conflict can only occur for restricted areas. Since we assume that all further use and logging is prohibited (initial pixels may be relics with a sanctioned status), new land use and especially new land use on former forest pixels here describes a conflict of interest (therefore only in weak conservation describable). For the no conservation scenario note that this evaluation takes place on the former restricted areas to display potential impact on such areas.

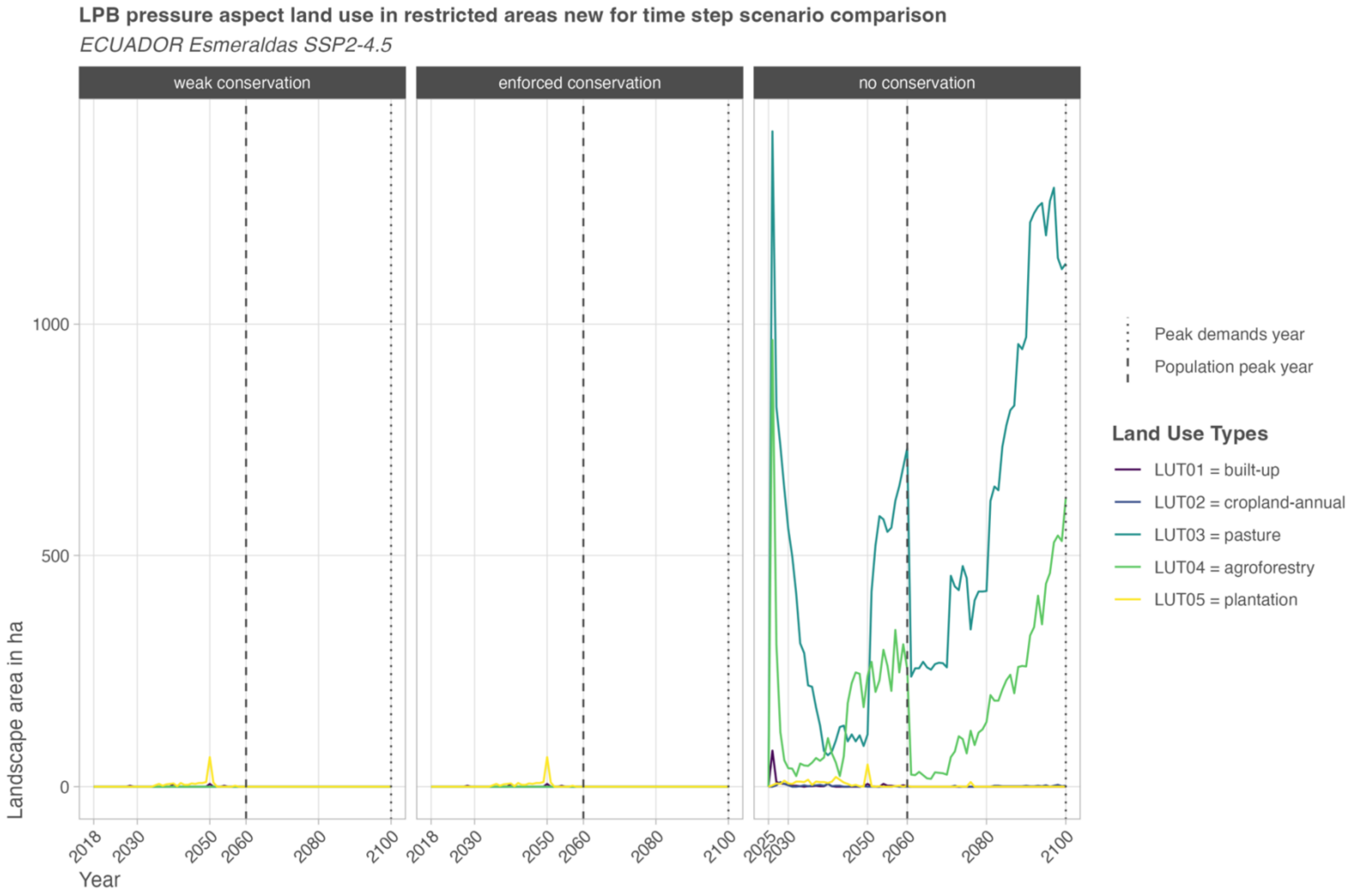

Figure D17: *The delayed or excluded land use of restricted areas in the weak conservation scenario is in the case of Esmeraldas due to the provided unprotected forest sites evasion space marginal. For the conservation) scenarios only the -based on population data- simulated new settlements as singular ha pixels change the landscape configuration as well as re-assignment of singular harvested plantation pixels. For the no conservation simulation based on the assumption of an ensued repeal development of the use of the available space in favorable terrain indicates immediate increased use if policy measures would be repealed. Marked are the probing dates according to the table D3 "Probing dates".*

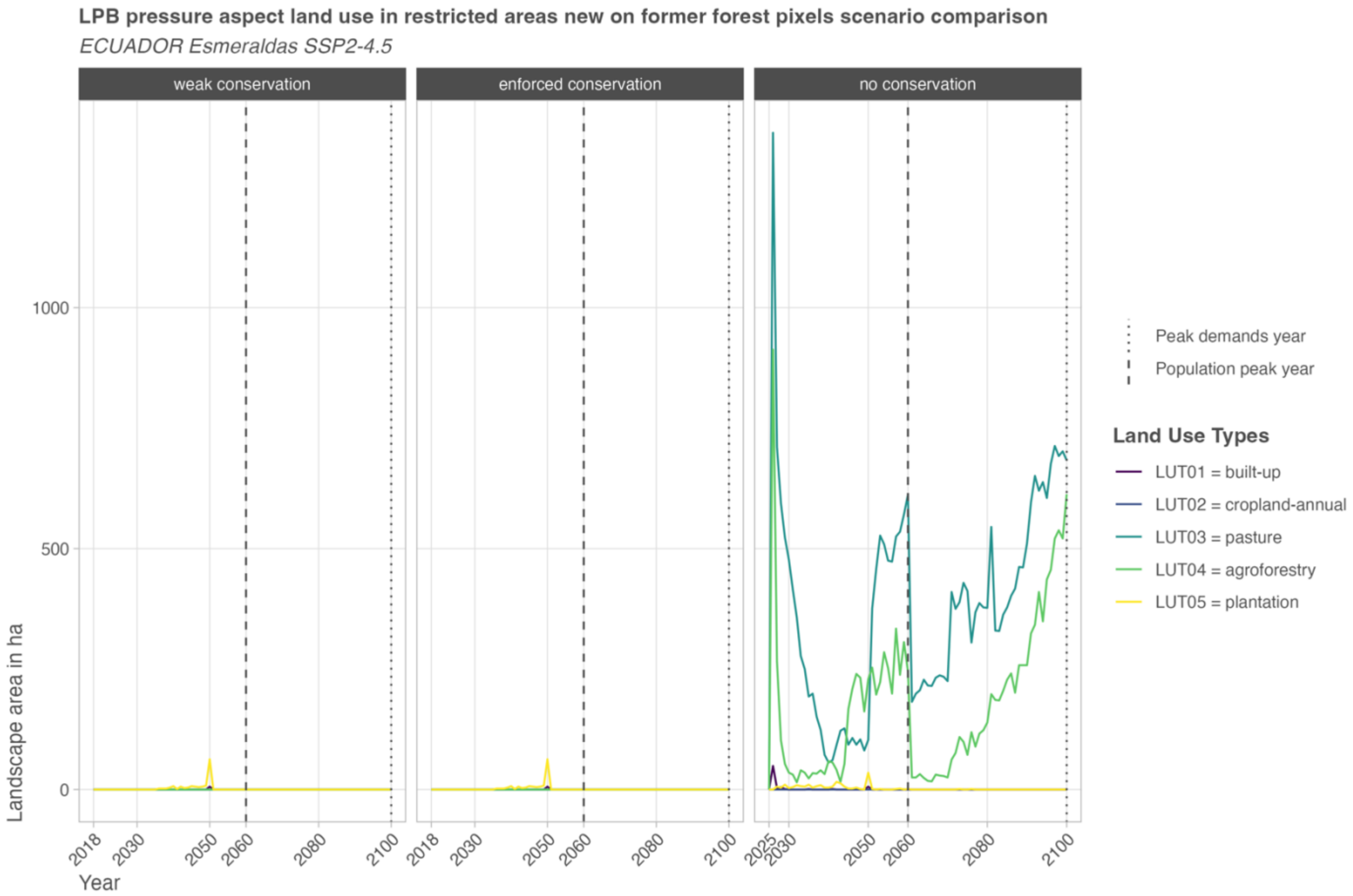

Figure D18: *The analysis shows that a significant share of new land use is allocated to former forest pixels, i.e., nearly exclusively agroforestry and pasture. This could indeed represent the circumstances or may overestimate the transformation if initial (remote sensing-based) information on land use and anthropogenic features is missing for the correction step. Marked are the probing dates according to the table D3 "Probing dates".*

1.4.5 Pressure on forest by deforestation, conversion and degradation of quality

Within the forest landscape, the deterministic and land use simulation-based demand implies overall similar trajectories of amounts of forest degradation and conversion for all three policy scenarios since growing demand can only be satisfied by forest pixel transformation within model logic/forest landscapes. The difference is here to be found in the spatial distribution of remaining, especially undisturbed, forest, based on the simulated use of the available landscape extents.

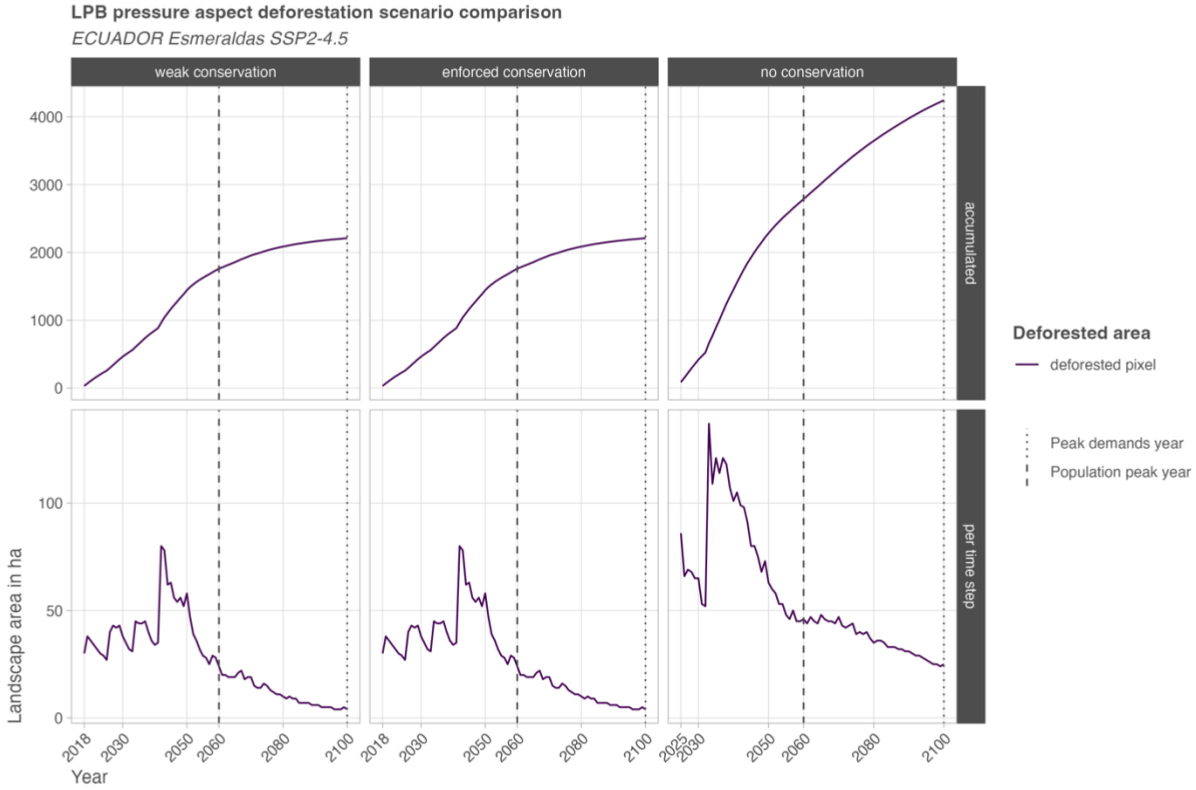

Figure D19: *The diagram shows solely the development of allocation for LUT17 = net forest - - deforested (which in this simulation only depicts the approximated fuelwood demand by the entire regional population, since for the Esmeraldas region neither commercial nor subsistence timber demand is known and charcoal production is not a factor), signifying deforested forest pixels remaining after land conversion, whose further trajectory is not decided in the time step. Simulated in mplc is the mean value of all remaining deforested pixels after allocation of other land use types of all samples in basic per time step. Note that this symbolizes the urban wood demand share in this study design. Note also, that the area allocated sinks with decreasing demand per time step. The no conservation scenario differs in magnitude due to spatial allocation; here restricted areas are not excluded in the first loop of deforestation allocation and the land use pattern results in a different net forest fringe spatially, containing other AGB amounts to satisfy the same demand. Marked are the probing dates according to the table D3 "Probing dates".*

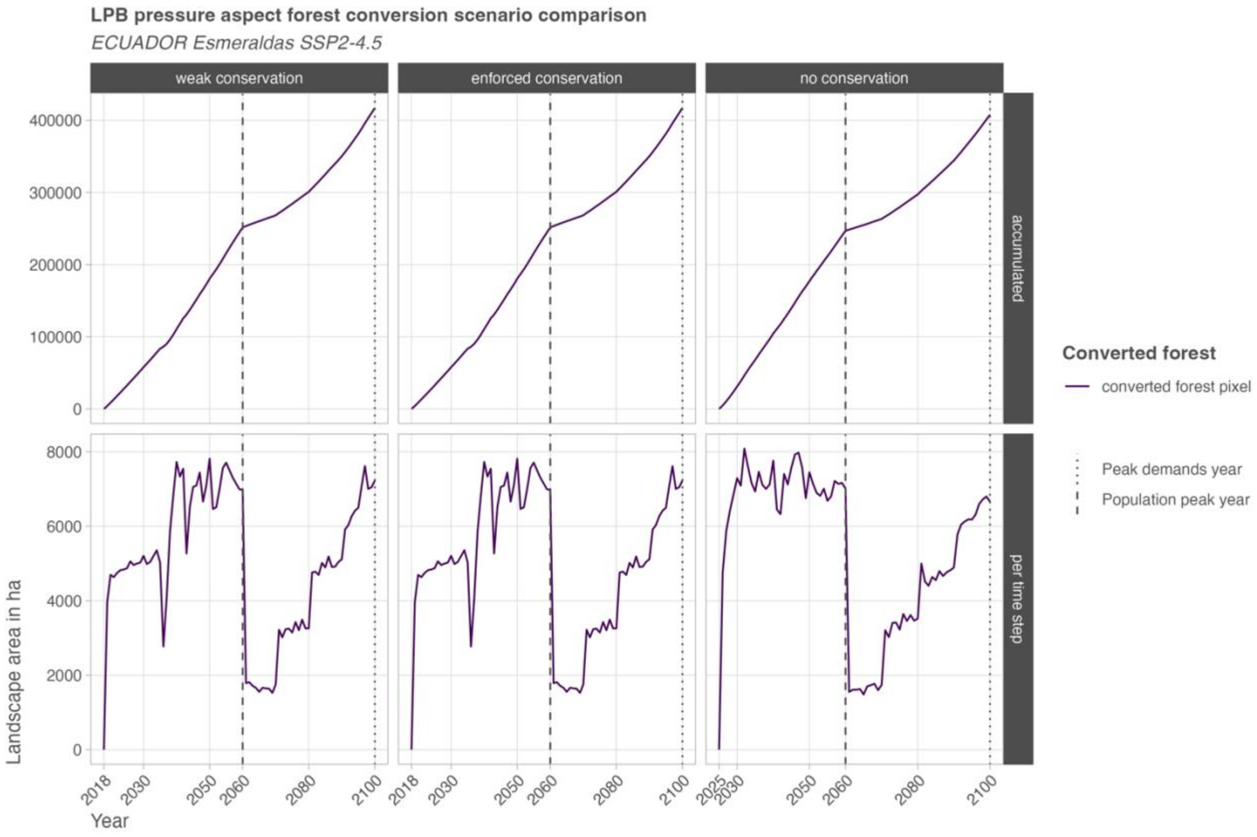

Figure D20: *The diagram visualizes solely the conversion of forest pixels to the combined primary active land use types 01 to 05 with area demands as derived in mplc, this can only be constrained viewed as sequence since no dynamic modeling is conducted and the results rely on aggregated landscapes per time step. The no conservation scenario differs due to the stochastic succession and plantation and different landscape use, which for example denotes a higher use of herbaceous wetland pixels. Marked are the probing dates according to the table D3 "Probing dates". For a depiction of the singular land use types shares divided in the simulation within LULCC_basic and LULCC_mplc see the next figures.*

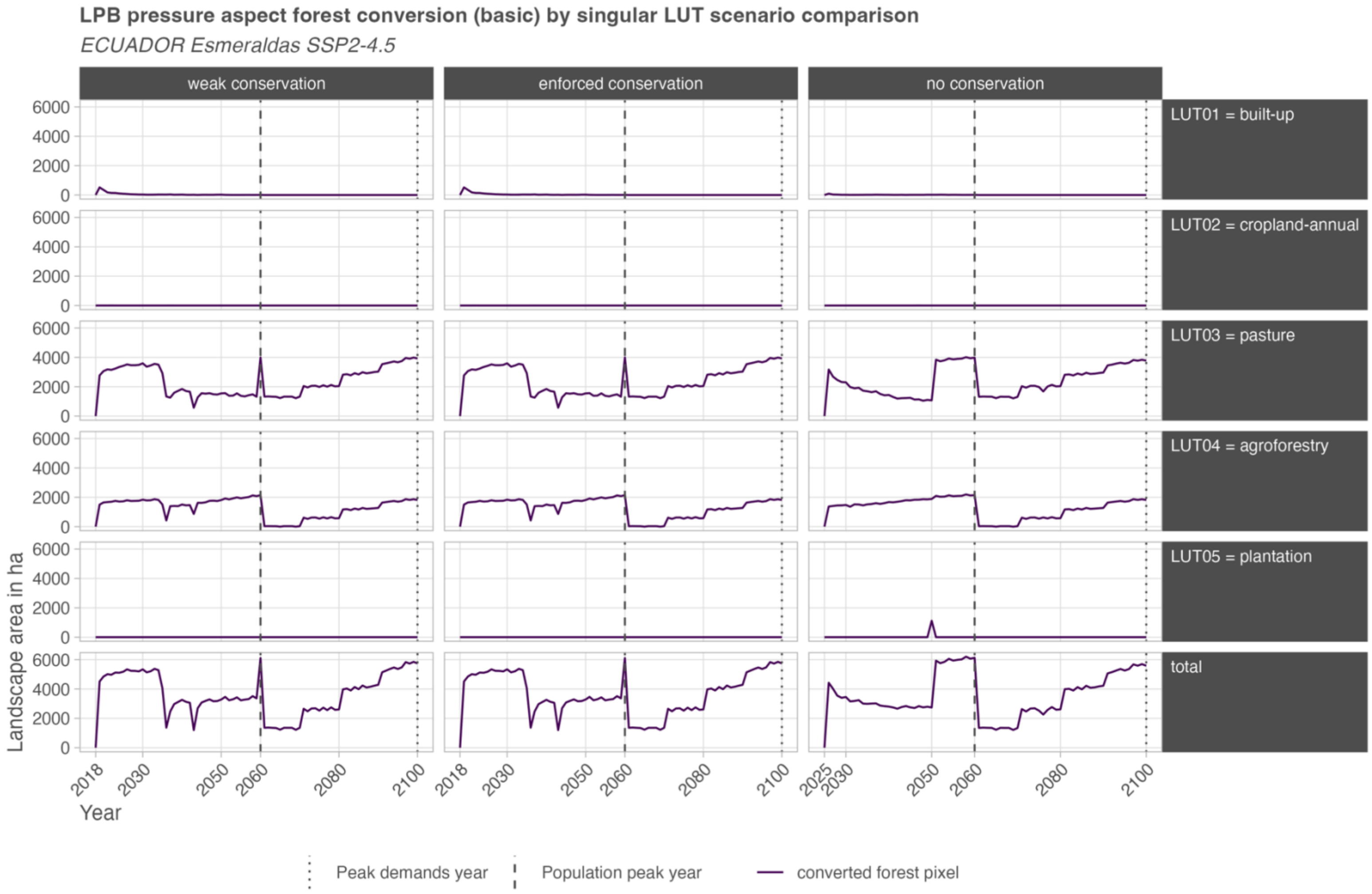

Figure D21: *The diagram depicts forest conversion per time step per active land use type and in total as simulated within the probabilistic basic module (aggregation value is the maximum over all samples). Marked are the probing dates according to the table D3 "Probing dates".*

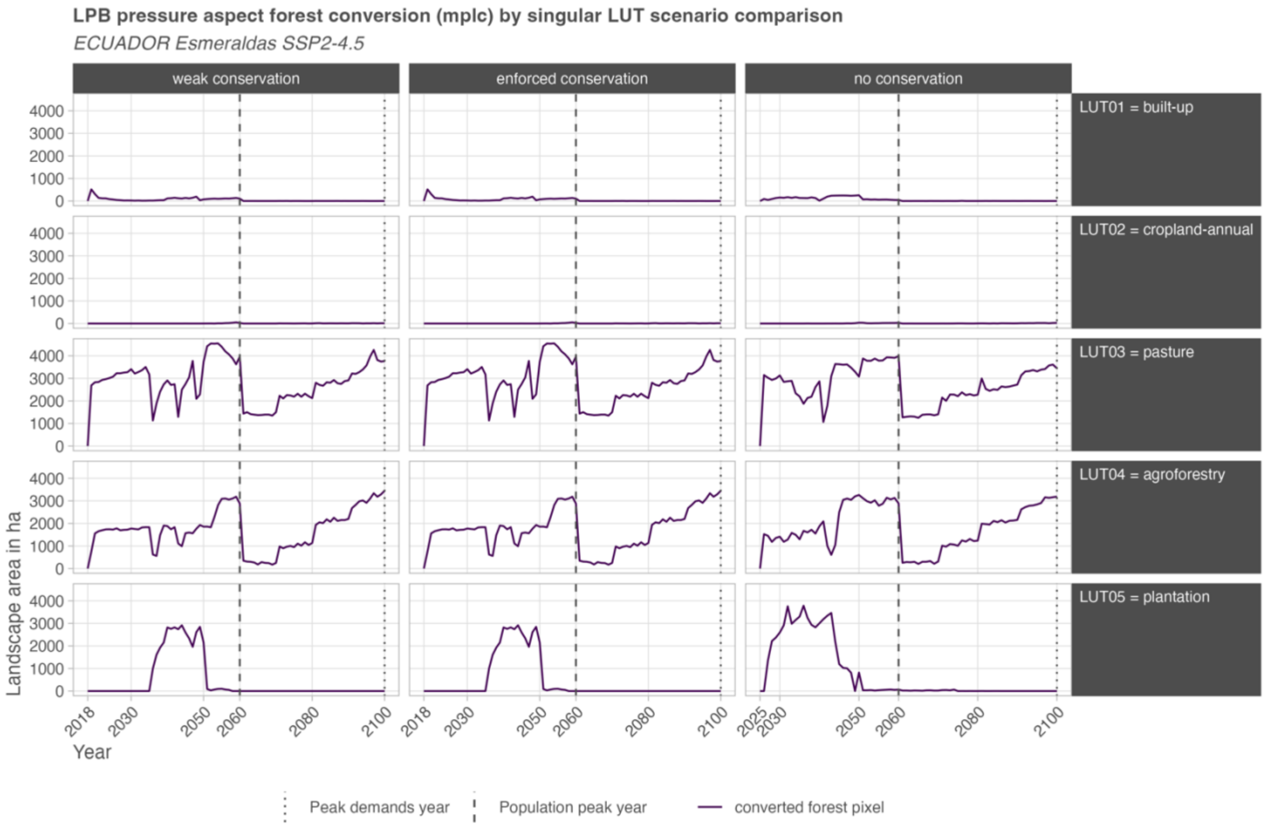

Figure D22: *Depicted is the conversion per time step per active land use type as derived in mplc. A difference can be seen between the conservation scenarios and the no conservation scenario due to 1) area availability steered by simulation of use of (current or former) restricted areas and 2) plantations, which is primarily based on the remote sensing data for the conservation scenarios but solved stochastically in the no conservation scenario. The here used TMF data accumulates plantation deforestation not per year but by selected dates, which accounts for major peaks in harvesting (one mean plantation rotation period applied), although within the model the earliest accumulating remote sensing-based date is already varied stochastically by use of the user-defined plantation rotation period. Marked are the probing dates according to the table D3 "Probing dates".*

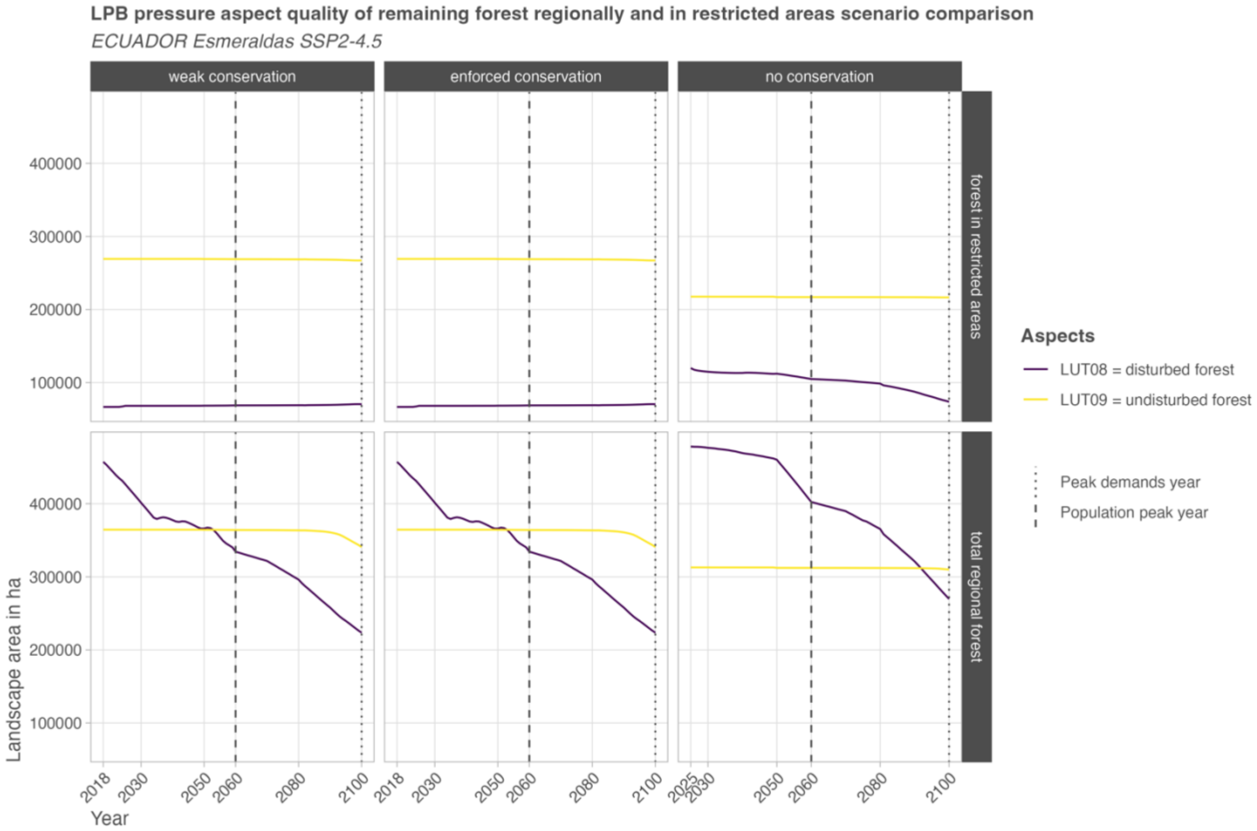

Figure D23: *Visible are steady trends in all three scenarios with a decline in area of forest towards the peak demands year (2100). While undisturbed forest stays relatively stable, decline is simulated for the disturbed forest simulated. This is to be explained based on the simulation of disturbed forest for each pixel affected by the anthropogenic impact distance as well as simulated wood extraction around settlements. For the larger portion of the simulation expanding land use exapands into these areas. Marked are the probing dates according to the table D3 “Probing dates”.*

Probing date	Weak conservation	Enforced conservation	No conservation
1 (2018/2025)	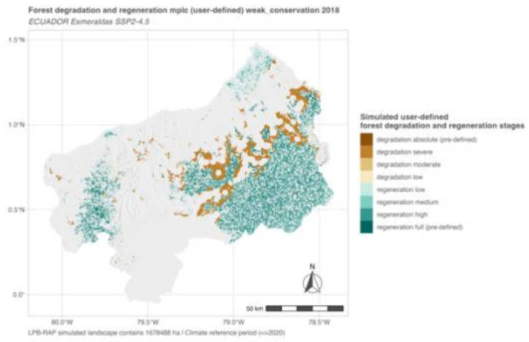	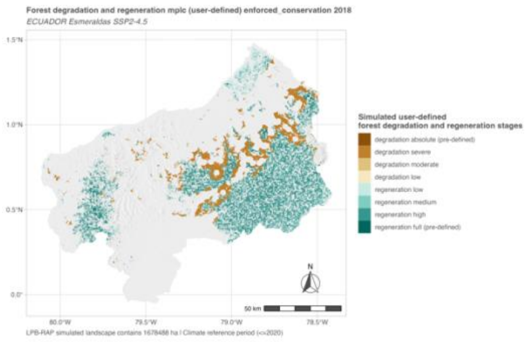	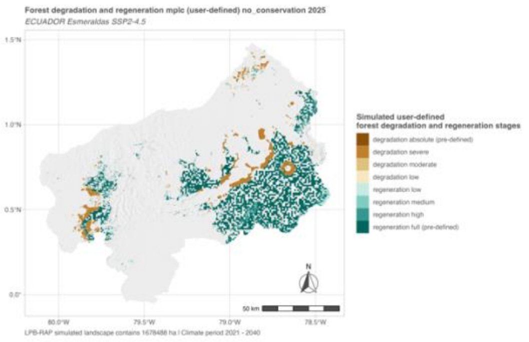
2 (2030)	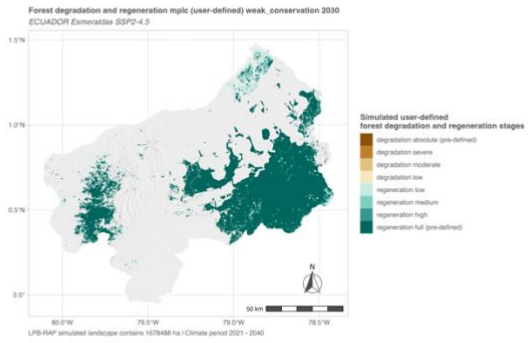	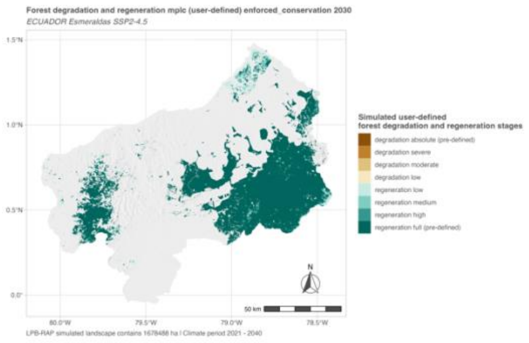	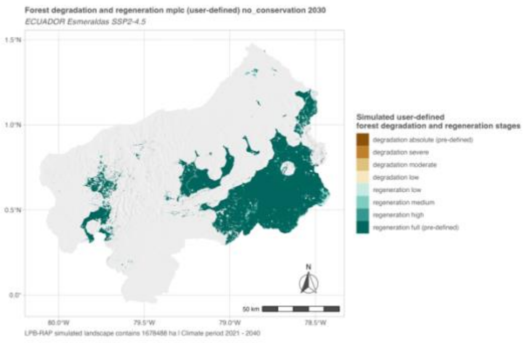
3 (2050)	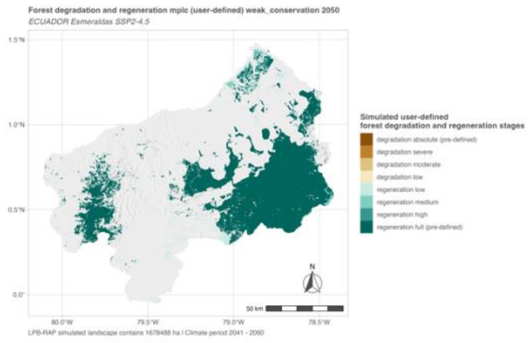	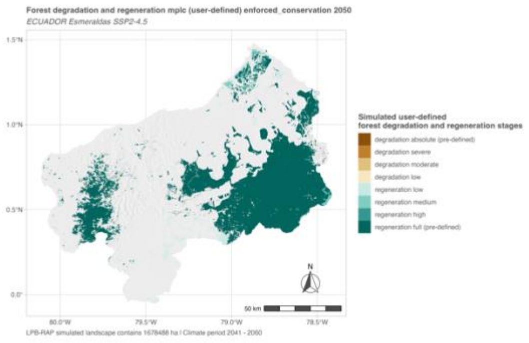	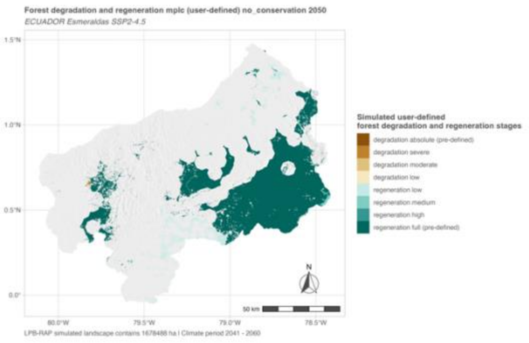

4 (2060, pp)

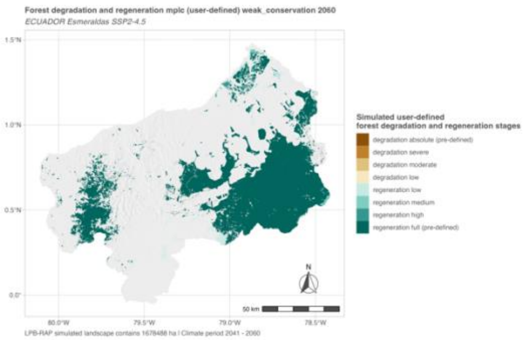

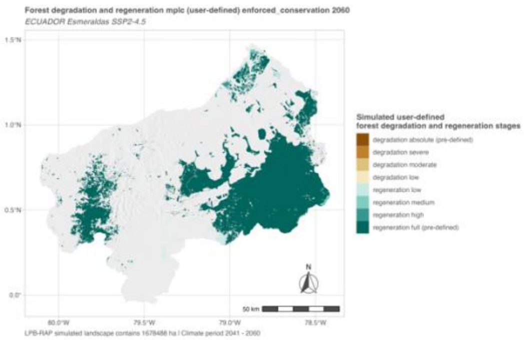

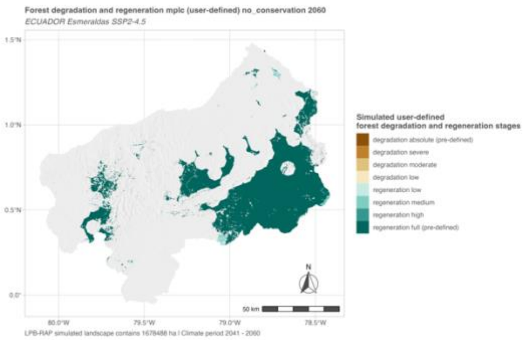

5 (2080)

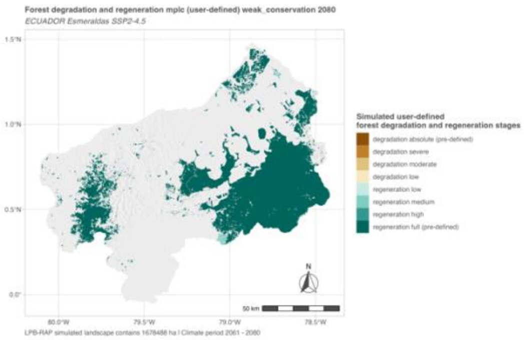

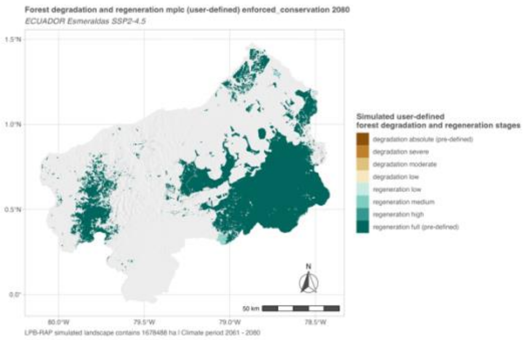

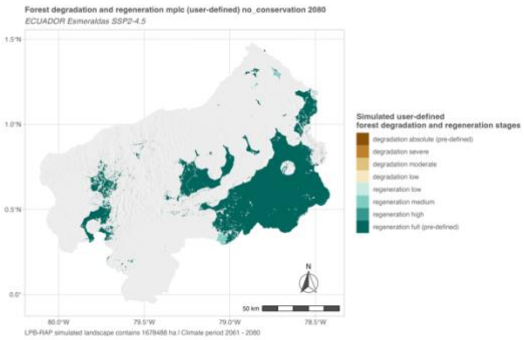

6 (2100, pd)

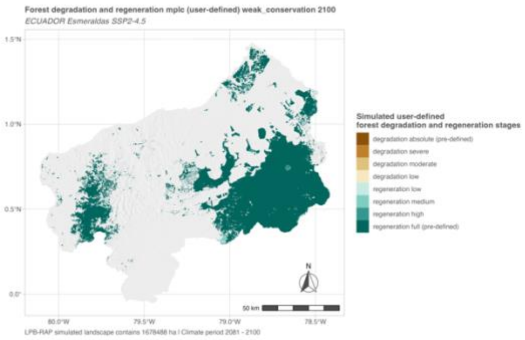

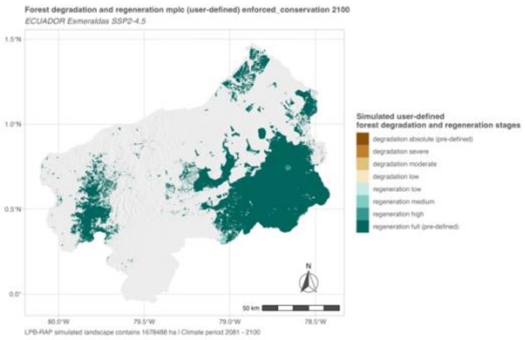

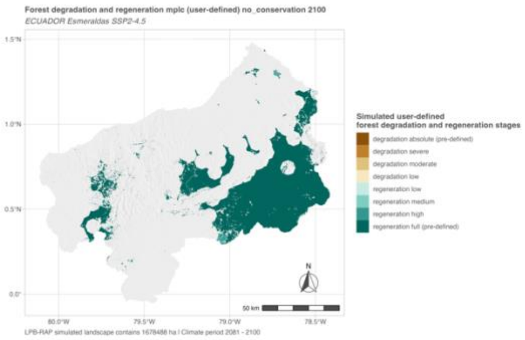

Figure D24: *Visualized are the user-defined degradation (browns) and regeneration classes (greens) as derived per time step. Note that within the simulation the initial time step displays the largest degradation, as it refers to the initial AGB map. For the later time steps degradation occurs only in minor quantities in the case of Esmeraldas, as forest pixels are converted to anthropogenic land use and thereby not counted any longer. In contrast, remaining forest is mainly simulated as in a state of regeneration due to the low and steadily declining wood demands that furthermore only distributed in fractions within the local wood extraction distance. However, the forest area in total declines gradually. Note the difference between the conservation scenarios and the no conservation scenario in use of restricted areas and thereby different emerging patterns of remaining forest.*

2 RAP results (possible within mplc) potential FLR scenario

The provided results are produced in the finishing module and depict the possible landscape configuration of to terrestrial surface level approximated land use under a restoration paradigm, i.e., the at maximum applicable measures within the scenario assumptions based simulated most probable landscape configuration (mplc module output serves as input). This means, potentially more remote sensing-based tree or forest cover could be in the landscape depending on the applied algorithm/percentage threshold (e.g., derived from singular trees within gardens or street adjacent etc.), but here the potential for forest land use site characteristics at terrestrial surface level is approximated. Note that RAP only depicts areas, that require action to be reinstated in functionality, the according conservation potential is implied in the remaining landscape area of disturbed and undisturbed forest pixels or other ecosystem forest pixels, which do not serve demands. Therefore, RAP increases with increasing land use. RAP is also simulated for the no conservation scenario, which in that case would require bottom-up actors alone to transform the landscape. No diverging simulation uncertainty is provided, since the algorithms base directly on the mplc results and which determine thereby the greatest probability for RAP. Furthermore, RAP depicts the potential entry into FLR for each time step, i.e. it assumes for each time step that FLR has not been implemented landscape wide and can therefore not be seen as a sequence.
In the case of the Esmeraldas province RAP can be interpreted as an increase in forest site characteristics and forest cover from 0 % (deforested plots) or for the general landscape with a large share for an increase above 15 % tree cover (landscape coverage according to Copernicus map) in regard to forest biome pixels. For other ecosystem pixels we currently have no information other than initial passive land use types in the landscape. For RAP-LUT25 we can only simulated a user-defined critical AGB content in percent, for which measures in replanting trees are required to gain AGB towards maximum potential AGB.

2.1 RAP targeted net forest

To make the connection to national data, targeted net forest is simulated directly on the initial net forest extent (national forest dataset) without alterations made by the correction step or time step 1 simulation in both conservation scenarios. Furthermore, net forest in the modeling contexts indicates potential only for forest that is not under management in any form (neither potentially fenced agricultural use nor forest management including harvesting) with the goal of a quasi-primary forest status for the climax stadium indicating potentially connected habitat without barriers at terrestrial surface level. Therefore, RAP plantation is not acknowledged for the calculation of possible net forest including succession and reforestation.
The map comparison shows the simulated targeted net forest area (existing net forest plus increment) per scenario, which is the basis for the RAP algorithm to apply the RAP LUTs “RAP reforestation” (within this area on forest biome pixels where no other ecosystem is indicated) and “RAP plantation” (outside this area on forest biome pixels where no other ecosystem is indicated). For Esmeraldas, the simulation indicates that the set target increment cannot be reached by succession and reforestation of deforested plots alone, not even after the population peak until 2100, if only disturbed and undisturbed pixels are accounted for and land use is persistent within the scenario assumptions.

Weak conservation / Enforced Conservation 2018	No conservation 2025
3 % target	7.38 % target
user-defined	calculated based on areas lost in correction step and dynamic simulation for the targeted no conservation initial simulation year within prior weak conservation run, incorporating the initial targeted increment
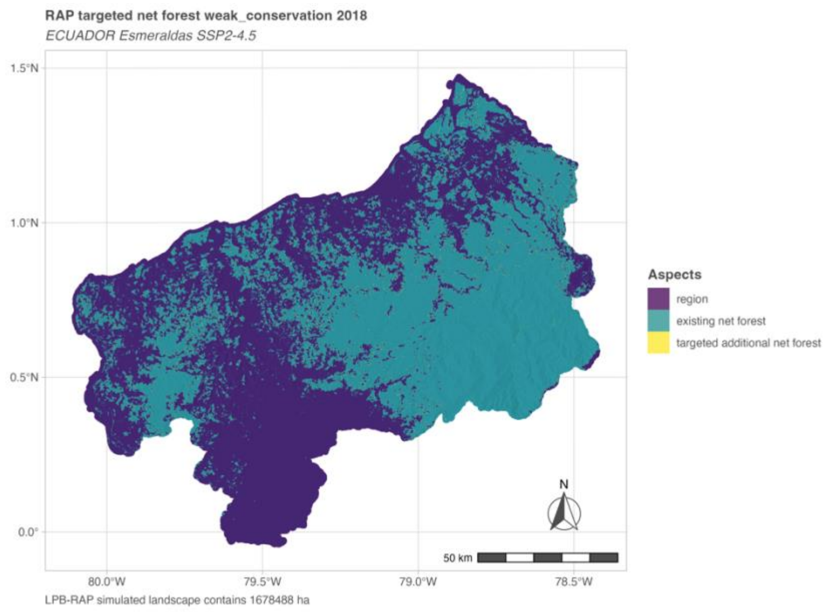	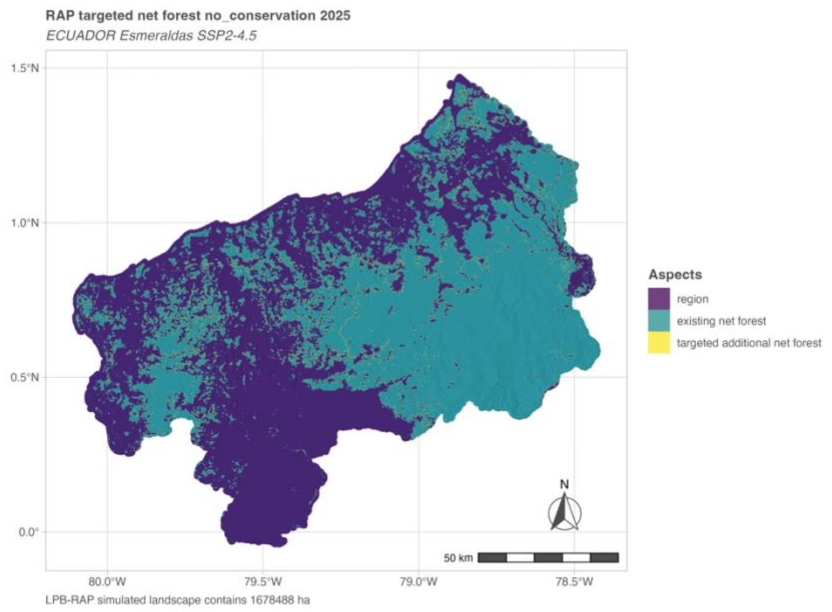

Figure D1: *The depicted maps show the calculated targeted net forest per scenario, that is in the current design an equal result for the conservation scenarios and the diverging result for the no conservation scenario. Note that for the no conservation scenario the weak conservation scenario 2025 output of initial net forest is used and therefore the applied algorithm indicates other areas as most suitable for the to be realized targeted increment in forest area.*

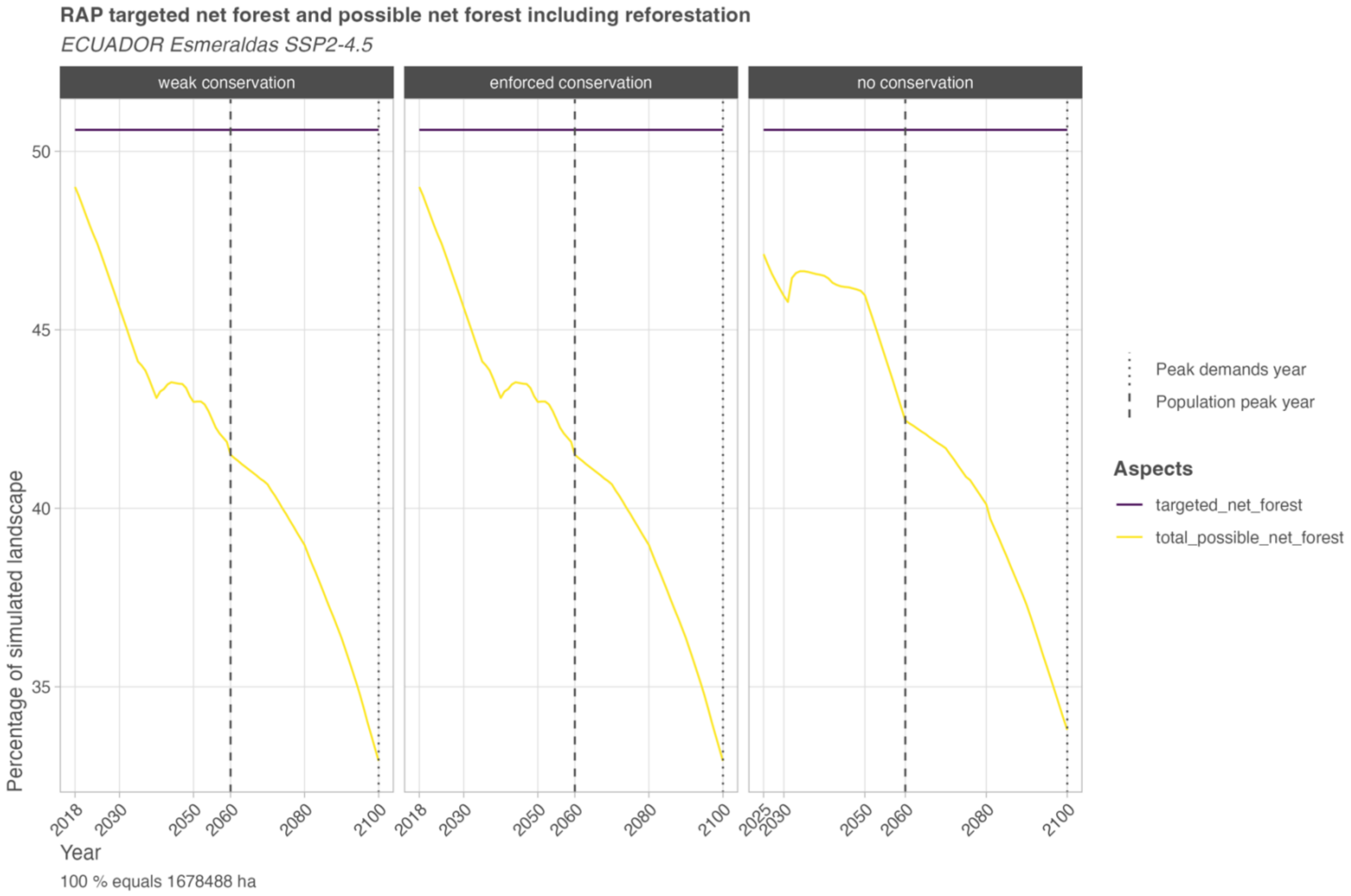

Figure D2: *The diagram visualizes firstly the discrepancy between an increment goal projected on forest cover and actual forest conditions at the terrestrial surface level in the gap for the initial starting year between the two variables. Secondly, it depicts the development of simulated possible net forest, i.e., the dynamically simulated net forest extent solely based on a) neighborhood operations of undisturbed and disturbed forest and succession as well as b) accounting for reforestation pixels. Note that the no conservation scenario depicts a slightly higher amount of forest. Marked are the probing dates according to the table D3 "Probing dates".*

2.2 RAP maximum - land use types shares and landscape configuration

The simulated available area for restoration for each year is a potential Forest Landscape Restoration (FLR) starting point (i.e., under the assumption that no restoration has been implemented landscape-wide so far). Due to deterministic demand these are similar in all three scenarios, but vary slightly in spatial distribution due to per scenario available areas in the cascading (corrective) allocation. Note that the greatest potential lies according to model logic for the Esmeraldas in the transformation of conventional farming systems (LUT21, which is an aggregation LUT for LUT02 = cropland-annual and LUT03 = pasture). LUT21 refers in the case of Esmeraldas to agroforestry systems that reach tree cover >15 % per ha, since this status is almost everywhere currently already achieved according to Copernicus imagery.

2.2.1 RAP total of formerly converted pixels (RAP-LUTs 21 to 24)

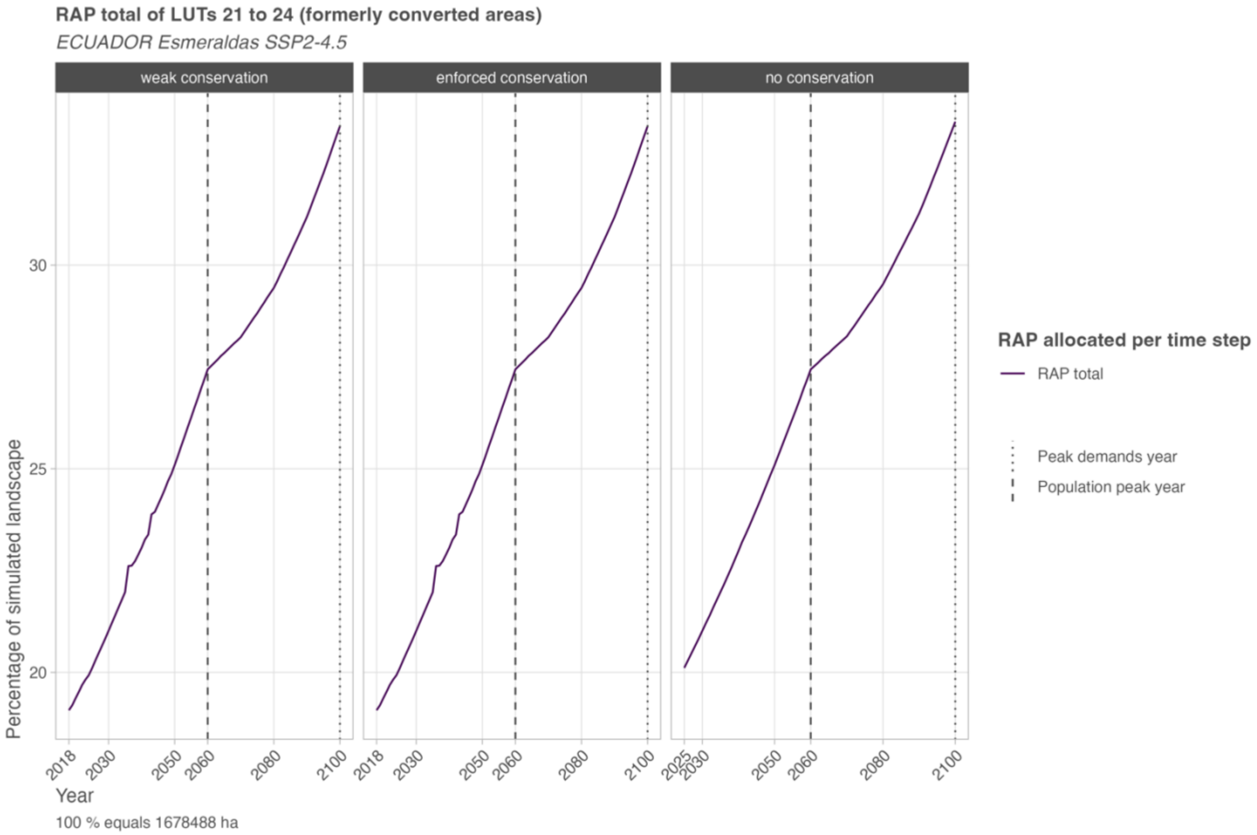

Figure D3: *Note that RAP does not depict a sequence of development (therefore it cannot be accumulated) but treats each year as a potential starting point for landscape-wide FLR implementation under the assumption that the same has not occurred until this point. The diagram shows that RAP total within the scenario assumptions and settings is mainly reliant on the transformation of conventional farming systems. Note that RAP signifies a planning instrument, which is why here only the areas that require action are depicted. Conservation is implied in the remaining forest landscape and landscape mosaic sites. Marked are the probing dates according to the table D3 "Probing dates".*

2.2.2 RAP of formerly degraded forest pixels (RAP-LUT25)

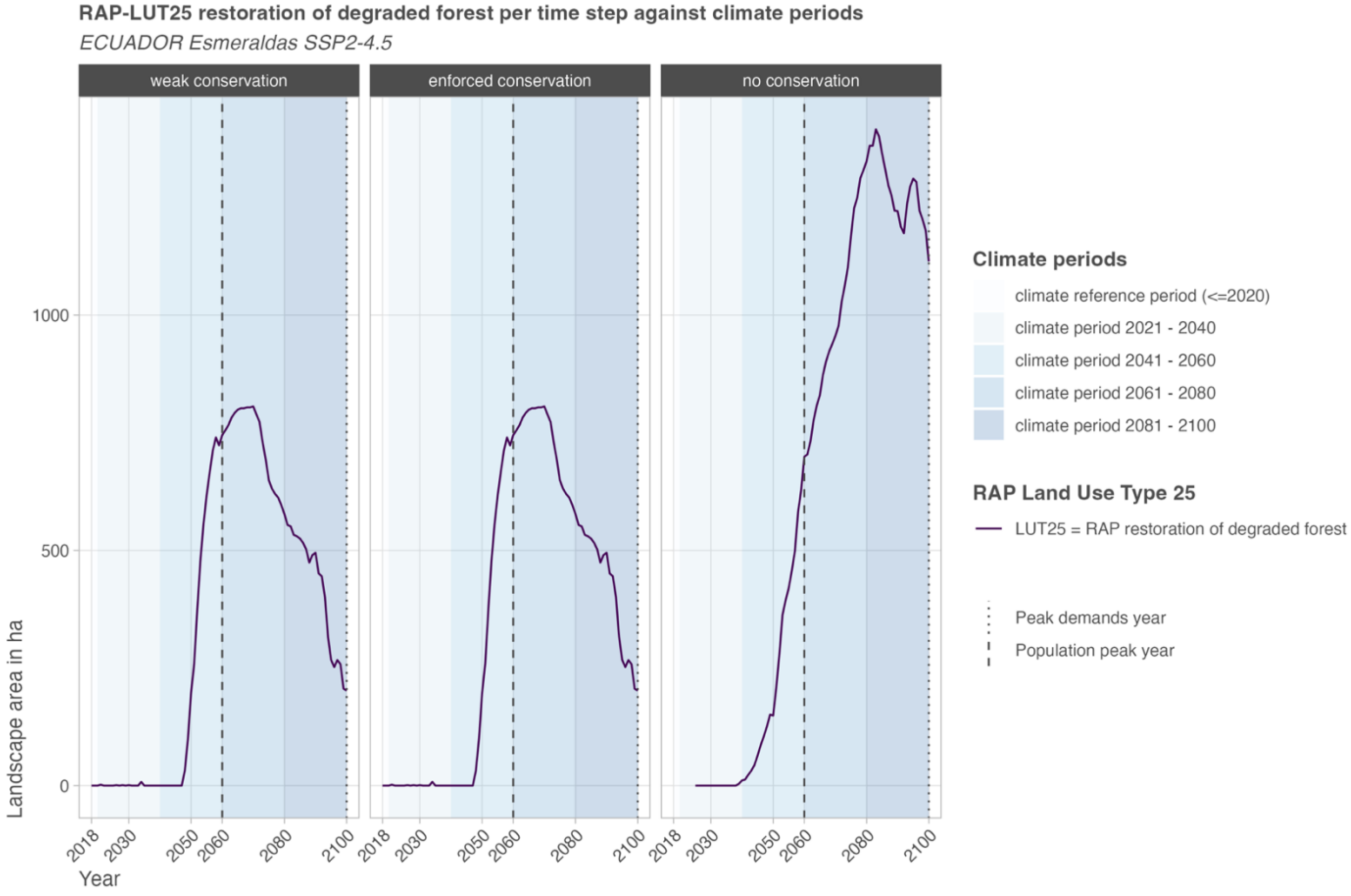

Figure D28: *Note that RAP-LUT25 is rather complex in simulation (see Appendix A Prelude) and. p.r.n. a strong influence of climate periods information can be visible. In this simulation this is only visible for the no conservation scenario. Marked are the probing dates according to the table D3 "Probing dates".*

2.2.3 RAP numerical time series

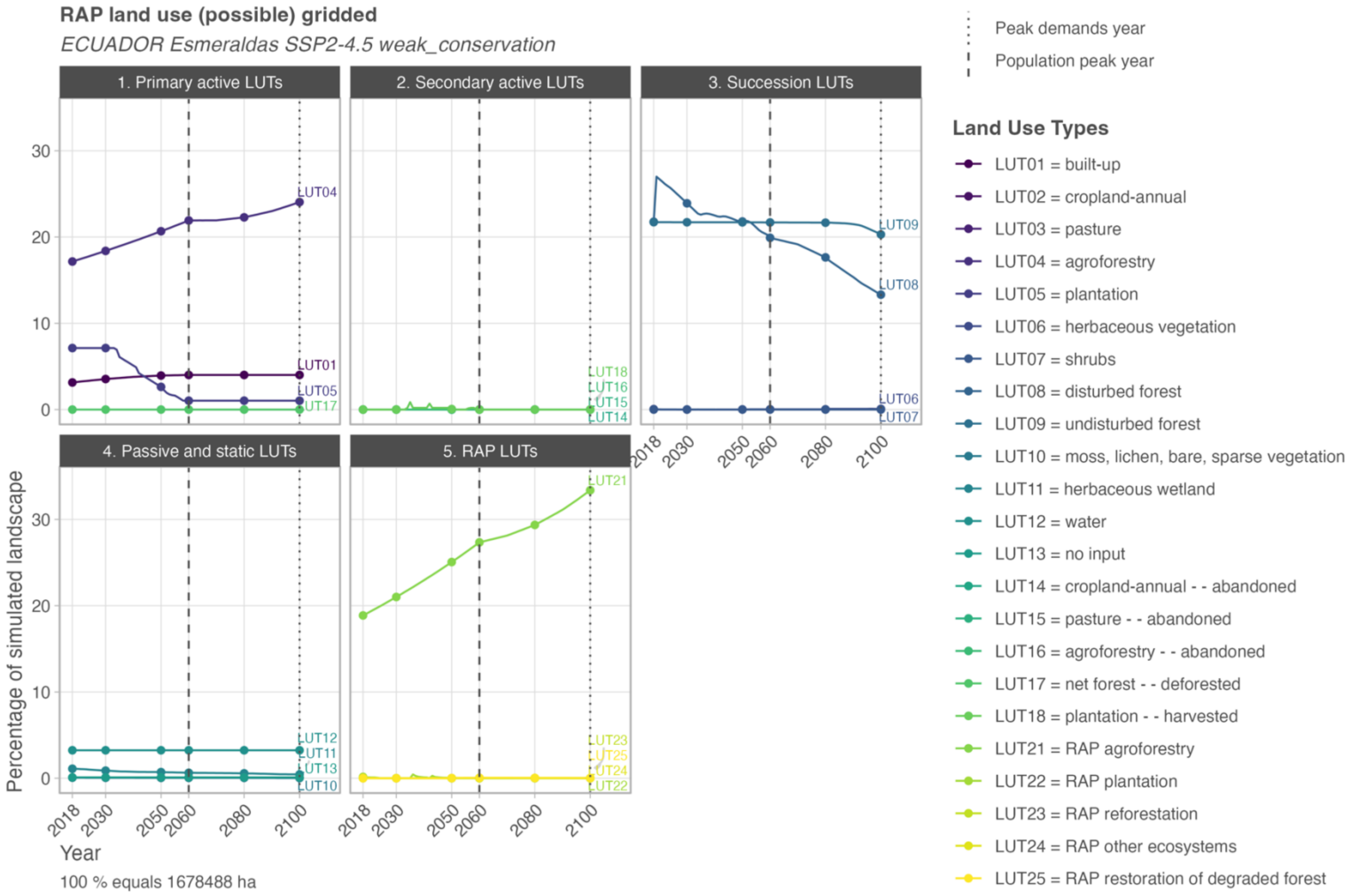

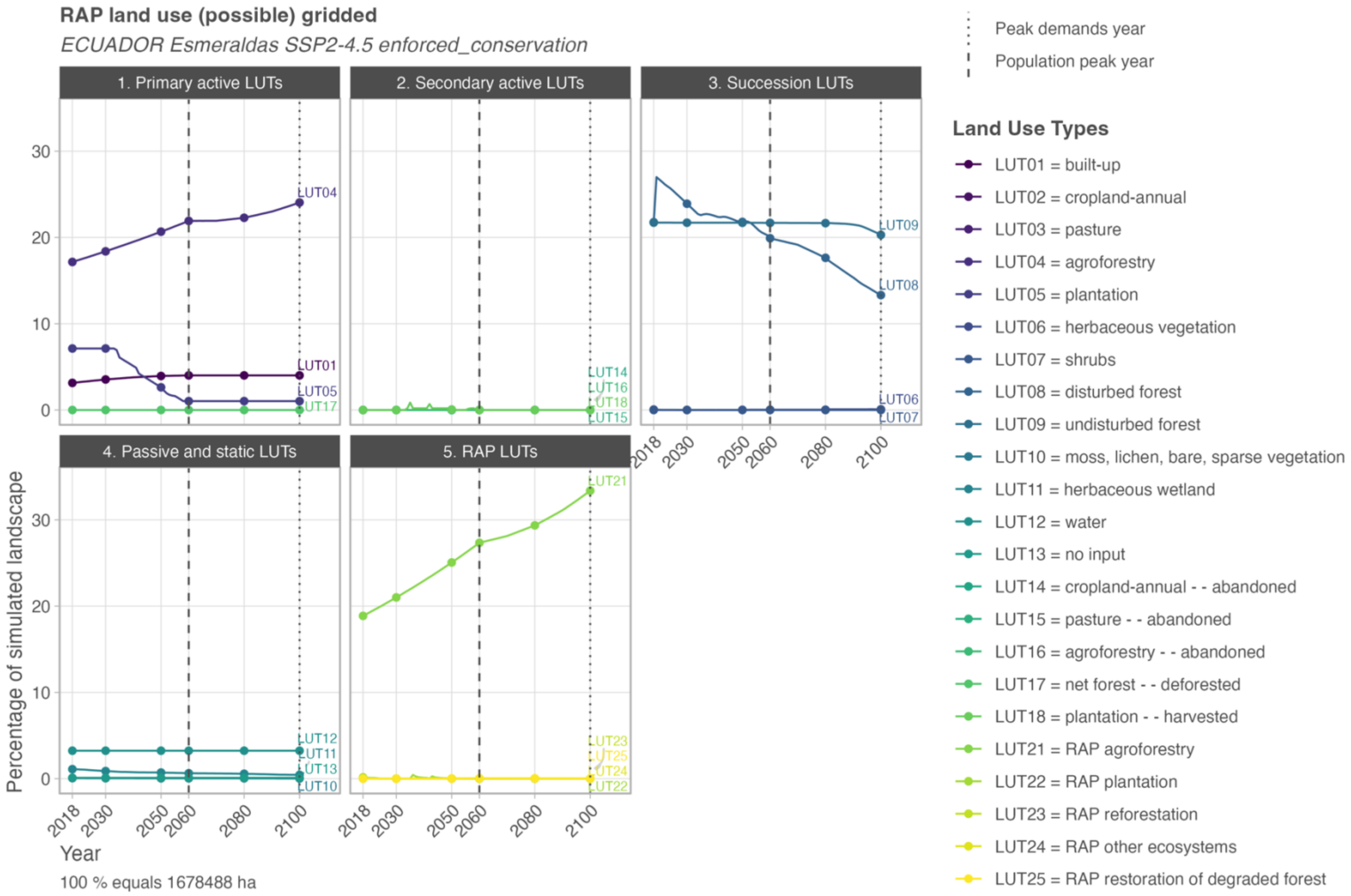

RAP land use (possible) gridded
ECUADOR Esmeraldas SSP2-4.5 enforced_conservation
1. Primary active LUTs
2. Secondary active LUTs
3. Succession LUTs
4. Passive and static LUTs
5. RAP LUTs
Percentage of simulated landscape
Year
100 % equals 1678488 ha
Peak demands year
Population peak year
Land Use Types
LUT01 = built-up
LUT02 = cropland-annual
LUT03 = pasture
LUT04 = agroforestry
LUT05 = plantation
LUT06 = herbaceous vegetation
LUT07 = shrubs
LUT08 = disturbed forest
LUT09 = undisturbed forest
LUT10 = moss, lichen, bare, sparse vegetation
LUT11 = herbaceous wetland
LUT12 = water
LUT13 = no input
LUT14 = cropland-annual - - abandoned
LUT15 = pasture - - abandoned
LUT16 = agroforestry - - abandoned
LUT17 = net forest - - deforested
LUT18 = plantation - - harvested
LUT21 = RAP agroforestry
LUT22 = RAP plantation
LUT23 = RAP reforestation
LUT24 = RAP other ecosystems
LUT25 = RAP restoration of degraded forest

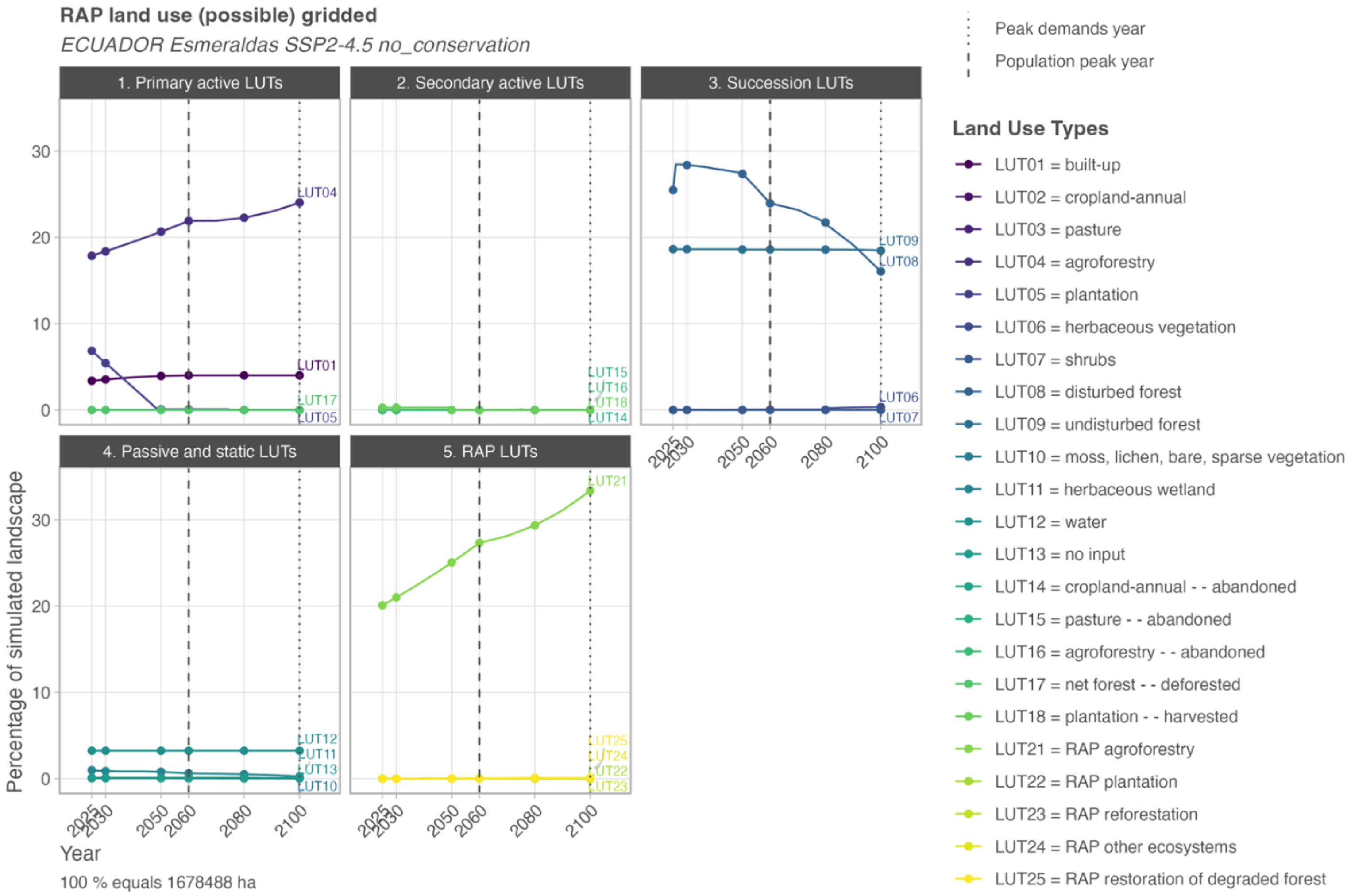

Figure D29: *The diagrams display the entire landscape configuration within the RAP simulation divided in thematic groups of land use types, here completed by the RAP LUTs section. Note that here cropland-annual and pastures are subsumed into the RAP LUT21 = agroforestry. The increase in LUT08 in the first simulation step is due to the application of the local wood extraction, which is only part of dynamic modeling in LULCC_basic. Marked are the probing dates according to the table D3 "Probing dates".*

2.2.4 RAP spatial probing dates

<table>
<tr><th>Probing date</th><th>Weak conservation</th><th>Enforced conservation</th><th>No conservation</th></tr>
<tr><td>1 (2018/2025)</td><td>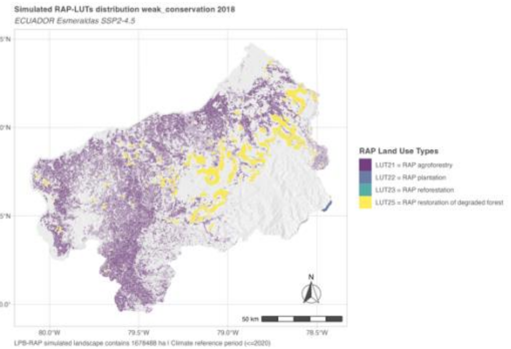
</td><td>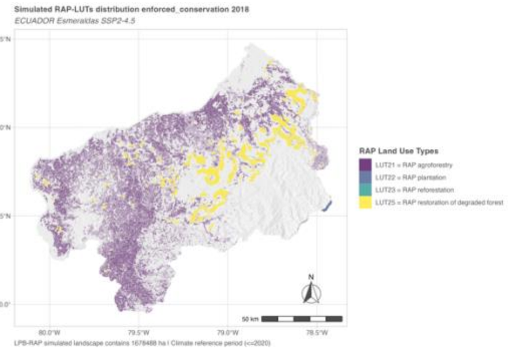
</td><td>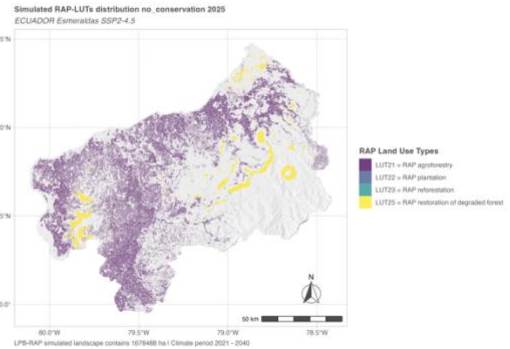
</td></tr>
<tr><td>2 (2030)</td><td>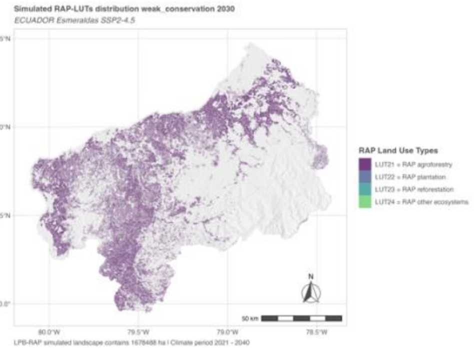
</td><td>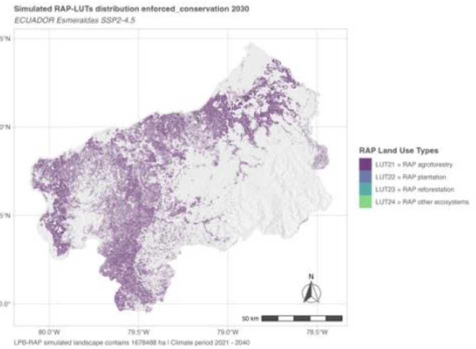
</td><td>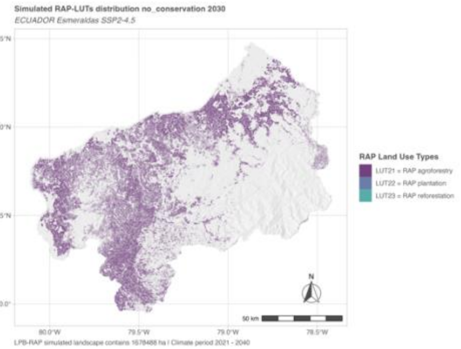
</td></tr>
</table>

3 (2050)

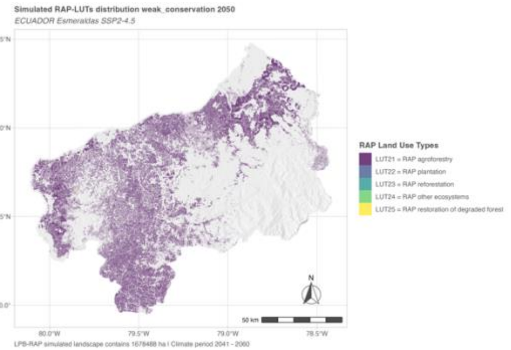

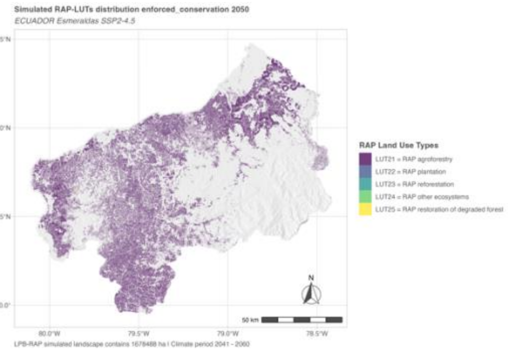

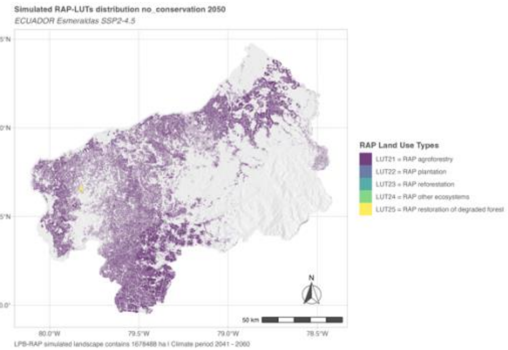

4 (2060, pp)

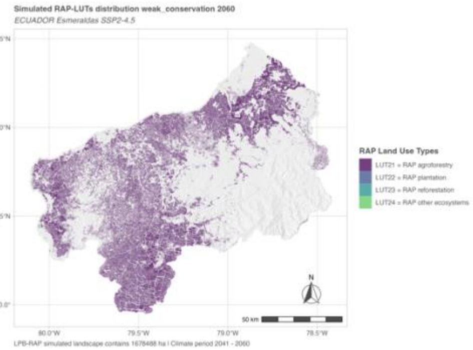

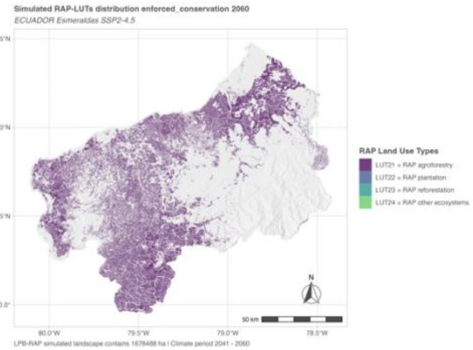

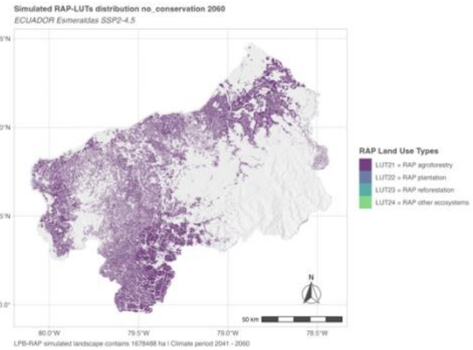

5(2080)

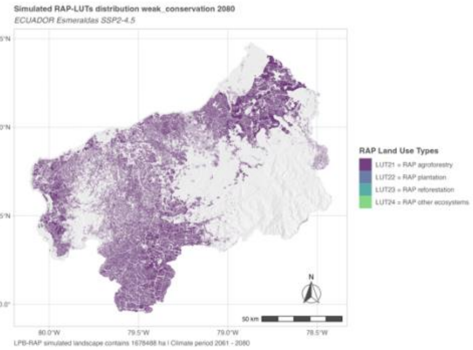

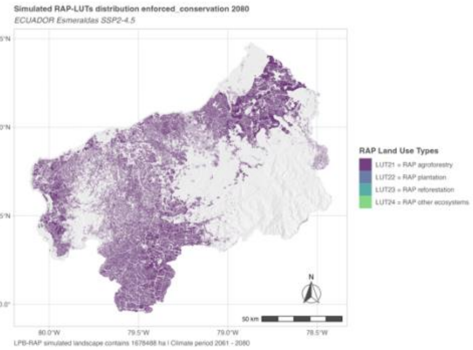

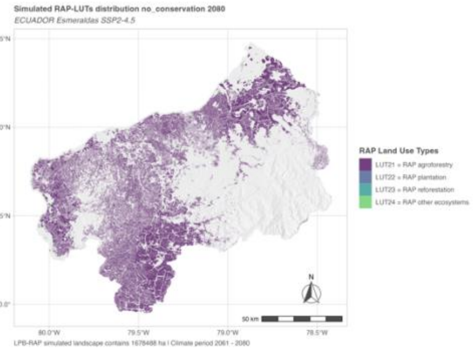

6 (2100)

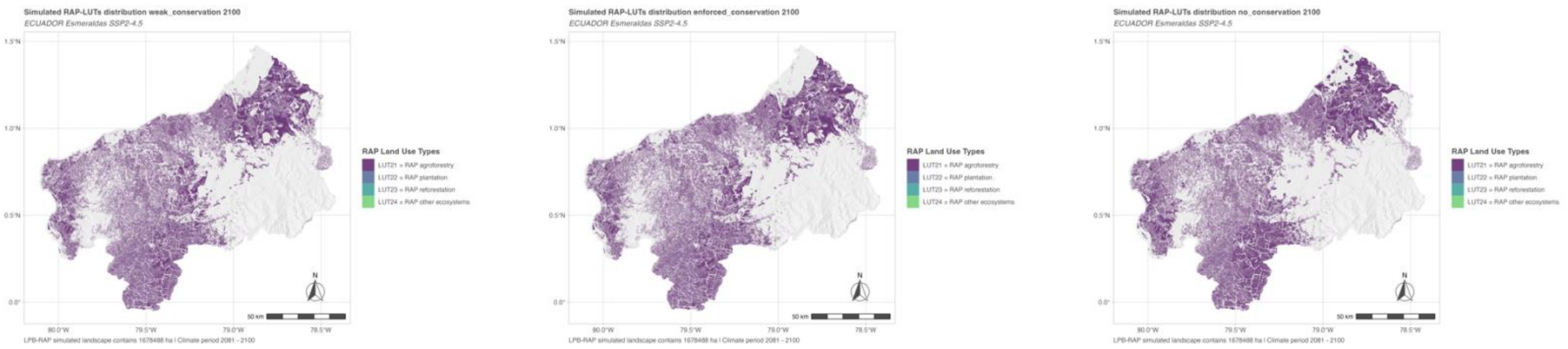

Figure D30: *The target outcome of under a restoration paradigm possible landscape configurations are displayed for the user defined probing dates in all three policy scenarios. Note that they do not depict a sequence but individual entry points into FLR or mitigation measures, since each year is simulated under the assumption, that landscape wide FLR has not been implemented yet. This is a reinterpretation of the mplc aggregated landscape in the module LULCC_RAP that uses user-defined up to five RAP-LUTs.*

2.3 RAP minimum

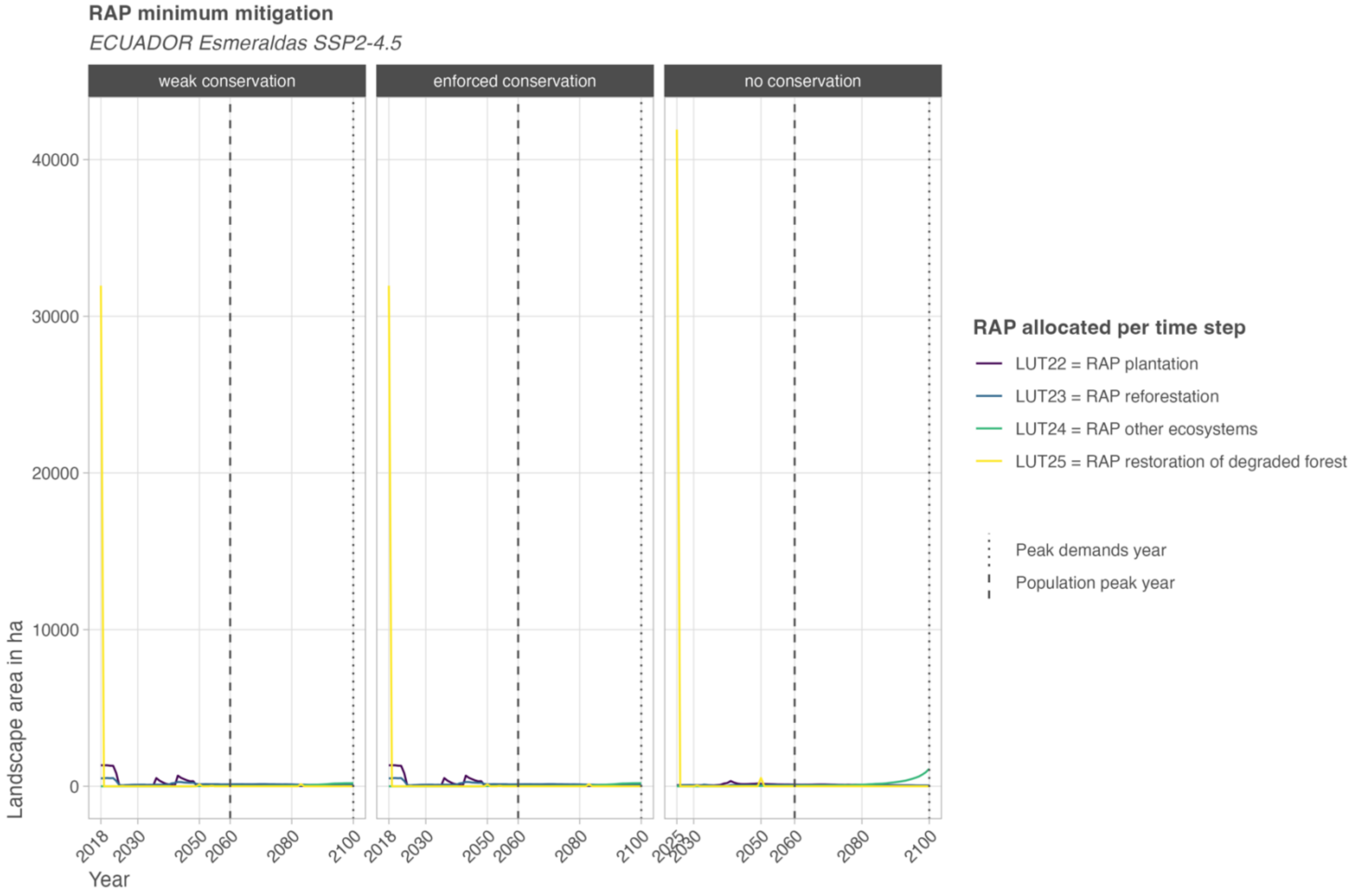

Figure D31: *The diagram enhances the aspects of FLR not reliant on the transformation of traditional farming systems which could be placed top-down without stakeholder cooperation in the conservation scenarios. Note that the initial peaks of RAP-LUT25 are simulated based on the discrepancy between the initial AGB map and the dynamic AGB map with applied scenario assumptions. This mitigation potential is very limited within scenario assumptions, which argues for more progressive measures in all political fields. Marked are the probing dates according to the table D3 “Probing dates”.*

2.4 RAP potential additional restricted areas

For the sake of policy development here are scenario simulation-based areas depicted, which could serve as additional restricted areas with a prospect of lasting results. Note that these areas display scenario-based remaining space, for the potential conservation of especially undisturbed forest additional scenario runs would have to be conducted.

<table>
<tr><th>Based on peak demands land use mask</th><th>Weak conservation/Enforced Conservation</th><th>No conservation</th></tr>
<tr><td>derived from 2100 peak demands year - applicable short-term under careful consideration</td><td>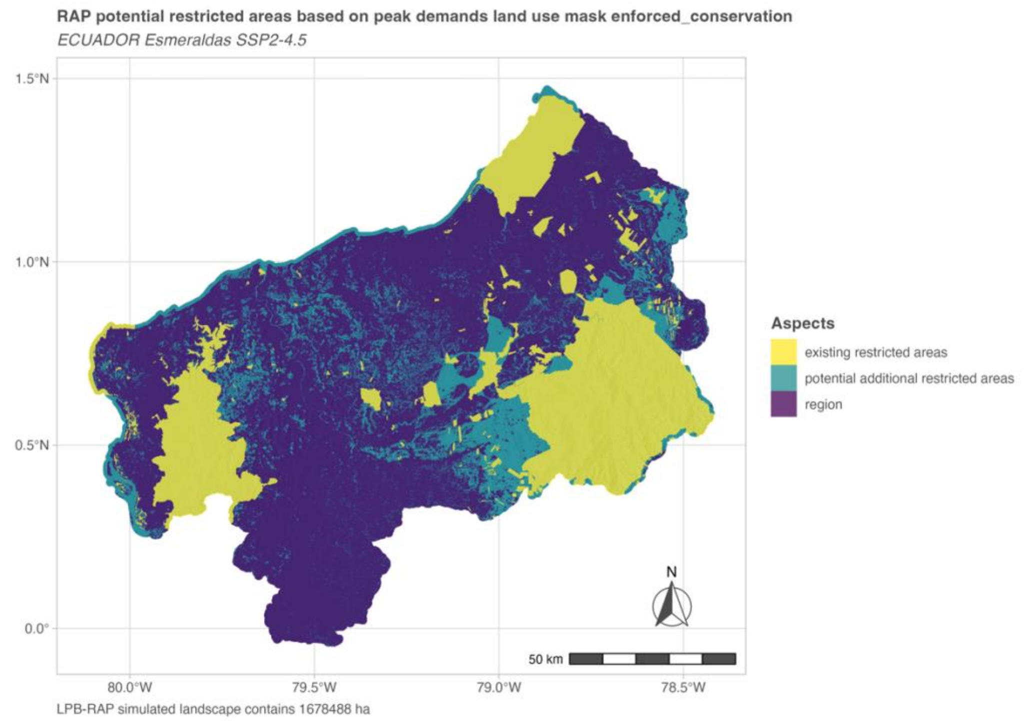
</td><td>The worst-case scenario depicts a narrative of an ensued repeal of all restricted areas top-down, therefore not applicable.</td></tr>
<tr><td>description</td><td>Existing restricted area: 414,987 ha [24.72 %]
Suggested additional restricted area: 261,660 ha
Based on the LUTs: 8, 9, 10, 11, 12, 23, 24
Potential total restricted area: 676,647 ha [40.31 %]</td><td></td></tr>
</table>

Figure D32: *The map displays potentially conflict-free conservation potentials, i.e., the areas do not collide with simulated anthropogenic demands for the peak demands year, as an extension of RAP. RAP produces an estimate for potential additional restricted areas to be implemented near-time based on user-defined input for to-be-recognized land use types and the simulated peak demands land use mask depicting simulated maximum anthropogenic area demands. The potential implementation of different degrees for conservation and allowed usage depends on local conditions. In the case of Esmeraldas, we simulated potential conservation with the maximum of potentially to be recognized land use types available in the LPB Copernicus-based portfolio.*

Discussion of selected results

For the case study area, scenario assumptions implied that restoration in the primarily by agricultural demands driven land respectively forest conversion can only be achieved via transformation of the LUTs cropland-annual and pasture to agroforestry systems on a significant level - allowing food production while increasing forest area. Reforestation, sustainable plantations, restoration of degraded forest and converted other ecosystems only play a minor role due to the steadily increasing land demand for agriculture. A decreasing demand in oil palm plantations and wood offsets this development despite increasing population and kilocalorie intake per person until the population peak (2060). However, peak demands due to long-term changes in diets are only simulated in 2100, which occurs far later than the simulated population peak.

Three guideline scenarios in combination with land use simulation were applied in restricting or not restricting land use expansions (weak conservation, enforced conservation, no conservation) within the chosen SSP2-4.5 baseline scenario. Due to the particular settings of the Esmeraldas study area, simulations resulted in only two distinctly different simulations (conservation vs. no conservation). The difference between the weak conservation and enforced conservation policy enforcement assumptions, and resulting simulated change in land use change, may still have a meaning for the investigations of plausible future landscape settings and should not be disregarded. Such land use dynamics may evolve in other landscape contexts according to different pressure conditions symbolizing limitation factors of land use, e.g. higher population or more demand, larger restricted areas share or less accessible terrain. Model results show that the imposed restrictions and land use patterns in the form of smallholder land use expansion influenced the simulated landscape trajectories within the moderate worst-case scenario, resulting in a general decline of initial forest types LUT08 and LUT09 pixels in the mplc scenario. As expected, the applied RAP scenario showed that restoration potentials for forest restoration in the form of permanent ecosystems or sustainable plantations of deforested areas are limited in this expansion scenario due to increasing demands for food production areas. This finding aligns with the recent Land Gap Report [1] illuminating that the currently pledged 1.2 billion hectares of land for carbon dioxide removal are in competition with the required land for food production. Mainly the potential for agroforestry as a mitigation measure for conventional cropland-annual and pasture farming systems would in the given scenario be a promising pathway under the considered land use change. However, this potential described per time step is in reality further limited by locally relevant aspects such as soil conditions [2], tenure aspects [3] or opportunity costs [4], which can only be evaluated for respective areas in subsequent FLR site investigations. This requires the development of an extended range of locally adapted concepts for agroforestry involving land managers and smallholders to sustain equivalent agricultural yields. Additionally, expertise and funding for the transformation and long-term maintenance of suitable concepts at plot and landscape level are required.

For the Esmeraldas region, this would, under the assumption of the continuation of the status quo, translate to restoration measures where the final tree cover would exceed the status quo of 15 or 30 %, which further requires the reinstatement of forest and other ecosystems such as herbaceous wetlands. We stress the finding that tree cover would be partially maintained in the future expansion of anthropogenic land use systems (i.e., in the simulated future distribution of the LUT04 = agroforestry) in the case of the mplc nested scenario context. However, this may come with the cost of a reduction or loss of ecosystem traits via degradation (in the range of reduced forest site characteristics, e.g., in soil parameters or microclimate and species composition in flora and fauna

associated to be present in a higher degree within the largely remote sensing based former pixels of the LUTs disturbed and undisturbed forest) and accessibility, which can only be partially mitigated in the associated RAP scenario through reforestation. The latter further calls for restoration and conservation investigations and concepts on the ground, which should not be based solely on tree or forest cover but also on other conditions such as habitat and ecosystem functionality.

We further stress the finding that peak demands hold relevant information for the realization of potential opportunities for restoration from a short- to long-term planning and policy design perspective. This is expressed primarily in the model outputs for RAP minimum and RAP suggested additional restricted areas, displaying both potential near-time and continuous action points that can be theoretically steered by top-down policy-making. Moreover, RAP maximum could be achieved over time in cooperation with and in support of smallholders and other bottom-up actors, such as NGOs or relevant forest landscape communities.

Further model findings for the case study area imply for the future that (1) the current path in this scenario of future development indicates future pressure on smallholders as indicated by increasing total land demands and expanding land use on difficult terrain; (2) the opportunity to maintain forest cover and forest with forest site characteristics is forgone by diminished forest extents; (3) the model did not suggest potential transregional leakage via unallocated regional demands during the simulated time frame (and under the assumption that smallholder land use covers subsistence needs, thereby no threatened food security for smallholders), indicating that the region could continue to be self-sufficient. The latter one however may come at the cost of ecosystem degradation, land use conversion and deforestation.

S4 File references

1. Dooley K, Keith H, Larson A, Catacora-Vargas G, Carton W, Christiansen KL, et al. The Land Gap Report 2022. Available at: https://www.landgap.org/. 2022 [cited 2022 Dec 10]; Available from: https://www.landgap.org/storage/2022/11/Land-Gap-Report_FINAL.pdf

2. Durbecq A, Jaunatre R, Buisson E, Cluchier A, Bischoff A. Identifying reference communities in ecological restoration: the use of environmental conditions driving vegetation composition. Restor Ecol. 2020;28(6):1445–53.

3. Mansourian S. Understanding the relationship between governance and forest landscape restoration. Conserv Soc. 2016;14(3).

4. Molin PG, Chazdon R, Frosini de Barros Ferraz S, Brancalion PHS. A landscape approach for cost-effective large-scale forest restoration. J Appl Ecol. 2018;55(6):2767–78.
