# Supplementary material for: Quo vadis, smallholder forest landscape? An introduction to the LPB-RAP model
Source: PLoS One. 2024 Feb 2;19(2):e0297439. doi: 10.1371/journal.pone.0297439 (PMC10836681; doi:10.1371/journal.pone.0297439)
Supplement: S5 File — Additionally, all secondary result outputs for this study are deposited. (PDF) [file pone.0297439.s005.pdf]

S5 File – GitHub repository link

Under the given link are all required model files and simulation data provided, a link to the model manual and all secondary modeling results. The repository is available after publication.

S5 File to: "Quo vadis, smallholder forest landscape? An introduction to the LPB-RAP model."

Link:

https://github.com/LPB-SDSS/LPB-RAP
